# New Function Annotation of PROSER2 in Pancreatic Ductal Adenocarcinoma

**DOI:** 10.1021/acs.jproteome.3c00632

**Published:** 2024-01-31

**Authors:** Yu-Sun Lee, Jieun Im, Yeji Yang, Hea Ji Lee, Mi Rim Lee, Sang-Myung Woo, Sang-Jae Park, Sun-Young Kong, Jin Young Kim, Heeyoun Hwang, Yun-Hee Kim

**Affiliations:** † Division of Convergence Technology, Research Institute of National Cancer Center, Goyang 10408, Republic of Korea; ‡ Department of Biomedical Science, Graduate School, Kyung Hee University, Seoul 02447, Republic of Korea; § Research Center for Bioconvergence Analysis, Korea Basic Science Institute, Cheongju 28119, Republic of Korea; ∥ Department of Cancer Biomedical Science, National Cancer Center Graduate School of Cancer Science and Policy, Goyang 10408, Republic of Korea; ⊥ Department of Center for Liver and Pancreatobiliary Cancer, National Cancer Center, Goyang 10408, Republic of Korea; # Department of Surgical Oncology Branch, Research Institute of National Cancer Center, Goyang 10408, Republic of Korea; ∇ Department of Targeted Therapy Branch, Research Institute of National Cancer Center, Goyang 10408, Republic of Korea; ○ Critical Diseases Diagnostics Convergence Research Center, Korea Research Institute of Bioscience and Biotechnology, Daejeon 34141, Republic of Korea

**Keywords:** pancreatic ductal adenocarcinoma, uncharacterized protein evidence level 1, proline and serine-rich 2, invasion, proliferation, serine/threonine-protein kinase 25, adenosine monophosphate-activated protein kinase

## Abstract

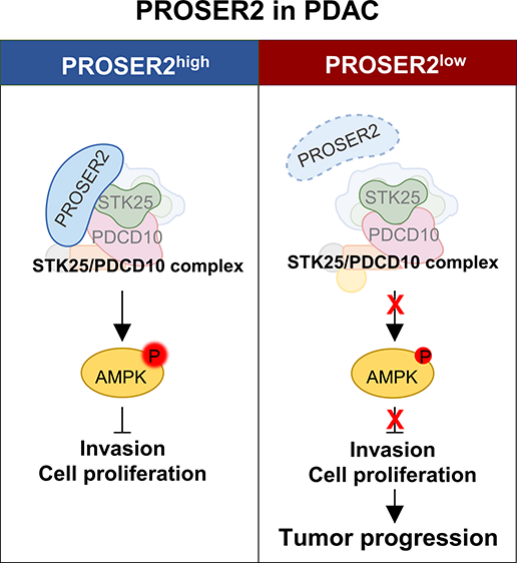

Pancreatic ductal adenocarcinoma (PDAC) has a dismal prognosis due to the absence of diagnostic markers and molecular targets. Here, we took an unconventional approach to identify new molecular targets for pancreatic cancer. We chose uncharacterized protein evidence level 1 without function annotation from extensive proteomic research on pancreatic cancer and focused on proline and serine-rich 2 (PROSER2), which ranked high in the cell membrane and cytoplasm. In our study using cell lines and patient-derived orthotopic xenograft cells, PROSER2 exhibited a higher expression in cells derived from primary tumors than in those from metastatic tissues. PROSER2 was localized in the cell membrane and cytosol by immunocytochemistry. PROSER2 overexpression significantly reduced the metastatic ability of cancer cells, whereas its suppression had the opposite effect. Proteomic analysis revealed that PROSER2 interacts with STK25 and PDCD10, and their binding was confirmed by immunoprecipitation and immunocytochemistry. STK25 knockdown enhanced metastasis by decreasing p-AMPK levels, whereas PROSER2-overexpressing cells increased the level of p-AMPK, indicating that PROSER2 suppresses invasion via the AMPK pathway by interacting with STK25. This is the first demonstration of the novel role of PROSER2 in antagonizing tumor progression via the STK25-AMPK pathway in PDAC. LC–MS/MS data are available at MassIVE (MSV000092953) and ProteomeXchange (PXD045646).

## Introduction

The 5-year survival rate of pancreatic cancer has increased slightly in recent years and ranges from 2 to 11%. However, this increase is largely limited to patients with early-stage localized pancreatic cancers; for the over 70% of patients with pancreatic ductal adenocarcinoma (PDAC) who are diagnosed at advanced stages, the survival rate remains in the low single digits at 3%.^1,2^ Therefore, pancreatic cancer remains a challenging disease with a low 5-year survival rate, primarily due to the absence of effective early diagnostic markers and limited treatment options. Several potential diagnostic markers, including CA 19–9, CEA, ADH/MIC-1, and osteonectin, have been studied, but most of them lack the sensitivity and specificity needed for use as prognostic indicators.^3−5^ Additionally, the absence of specific molecular markers for pancreatic cancer growth and progression, apart from the G12C KRAS mutation target inhibitor developed recently, has made targeted therapy ineffective.^6^

Although pancreatic cancers are characterized by early invasion and aggressive metastasis, research on the development of molecular markers and deciphering mechanisms specific to advanced-stage pancreatic cancers has been limited. This limitation is attributed to several factors, including inaccessibility to samples for research due to the inoperable nature of 70% of advanced pancreatic cancers, analysis of clinical outcome correlated with molecular marker on gene expression levels, and the limited identification of molecules with functional importance that are not included in existing databases.^7,8^ In this context, the function annotation of uncharacterized protein evidence level 1 (uPE1) proteins could offer a promising approach to uncover new mechanisms underlying cancer progression.^9^

uPE1 refers to uncharacterized proteins that have been detected at the proteome level but whose functions have not yet been identified. After the initiation of the Human Proteome Project (HPP), significant progress has been made in registering more than 90% of protein-coding genes at the proteome level. However, despite various protein function annotation methods based on different data types (sequence, protein interactions, coexpression, etc.), there is no evidence for the functions of a substantial proportion (13%) of detected proteins. The human protein database neXtProt (http://nextprot.org) curates and documents uPE1 proteins in a chromosome-specific manner as part of the HPP of the Human Proteome Organization (HUPO). According to the latest version of neXtProt (April 2023), 18,397 proteins have been classified as protein evidence level 1 (PE1) from human genes encoding 20,389 protein entries, based on mass spectrometry and/or antibody-based analysis.^10^ Among these, 1191 uPE1 proteins can be searched and downloaded from the neXtProt Web site using the SPARQL query “NXQ_00022”.^11^

In this study, 181 uPE1 proteins were selected from large-scale proteogenomic research on PDAC, which were identified and quantified. Among the top-ranked molecules based on number of transcripts per million (nTPM) values in tissue expression data from the Human Protein Atlas and cell compartment scores in gene ontology (GO), PROSER2 was chosen for further investigation. PROSER2, also known as chromosome 10 open reading frame 47 (C10orf47), is a protein encoded by the human *PROSER2* gene. It is located on band 14 of the short arm (10p14) of chromosome 10 and includes a highly conserved SARG domain. According to data from The Human Protein Atlas database (https://www.proteinatlas.org), its expression levels were found to be associated with pancreatic cancer survival rates. Furthermore, PROSER2 has been identified as a potential biomarker in epithelial cell, breast, prostate, ovarian, lung, brain, and hematological cancers.^12,13^ It is highly expressed in both benign and malignant bone tumors and is considered to be a factor in poor prognosis. PROSER2 also interacts with several other proteins associated with cell death, germ cell differentiation, chromatin maintenance, cell integrity, and cell cycle progression, although its specific function and mechanism have not yet been fully elucidated.

This study was aimed at determining the function of PROSER2 in PDAC and unraveling the underlying mechanism using a multidisciplinary approach. We evaluated the expression of PROSER2 in pancreatic cancer cell lines and patient-derived orthotopic xenograft cells (PDOXc). Furthermore, its role as a regulator inhibiting the growth and invasion of pancreatic cancer cells was elucidated. We identified that PROSER2 can bind to STK25 and PDCD10 complexes from proteomics, and we proposed and validated the STK25-AMPK pathway as the mechanism regulating the function of PROSER2. This study lays the foundation for inferring the functional mechanisms of PROSER2 and deciphering novel mechanisms for the regulation of pancreatic cancer metastasis in the future. The study also underscores the possibility that uPE1 proteins play important roles in cancer progression that can potentially contribute to the development of diagnostic markers and targeted therapies for challenging diseases.

## Experimental Procedures

### Cell Culture

The human PDAC cell lines BxPC-3, MIA PaCa-2, HPAF-II, AsPC-1, and Capan-2 were purchased from the American Type Culture Collection (ATCC; Manassas, VA, USA), whereas SNU-324, SNU-213, and SNU-410 cells were purchased from the Korean Cell Line Bank (KCLB; Seoul, Korea).

The BxPC-3, SNU-324, SNU-213, MIA PaCa-2, HPAF-II, AsPC-1, and SNU-410 cells were cultured in RPMI-1640 (Hyclone, South Logan, UT, USA). MIA PaCa-2 and PANC-1 cells were cultured in Dulbecco’s modified Eagle medium (DMEM, Hyclone), HPAF-II cells in Eagle’s Minimum Essential Medium (EMEM, Gibco), and Capan-2 cells in McCoy’s 5a modified medium (Gibco Thermo Fisher Scientific, Waltham, MA, USA). All of the culture media were supplemented with 10% fetal bovine serum (FBS; Gibco) and 1% antibiotic-antimycotic solution (Gibco). Capan-1 cells were also cultured in Iscove’s Modified Dulbecco’s Medium (IMDM, Gibco) supplemented with 20% FBS and 1% antibiotic-antimycotic solution.

PDOXc were isolated from tumor tissues of patient-derived orthotopic xenograft (PDOX) models: SPDOXc from PDOX with surgically obtained primary pancreas tumor tissue and GPDOXc from PDOX with ultrasonic-guided gun biopsy of liver metastasized tumor tissues. Details regarding the establishment of PDOXc and culture method for these cell samples were described in detail previously.^14^ Both SPDOXc and GPDOXc were cultured in RPMI-1640 (Hyclone) medium supplemented with 10% heat-inactivated FBS and a 1% ZellShield (Minerva Biolabs, Berlin, Germany).

### Western Blot Analysis

PDAC cell lines and PDOXc were lysed in RIPA buffer (Biosesang, Sungnam, Korea) containing a protease and phosphatase inhibitor cocktail (Thermo Fisher Scientific). The protein samples were electrophoresed at 100 V on 4–20% gradient SDS-PAGE gels (Thermo Fisher Scientific) and transferred to polyvinylidene fluoride (PVDF) membranes (GE Healthcare Amersham Biosciences, Uppsala, Sweden). The membranes were incubated at 4 °C for 18 h with primary antibodies against PROSER2 (Santa Cruz Biotechnology, TX, USA), DDK (Cell Signaling Technology, Berkeley, CA, USA), and tubulin (Sigma-Aldrich, St. Louis, MO, USA). After washing, the membranes were incubated with HRP-conjugated antimouse-HRP (GenDEPOT, Barker, TX, USA) or antirabbit-HRP (GenDEPOT), and the blots were subsequently developed using Amersham ECL Prime Western Blotting Detection Reagent (GE Healthcare Amersham Biosciences).

### Immunofluorescence Staining

To determine the localization of PROSER2, cells were seeded in a 96-well plate (1 × 10^4^ cells/well) and cultured for 24 h. After washing with PBS, the cells were fixed with 4% paraformaldehyde, incubated with 1:50 diluted anti-PROSER2 (Santa Cruz Biotechnology) at 4 °C for 18 h, followed by incubation with Alexa Flour-488 conjugated antimouse IgG (Invitrogen, Oregon, USA) at 25 °C for 1 h, and staining with DAPI using Hoechst 33342 (Invitrogen). The cells were imaged using an Operetta CLS High Content Imaging System (PerkinElmer Operetta, Waltham, MA, USA), and the numbers and locations of stained cells from 40 fields in two wells were determined using Harmony 4.5 software (PerkinElmer).

To determine the colocalization of PROSER2 and STK25, cells were seeded on poly-l-lysine (Sigma-Aldrich) coated 8-well chamber slides (Thermo Fisher Scientific) at a density of 1 × 10^4^ cells/well and cultured for 24 h. After washing with PBS, the cells were fixed in 4% paraformaldehyde and incubated with 1:50 diluted anti-PROSER2 (Santa Cruz Biotechnology) and 1:100 diluted rabbit anti-STK25 (Abcam, Cambridge, MA, USA) or 1:100 diluted anti-PDCD10 (ProteinTech Group, Beijing, China) at 4 °C for 18 h. Subsequently, the slide was incubated with Alexa Flour-633 conjugated antimouse IgG and Alexa Flour-546 conjugated antirabbit IgG at 25 °C for 1 h. The images showing the colocalization of PROSER2 and STK25 and that of PROSER2 and PDCD10 were captured using a confocal microscope (LSM780; Cark Zeiss, Jena, Germany), and the intensity of colocalization of PROSER2 with STK25 or PDCD10 was determined using the ZEN blue software (Carl Zeiss) from merged images. Colocalization was analyzed by quantifying pixel counts and areas from the background, as Mander’s overlap coefficient (MOC) from merged images. MOC was calculated using the following equation: 

### Lentivirus Preparation and Cell Transduction

Human PROSER2 cDNA (Origene Technologies, MD, USA) was subcloned into a Myc-DDK-tagged pHRST-IRISeGFP vector. For lentivirus packaging, 2 μg of this plasmid, 3 μg of pMD2.G, and 4 μg of psPAX2 were transfected into HEK-293T cells using LipoEZ (Aptabio Therapeutics, Suwon, Korea) with 5 mL of DMEM, supplemented with 10% FBS (Gibco), sodium pyruvate (WelGENE Inc., Daegu, Korea), HEPES (Gibco), nonessential amino acid culture supernatant (NEAA, Gibco), and GlutaMAX (Gibco). After 18 h, 4 μg of caffeine (Sigma-Aldrich) was added, and the incubation was continued for an additional 18 h. Culture medium containing lentivirus was harvested and concentrated 10-fold using a lenti-X concentrator (Clontech-Takara, Saint-Germainen-Laye, France). For generating the PROSER2-overexpressing cell line, MIA PaCa-2 cells were transfected with lentivirus-containing polybrene (8 μg/mL) and incubated for 48 h. Transfected cells were sorted by GFP fluorescence using flow cytometry. PROSER2-knockdown and STK25-knockdown cells were seeded in 6-well plates (5 × 10^5^ cells/well) and cultured for 18 h, after which they were transfected with 50 μM of PROSER2 siRNA (Santa Cruz Biotechnology), 50 μM of STK25 siRNA (Origene Technologies, Inc., Rockville, MD, USA), or control siRNA for 48 h using LipoEZ (Aptabio Therapeutics). The knockdown of the respective gene in these cells was confirmed using Western blotting.

### Invasion and Proliferation Assay

Invasion assays were performed using a Boyden chamber (8 μm pore size) coated with 100 μg of reduced growth factor Matrigel (Thermo Fisher Scientific) in 24-well plates. Briefly, cells were seeded at 1 × 10^5^ cells/well in the upper chamber with 200 μL serum-free medium. Six hundred microliters of complete medium supplemented with 10% FBS was added to the lower chamber, followed by incubation at 37 °C for 48 h. The invading cells were fixed, stained with Diff-Quick solution (Sysmex, Japan), and observed under a microscope. The average count in at least five fields was determined, and the invasive cells were quantified using ImageJ 1.53k software.

For the cell growth assay, PROSER2-overexpressing MIA PaCa-2 cells (1 × 10^4^ cells/well), PROSER2-knockdown SNU-213 cells (1 × 10^4^ cells/well), or SNU-410 cells (2 × 10^3^ cells/well) were seeded in 96-well plates and cultured for 18 h. Cell proliferation was measured every 6 h during 60 to 100 h using the IncuCyte Live Cell Analysis System (Sartorius, USA). The percent of cell growth was the average of six wells and normalized to the values on day 1.

### Sample Preparation for Mass Spectrometry Analysis

Cells were lysed with 1× sodium dodecyl sulfate (SDS) buffer (5% SDS, 50 mM triethylammonium bicarbonate (TEAB), pH 8.5). Aliquots containing 300 μg of proteins were reduced and alkylated with 10 mM Tris (2-carboxyethyl) phosphine (TCEP) and 20 mM indole 3-acetic acid (IAA), respectively. The proteins were digested with 15 μg aliquots of mass-spectrometry-grade Trypsin Gold (Promega, Madison, WI) using S-trap mini digestion kits (ProtiFi, Huntington, NY), according to the manufacturer’s protocol. The final eluted samples were dried in a speed vacuum, and the concentrations of the dried peptides were quantified with a Pierce quantitative colorimetric peptide assay kit (Thermo Fisher Scientific). Aliquots containing 100 μg of peptides were labeled with TMT 10-plex isobaric label reagent kits (Thermo Fisher Scientific). The samples were pooled, dried, and desalted using Pierce peptide desalting spin columns (Thermo Fisher Scientific). Total peptides were fractionated into 20 fractions using reverse-phase liquid chromatography, and each eluted peptide sample was vacuum-dried. The fractionated peptides were diluted with mobile phase A (99.9% water and 0.1% formic acid) for liquid chromatography-tandem mass spectrometry (LC–MS/MS) analysis.

### LC–MS/MS Analysis

Equal volumes containing 1 μg of each peptide fraction were analyzed using an Orbitrap Fusion Lumos mass spectrometer (Thermo Fisher Scientific) coupled to an Easy nLC device (Thermo Fisher Scientific). Samples were loaded onto a PepMap C18 column (particle size of 2 μm; pore size of 100 Å; internal diameter of 75 μm; length of 50 cm; Thermo Fisher Scientific), which was developed for 120 min at a flow rate of 0.25 μL/min. The column was developed by elution with 5–7% buffer B (80% acetonitrile (ACN) in 0.1% formic acid) for 5 min, 7–25% buffer B for 78 min, 25–40% buffer B for 13 min, 40–95% buffer B for 5 min, and 95% buffer B for 8 min. The column was subsequently washed with 5% B for 11 min. The full scan resolution was 120,000 at *m*/*z* 400. The maximum ion injection times for the full scan and MS/MS scans were 100 and 118 ms, respectively. The scan range was 400–2000 *m*/*z*, and the MS2 scans were performed with HCD fragmentation (37.5% collision energy). The electrospray voltage was maintained at 2.0 kV, and the capillary temperature was set at 275 °C.

### Data Processing and Bioinformatic Analysis

Raw MS data were converted to an MS2 file using RawConverter (v. 1.1.0.18, The Scripps Research Institute, La Jolla, CA, USA) and analyzed using the Integrated Proteomic Pipeline (IP2, v. 6.5.5, Bruker) platform against neXtProt fasta DB (2022-feb) modified from the same version of a.peff file. Trypsin protease was set as the digestion enzyme, and a maximum of two missed cleavages were allowed. Initial precursor mass deviation was up to 20 ppm and fragment mass tolerance was 20 ppm. The false positive rate for the spectrum was 1%, and proteins were identified with two or more unique peptides, where the estimated false discovery rate (FDR) was 0.22, 0.26, and 2.08% at the spectra, peptide, and protein level, respectively. Protein quantification was performed by the census in the IP2 pipeline using the TMT 10-plex isobaric ions with a 20 ppm tandem tolerance. GO analysis was conducted using the DAVID functional annotation tool (https://david.ncifcrf.gov/tools.jsp) and pathway analysis was performed using STRING DB (https://string-db.org/).

### Immunoprecipitation

PROSER2 was purified from among the total proteins in PROSER2-overexpressing MIA PaCa-2 cells using Myc tagging. Cells were lysed with a buffer containing 50 mM Tris–HCl (pH 7.4), 150 mM NaCl, 1 mM EDTA, 1% Triton-X 100, and a protease and phosphatase inhibitor cocktail (Thermo Fisher Scientific). Thereafter, 1 mg of cell proteins was incubated with 50 μL of anti-FLAG-M2 magnetic beads (Thermo Fisher Scientific) for 18 h at 4 °C under constant shaking at 15 rpm. After the beads were washed with a buffer containing 50 mM Tris–HCl (pH 7.4), 150 mM NaCl, and 1 mM EDTA, PROSER2 was immunoprecipitated with an elution buffer containing 3× FLAG peptide (Sigma) and confirmed by immunoblotting using an anti-FLAG antibody.

### Statistics

The *p*-value was calculated using the student’s *t* test. All statistical analysis were performed using the GraphPad Prism software (version 8.4.3). *p < 0.05, **p < 0.01, and ***p < 0.001.

## Results

### Selection of uPE1 Candidates Based on Proteogenomic Study of PDAC

**Figure 1:**
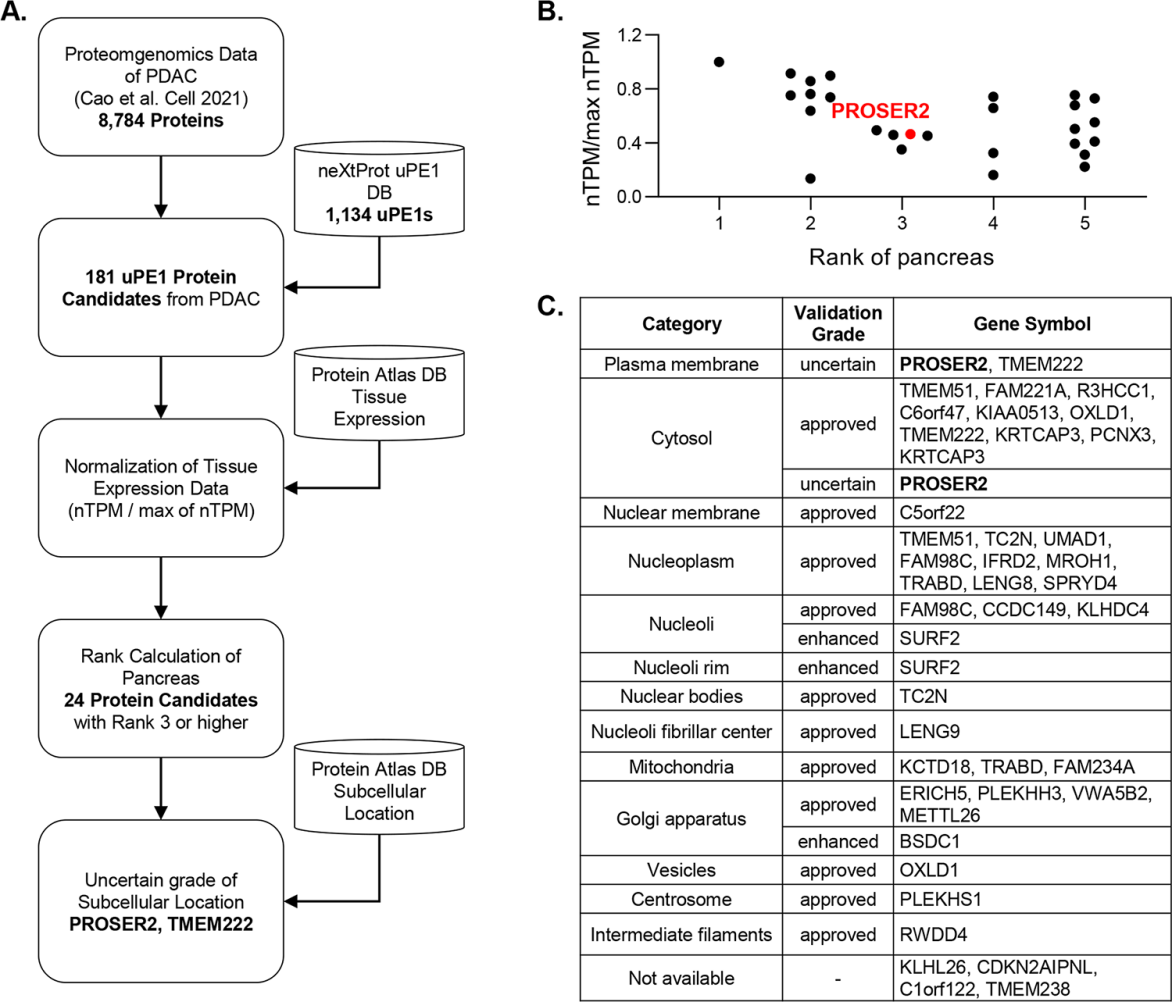
Selection of uPE1 candidates from a proteogenomics study of pancreatic ductal adenocarcinoma (PDAC). (A) A workflow for uPE1 selection from large-scale proteomics data. (B) uPE1 candidates with a high rank and normalized number of transcripts per million (nTPM) value in the pancreas from the Human Protein Atlas (https://proteinatlas.org). The red spot indicates the rank and normalized nTPM value for PROSER2. (C) Table listing the subcellular localization of 24 uPE1 candidates with rank 3 or higher. The validation grade and category are supported by the Human Protein Atlas.

To select uPE1 candidates from 1,191 uPE1 documented in the neXtProt database (https://www.nextprot.org/) for function annotation study, we selected 181 uPE1 from one of the large scale proteogenomic studies on PDAC with 8784 identified and quantified proteins (Table S1, Figure 1A).^15^ From the Human Protein Atlas database, we extracted the nTPM values for 40 tissue expression data for 181 uPE1 candidates, and divided them by the maximum value for normalization. Using the normalized nTPM value and the rank of the nTPM value, we selected 24 protein candidates with a third or higher rank and a 0.4 or more normalized nTPM value (Figure 1B). Then we searched for the subcellular localization of the candidate proteins and found that PROSER2 has “uncertain” grade plasma membrane and cytosol, and we could start a function annotation study on PROSER2 with validation of its subcellular localization (Figure 1C). According to the latest neXtProt database (released in September, 2023), D-I-TASSER and COFACTOR predict PROSER2 to have a high CC score of intracellular (1.0) and cytoplasmic (0.79) parts in the GO term of cellular components.^16−18^

### PROSER2 Inhibited Invasion and Proliferation in PDAC

**Figure 2:**
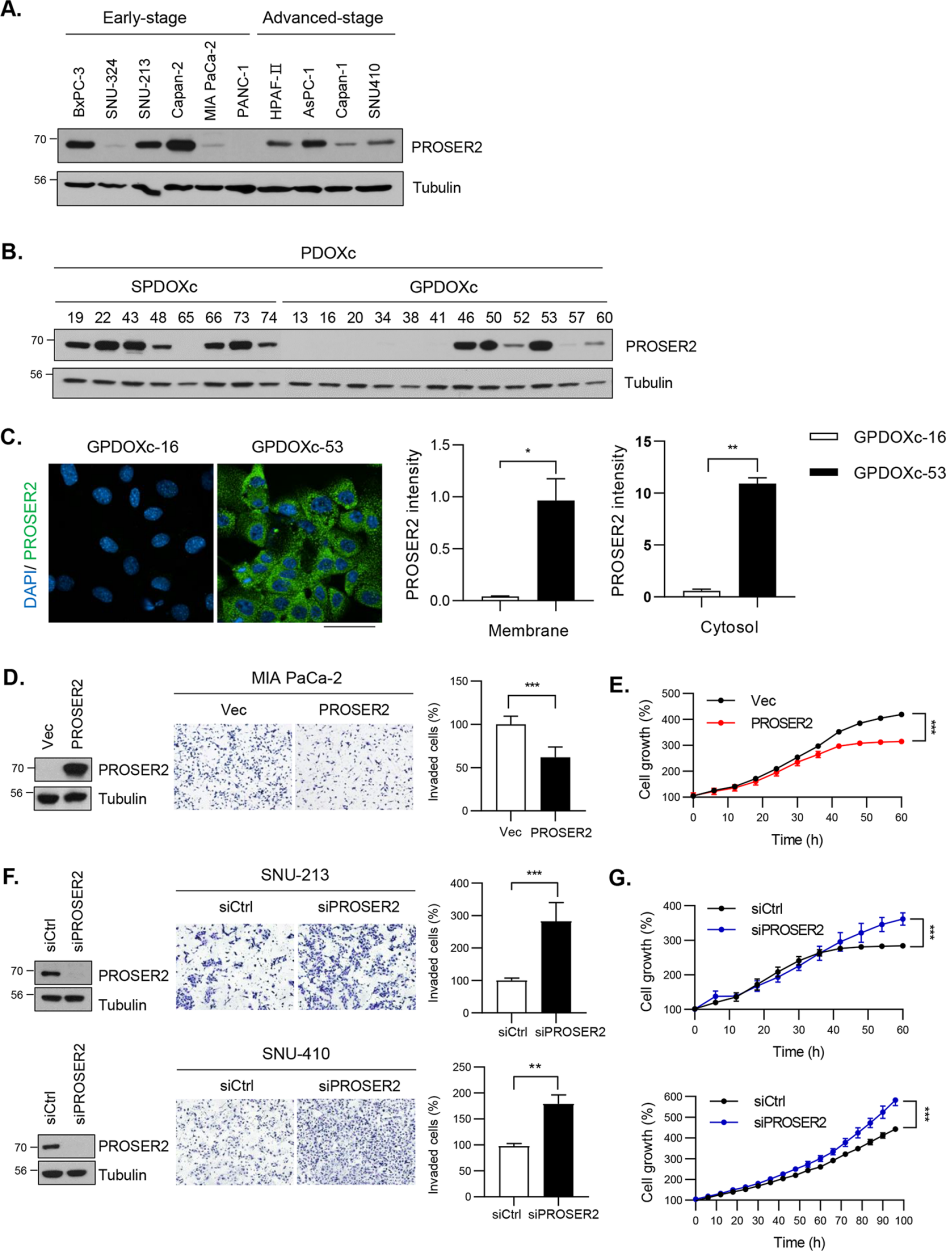
Effect of PROSER2 expression on pancreatic adenocarcinoma (PDAC) cells. (A) Evaluation of PROSER2 expression in PDAC cell lines and (B) PDOXc as assessed using Western blot analysis. (C) Representative confocal micrographs of PROSER2 (green) in GPDOXc-16 and GPDOXc-53 (scale bar = 50 μm). The intensity of PROSER2 expression in membrane or cytoplasmic regions was analyzed using the Harmony 4.5 software. The graphs are present as mean ± SD (*n* = 40). (D) Invasion analysis in the PROSER2-overexpressing MIA PaCa-2 cell line. The cell line was verified using Western blot analysis and invaded cells were quantified using five randomly selected images (×10 magnification) with the ImageJ software. The graphs are present as mean ± SD (*n* = 5). (E) Cell proliferation assay using the PROSER2-overexpressing MIA PaCa-2 cell line and quantification by cell area from images captured over 80 h. The graphs are present as mean ± SD (*n* = 6). (F) Cell invasion in the PROSER2-knockdown SNU-213 and SNU-410 cell lines. PROSER2-knockdown cell lines were transfected with siCtrl or siPROSER2 and verified using Western blot analysis. (G) Cell proliferation assay using the PROSER2-knockdown SNU-213 and SNU-410 cell lines and quantification by cell growth area from images captured over 60 or 100 h. *p < 0.05, **p < 0.01, and ***p < 0.001.

The expression of PROSER2 in cell lines and PDOXc was detected to determine its association with tumor progression. PROSER2 was strongly expressed in almost all PDAC cell lines from early-stage patients (BxPC-3, SNU-213, and Capan-2) but weakly expressed in cell lines from advanced-stage patients (AsPC-1, HPAF-II, Capan-1, and SNU-410) (Figure 2A). PROSER2 was also strongly expressed in all SPDOXc, except HPDOXc-65. In contrast, PROSER2 was nearly undetectable in the majority of GPDOXc, except in three (GPDOXc-46, 50, and 53) out of 12 GPDOXc samples (Figure 2B). Thus, PROSER2 expression was higher in cells from primary tumor tissue than in those from metastasized tumor tissue, which indicates that it may be associated with tumor progression, especially metastasis.

Immunofluorescence staining of GPDOXc for PROSER2 showed an intense signal in the membrane and cytosol regions in GPDOXc-53 (a PROSER2-positive cell line) compared with that in GPDOXc-16 (a PROSER2-negative cell line), indicating that PROSER2 localizes to the membrane as well as the cytosol (Figure 2C).

Next, we examined the function of PROSER2 in PDAC progression using invasion and proliferation assays with PROSER2-overexpressing (MIA PaCa-2) and PROSER2-knockdown (SNU-213 and SNU-410) cells. Overexpression of PROSER2 significantly inhibited the invasion and proliferation of MIA PaCa-2 cells (Figure 2D,E). Compared with that in the empty vector (Vec) group, cell invasion and proliferation were decreased by 40 and 75%, respectively, in the overexpression group. In contrast, knockdown of PROSER2 in SNU-213 and SNU-410 dramatically promoted cell invasion approximately by 150% and 80%, and also increased proliferation by 80% compared with that in the control group (siCtrl) (Figure 2F,G). These results indicate that PROSER2 inhibited cancer progression in PDAC.

### Proteomic Analysis of PROSER2-Overexpressing MIA PaCa-2 Cells

**Figure 3:**
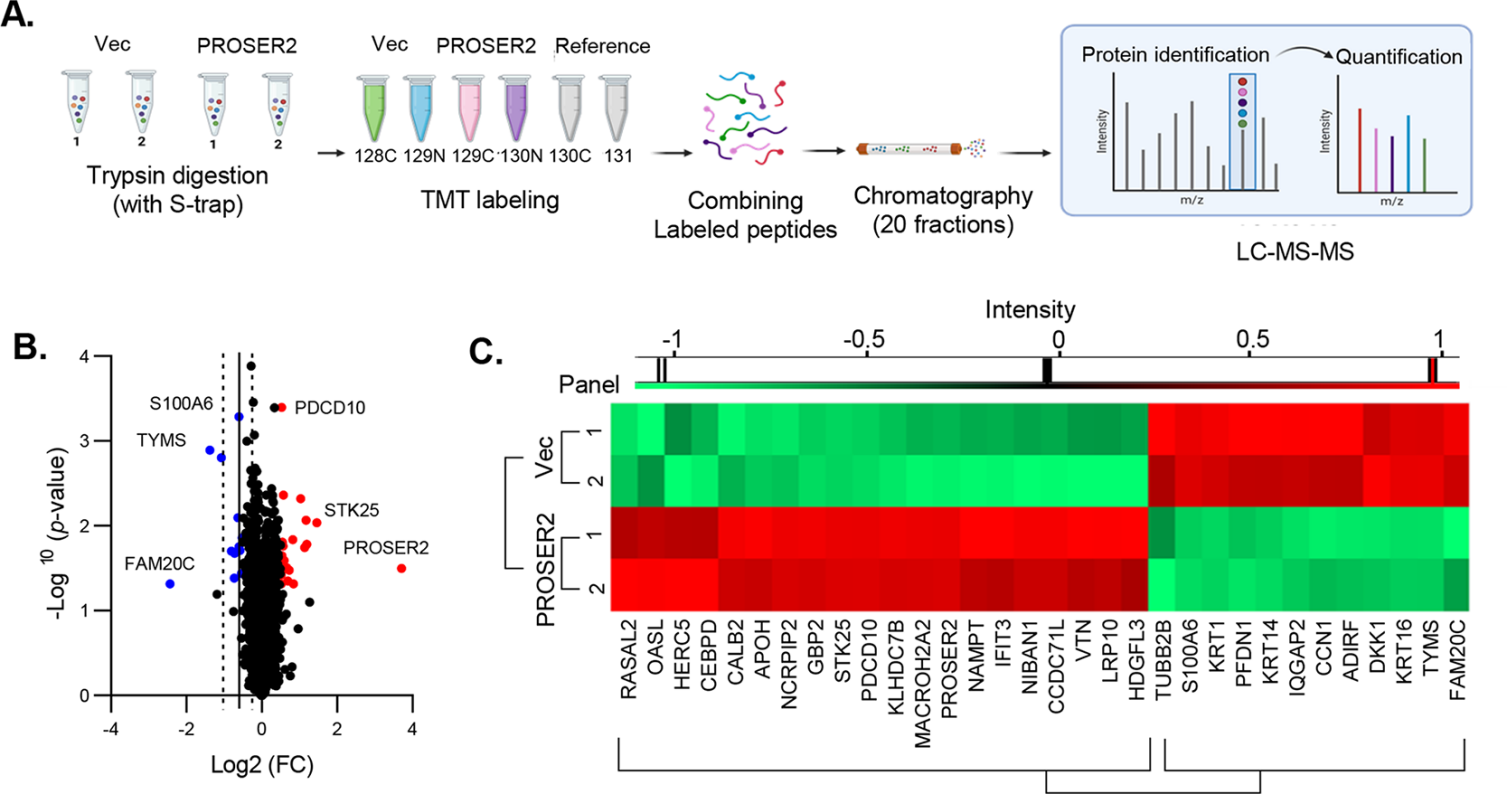
Identification of PROSER2-regulated proteins using LC–MS/MS analysis. (A) Schematic of LC–MS/MS analysis of MIA PaCa-2 cells. (B) Volcano plot of proteins identified using LC–MS/MS analysis. Among the proteins, 12 were downregulated in PROSER2-expressing cells (indicated with blue dots), whereas 20 were upregulated (indicated with red dots) based on fold change (|log2FC|) > 0.5 and *p* < 0.05. (C) Heatmap showing differentially expressed proteins (DEPs) between PROSER2-overexpressing and blank vector-transfected cells. *Z*-score shows normalized protein intensity, with high (red) and low (green) expression levels.

To elucidate the mechanism through which PROSER2 reduced cell invasion and proliferation, we analyzed the interacting partners of PROSER2 in Flag-PROSER2-overexpressing MIA PaCa-2 cells using LC–MS/MS. For quality control, duplicated references pooled from all samples were used, and the samples were trypsin-digested and labeled with a tandem mass tag (TMT). To maximize the number of identified proteins, combined samples were fractioned into 20 tubes using basic reverse-phase liquid chromatography, followed by combined analyses using Orbitrap Fusion mass spectrometry (Figure 3A). A total of 4889 proteins were identified in the control (Vec) and PROSER2-overexpression groups (Table S2). The expression of PROSER2 was 10-fold higher in overexpressing cells than in the control cells (|log2FC| = 3.71, *p* < 0.05). Additionally, 20 proteins, including STK25 and PDCD10, were upregulated, and 12 proteins, including family with sequence similarity member 20C (FAM20C), thymidylate synthase (TYMS), and S100-calcium-binding protein A6 (S100A6), were downregulated (|log2FC| > 0.5 and *p* < 0.05) in overexpressing cells (Figure 3B, Table S3). Hierarchical clustering separated the differentially expressed proteins (DEPs) into two clusters. In particular, STK25 and PDCD10 were significantly upregulated in MIA PaCa-2 cells (Figure 3C). STRING-based protein–protein network analysis was performed to elucidate the functions of PROSER2, with a wider range of DEPs considered (|log2FC| > 0.3, *p* < 0.05). Additionally, GO annotation showed that these proteins were related to apoptosis pathway, cell migration in sprouting angiogenesis, type I interferon pathway, and mitotic cell cycle. Specifically, STK25 and PDCD10, which were enriched in apoptosis pathway, as well as interferon-related proteins, interferon-induced protein with tetratricopeptide repeats 3 (IFIT3) and interferon regulatory factor 9 (IRF9), were upregulated, whereas the mitotic cell cycle-related protein thymidylate synthetase (TYMS) was downregulated (Table S3, Figure S1). Among the DEPs, STK25 and PDCD10, which were the most highly expressed proteins in the PROSER2-overexpressing cells, were selected for further analysis.

### PROSER2 Binds with STK25 and PDCD10

**Figure 4:**
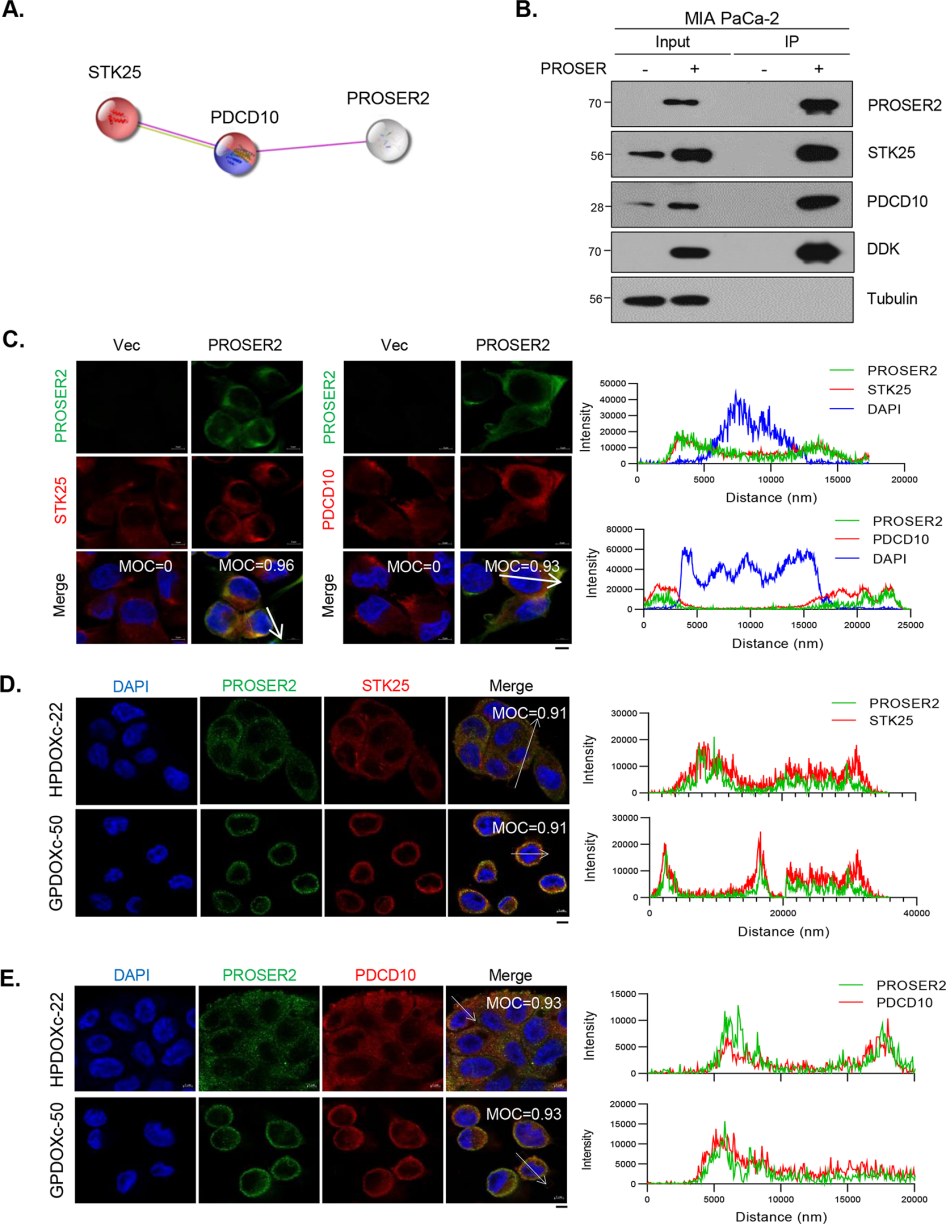
Identification of the Relationship of PROSER2 with STK25 and PDCD10. (A) Protein–protein interaction analysis of PROSER2 based on the STRING database (https://string-db.org) using the Cytoscape 3.9.1 program. Red indicates proteins involved in intrinsic apoptotic signaling pathways in response to hydrogen, and blue indicates proteins involved in the regulation of cell migration associated with angiogenesis. (B) Expression of STK25 and PDCD10 and demonstration of binding of PROSER2 with SKT25 and PDCD10 in PROSER2-overexpressing MIA PaCa-2 cells, as assessed using immunoprecipitation and Western blot analyses, respectively. (C) Confocal micrographs of PROSER2 (green), STK25 (red), PDCD10 (red), and DAPI (blue) in the PROSER2-overexpressing MIA PaCa-2 cell line (scale bar = 5 μm). Graphs indicate the colocalization of PROSER2 (green) with STK25 (red) and DAPI (blue) based on expression intensity. (D) Confocal micrographs of PROSER2 (green) and STK25 (red) in HPDOXc-22 and GPDOXc-50 (scale bar = 5 μm). Graphs indicate the colocalization of PROSER2 (green) with STK25 (red) based on expression intensity. (E) Confocal micrographs of PROSER2 (green) and PDCD10 (red) in HPDOXc-22 and GPDOXc-50. Graphs indicate the colocalization of PROSER2 (green) with PDCD10 (red) based on expression intensity (scale bar = 5 μm).

We examined whether PROSER2 can bind to STK25 and PDCD10. PDCD10 was indicated as a protein with potential for interaction with PROSER2,^19,20^ and identified as a PROSER2-related protein based on STRING network (Figure 4A). However, interaction between STK25 and PROSER2 has not been investigated, and there is no direct line of interaction with PROSER2 in the STRING network. Therefore, we checked whether PROSER2 binds with STK25 and PDCD10 by using immunoprecipitation. The expression of STK25 and PDCD10 was increased, and they were found to bind with PROSER2 in PROSER2-overexpressing MIA PaCa-2 cells (Figure 4B).

Immunofluorescence staining revealed the colocalization of PROSER2 with STK25 and PDCD10 in PROSER2-overexpressing MIA PaCa-2 cells, as evident from MOC and intensity graphs (Figure 4C). MOC ranges from 0 to 1, with 0 indicating no overlap between the intensity from both channels and 1 reflecting a complete colocalization between both channels. STK25 was expressed in both PROSER2-overexpressing and Vec-expressing cells but colocalized with PROSER2 only in PROSER2-overexpressing cells (MOC = 0.96). Additionally, the graphs show that the intensity of both PROSER2 and STK25 increased in same region. Similarly, PDCD10 was expressed in both cells, but colocalized with PROSER2 only in PROSER2-overexpressing cells (MOC = 0.92). The graphs show an increased intensity of both PDCD10 and PROSER2 in the cytosol (Figure 4C). Based on these results, to confirm the colocalization of STK25 and endogenous PROSER2, we performed IF staining in PDOXc (HPDOXc-22 and GPDOXc-50). In HPDOXc-22 and GPDOXc-50, PROSER2 was colocalized with STK25 (MOC = 0.91), as well as with PDCD10 (MOC = 0.93). The graphs show that the intensities of STK25 and PDCD10 increased in the same region as that of PROSER2 (Figure 4D, E). To validate the results in different cell lines, we confirmed colocalization in SNU-213 and SNU-410 cells. PROSER2 colocalized with STK25 (MOC = 0.91 or MOC = 0.89), as well as with PDCD10 (MOC = 0.9 or MOC = 0.91) in both cells. Additionally, the graphs show a higher intensity of PROSER2 in SNU-213 cells compared with that in SNU-410 cells, which is consistent with the results of Western blot analysis (Figure S2). These results suggest that PROSER2 regulates the STK25- and PDCD10-mediated functions by interaction with them and suppresses the invasion pathway.

### PROSER2 Reduces Invasion through STK25-p-AMPK Signaling

**Figure 5:**
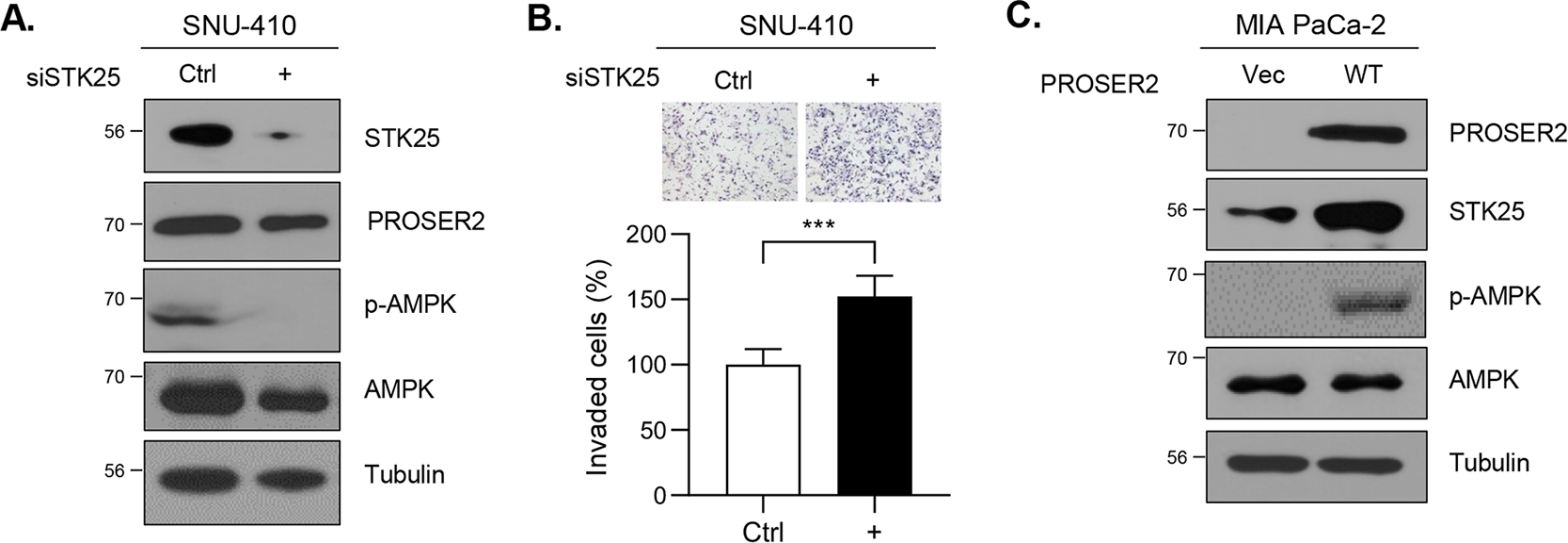
PROSER2 regulates STK25-AMPK signaling. (A) Expression of p-AMPK and PROSER2 in STK25-knockdown SNU-410 cells, as assessed by Western blot analysis. SNU-410 cells were transfected with siCtrl or siSTK25 and verified before use in the invasion assay. (B) Cell invasion of the STK25-knockdown SNU-410 cells. The graphs are present as mean ± SD (*n* = 5). ***p < 0.001. (C) Expression of p-AMPK in PROSER2-overexpressing MIA PaCa-2 cells.

To examine the plausible mechanism by which PROSER2 reduces tumor invasion while interacting with STK25, we investigated its impact on invasion by suppressing STK25 in PDAC cells and assessing p-AMPK levels through Western blot analysis. In SNU-410 cells where STK25 was knocked down, there was a decrease in p-AMPK levels along with a reduction in PROSER2 expression (Figure 5A). Consequently, cell invasion was significantly boosted by 50% (Figure 5B). Conversely, p-AMPK levels increased in PROSER2-overexpressing MIA PaCa-2 cells, leading to a decrease in cell invasion (Figure 5C, Figure 2B). These findings indicate that PROSER2 is involved in the regulation of the p-AMPK level through STK25, thereby reducing cell invasion. Our results highlight the role of PROSER2 in suppressing tumors and suggest its potential as a marker for PDAC progression.

## Discussion

In this study, we employed uPE1 in a novel strategy to identify markers that can regulate the progression of pancreatic cancer or predict its prognosis. Potential candidates were selected from large-scale proteomic cancer research data, and PROSER2 was chosen for functional analyses. Furthermore, it suggested that PROSER2 may inhibit the growth and metastasis of pancreatic cancer cells and regulate its mechanism through binding with STK25 and the PDCD10 complex.

Although PROSER2 has not been functionally annotated, there are reports of its relevance to cancer. Higher expression of PROSER2 is associated with decreased survival rates in pancreatic cancer patients, according to the Human Protein Atlas. Moreover, high PROSER2 expression has been reported to promote cancer cell metastasis in osteosarcoma.^21^ However, in this study, a contrasting pattern was observed with no or minimal expression of PROSER2 in advanced-stage pancreatic cancer (GPDOXc), and knockdown of PROSER2 in cells with high expression led to a significant increase in the growth and metastatic capability of cancer cells (Figure 2). This inconsistency may be attributed to differences in the samples analyzed. Considering that more than 90% of the samples analyzed in the Human Protein Atlas are surgical tissues from early-stage patients, advanced-stage patients with a pattern of decreased PROSER2 expression were not included. Although the sample size in this study was small, the results provide evidence that PROSER2 expression patterns may vary depending on the stage of the disease and also provide functional relevance through overexpression and knockdown at the cellular level. Furthermore, the analysis of binding partners of PROSER2, STK25, and PDCD10 revealed the potential mechanisms involved in the progression of pancreatic cancer, closely related to cell proliferation and migration.

STK25 is a germinal-center kinase (GCK) III subfamily and is highly expressed in liver and colorectal cancer, suggesting the possibility of STK25 as a prognostic marker for cancer. In contrast, the survival curve is long-rank, with high expression of STK25 in pancreatic cancer, indicating that STK25 may have different functions depending on its expression in various cancers. Accumulating evidence suggests that STK25 can act as either a tumor suppressor or a promoter. PDCD10, which is associated with STK25, can interact with other proteins, such as the GCKIII kinase family in the dimerization domain and Paxillin, Striatin, and CCM2 in the FAT-Homology domain. PDCD10 can form stratin-interacting phosphatase and kinase (STRIPAK) complexes, including STRN and PP2AC. In this complex, GCKIII can be inhibited by PP2AC, resulting in negative regulation of GCKIII.^22,23^ PDCD10 is overexpressed in several cancers, including pancreatic cancer, and is thought to induce tumor progression by enhancing cell proliferation, migration, and invasion.^23−26^ PDCD10 has a dimerization domain at its N-terminus and binds to STK25 in its Golgi localization, protecting and stabilizing STK25 from ubiquitin ligation, indicating that PDCD10 functions as an adapter protein for STK25.^22^ The binding of PROSER2 and PDCD10 in the STRING database was also confirmed experimentally.^19,20^ While the STRING database suggests interactions among PROSER2, PDCD10, and STK25, experimental validation for the binding of PROSER2 and PDCD10, excluding the interaction between STK25 and PDCD10, has not been reported. Furthermore, there is no existing research on the interaction between STK25 and PROSER2. We experimentally validated the potential binding of PROSER2 with PDCD10 and STK25 through immunoprecipitation analysis. Additionally, it provided evidence that PROSER2, PDCD10, and STK25 could form a complex and potentially interact.

Here, we have discovered the relevance of the PROSER2-STK25-AMPK pathway in regulating metastasis in pancreatic cancer. Phosphorylated AMPK at Thr172 has been reported not only to inhibit tumor cell proliferation and energy metabolism but also to exert dual regulatory effects on tumor cell invasion, with its association with STK25 also documented.^27−29^ Our experimental model suggests that p-AMPK plays a role in reducing metastasis. Deficiencies of PROSER2 and STK25 could induce cell invasion by reducing the p-AMPK. Moreover, in MIA PaCa-2 cells overexpressing PROSER2, an increase in p-AMPK expression led to a decrease in invasion (Figure 5C). These findings propose a new pathway where PROSER2, upon binding to the STK25-PDCD10 complex, increases downstream p-AMPK levels, thereby potentially escalating invasion and suggesting its possible utility as an indicator of PDAC progression.

The results of proteomic analysis showed that the expression of proteins related to tumor progression was reduced by PROSER2 (Figure 3). These results warrant the need for investigating the mechanism of action of PROSER2. For instance, proteins such as CCN1, the expression of which was decreased in PROSER2-expressing cells, may be involved in promoting cell migration and tumor vascularization. Additionally, the decrease in the expression of proteins, such as FAM20C, Dickkopf-1 (DKK1), prefoldin subunit 1 (PFDN1), TYMS, and S100-calcium-binding protein 6 (S100A6), in PROSER2-overexpressing cells can potentially promote the epithelial–mesenchymal transition in cancer. Among the downregulated proteins in PROSER2-overexpressing cells, TYMS and S100A6 are highly expressed in breast, gastric, and liver cancer, and in lymph node metastatic tissues, suggesting their potential as biomarkers for cancer metastasis.^30,31^ Furthermore, FAM20C is a marker for tumor progression in glioma and may promote metastasis in triple-negative breast cancer through the phosphorylation of target proteins.^32−34^ Therefore, PROSER2 may function as a tumor suppressor protein in PDAC.

This study highlights the need for further research in different cancer types to explore the potential value of PROSER2 as a novel regulator of tumor progression. It also underscores the significance of uPE1 in discovering new prognostic indicators and regulators in cancer. This serves as a promising example of the utility of uPE1, which could contribute significantly to future phases of the HPP.

## Data Availability

Proteome data is available at MassIVE (MSV000092953) and ProteomeXchange (PXD045646).

## Supporting Information Available

List of total proteins from PROSER2 overexpressed in MIA PaCa-2 cell line (PDF)
List of selected 97 proteins for STRING analysis (PDF)
List of upregulated and downregulated proteins (|log2FC| < 0.3 and *p*-value < 0.05) (PDF)
Evaluation of PROSER2 expression in PDAC cell lines and PDOXc, validation of PROSER2 in PROSER2-overexpressing MIA PaCa-2 cell line, and PROSER2 knockdown SNU-213 and SNU-410 cell lines; STRING analysis images and GO-term analysis; expression of STK25 and PDCD10 and demonstration of binding of PROSER2 with SKT25 and PDCD10 in PROSER2-overexpressing MIA PaCa-2 cells; the colocalization analysis between PROSER2 and STK25, PDCD10 in SNU-213 and SNU-410 cells; and expression of p-AMPK and PROSER2 in STK25-knockdown SNU-410 cells and expression of p-AMPK in PROSER2-overexpressing MIA PaCa-2 cells (PDF)

## Abbreviations

HPP: human proteome project
HUPO: human proteome organization
LC–MS/MS: liquid chromatography–mass spectrometry
PDAC: pancreatic ductal adenocarcinoma
PDCD10: programmed cell death 10
PROSER2: proline and serine-rich 2
STK25: serine/threonine kinase 25
uPE1: uncharacterized protein 1
