# Supplementary material for: New Function Annotation of PROSER2 in Pancreatic Ductal Adenocarcinoma
Source: J Proteome Res. 2024 Jan 31;23(3):905–15. doi: 10.1021/acs.jproteome.3c00632 (PMC10913870; doi:10.1021/acs.jproteome.3c00632)
Supplement: Supplementary file 1 — pr3c00632_si_001.pdf [file pr3c00632_si_001.pdf]

Table S1. List of total proteins from PROSER2 overexpressed in Miapaca-2 cell line.

Uniprot	Gene name	Protein abundances (They were log2-transformed and divided by reference value after normalization by column-median method)				Miapaca_C_avg	Miapaca_P_avg	P-value	log2FC(M_P/M_C)
		Miapaca_C_1	Miapaca_C_2	Miapaca_P_1	Miapaca_P_2				
Q14534	SQLE	-0.832	-0.295	-0.456	-0.451	-0.564	-0.454	0.752	0.110
Q9H8Y8	GORASP2	-3.949	-4.073	-3.988	-3.993	-4.011	-3.991	0.799	0.020
Q92623	TTC9	-1.974	-1.899	-1.872	-1.937	-1.936	-1.905	0.589	0.032
Q7Z2K8	GPRIN1	-0.275	-0.324	-0.408	-0.367	-0.300	-0.388	0.112	-0.088
Q9P0V3	SH3BP4	0.354	0.351	0.514	0.390	0.353	0.452	0.357	0.099
Q5T6S3	PHF19	-2.048	-1.928	-2.146	-1.954	-1.988	-2.050	0.648	-0.062
Q96PU4	UHRF2	-0.391	-0.302	-0.113	-0.074	-0.347	-0.094	0.073	0.253
Q8NG08	HELB	-4.206	-4.225	-4.197	-4.074	-4.216	-4.136	0.412	0.080
O14976	GAK	-1.466	-1.718	-1.583	-1.658	-1.592	-1.621	0.859	-0.029
Q9NRN7	AASDHPPT	1.491	1.331	1.355	1.313	1.411	1.334	0.508	-0.077
Q8IUL8	CILP2	-2.236	-2.293	-2.094	-2.137	-2.264	-2.115	0.060	0.149
P04350	TUBB4A	1.061	0.922	0.855	0.853	0.992	0.854	0.298	-0.138
P22033	MMUT	2.722	2.791	2.796	2.746	2.756	2.771	0.772	0.014
Q9HB07	MYG1	3.306	3.267	3.327	3.239	3.286	3.283	0.954	-0.003
Q9NVR2	INTS10	0.396	0.401	0.401	0.390	0.399	0.396	0.707	-0.003
Q9BYM8	RBCK1	-0.937	-0.933	-0.738	-0.761	-0.935	-0.749	0.034	0.185
Q9P2E5	CHPF2	-3.944	-3.988	-4.136	-4.057	-3.966	-4.097	0.134	-0.130
O43520	ATP8B1	-3.322	-3.243	-3.116	-2.976	-3.282	-3.046	0.130	0.237
Q9BV23	ABHD6	-1.937	-1.991	-1.961	-1.935	-1.964	-1.948	0.668	0.016
Q13233	MAP3K1	-3.227	-3.230	-3.208	-3.181	-3.228	-3.194	0.232	0.034
P61221	ABCE1	4.533	4.473	4.523	4.466	4.503	4.495	0.857	-0.008
Q9BRT9	GINS4	-0.466	-0.483	-0.874	-0.858	-0.475	-0.866	0.001	-0.391
Q7L3T8	PARS2	0.588	0.749	0.733	0.833	0.669	0.783	0.372	0.114
Q13445	TMED1	1.026	1.010	1.025	0.996	1.018	1.010	0.702	-0.008
Q9NWX5	ASB6	-3.858	-3.935	-4.127	-3.886	-3.897	-4.006	0.525	-0.110
P53701	HCCS	1.949	1.827	1.951	1.890	1.888	1.921	0.693	0.033
P51858	HDGF	0.849	0.631	0.932	0.736	0.740	0.834	0.587	0.094
P82914	MRPS15	2.234	2.203	2.268	2.149	2.219	2.208	0.890	-0.010
O96008	TOMM40	2.509	2.784	2.591	2.743	2.646	2.667	0.909	0.021
Q9NP50	SINHCAF	-1.944	-1.992	-1.957	-1.894	-1.968	-1.926	0.405	0.042
P36021	SLC16A2	-5.294	-4.652	-5.645	-5.320	-4.973	-5.482	0.331	-0.509

Q14679	TTLL4	-4.217	-4.142	-4.305	-4.234	-4.179	-4.269	0.222	-0.090
O95372	LYPLA2	3.609	3.574	3.537	3.448	3.592	3.492	0.237	-0.099
P26440	IVD	-1.589	-1.628	-1.401	-1.506	-1.608	-1.454	0.176	0.155
Q9NYF3	FAM53C	-2.364	-2.351	-2.526	-2.416	-2.357	-2.471	0.284	-0.113
Q8IY22	CMIP	-3.103	-3.052	-2.834	-2.934	-3.078	-2.884	0.112	0.194
O14832	PHYH	-2.308	-2.358	-1.936	-2.007	-2.333	-1.971	0.019	0.361
O14907	TAX1BP3	3.337	3.363	3.198	3.167	3.350	3.183	0.016	-0.167
P30838	ALDH3A1	1.379	2.302	1.586	1.637	1.841	1.611	0.707	-0.229
Q86VS8	HOOK3	2.509	2.427	2.430	2.389	2.468	2.409	0.364	-0.059
Q9HCL2	GPAM	-2.147	-2.123	-2.116	-1.954	-2.135	-2.035	0.428	0.100
Q9NTJ3	SMC4	0.996	1.153	0.909	0.873	1.074	0.891	0.244	-0.183
Q8WUM9	SLC20A1	0.161	0.117	-0.394	-0.319	0.139	-0.357	0.016	-0.496
Q9NX05	FAM120C	-0.734	-0.763	-0.698	-0.663	-0.748	-0.680	0.100	0.068
P50552	VASP	3.847	3.866	3.846	3.859	3.857	3.853	0.765	-0.004
Q9NYT0	PLEK2	-0.739	-0.327	-0.425	-0.478	-0.533	-0.452	0.760	0.082
Q9H307	PNN	3.745	3.719	3.810	3.748	3.732	3.779	0.349	0.047
O15270	SPTLC2	1.547	1.617	1.618	1.572	1.582	1.595	0.792	0.013
Q9BQ70	TCF25	0.918	0.881	0.860	0.904	0.899	0.882	0.613	-0.017
P55899	FCGRT	-1.015	-1.025	-0.910	-0.777	-1.020	-0.844	0.228	0.176
P50281	MMP14	-2.051	-1.839	-1.954	-1.774	-1.945	-1.864	0.620	0.081
P35367	HRH1	-2.008	-2.031	-1.864	-1.997	-2.020	-1.930	0.402	0.089
P61158	ACTR3	4.927	4.952	4.991	4.976	4.940	4.983	0.125	0.044
O60783	MRPS14	-0.169	-0.246	-0.290	-0.248	-0.208	-0.269	0.331	-0.061
Q96G23	CERS2	0.896	0.831	0.837	0.819	0.864	0.828	0.466	-0.036
P49006	MARCKSL1	1.443	1.325	1.235	1.210	1.384	1.222	0.209	-0.162
P35227	PCGF2	0.195	0.135	0.115	0.175	0.165	0.145	0.675	-0.020
O43488	AKR7A2	3.112	2.948	3.118	2.982	3.030	3.050	0.869	0.020
O14879	IFIT3	0.639	0.552	1.192	1.091	0.596	1.141	0.016	0.545
P52566	ARHGDIB	-0.295	-0.273	0.513	-0.111	-0.284	0.201	0.364	0.485
O00399	DCTN6	-0.969	-0.988	-0.820	-1.003	-0.978	-0.912	0.599	0.067
O43609	SPRY1	-0.662	-0.789	-0.901	-0.926	-0.725	-0.914	0.197	-0.188
Q9UGP5	POLL	-0.063	0.033	0.001	0.111	-0.015	0.056	0.437	0.071
Q12774	ARHGEF5	-2.506	-2.205	-2.376	-2.231	-2.355	-2.304	0.796	0.052
P42336	PIK3CA	-0.556	-0.609	-0.446	-0.434	-0.582	-0.440	0.102	0.143
Q3MIX3	ADCK5	-2.242	-2.382	-2.267	-2.368	-2.312	-2.318	0.952	-0.006

Q9H9D4	ZNF408	-2.436	-2.276	-2.425	-2.266	-2.356	-2.345	0.932	0.011
P32119	PRDX2	4.765	4.724	4.654	4.756	4.744	4.705	0.578	-0.039
Q9H8W3	FAM204A	-1.152	-1.106	-1.357	-1.094	-1.129	-1.226	0.595	-0.097
Q9P2X3	IMPACT	-1.431	-1.272	-1.476	-1.280	-1.352	-1.378	0.856	-0.026
P23246	SFPQ	1.932	1.931	1.867	1.917	1.932	1.892	0.355	-0.040
P23458	JAK1	0.420	0.469	0.706	0.759	0.445	0.733	0.015	0.288
Q15149	PLEC	0.880	0.992	0.947	0.866	0.936	0.906	0.716	-0.029
Q8N0Z6	TTC5	-0.445	-0.248	-0.515	-0.453	-0.346	-0.484	0.380	-0.138
P51828	ADCY7	-4.469	-4.136	-4.907	-4.478	-4.302	-4.693	0.294	-0.390
P05386	RPLP1	4.166	4.205	4.002	4.135	4.186	4.068	0.311	-0.117
P51003	PAPOLA	2.130	2.202	1.961	2.012	2.166	1.986	0.065	-0.180
P55081	MFAP1	1.991	1.991	2.034	2.081	1.991	2.057	0.219	0.066
P61966	AP1S1	-2.436	-2.554	-2.554	-2.545	-2.495	-2.549	0.524	-0.055
P63220	RPS21	3.390	3.183	3.442	3.236	3.286	3.339	0.753	0.053
Q9BVS4	RIOK2	-1.444	-1.413	-1.337	-1.287	-1.429	-1.312	0.078	0.117
Q96DV4	MRPL38	0.777	0.738	0.850	0.840	0.758	0.845	0.121	0.087
Q9H1U4	MEGF9	-3.568	-3.601	-3.614	-3.728	-3.585	-3.671	0.355	-0.087
Q96EY7	PTCD3	3.078	3.047	2.982	2.902	3.063	2.942	0.170	-0.120
Q9Y315	DERA	3.138	3.091	3.003	3.048	3.114	3.026	0.114	-0.089
Q6Y288	B3GLCT	-1.259	-1.284	-1.363	-1.336	-1.271	-1.349	0.055	-0.078
P62306	SNRPF	2.946	2.879	2.859	2.791	2.913	2.825	0.208	-0.088
P04083	ANXA1	8.164	8.117	8.574	8.513	8.141	8.544	0.011	0.403
O75907	DGAT1	-1.329	-1.547	-1.482	-1.624	-1.438	-1.553	0.482	-0.115
Q6P1M9	ARMCX5	-0.970	-1.074	-1.044	-0.990	-1.022	-1.017	0.942	0.005
P50213	IDH3A	2.509	2.367	2.723	2.538	2.438	2.630	0.250	0.192
O15060	ZBTB39	-3.811	-3.862	-3.875	-3.867	-3.836	-3.871	0.397	-0.035
Q8N9Z2	CCDC71L	0.143	0.044	0.662	0.608	0.093	0.635	0.023	0.542
Q7Z7A3	CTU1	-0.864	-0.894	-0.877	-0.847	-0.879	-0.862	0.516	0.017
Q9Y2Y1	POLR3K	-2.504	-2.553	-2.571	-2.603	-2.528	-2.587	0.202	-0.059
Q86VF2	IGFN1	0.950	0.809	1.110	1.053	0.879	1.081	0.178	0.202
O00194	RAB27B	3.265	3.013	3.285	3.173	3.139	3.229	0.607	0.090
Q9Y5B6	PAXBP1	-0.353	-0.394	-0.524	-0.385	-0.373	-0.454	0.443	-0.081
Q3MIT2	PUS10	-1.418	-1.312	-1.437	-1.301	-1.365	-1.369	0.969	-0.004
Q92979	EMG1	2.686	2.608	2.582	2.616	2.647	2.599	0.416	-0.049
Q3B7J2	GFOD2	-1.466	-1.448	-1.419	-1.455	-1.457	-1.437	0.457	0.020

P16144	ITGB4	-2.106	-1.878	-2.013	-1.967	-1.992	-1.990	0.988	0.002
P67870	CSNK2B	4.040	3.908	3.999	3.908	3.974	3.954	0.828	-0.020
Q5VT06	CEP350	0.126	0.078	0.018	0.101	0.102	0.060	0.490	-0.042
P0CG12	DERPC	-0.242	-0.337	-0.069	-0.103	-0.289	-0.086	0.117	0.203
P78344	EIF4G2	-1.235	-1.537	-1.549	-1.416	-1.386	-1.483	0.640	-0.097
Q9UBU9	NXF1	1.960	1.975	1.883	1.936	1.967	1.909	0.250	-0.058
Q02818	NUCB1	1.953	1.911	2.154	2.113	1.932	2.133	0.021	0.201
Q9H9J2	MRPL44	3.752	3.740	3.667	3.657	3.746	3.662	0.009	-0.084
Q15645	TRIP13	3.592	3.490	3.445	3.403	3.541	3.424	0.227	-0.117
P38432	COIL	1.852	1.897	1.905	1.957	1.874	1.931	0.242	0.057
Q8N4A0	GALNT4	-1.266	-1.187	-1.262	-1.179	-1.227	-1.220	0.924	0.006
Q9Y2K6	USP20	-2.200	-2.224	-2.191	-2.198	-2.212	-2.195	0.360	0.018
O75071	EFCAB14	-1.800	-1.855	-1.723	-1.798	-1.828	-1.761	0.297	0.067
O00625	PIR	1.699	1.769	1.459	1.560	1.734	1.510	0.080	-0.224
Q9H2H8	PPIL3	-3.297	-3.208	-3.154	-3.041	-3.253	-3.097	0.169	0.155
P23368	ME2	1.861	1.787	1.828	1.848	1.824	1.838	0.766	0.014
Q9Y5X3	SNX5	3.201	3.217	3.138	3.188	3.209	3.163	0.297	-0.046
O43240	KLK10	-3.750	-3.619	-3.979	-3.775	-3.684	-3.877	0.274	-0.192
O00566	MPHOSPH10	3.675	3.727	3.754	3.857	3.701	3.805	0.254	0.104
Q9BS26	ERP44	3.709	3.752	3.794	3.756	3.731	3.775	0.267	0.044
O75084	FZD7	-3.323	-3.324	-3.019	-3.137	-3.323	-3.078	0.150	0.246
Q9UKV8	AGO2	-2.936	-2.994	-2.941	-2.924	-2.965	-2.932	0.457	0.032
Q9H0A0	NAT10	0.855	0.833	0.804	0.834	0.844	0.819	0.314	-0.025
Q15014	MORF4L2	1.545	1.467	1.624	1.509	1.506	1.567	0.483	0.061
Q13144	EIF2B5	2.649	2.549	2.523	2.485	2.599	2.504	0.283	-0.095
Q9P1Z0	ZBTB4	-4.018	-3.933	-3.809	-3.766	-3.976	-3.787	0.094	0.188
Q6PI77	BHLHB9	-4.254	-4.223	-4.446	-4.381	-4.239	-4.413	0.074	-0.174
Q8NBN7	RDH13	0.409	0.237	0.495	0.427	0.323	0.461	0.331	0.138
O75716	STK16	-2.579	-2.473	-2.500	-2.419	-2.526	-2.460	0.433	0.066
Q9Y2M0	FAN1	-3.396	-3.325	-3.274	-3.257	-3.361	-3.266	0.212	0.095
Q13432	UNC119	-2.851	-2.763	-2.805	-2.789	-2.807	-2.797	0.854	0.010
P49368	CCT3	-0.723	-0.987	-0.796	-0.926	-0.855	-0.861	0.973	-0.006
Q8N5F7	NKAP	-1.065	-1.093	-1.009	-0.951	-1.079	-0.980	0.132	0.099
Q9BQ39	DDX50	3.559	3.534	3.446	3.478	3.547	3.462	0.058	-0.085
Q9Y619	SLC25A15	2.270	2.252	2.302	2.338	2.261	2.320	0.145	0.058

P09001	MRPL3	3.815	3.844	3.784	3.811	3.829	3.797	0.251	-0.032
P07737	PFN1	7.333	7.302	7.259	7.226	7.317	7.242	0.081	-0.075
Q8TBC3	SHKBP1	-2.551	-2.461	-2.451	-2.349	-2.506	-2.400	0.258	0.106
Q9H444	CHMP4B	1.487	1.578	1.651	1.620	1.532	1.635	0.240	0.103
Q07954	LRP1	-0.358	-0.410	-0.406	-0.437	-0.384	-0.421	0.370	-0.037
Q9BZE1	MRPL37	3.491	3.421	3.424	3.395	3.456	3.410	0.391	-0.046
Q8IVD9	NUDCD3	3.198	3.121	3.114	3.107	3.160	3.110	0.420	-0.049
P47989	XDH	-3.988	-4.177	-4.362	-4.388	-4.082	-4.375	0.193	-0.293
O95299	NDUFA10	0.380	0.301	0.403	0.387	0.340	0.395	0.391	0.055
Q9H267	VPS33B	-0.895	-0.810	-0.793	-0.940	-0.853	-0.866	0.891	-0.014
Q8N5M9	JAGN1	0.600	0.536	0.511	0.581	0.568	0.546	0.686	-0.022
O95835	LATS1	-1.967	-1.872	-2.021	-1.893	-1.919	-1.957	0.690	-0.037
Q9C0C4	SEMA4C	-1.903	-2.078	-1.719	-1.839	-1.991	-1.779	0.201	0.212
P41252	IARS1	6.415	6.375	6.316	6.318	6.395	6.317	0.159	-0.078
Q9Y375	NDUFAF1	0.878	0.913	0.849	0.851	0.896	0.850	0.237	-0.045
Q9ULX3	NOB1	1.505	1.410	1.490	1.407	1.457	1.449	0.905	-0.009
P60228	EIF3E	4.338	4.391	4.267	4.322	4.364	4.295	0.210	-0.069
P07910	HNRNPC	-5.078	-4.307	-4.750	-4.903	-4.693	-4.827	0.787	-0.134
P31751	AKT2	-1.267	-1.289	-1.296	-1.394	-1.278	-1.345	0.397	-0.066
Q13889	GTF2H3	-2.215	-2.336	-2.194	-2.249	-2.276	-2.222	0.533	0.054
P22492	H1	-3.102	-3.244	-3.195	-3.114	-3.173	-3.155	0.851	0.018
P61970	NUTF2	5.546	5.428	5.556	5.508	5.487	5.532	0.586	0.045
Q9UHD1	CHORDC1	-0.772	-0.842	-0.892	-0.925	-0.807	-0.909	0.167	-0.101
Q08752	PPID	3.595	3.524	3.541	3.484	3.559	3.512	0.412	-0.047
P07919	UQCRH	-1.126	-1.169	-0.966	-0.934	-1.147	-0.950	0.022	0.197
O43237	DYNC1LI2	-5.077	-4.967	-5.192	-4.951	-5.022	-5.071	0.759	-0.049
Q8WY91	THAP4	-2.045	-2.135	-2.045	-2.042	-2.090	-2.044	0.492	0.046
P30566	ADSL	-0.434	-0.506	-0.530	-0.422	-0.470	-0.476	0.936	-0.006
Q15172	PPP2R5A	-1.256	-1.363	-1.367	-1.383	-1.310	-1.375	0.432	-0.065
Q8ND24	RNF214	-1.208	-1.245	-1.364	-1.362	-1.226	-1.363	0.084	-0.137
Q02750	MAP2K1	0.681	1.094	0.791	0.868	0.888	0.829	0.826	-0.058
P51149	RAB7A	5.648	5.614	5.590	5.597	5.631	5.593	0.261	-0.038
Q9BRQ6	CHCHD6	0.602	0.592	0.799	0.820	0.597	0.810	0.011	0.212
Q7L8W6	DPH6	-2.647	-2.603	-2.635	-2.430	-2.625	-2.533	0.530	0.092
Q8NFG4	FLCN	-1.316	-1.416	-1.397	-1.414	-1.366	-1.405	0.577	-0.039

Q9NPL8	TIMMDC1	1.251	1.182	1.429	1.453	1.217	1.441	0.070	0.224
A8CG34	POM121C	-1.590	-1.464	-1.707	-1.543	-1.527	-1.625	0.448	-0.098
O43900	PRICKLE3	-5.140	-5.282	-5.112	-4.752	-5.211	-4.932	0.343	0.280
Q99567	NUP88	2.784	2.704	2.871	2.805	2.744	2.838	0.215	0.094
O15111	CHUK	1.877	1.846	1.818	1.827	1.861	1.823	0.226	-0.039
Q13115	DUSP4	-4.195	-3.538	-3.728	-3.628	-3.867	-3.678	0.668	0.188
P49642	PRIM1	1.737	1.746	1.465	1.484	1.741	1.474	0.007	-0.267
O94953	KDM4B	-1.743	-1.667	-1.607	-1.495	-1.705	-1.551	0.168	0.154
Q9H0H5	RACGAP1	1.630	1.747	1.634	1.580	1.689	1.607	0.375	-0.082
Q9NP81	SARS2	-1.665	-1.694	-1.569	-1.601	-1.679	-1.585	0.048	0.094
P02144	MB	-3.478	-3.529	-3.554	-3.636	-3.503	-3.595	0.221	-0.092
P61088	UBE2N	2.204	2.272	2.006	1.996	2.238	2.001	0.086	-0.237
O43805	SSNA1	-2.055	-1.821	-1.931	-1.927	-1.938	-1.929	0.951	0.009
Q8NI36	WDR36	4.041	4.005	4.092	4.081	4.023	4.087	0.152	0.064
P35268	RPL22	4.907	4.803	4.838	4.785	4.855	4.811	0.557	-0.043
Q99797	MIPEP	2.856	2.887	2.818	2.836	2.872	2.827	0.162	-0.045
Q16644	MAPKAPK3	-0.246	-0.215	-0.136	-0.169	-0.231	-0.152	0.076	0.078
Q6EMB2	TTLL5	-3.657	-3.695	-3.865	-3.620	-3.676	-3.743	0.682	-0.067
P57105	SYNJ2BP	1.144	1.218	1.221	1.212	1.181	1.216	0.517	0.035
Q6NSJ2	PHLDB3	-0.137	-0.199	-0.179	-0.275	-0.168	-0.227	0.424	-0.059
Q9BV81	EMC6	-1.139	-1.079	-0.989	-1.006	-1.109	-0.997	0.144	0.112
Q9BVG9	PTDSS2	-2.780	-2.687	-2.958	-2.782	-2.734	-2.870	0.337	-0.137
O15212	PFDN6	3.737	3.676	3.650	3.576	3.706	3.613	0.197	-0.093
Q9H5X1	CIAO2A	-0.906	-1.148	-0.994	-0.973	-1.027	-0.983	0.779	0.044
Q9Y490	TLN1	6.254	6.157	6.289	6.181	6.206	6.235	0.721	0.030
Q8WYA6	CTNNBL1	-3.507	-3.853	-3.605	-4.086	-3.680	-3.845	0.637	-0.166
Q9NVF7	FBXO28	-0.713	-0.723	-0.746	-0.660	-0.718	-0.703	0.794	0.014
Q15223	NECTIN1	-3.347	-2.967	-3.452	-3.074	-3.157	-3.263	0.731	-0.106
Q9UPU5	USP24	3.344	3.235	3.219	3.185	3.289	3.202	0.340	-0.087
O60869	EDF1	-0.047	-0.034	0.224	0.004	-0.040	0.114	0.392	0.155
Q8IWB7	WDFY1	1.561	1.540	1.522	1.475	1.550	1.498	0.234	-0.052
P63279	UBE2I	4.033	4.034	4.127	4.130	4.034	4.128	0.007	0.095
P03973	SLPI	-4.409	-4.867	-4.174	-4.497	-4.638	-4.336	0.404	0.302
Q7L014	DDX46	5.234	5.230	5.189	5.174	5.232	5.182	0.073	-0.050
Q86V24	ADIPOR2	-3.391	-3.325	-3.488	-3.460	-3.358	-3.474	0.134	-0.116

Q96GI7	FAM89A	-4.591	-4.613	-4.466	-4.517	-4.602	-4.492	0.104	0.110
Q15007	WTAP	0.983	0.874	1.017	0.875	0.928	0.946	0.861	0.018
P62851	RPS25	6.471	6.391	6.419	6.307	6.431	6.363	0.437	-0.068
Q52LR7	EPC2	-1.993	-2.017	-2.023	-1.965	-2.005	-1.994	0.770	0.011
Q9H2M9	RAB3GAP2	3.536	3.484	3.515	3.481	3.510	3.498	0.743	-0.012
Q53H82	LACTB2	2.696	2.731	2.693	2.648	2.714	2.671	0.278	-0.043
Q9UKT8	FBXW2	-3.392	-3.288	-3.523	-3.453	-3.340	-3.488	0.159	-0.148
O15126	SCAMP1	-0.925	-0.978	-0.949	-1.008	-0.952	-0.979	0.567	-0.027
Q9Y2H2	INPP5F	-0.906	-0.830	-0.975	-0.887	-0.868	-0.931	0.397	-0.063
Q13416	ORC2	2.239	2.269	2.188	2.180	2.254	2.184	0.114	-0.070
Q04771	ACVR1	-3.119	-2.667	-3.140	-2.865	-2.893	-3.003	0.726	-0.110
A8MTT3	CEBPZOS	-0.864	-0.980	-0.696	-0.776	-0.922	-0.736	0.134	0.186
Q9HCM1	RESF1	-3.100	-3.190	-2.916	-3.097	-3.145	-3.007	0.343	0.139
Q6PL18	ATAD2	1.235	1.397	1.198	1.217	1.316	1.208	0.406	-0.109
Q9NS00	C1GALT1	-1.628	-1.723	-1.551	-1.594	-1.676	-1.572	0.238	0.103
Q13049	TRIM32	1.238	1.257	1.292	1.336	1.248	1.314	0.159	0.067
Q9NW64	RBM22	0.263	0.199	0.207	0.143	0.231	0.175	0.337	-0.056
Q5SXM8	DNLZ	-0.122	-0.003	-0.149	-0.183	-0.062	-0.166	0.313	-0.104
Q9NR33	POLE4	-0.625	-0.512	-0.816	-0.733	-0.569	-0.774	0.109	-0.206
Q4G0I0	CCSMST1	-4.495	-4.372	-4.416	-4.388	-4.434	-4.402	0.698	0.032
Q86UE4	MTDH	2.867	2.905	2.960	2.852	2.886	2.906	0.778	0.020
Q6IQ22	RAB12	0.760	0.700	0.569	0.510	0.730	0.540	0.046	-0.190
Q9BUI4	POLR3C	1.104	0.896	1.104	0.904	1.000	1.004	0.981	0.004
Q9NNW5	WDR6	-0.793	-0.857	-0.895	-0.840	-0.825	-0.868	0.420	-0.043
O75808	CAPN15	-1.826	-1.924	-1.869	-1.887	-1.875	-1.878	0.968	-0.002
Q9H3P2	NELFA	2.053	2.011	2.094	2.088	2.032	2.091	0.213	0.059
P26045	PTPN3	-4.512	-4.305	-4.398	-4.437	-4.409	-4.417	0.949	-0.008
Q9C086	INO80B	-2.303	-2.311	-2.357	-2.223	-2.307	-2.290	0.844	0.017
Q07157	TJP1	-2.824	-2.680	-2.646	-2.679	-2.752	-2.663	0.425	0.090
Q08188	TGM3	-3.606	-3.866	-3.659	-4.015	-3.736	-3.837	0.696	-0.101
A6NHX0	CASTOR2	-1.317	-1.489	-1.539	-1.469	-1.403	-1.504	0.435	-0.101
O75385	ULK1	-2.274	-2.253	-2.315	-2.208	-2.263	-2.261	0.977	0.002
Q86Y79	PTRH1	-0.447	-0.415	-0.415	-0.298	-0.431	-0.356	0.411	0.075
Q9GZV4	EIF5A2	-1.752	-1.884	-1.802	-1.831	-1.818	-1.817	0.992	0.001
Q14232	EIF2B1	2.168	2.198	2.043	2.113	2.183	2.078	0.165	-0.105

Q96M94	KLHL15	-4.643	-4.649	-4.638	-4.438	-4.646	-4.538	0.477	0.108
P13010	XRCC5	7.039	6.987	7.012	6.954	7.013	6.983	0.524	-0.030
O15182	CETN3	-1.105	-1.158	-1.092	-1.128	-1.132	-1.110	0.575	0.022
Q9GZM5	YIPF3	0.446	0.415	0.554	0.415	0.430	0.485	0.576	0.054
Q9NX57	RAB20	-4.370	-4.139	-4.142	-4.078	-4.254	-4.110	0.420	0.144
Q92625	ANKS1A	0.339	0.337	0.323	0.339	0.338	0.331	0.552	-0.007
Q9UMF0	ICAM5	-3.467	-3.493	-3.444	-3.202	-3.480	-3.323	0.417	0.157
O15127	SCAMP2	0.518	0.689	0.551	0.530	0.603	0.541	0.597	-0.063
P08648	ITGA5	-0.839	-0.903	-0.885	-0.871	-0.871	-0.878	0.867	-0.007
Q9Y2U9	KLHDC2	-2.965	-3.398	-3.036	-3.142	-3.182	-3.089	0.743	0.093
O95714	HERC2	2.006	1.972	1.892	1.930	1.989	1.911	0.096	-0.078
Q9HCE1	MOV10	-1.472	-1.485	-1.583	-1.586	-1.478	-1.585	0.028	-0.107
Q8WUA8	TSKU	-2.541	-2.528	-2.618	-2.482	-2.535	-2.550	0.862	-0.015
Q5QJ74	TBCEL	-0.557	-0.738	-0.554	-0.647	-0.648	-0.601	0.701	0.047
Q92934	BAD	0.127	0.153	0.418	0.248	0.140	0.333	0.257	0.193
Q9Y6D0	SELENOK	-1.662	-1.572	-1.471	-1.590	-1.617	-1.530	0.371	0.087
O75781	PALM	0.051	0.000	-0.057	-0.030	0.026	-0.043	0.176	-0.069
P28072	PSMB6	2.825	2.742	2.731	2.732	2.783	2.732	0.432	-0.051
Q9HC44	GPBP1L1	-3.656	-3.711	-3.768	-3.495	-3.684	-3.632	0.768	0.052
Q9H0L4	CSTF2T	1.015	0.968	1.009	1.003	0.991	1.006	0.646	0.015
O15145	ARPC3	4.399	4.456	4.399	4.471	4.427	4.435	0.886	0.007
Q12980	NPRL3	-4.112	-3.906	-4.091	-3.769	-4.009	-3.930	0.727	0.079
P37108	SRP14	4.013	3.976	3.996	4.012	3.995	4.004	0.705	0.009
P10589	NR2F1	-2.513	-2.670	-2.751	-2.683	-2.591	-2.717	0.331	-0.126
P38117	ETFB	1.219	1.138	1.202	1.166	1.179	1.184	0.916	0.006
O14786	NRP1	-6.773	-6.286	-6.052	-6.559	-6.529	-6.306	0.590	0.223
Q16891	IMMT	-3.310	-3.467	-3.243	-3.248	-3.388	-3.245	0.319	0.143
P30086	PEBP1	6.145	6.091	6.131	6.180	6.118	6.156	0.411	0.038
Q14126	DSG2	1.066	1.451	1.428	1.336	1.259	1.382	0.635	0.123
P50991	CCT4	3.013	2.739	3.061	2.779	2.876	2.920	0.844	0.044
O00257	CBX4	0.158	0.101	0.324	0.262	0.129	0.293	0.060	0.164
Q8IY21	DDX60	-1.879	-1.867	-1.305	-1.490	-1.873	-1.397	0.121	0.475
Q9BW04	SARG	-2.806	-2.457	-2.738	-2.606	-2.632	-2.672	0.858	-0.040
Q9NU22	MDN1	4.514	4.473	4.501	4.539	4.494	4.520	0.446	0.026
O75351	VPS4B	3.029	2.970	3.060	3.058	3.000	3.059	0.290	0.060

Q9H074	PAIP1	-0.108	-0.116	-0.165	-0.035	-0.112	-0.100	0.889	0.011
Q9Y365	STARD10	-0.453	-0.233	-0.358	-0.308	-0.343	-0.333	0.942	0.010
Q7Z6J9	TSEN54	-1.140	-1.170	-1.254	-1.264	-1.155	-1.259	0.065	-0.104
P09132	SRP19	0.951	0.959	0.806	0.806	0.955	0.806	0.017	-0.149
O75151	PHF2	0.466	0.419	0.529	0.459	0.442	0.494	0.362	0.051
Q92636	NSMAF	-3.338	-3.291	-3.356	-3.332	-3.314	-3.344	0.406	-0.030
O43598	DNPH1	0.247	0.083	0.098	0.175	0.165	0.137	0.792	-0.028
Q9BQ90	KLHDC3	-2.106	-2.048	-2.002	-1.954	-2.077	-1.978	0.122	0.099
Q14197	MRPL58	2.820	2.791	2.951	2.857	2.806	2.904	0.262	0.099
Q96GA7	SDSL	1.740	1.679	1.801	1.815	1.710	1.808	0.176	0.098
O00505	KPNA3	2.513	2.434	2.335	2.386	2.474	2.360	0.158	-0.114
Q9BYT8	NLN	4.271	4.240	4.271	4.224	4.255	4.247	0.809	-0.008
Q5T6V5	C9orf64	-0.331	0.564	0.229	0.039	0.116	0.134	0.975	0.018
Q14517	FAT1	-0.370	-0.285	-0.511	-0.457	-0.328	-0.484	0.113	-0.157
Q9UBZ4	APEX2	-3.762	-3.733	-3.269	-3.488	-3.748	-3.379	0.178	0.369
P36404	ARL2	0.782	0.703	0.651	0.652	0.742	0.652	0.262	-0.090
P42226	STAT6	-0.863	-0.846	-0.767	-0.828	-0.854	-0.797	0.301	0.057
Q96B45	BORCS7	-2.063	-2.153	-2.132	-2.254	-2.108	-2.193	0.386	-0.085
O43447	PPIH	0.534	0.441	0.304	0.333	0.488	0.318	0.146	-0.169
Q9P1Y5	CAMSAP3	-3.561	-3.474	-3.528	-3.334	-3.518	-3.431	0.533	0.087
O75712	GJB3	-4.103	-3.441	-4.134	-3.673	-3.772	-3.904	0.778	-0.132
P40939	HADHA	6.418	6.343	6.503	6.487	6.380	6.495	0.189	0.114
Q53GQ0	HSD17B12	3.456	3.395	3.458	3.427	3.426	3.442	0.685	0.017
Q8TBE9	NANP	-1.792	-1.793	-1.701	-1.822	-1.793	-1.761	0.695	0.031
Q12891	HYAL2	-2.823	-2.813	-2.849	-2.835	-2.818	-2.842	0.128	-0.024
Q92692	NECTIN2	-1.060	-1.048	-1.002	-1.060	-1.054	-1.031	0.574	0.023
Q16254	E2F4	-0.536	-0.576	-0.623	-0.522	-0.556	-0.573	0.797	-0.017
Q96NB2	SFXN2	-0.054	-0.095	-0.184	-0.146	-0.074	-0.165	0.084	-0.091
Q9Y496	KIF3A	1.245	1.243	1.217	1.216	1.244	1.217	0.006	-0.028
Q8IYT2	CMTR2	0.971	0.936	1.052	1.044	0.953	1.048	0.099	0.095
Q9BUZ4	TRAF4	-3.353	-3.296	-3.119	-3.195	-3.325	-3.157	0.080	0.168
Q9UKK3	PARP4	2.876	2.767	3.034	2.977	2.822	3.006	0.132	0.184
Q15904	ATP6AP1	0.785	0.863	0.703	0.769	0.824	0.736	0.229	-0.089
Q9NZM5	NOP53	2.074	1.967	2.199	2.002	2.021	2.100	0.567	0.080
Q9BVQ7	SPATA5L1	-2.019	-1.954	-2.117	-1.965	-1.986	-2.041	0.601	-0.055

Q9Y2X3	NOP58	4.484	4.400	4.523	4.498	4.442	4.510	0.337	0.068
Q9H5N1	RABEP2	-1.260	-1.279	-1.364	-1.332	-1.269	-1.348	0.075	-0.078
Q9BVT8	TMUB1	-1.138	-1.272	-1.134	-1.204	-1.205	-1.169	0.692	0.036
P50402	EMD	3.569	3.522	3.647	3.460	3.546	3.554	0.947	0.008
P33552	CKS2	-0.373	-0.312	-0.532	-0.479	-0.342	-0.506	0.057	-0.163
Q9Y244	POMP	-1.097	-0.899	-1.101	-0.959	-0.998	-1.030	0.819	-0.032
Q9BRR8	GPATCH1	0.493	0.614	0.460	0.659	0.553	0.560	0.963	0.006
Q8NFQ8	TOR1AIP2	2.329	2.147	2.192	2.038	2.238	2.115	0.414	-0.123
Q8IXT5	RBM12B	2.691	2.664	2.807	2.763	2.677	2.785	0.071	0.108
Q6PI78	TMEM65	-2.987	-2.994	-3.084	-2.903	-2.990	-2.994	0.978	-0.003
Q8TBN0	RAB3IL1	-3.489	-3.490	-3.679	-3.395	-3.489	-3.537	0.793	-0.048
O75317	USP12	-3.155	-3.100	-3.343	-3.167	-3.127	-3.255	0.370	-0.128
P25098	GRK2	1.772	1.864	1.861	1.811	1.818	1.836	0.765	0.019
O15260	SURF4	-1.410	-1.351	-1.528	-1.467	-1.381	-1.497	0.112	-0.117
Q9BX46	RBM24	-1.270	-1.320	-1.299	-1.337	-1.295	-1.318	0.550	-0.023
P28370	SMARCA1	-1.495	-1.772	-1.552	-1.553	-1.634	-1.553	0.662	0.081
Q03252	LMNB2	5.429	5.355	5.428	5.413	5.392	5.420	0.577	0.029
P50453	SERPINB9	5.927	5.793	6.276	6.225	5.860	6.250	0.077	0.390
P31943	HNRNPH1	4.344	4.395	4.270	4.260	4.369	4.265	0.139	-0.104
Q9BY32	ITPA	-4.166	-4.127	-4.241	-4.241	-4.147	-4.241	0.130	-0.094
P57076	CFAP298	1.630	1.581	1.525	1.597	1.605	1.561	0.426	-0.044
O75439	PMPCB	3.676	3.645	3.695	3.700	3.660	3.697	0.238	0.037
Q92620	DHX38	1.939	1.923	1.877	1.860	1.931	1.869	0.036	-0.062
Q6NUQ4	TMEM214	-3.068	-3.051	-3.243	-3.311	-3.059	-3.277	0.083	-0.218
P46459	NSF	0.948	1.051	0.798	0.672	0.999	0.735	0.087	-0.264
Q9NVU7	SDAD1	0.075	0.064	0.135	0.137	0.069	0.136	0.045	0.067
Q9H6K1	ILRUN	-1.611	-1.820	-1.579	-1.650	-1.715	-1.615	0.503	0.101
Q9H4E7	DEF6	2.054	1.949	1.910	1.906	2.001	1.908	0.325	-0.093
Q9ULJ3	ZBTB21	-2.353	-2.383	-2.276	-2.194	-2.368	-2.235	0.160	0.133
Q9UL54	TAOK2	-2.508	-2.437	-2.480	-2.484	-2.473	-2.482	0.838	-0.009
Q9Y664	KPTN	-2.966	-2.973	-2.547	-2.509	-2.970	-2.528	0.023	0.442
Q96C90	PPP1R14B	-2.204	-2.006	-2.109	-1.948	-2.105	-2.028	0.612	0.077
Q15751	HERC1	0.598	0.570	0.528	0.533	0.584	0.531	0.156	-0.053
Q6P5Z2	PKN3	-4.337	-4.341	-4.301	-4.320	-4.339	-4.310	0.185	0.029
O60825	PFKFB2	-1.972	-1.835	-1.683	-1.665	-1.903	-1.674	0.179	0.230

Q08945	SSRP1	5.767	5.697	5.697	5.614	5.732	5.655	0.297	-0.077
Q96CN5	LRRC45	-4.094	-3.991	-4.260	-4.172	-4.042	-4.216	0.129	-0.174
P29692	EEF1D	-0.519	-0.553	-0.573	-0.571	-0.536	-0.572	0.280	-0.036
Q99598	TSNAX	3.501	3.473	3.523	3.528	3.487	3.526	0.215	0.038
Q9NRX4	PHPT1	-0.372	-0.508	-0.355	-0.623	-0.440	-0.489	0.784	-0.049
O60294	LCMT2	-2.576	-2.613	-2.631	-2.667	-2.594	-2.649	0.169	-0.055
P61247	RPS3A	6.647	6.560	6.657	6.558	6.604	6.608	0.958	0.004
Q15276	RABEP1	-0.870	-1.050	-0.918	-0.966	-0.960	-0.942	0.873	0.018
Q14568	HSP90AA2P	-2.373	-2.691	-2.554	-2.619	-2.532	-2.586	0.793	-0.054
Q9BQB6	VKORC1	-2.339	-2.151	-2.410	-2.019	-2.245	-2.215	0.907	0.030
P07311	ACYP1	0.219	0.174	0.077	0.113	0.196	0.095	0.078	-0.102
Q3SXM5	HSDL1	0.186	0.131	0.276	0.284	0.158	0.280	0.136	0.121
P49914	MTHFS	-3.401	-3.327	-3.244	-3.356	-3.364	-3.300	0.458	0.064
Q15382	RHEB	3.253	3.169	3.084	3.040	3.211	3.062	0.126	-0.149
Q8WWM7	ATXN2L	-2.441	-2.325	-2.466	-2.491	-2.383	-2.479	0.335	-0.096
Q12846	STX4	-2.262	-2.309	-2.032	-2.088	-2.285	-2.060	0.028	0.225
Q13112	CHAF1B	0.741	0.736	0.516	0.528	0.739	0.522	0.007	-0.217
Q9Y6G3	MRPL42	2.526	2.384	2.551	2.474	2.455	2.513	0.568	0.058
Q14442	PIGH	-3.180	-3.273	-3.290	-3.256	-3.226	-3.273	0.490	-0.047
P13984	GTF2F2	2.376	2.330	2.368	2.390	2.353	2.379	0.457	0.025
Q9Y6E2	BZW2	3.468	3.344	3.335	3.307	3.406	3.321	0.392	-0.085
Q8N4T8	CBR4	-1.745	-1.609	-1.519	-1.576	-1.677	-1.547	0.277	0.130
Q9C0B0	UNK	0.727	0.707	0.630	0.731	0.717	0.680	0.596	-0.037
Q9NVV0	TMEM38B	1.227	1.199	1.205	1.198	1.213	1.202	0.563	-0.011
Q96D09	GPRASP2	-0.918	-1.053	-0.847	-0.874	-0.986	-0.861	0.306	0.125
P57764	GSDMD	-2.831	-2.799	-2.583	-3.014	-2.815	-2.798	0.952	0.016
P40425	PBX2	-3.521	-3.566	-3.495	-3.482	-3.543	-3.488	0.230	0.055
Q9UBU6	FAM8A1	-1.795	-1.775	-1.785	-1.705	-1.785	-1.745	0.498	0.040
P28066	PSMA5	3.701	3.652	3.463	3.489	3.677	3.476	0.037	-0.201
Q9UHD9	UBQLN2	1.706	1.775	1.577	1.793	1.741	1.685	0.699	-0.056
Q9UBR2	CTSZ	2.145	1.866	2.132	1.800	2.005	1.966	0.872	-0.040
Q99519	NEU1	-1.142	-1.197	-0.996	-1.034	-1.170	-1.015	0.054	0.155
Q9GZY8	MFF	-3.903	-3.957	-3.754	-3.764	-3.930	-3.759	0.093	0.171
O95433	AHSA1	4.652	4.613	4.627	4.559	4.632	4.593	0.441	-0.039
Q9H3J6	MTRFR	-2.550	-2.981	-2.525	-2.875	-2.765	-2.700	0.836	0.065

P43007	SLC1A4	-0.582	-0.364	-0.339	-0.277	-0.473	-0.308	0.359	0.165
Q9H0J9	PARP12	-1.387	-1.400	-1.184	-1.188	-1.393	-1.186	0.010	0.207
P60520	GABARAPL2	0.725	0.637	0.572	0.575	0.681	0.573	0.249	-0.108
Q9BYN8	MRPS26	1.895	1.919	1.867	1.829	1.907	1.848	0.146	-0.059
Q8WV93	AFG1L	0.066	-0.038	0.442	0.436	0.014	0.439	0.077	0.425
Q96H35	RBM18	-3.015	-2.955	-2.837	-2.752	-2.985	-2.794	0.078	0.191
Q9NYK5	MRPL39	-0.257	-0.819	-0.135	-0.741	-0.538	-0.438	0.831	0.100
P30419	NMT1	-0.254	-0.338	-0.243	-0.349	-0.296	-0.296	0.997	0.000
O94761	RECQL4	-0.438	-0.385	-0.532	-0.532	-0.412	-0.532	0.138	-0.121
P16435	POR	4.943	4.858	5.054	4.979	4.901	5.017	0.180	0.116
Q8NCN4	RNF169	0.470	0.502	0.460	0.473	0.486	0.466	0.422	-0.020
Q12766	HMGXB3	-3.821	-3.821	-3.809	-3.784	-3.821	-3.796	0.300	0.025
O95685	PPP1R3D	-1.574	-1.494	-1.605	-1.475	-1.534	-1.540	0.947	-0.006
O75208	COQ9	2.458	2.313	2.498	2.497	2.386	2.498	0.365	0.112
Q9UK22	FBXO2	-0.276	-0.275	-0.297	-0.249	-0.276	-0.273	0.922	0.003
Q6FI81	CIAPIN1	-1.021	-1.167	-1.195	-1.161	-1.094	-1.178	0.448	-0.084
P78346	RPP30	-4.059	-4.138	-4.154	-4.002	-4.099	-4.078	0.839	0.021
Q8NEC7	GSTCD	-2.054	-1.975	-2.110	-1.892	-2.015	-2.001	0.919	0.014
Q9BU89	DOHH	0.442	0.378	0.338	0.334	0.410	0.336	0.256	-0.074
P55064	AQP5	-2.637	-2.110	-2.739	-2.435	-2.374	-2.587	0.571	-0.213
Q96CW5	TUBGCP3	-2.227	-2.121	-2.063	-2.124	-2.174	-2.094	0.345	0.080
O43583	DENR	2.972	2.879	2.943	2.905	2.926	2.924	0.976	-0.002
P41236	PPP1R2	1.801	1.934	1.901	1.965	1.867	1.933	0.498	0.066
Q6P1J9	CDC73	3.240	3.194	3.222	3.187	3.217	3.205	0.711	-0.012
Q6ZU35	CRACD	-4.567	-4.757	-4.613	-4.446	-4.662	-4.529	0.405	0.133
Q6IBS0	TWF2	2.507	2.471	2.326	2.323	2.489	2.324	0.068	-0.165
P21741	MDK	-3.065	-2.791	-2.514	-2.819	-2.928	-2.666	0.331	0.262
Q9UMY1	NOL7	2.146	1.994	2.160	2.075	2.070	2.118	0.652	0.047
Q9H8H2	DDX31	-0.313	-0.273	-0.319	-0.309	-0.293	-0.314	0.481	-0.021
Q86XP3	DDX42	0.972	0.865	1.024	0.941	0.918	0.982	0.450	0.064
Q9NRS6	SNX15	-3.347	-3.406	-3.228	-3.298	-3.377	-3.263	0.137	0.114
P42685	FRK	-4.605	-4.602	-4.479	-4.505	-4.604	-4.492	0.073	0.112
P84090	ERH	2.495	2.408	2.608	2.528	2.451	2.568	0.187	0.117
Q96EK5	KIFBP	2.435	2.418	2.413	2.384	2.426	2.399	0.271	-0.027
Q02040	AKAP17A	-0.255	-0.352	-0.192	-0.228	-0.304	-0.210	0.277	0.094

P21266	GSTM3	4.633	4.659	4.450	4.493	4.646	4.471	0.033	-0.174
Q68DH5	LMBRD2	-0.735	-0.817	-0.483	-0.541	-0.776	-0.512	0.043	0.264
Q15648	MED1	1.454	1.387	1.413	1.384	1.420	1.398	0.629	-0.022
Q32P44	EML3	-1.179	-1.185	-0.992	-0.963	-1.182	-0.977	0.038	0.204
P35237	SERPINB6	5.390	5.317	5.687	5.661	5.353	5.674	0.048	0.321
P30153	PPP2R1A	5.834	5.846	5.830	5.819	5.840	5.825	0.192	-0.015
P20700	LMNB1	6.487	6.359	6.577	6.492	6.423	6.535	0.301	0.112
P82663	MRPS25	-0.023	-0.054	-0.258	-0.180	-0.038	-0.219	0.100	-0.181
P62487	POLR2G	1.383	1.394	1.346	1.355	1.388	1.350	0.036	-0.038
Q9HBH5	RDH14	1.989	2.001	2.013	2.064	1.995	2.038	0.332	0.043
Q9H9L3	ISG20L2	1.704	1.713	1.736	1.739	1.709	1.738	0.083	0.029
Q9Y5S9	RBM8A	-1.867	-1.918	-1.775	-1.900	-1.892	-1.838	0.538	0.054
O14647	CHD2	-3.503	-3.570	-3.504	-3.426	-3.536	-3.465	0.302	0.071
O94919	ENDOD1	1.649	1.708	1.807	1.782	1.678	1.794	0.116	0.116
Q9ULI3	HEG1	-5.514	-5.321	-5.512	-5.439	-5.418	-5.476	0.657	-0.058
O43617	TRAPPC3	-1.903	-2.137	-2.038	-2.270	-2.020	-2.154	0.501	-0.134
Q9NP97	DYNLRB1	0.964	0.901	0.859	0.978	0.933	0.918	0.856	-0.014
Q9P0L0	VAPA	1.840	1.761	1.837	1.735	1.801	1.786	0.847	-0.014
Q9BSL1	UBAC1	0.242	0.221	0.191	0.237	0.232	0.214	0.585	-0.018
O43731	KDELR3	-2.722	-2.794	-2.965	-2.908	-2.758	-2.937	0.067	-0.178
O14908	GIPC1	0.324	0.277	0.375	0.367	0.301	0.371	0.199	0.071
Q9UN79	SOX13	-3.825	-3.570	-3.793	-3.262	-3.698	-3.527	0.640	0.171
O75792	RNASEH2A	2.240	2.333	2.226	2.150	2.287	2.188	0.247	-0.099
Q9BTL3	RAMAC	1.263	1.164	1.456	1.361	1.213	1.408	0.105	0.195
Q8TEA8	DTD1	1.879	1.729	1.844	1.741	1.804	1.793	0.913	-0.011
P17252	PRKCA	1.663	1.632	1.906	1.913	1.648	1.909	0.029	0.262
P00558	PGK1	-0.157	-0.040	-0.056	0.013	-0.099	-0.021	0.393	0.078
P63313	TMSB10	1.133	0.981	1.113	1.029	1.057	1.071	0.892	0.014
P98179	RBM3	1.997	2.003	1.627	1.660	2.000	1.643	0.025	-0.356
Q5VUM1	SDHAF4	-1.019	-1.331	-1.521	-1.311	-1.175	-1.416	0.343	-0.241
P46977	STT3A	2.256	2.223	2.187	2.186	2.240	2.187	0.192	-0.053
Q9Y2C4	EXOG	-1.458	-1.444	-1.603	-1.540	-1.451	-1.571	0.149	-0.120
P13284	IFI30	-1.326	-1.074	-1.459	-0.879	-1.200	-1.169	0.934	0.031
P61163	ACTR1A	2.200	2.030	2.101	2.047	2.115	2.074	0.714	-0.041
Q15678	PTPN14	1.096	1.067	0.910	0.938	1.081	0.924	0.016	-0.157

Q06203	PPAT	2.917	2.863	2.973	2.891	2.890	2.932	0.494	0.042
P32780	GTF2H1	-0.533	-0.577	-0.619	-0.653	-0.555	-0.636	0.110	-0.080
Q9NUQ3	TXLNG	-0.168	-0.327	-0.259	-0.254	-0.247	-0.257	0.925	-0.009
Q6UN15	FIP1L1	-2.636	-2.562	-2.516	-2.517	-2.599	-2.517	0.269	0.082
P34932	HSPA4	7.202	7.114	7.106	7.107	7.158	7.107	0.449	-0.051
P49454	CENPF	2.620	2.637	2.451	2.459	2.628	2.455	0.013	-0.174
P14649	MYL6B	-1.281	-1.264	-1.334	-1.248	-1.273	-1.291	0.744	-0.018
P16152	CBR1	5.003	4.847	4.924	4.848	4.925	4.886	0.708	-0.039
P47756	CAPZB	2.433	2.351	2.320	2.317	2.392	2.318	0.321	-0.074
Q9NP79	VTA1	2.944	2.910	2.886	2.785	2.927	2.835	0.297	-0.092
Q14703	MBTPS1	-2.569	-2.636	-2.472	-2.486	-2.603	-2.479	0.156	0.124
O60292	SIPA1L3	1.173	1.145	1.171	1.123	1.159	1.147	0.718	-0.012
Q9Y5Z7	HCFC2	-1.659	-1.864	-1.643	-1.774	-1.761	-1.709	0.713	0.053
C9JLW8	MCRIP1	2.345	2.452	2.050	2.243	2.399	2.147	0.184	-0.252
Q9NZJ6	COQ3	0.227	0.165	0.205	0.197	0.196	0.201	0.891	0.005
O75347	TBCA	3.108	2.912	2.945	2.823	3.010	2.884	0.408	-0.126
P53618	COPB1	5.190	5.121	5.136	5.107	5.155	5.121	0.493	-0.034
Q9BXK1	KLF16	-0.266	-0.379	-0.221	-0.176	-0.322	-0.198	0.240	0.124
Q15126	PMVK	2.450	2.444	2.314	2.286	2.447	2.300	0.051	-0.147
Q9Y3D5	MRPS18C	-0.202	-0.246	-0.286	-0.287	-0.224	-0.287	0.213	-0.062
O43543	XRCC2	-3.734	-3.706	-3.828	-3.743	-3.720	-3.785	0.351	-0.066
Q00577	PURA	1.799	1.695	1.761	1.653	1.747	1.707	0.647	-0.040
P60891	PRPS1	-1.423	-1.406	-1.556	-1.522	-1.415	-1.539	0.044	-0.125
Q93096	PTP4A1	-0.290	-0.283	-0.342	-0.361	-0.287	-0.352	0.066	-0.065
Q9C0J9	BHLHE41	-5.318	-4.940	-5.130	-5.025	-5.129	-5.077	0.831	0.052
Q49AG3	ZBED5	-2.576	-2.533	-2.498	-2.273	-2.554	-2.386	0.368	0.169
P55039	DRG2	2.601	2.572	2.539	2.600	2.587	2.570	0.683	-0.017
Q9BT09	CNPY3	0.544	1.038	0.786	0.849	0.791	0.818	0.932	0.026
A6NK58	LIPT2	-3.481	-3.333	-3.374	-3.174	-3.407	-3.274	0.403	0.133
Q9HCD5	NCOA5	2.459	2.505	2.467	2.473	2.482	2.470	0.697	-0.012
Q00059	TFAM	2.346	2.214	2.516	2.349	2.280	2.432	0.296	0.152
O96011	PEX11B	-2.630	-2.518	-2.628	-2.364	-2.574	-2.496	0.663	0.078
P01009	SERPINA1	-2.059	-1.900	-1.964	-1.966	-1.980	-1.965	0.883	0.015
P51812	RPS6KA3	3.051	3.056	3.078	3.045	3.053	3.062	0.707	0.008
O15213	WDR46	2.905	2.836	2.841	2.884	2.871	2.863	0.865	-0.008

P63104	YWHAZ	5.126	5.311	5.103	5.055	5.218	5.079	0.361	-0.140
Q9Y3C7	MED31	-2.678	-2.624	-2.864	-2.676	-2.651	-2.770	0.415	-0.119
Q9BUE0	MED18	-0.163	0.041	-0.070	-0.165	-0.061	-0.118	0.681	-0.057
Q6IQ21	ZNF770	-1.102	-1.083	-1.091	-1.145	-1.092	-1.118	0.511	-0.026
P49257	LMAN1	3.753	3.767	3.698	3.815	3.760	3.757	0.963	-0.003
P09110	ACAA1	-0.123	0.056	-0.096	-0.024	-0.033	-0.060	0.818	-0.027
P61981	YWHAG	6.620	6.595	6.730	6.744	6.608	6.737	0.024	0.129
Q9Y4R8	TELO2	2.029	2.012	1.834	1.868	2.020	1.851	0.028	-0.169
Q92598	HSPH1	-5.788	-5.373	-5.833	-5.891	-5.580	-5.862	0.401	-0.281
Q53EP0	FNDC3B	-0.713	-0.603	-0.532	-0.527	-0.658	-0.530	0.257	0.128
Q9NRW7	VPS45	0.528	0.379	0.327	0.331	0.453	0.329	0.345	-0.124
Q92628	KIAA0232	-2.871	-2.909	-2.895	-2.929	-2.890	-2.912	0.478	-0.022
O43252	PAPSS1	2.941	2.886	2.923	2.932	2.914	2.927	0.702	0.014
P26373	RPL13	1.588	1.645	1.407	1.528	1.616	1.467	0.205	-0.149
Q9BTC0	DIDO1	1.057	1.161	1.077	1.073	1.109	1.075	0.633	-0.034
A1A4S6	ARHGAP10	-1.166	-1.231	-1.338	-1.374	-1.199	-1.356	0.079	-0.157
Q5T2D3	OTUD3	-3.401	-3.380	-3.381	-3.368	-3.390	-3.375	0.353	0.016
Q8TED1	GPX8	1.600	1.504	1.558	1.489	1.552	1.524	0.682	-0.029
Q8NAT1	POMGNT2	-1.804	-1.878	-1.744	-1.840	-1.841	-1.792	0.507	0.049
P30740	SERPINB1	3.138	3.084	3.549	3.494	3.111	3.521	0.009	0.411
O75414	NME6	-1.344	-1.268	-1.278	-1.105	-1.306	-1.192	0.395	0.114
Q9H967	WDR76	-0.211	-0.334	-0.293	-0.473	-0.273	-0.383	0.431	-0.110
Q8IUX4	APOBEC3F	-0.212	-0.249	-0.198	-0.153	-0.231	-0.176	0.202	0.055
Q9H814	PHAX	2.160	2.091	2.000	1.941	2.125	1.970	0.078	-0.155
Q9BTV6	DPH7	-2.712	-2.754	-2.796	-2.804	-2.733	-2.800	0.183	-0.067
Q9NQT4	EXOSC5	1.672	1.528	1.626	1.532	1.600	1.579	0.833	-0.021
P46531	NOTCH1	-0.311	-0.488	-0.380	-0.399	-0.400	-0.390	0.929	0.010
O00471	EXOC5	2.465	2.485	2.381	2.441	2.475	2.411	0.256	-0.064
P53985	SLC16A1	0.780	0.523	0.632	0.458	0.651	0.545	0.572	-0.106
Q7Z6Z7	HUWE1	-5.767	-5.788	-6.105	-5.815	-5.777	-5.960	0.427	-0.182
Q8IY92	SLX4	-3.713	-3.573	-3.753	-3.591	-3.643	-3.672	0.813	-0.029
Q15596	NCOA2	-0.733	-0.684	-0.655	-0.553	-0.709	-0.604	0.252	0.104
P53370	NUDT6	-3.633	-3.687	-3.708	-3.661	-3.660	-3.684	0.567	-0.024
Q8TBM8	DNAJB14	-1.571	-1.692	-1.497	-1.598	-1.631	-1.548	0.400	0.084
P46108	CRK	0.879	0.886	0.908	0.951	0.883	0.930	0.266	0.047

Q15758	SLC1A5	0.403	0.537	0.651	0.620	0.470	0.636	0.231	0.165
Q14691	GINS1	1.161	1.253	0.866	1.000	1.207	0.933	0.093	-0.274
Q9UBB4	ATXN10	0.896	0.739	1.102	0.953	0.818	1.027	0.193	0.210
O75616	ERAL1	0.055	0.007	-0.180	0.029	0.031	-0.075	0.489	-0.107
O95747	OXSR1	1.883	1.864	1.895	1.904	1.874	1.900	0.185	0.026
P41240	CSK	2.453	2.443	2.420	2.363	2.448	2.391	0.289	-0.056
Q96RE7	NACC1	1.162	1.119	0.999	1.040	1.140	1.020	0.055	-0.121
Q9BQ48	MRPL34	-0.675	-0.962	-0.609	-0.894	-0.818	-0.751	0.772	0.067
Q6P1R4	DUS1L	0.834	0.736	0.758	0.685	0.785	0.721	0.416	-0.063
Q15697	ZNF174	-4.373	-4.470	-4.323	-4.325	-4.422	-4.324	0.291	0.098
Q9NYY3	PLK2	0.679	0.720	0.228	0.228	0.699	0.228	0.027	-0.472
P30536	TSPO	2.375	2.293	2.403	2.441	2.334	2.422	0.242	0.088
O94907	DKK1	-1.769	-1.695	-2.392	-2.341	-1.732	-2.367	0.008	-0.635
A6NHC0	CAPN8	-4.376	-4.313	-4.278	-4.146	-4.345	-4.212	0.258	0.133
Q16740	CLPP	2.037	2.142	2.208	2.268	2.089	2.238	0.163	0.149
P63173	RPL38	5.336	5.365	5.323	5.309	5.351	5.316	0.222	-0.035
Q5SNT2	TMEM201	-1.157	-1.151	-0.958	-0.853	-1.154	-0.905	0.132	0.249
Q9H0U4	RAB1B	1.073	1.129	1.044	1.001	1.101	1.023	0.168	-0.078
O15525	MAFG	-1.530	-1.250	-1.264	-1.208	-1.390	-1.236	0.465	0.155
P56377	AP1S2	-4.836	-4.652	-5.092	-4.486	-4.744	-4.789	0.908	-0.045
P30825	SLC7A1	1.560	1.535	1.619	1.608	1.548	1.614	0.080	0.066
Q99698	LYST	-3.202	-3.168	-3.386	-3.226	-3.185	-3.306	0.362	-0.121
Q9UM54	MYO6	-3.863	-4.211	-3.820	-3.869	-4.037	-3.845	0.466	0.192
P17098	ZNF8	-1.730	-1.827	-1.752	-1.787	-1.779	-1.769	0.878	0.010
Q8N806	UBR7	1.384	1.460	1.321	1.293	1.422	1.307	0.171	-0.115
P18124	RPL7	7.215	7.208	7.242	7.205	7.212	7.223	0.649	0.012
Q5VYK3	ECPAS	5.129	5.032	5.129	5.087	5.080	5.108	0.675	0.027
Q96ER9	CCDC51	-0.454	-0.331	-0.522	-0.228	-0.393	-0.375	0.927	0.017
O14817	TSPAN4	-0.681	-0.864	-0.850	-0.956	-0.772	-0.903	0.367	-0.131
Q8IWP9	CCDC28A	-1.817	-1.808	-1.414	-1.485	-1.812	-1.450	0.059	0.363
Q01581	HMGCS1	0.790	0.997	0.807	0.794	0.894	0.801	0.533	-0.093
P23284	PPIB	6.883	6.815	6.753	6.670	6.849	6.712	0.130	-0.137
P54105	CLNS1A	1.308	1.305	1.042	1.089	1.306	1.065	0.062	-0.241
Q5UIP0	RIF1	-1.220	-1.267	-1.341	-1.276	-1.243	-1.309	0.255	-0.065
P43490	NAMPT	5.820	5.672	6.637	6.509	5.746	6.573	0.015	0.827

Q9UBM7	DHCR7	2.392	2.339	2.587	2.410	2.366	2.498	0.359	0.133
P53004	BLVRA	4.641	4.596	4.547	4.537	4.619	4.542	0.166	-0.077
Q9HAN9	NMNAT1	0.033	-0.033	0.237	0.120	0.000	0.179	0.149	0.179
Q5T2T1	MPP7	-3.578	-2.888	-3.323	-3.382	-3.233	-3.353	0.788	-0.120
Q96HE9	PRR11	-1.049	-0.998	-1.130	-1.171	-1.024	-1.151	0.067	-0.127
Q8IUR0	TRAPPC5	1.434	1.284	1.341	1.317	1.359	1.329	0.756	-0.030
Q6PIJ6	FBXO38	-4.038	-3.951	-4.024	-3.949	-3.995	-3.987	0.903	0.008
Q9P2G1	ANKIB1	-1.210	-1.179	-1.269	-1.054	-1.195	-1.161	0.810	0.033
Q9Y5P8	PPP2R3B	-2.475	-2.440	-2.542	-2.346	-2.457	-2.444	0.915	0.013
P17029	ZKSCAN1	-0.645	-0.614	-0.470	-0.426	-0.630	-0.448	0.026	0.182
P54652	HSPA2	-2.065	-2.166	-2.152	-2.254	-2.116	-2.203	0.349	-0.088
O95059	RPP14	-0.924	-0.791	-1.018	-0.775	-0.858	-0.896	0.813	-0.039
Q9HAV0	GNB4	-4.093	-4.213	-3.671	-4.022	-4.153	-3.846	0.310	0.307
Q9HAU4	SMURF2	-0.406	-0.442	-0.624	-0.487	-0.424	-0.556	0.290	-0.132
Q92917	GPKOW	3.064	3.059	2.946	2.995	3.061	2.970	0.164	-0.091
P51687	SUOX	-0.786	-0.915	-0.818	-0.882	-0.850	-0.850	0.996	0.000
Q7Z2Z2	EFL1	-2.555	-2.659	-2.385	-2.603	-2.607	-2.494	0.481	0.113
P48163	ME1	-3.535	-3.535	-3.833	-3.807	-3.535	-3.820	0.029	-0.285
O60583	CCNT2	-4.708	-4.662	-4.819	-4.785	-4.685	-4.802	0.063	-0.117
P35659	DEK	1.477	1.543	1.458	1.433	1.510	1.445	0.273	-0.065
O95081	AGFG2	-1.168	-1.270	-1.078	-1.115	-1.219	-1.096	0.225	0.122
Q9BUB4	ADAT1	-2.046	-2.050	-2.083	-2.084	-2.048	-2.084	0.023	-0.035
O00443	PIK3C2A	1.835	1.786	2.260	2.198	1.811	2.229	0.011	0.418
P78356	PIP4K2B	-2.910	-3.069	-3.014	-2.984	-2.989	-2.999	0.925	-0.009
Q66K64	DCAF15	-4.018	-3.901	-3.861	-3.990	-3.959	-3.925	0.737	0.034
O15078	CEP290	-1.007	-1.011	-1.048	-0.968	-1.009	-1.008	0.989	0.001
Q9UQ80	PA2G4	1.865	1.955	2.007	1.941	1.910	1.974	0.376	0.064
P07858	CTSB	2.675	2.802	2.947	2.984	2.739	2.966	0.152	0.227
Q9HA77	CARS2	2.261	2.178	2.202	2.187	2.219	2.195	0.654	-0.025
Q92545	TMEM131	-1.037	-0.946	-0.899	-0.797	-0.992	-0.848	0.171	0.144
Q86UT8	CENATAC	-1.871	-1.956	-2.212	-2.123	-1.913	-2.168	0.054	-0.254
P50914	RPL14	6.249	6.147	6.268	6.250	6.198	6.259	0.440	0.061
A2RU67	FAM234B	-1.322	-1.419	-1.377	-1.466	-1.371	-1.421	0.525	-0.050
Q14566	MCM6	5.553	5.550	5.398	5.368	5.552	5.383	0.053	-0.169
Q9ULM3	YEATS2	0.362	0.286	0.294	0.337	0.324	0.316	0.872	-0.008

O77932	DXO	-1.831	-1.894	-1.781	-1.823	-1.863	-1.802	0.270	0.060
Q8N302	AGGF1	-2.251	-2.172	-2.445	-2.344	-2.211	-2.395	0.111	-0.183
Q03468	ERCC6	-1.174	-1.080	-0.865	-0.845	-1.127	-0.855	0.097	0.272
A2RUC4	TYW5	-2.860	-2.792	-2.838	-2.787	-2.826	-2.812	0.785	0.013
Q9NXF7	DCAF16	-0.395	-0.356	-0.571	-0.482	-0.376	-0.527	0.143	-0.151
Q6IN84	MRM1	-0.876	-0.734	-0.716	-0.843	-0.805	-0.780	0.814	0.026
O95182	NDUFA7	1.864	1.785	1.996	1.963	1.825	1.980	0.120	0.155
Q6IPR1	ETFRF1	-0.659	-0.018	-0.263	-0.284	-0.339	-0.273	0.872	0.065
Q9BX70	BTBD2	-2.126	-2.165	-2.278	-2.254	-2.145	-2.266	0.049	-0.121
Q9NUQ9	CYRIB	2.181	2.219	2.142	2.104	2.200	2.123	0.104	-0.077
Q6NVH7	SWSAP1	-3.113	-3.209	-3.171	-3.088	-3.161	-3.129	0.667	0.032
P07204	THBD	-5.623	-4.920	-5.380	-5.115	-5.271	-5.247	0.958	0.024
O14595	CTDSP2	-2.598	-2.364	-2.587	-2.381	-2.481	-2.484	0.986	-0.003
O75817	POP7	-1.037	-0.986	-0.893	-0.867	-1.012	-0.880	0.074	0.132
O75841	UPK1B	-3.260	-2.584	-2.977	-2.803	-2.922	-2.890	0.940	0.032
Q9UM13	ANAPC10	-1.489	-1.441	-1.564	-1.419	-1.465	-1.491	0.780	-0.026
Q9NYL2	MAP3K20	-0.187	-0.201	-0.111	-0.115	-0.194	-0.113	0.040	0.081
Q14781	CBX2	-2.211	-2.312	-2.298	-2.440	-2.261	-2.369	0.352	-0.108
P13498	CYBA	-0.375	-0.486	-0.283	-0.366	-0.430	-0.325	0.278	0.106
Q9BV73	CEP250	-2.236	-2.280	-2.240	-2.270	-2.258	-2.255	0.925	0.003
Q9ULE6	PALD1	1.871	1.806	1.719	1.737	1.838	1.728	0.163	-0.111
O15318	POLR3G	-2.521	-2.482	-2.594	-2.446	-2.501	-2.520	0.844	-0.019
P50613	CDK7	1.649	1.563	1.662	1.635	1.606	1.648	0.499	0.042
O94776	MTA2	0.526	0.526	0.470	0.495	0.526	0.483	0.178	-0.043
Q5VZE5	NAA35	1.978	2.027	1.968	1.997	2.002	1.983	0.573	-0.020
P19474	TRIM21	-2.076	-2.065	-1.776	-1.852	-2.071	-1.814	0.088	0.257
P56589	PEX3	1.359	1.355	1.310	1.383	1.357	1.347	0.830	-0.010
O75363	BCAS1	-4.821	-4.692	-4.570	-4.554	-4.757	-4.562	0.199	0.194
P09493	TPM1	-4.260	-4.253	-4.318	-4.416	-4.257	-4.367	0.265	-0.110
P23434	GCSH	3.457	3.459	3.447	3.598	3.458	3.523	0.551	0.065
Q8NB14	USP38	-3.979	-4.300	-4.368	-4.058	-4.139	-4.213	0.771	-0.074
Q8IYN2	TCEAL8	-3.408	-3.400	-3.534	-3.296	-3.404	-3.415	0.942	-0.011
O43464	HTRA2	-2.610	-2.442	-2.763	-2.493	-2.526	-2.628	0.598	-0.102
Q9P035	HACD3	1.416	1.539	1.384	1.403	1.477	1.394	0.401	-0.084
Q9UH65	SWAP70	3.073	2.987	3.076	3.016	3.030	3.046	0.789	0.016

Q9UPT8	ZC3H4	2.672	2.685	2.661	2.659	2.679	2.660	0.211	-0.019
Q969H8	MYDGF	3.166	3.143	3.057	3.070	3.155	3.064	0.034	-0.091
Q9H0S4	DDX47	-1.164	-1.171	-1.248	-1.223	-1.168	-1.236	0.096	-0.068
Q8TF74	WIPF2	2.088	2.114	2.115	2.129	2.101	2.122	0.332	0.021
Q8N142	ADSS1	-3.120	-3.107	-3.282	-3.299	-3.113	-3.290	0.006	-0.177
Q9Y289	SLC5A6	-1.523	-1.623	-1.668	-1.419	-1.573	-1.544	0.856	0.029
Q6PJ69	TRIM65	-0.045	0.108	0.058	0.111	0.031	0.084	0.612	0.053
P84022	SMAD3	-1.659	-1.080	-1.590	-1.479	-1.370	-1.534	0.670	-0.165
P52758	RIDA	1.223	1.209	1.380	1.332	1.216	1.356	0.084	0.140
O15534	PER1	-3.350	-3.463	-3.448	-3.425	-3.407	-3.436	0.693	-0.030
P13726	F3	-3.706	-3.261	-3.239	-3.688	-3.483	-3.464	0.956	0.020
O75695	RP2	3.540	3.502	3.466	3.454	3.521	3.460	0.164	-0.061
Q15643	TRIP11	-1.207	-1.168	-1.328	-1.317	-1.188	-1.322	0.072	-0.135
P11586	MTHFD1	6.315	6.360	6.218	6.202	6.338	6.210	0.082	-0.128
O00515	LAD1	0.533	1.278	0.785	0.921	0.906	0.853	0.910	-0.053
O43299	AP5Z1	-0.345	-0.346	-0.305	-0.294	-0.346	-0.300	0.071	0.046
Q9UKZ1	CNOT11	-0.240	-0.093	-0.177	-0.057	-0.166	-0.117	0.656	0.049
Q8WU67	ABHD3	-2.786	-2.877	-2.918	-2.790	-2.831	-2.854	0.799	-0.023
Q5SW96	LDLRAP1	-0.652	-0.676	-0.834	-0.726	-0.664	-0.780	0.266	-0.116
Q8IUE6	H2AC21	-1.917	-1.836	-1.891	-1.758	-1.876	-1.825	0.588	0.052
O75161	NPHP4	-3.859	-3.926	-3.861	-3.848	-3.892	-3.855	0.457	0.038
O43292	GPAA1	-0.739	-0.681	-0.814	-0.751	-0.710	-0.783	0.232	-0.072
Q9NRD5	PICK1	-3.142	-3.174	-3.154	-3.154	-3.158	-3.154	0.842	0.004
P34913	EPHX2	-0.585	-0.647	-0.638	-0.757	-0.616	-0.697	0.381	-0.081
Q9UJV9	DDX41	3.161	3.126	3.157	3.155	3.144	3.156	0.597	0.013
Q96NL6	SCLT1	-3.143	-2.867	-3.138	-3.167	-3.005	-3.152	0.479	-0.147
O75934	BCAS2	2.845	2.848	2.732	2.797	2.847	2.764	0.240	-0.082
O94901	SUN1	-3.700	-3.412	-3.843	-3.445	-3.556	-3.644	0.758	-0.088
P55318	FOXA3	-5.878	-5.026	-5.747	-5.493	-5.452	-5.620	0.762	-0.168
P13073	COX4I1	3.811	3.660	3.796	3.690	3.735	3.743	0.944	0.007
Q12788	TBL3	3.728	3.663	3.764	3.768	3.695	3.766	0.271	0.071
P22102	GART	5.504	5.453	5.431	5.381	5.479	5.406	0.179	-0.073
P62330	ARF6	3.695	3.606	3.511	3.464	3.651	3.488	0.120	-0.163
P10768	ESD	4.144	4.062	4.125	4.051	4.103	4.088	0.807	-0.015
Q9H9P8	L2HGDH	-0.171	-0.206	-0.401	-0.394	-0.189	-0.398	0.045	-0.209

Q96B70	LENG9	0.000	-0.110	0.040	0.075	-0.055	0.058	0.268	0.112
Q96AE4	FUBP1	-0.595	-0.591	-0.454	-0.532	-0.593	-0.493	0.235	0.100
Q9Y266	NUDC	5.025	4.915	4.654	4.611	4.970	4.632	0.071	-0.337
P55795	HNRNPH2	3.918	3.901	4.085	4.063	3.909	4.074	0.009	0.165
Q9NQG5	RPRD1B	3.105	3.145	3.145	3.095	3.125	3.120	0.889	-0.005
Q12849	GRSF1	-6.089	-6.234	-6.053	-6.273	-6.162	-6.163	0.993	-0.001
Q8IYU8	MICU2	0.918	0.868	0.861	0.834	0.893	0.847	0.282	-0.046
O95219	SNX4	0.159	-0.049	0.334	0.321	0.055	0.328	0.231	0.272
Q8IZ81	ELMOD2	-0.227	-0.218	-0.195	-0.141	-0.222	-0.168	0.283	0.054
P61803	DAD1	2.022	1.911	1.948	1.934	1.967	1.941	0.724	-0.026
Q9Y6N5	SQOR	3.977	3.963	4.094	3.998	3.970	4.046	0.354	0.076
Q14683	SMC1A	4.702	4.779	4.759	4.726	4.740	4.742	0.963	0.002
Q9BRJ6	C7orf50	1.537	1.511	1.391	1.436	1.524	1.413	0.074	-0.110
A6NGB9	WIPF3	-5.026	-4.937	-4.665	-4.564	-4.982	-4.614	0.033	0.367
Q02241	KIF23	-1.893	-1.892	-1.969	-1.972	-1.893	-1.971	0.011	-0.078
Q9BV68	RNF126	-1.304	-1.360	-1.488	-1.485	-1.332	-1.487	0.113	-0.154
P21281	ATP6V1B2	3.041	2.946	3.000	2.969	2.993	2.985	0.889	-0.008
Q6NZY4	ZCCHC8	0.612	0.664	0.750	0.877	0.638	0.814	0.183	0.176
Q9UIA9	XPO7	4.478	4.405	4.392	4.393	4.441	4.393	0.409	-0.049
Q9NXH8	TOR4A	1.707	1.606	1.662	1.609	1.656	1.635	0.755	-0.021
Q96EU7	C1GALT1C1	-3.542	-3.375	-3.368	-3.403	-3.459	-3.386	0.541	0.073
O75563	SKAP2	1.917	1.795	1.795	1.778	1.856	1.787	0.455	-0.070
Q13148	TARDBP	1.123	0.965	1.046	0.995	1.044	1.021	0.818	-0.023
Q9UL26	RAB22A	0.057	0.091	0.047	0.044	0.074	0.046	0.348	-0.028
Q9BS16	CENPK	-3.202	-3.247	-3.316	-3.297	-3.225	-3.306	0.127	-0.082
Q9Y3U8	RPL36	3.942	3.872	3.944	3.826	3.907	3.885	0.788	-0.022
Q9UDW1	UQCR10	-0.100	-0.059	-0.087	-0.079	-0.079	-0.083	0.879	-0.004
Q9BZV1	UBXN6	-1.198	-1.455	-0.928	-1.169	-1.326	-1.048	0.256	0.278
O60218	AKR1B10	-2.987	-2.941	-3.099	-3.055	-2.964	-3.077	0.070	-0.113
Q2TBE0	CWF19L2	-2.306	-2.458	-2.312	-2.316	-2.382	-2.314	0.535	0.068
P62266	RPS23	4.918	4.871	4.848	4.857	4.894	4.852	0.315	-0.042
O75496	GMNN	-1.313	-1.324	-1.490	-1.108	-1.318	-1.299	0.935	0.020
Q04446	GBE1	4.078	3.937	3.894	3.870	4.007	3.882	0.321	-0.125
O00488	ZNF593	-0.218	-0.189	-0.104	-0.128	-0.204	-0.116	0.045	0.088
Q9H7Z3	NRDE2	0.178	0.209	0.173	0.201	0.193	0.187	0.797	-0.006

P10301	RRAS	1.900	1.799	2.034	2.008	1.849	2.021	0.164	0.172
P23528	CFL1	6.329	6.298	6.314	6.281	6.314	6.298	0.555	-0.016
Q9H3L0	MMADHC	-3.457	-3.527	-3.681	-3.556	-3.492	-3.619	0.252	-0.127
Q13155	AIMP2	0.739	0.627	0.612	0.585	0.683	0.598	0.360	-0.085
Q5VWJ9	SNX30	-0.573	-0.593	-0.646	-0.653	-0.583	-0.649	0.068	-0.066
Q8WVS4	DYNC2I1	-2.515	-2.618	-2.784	-2.775	-2.567	-2.779	0.148	-0.213
O75064	DENND4B	-2.879	-2.843	-2.882	-2.905	-2.861	-2.893	0.289	-0.033
P48382	RFX5	-2.466	-2.487	-2.403	-2.284	-2.476	-2.344	0.261	0.133
Q15649	ZNHIT3	-1.829	-1.915	-1.861	-1.848	-1.872	-1.854	0.751	0.018
P08134	RHOC	2.498	2.441	2.433	2.397	2.469	2.415	0.272	-0.055
Q53FT3	HIKESHI	1.621	1.670	1.476	1.438	1.646	1.457	0.030	-0.189
Q9ULI4	KIF26A	-2.169	-1.742	-1.955	-1.854	-1.956	-1.904	0.850	0.051
Q8N884	CGAS	0.397	0.304	0.386	0.294	0.350	0.340	0.890	-0.010
Q9Y617	PSAT1	3.130	3.160	3.148	3.114	3.145	3.131	0.599	-0.014
P13995	MTHFD2	0.690	0.716	0.813	0.836	0.703	0.825	0.021	0.121
Q969J3	BORCS5	-0.586	-0.635	-0.650	-0.620	-0.610	-0.635	0.504	-0.024
Q8TAE8	GADD45GIP1	2.671	2.620	2.693	2.635	2.645	2.664	0.675	0.019
Q2NL82	TSR1	3.701	3.645	3.639	3.638	3.673	3.639	0.433	-0.035
P36405	ARL3	1.844	1.750	1.630	1.605	1.797	1.618	0.143	-0.180
Q9Y3X0	CCDC9	1.851	1.814	1.854	1.815	1.832	1.834	0.949	0.002
P28676	GCA	-1.358	-0.695	-1.013	-1.263	-1.026	-1.138	0.796	-0.112
Q9UHR4	BAIAP2L1	2.915	3.109	3.038	2.913	3.012	2.975	0.784	-0.037
Q9BTE7	DCUN1D5	1.832	1.757	1.817	1.812	1.795	1.815	0.685	0.020
P09668	CTSH	1.326	1.632	1.583	1.707	1.479	1.645	0.464	0.166
Q8N511	TMEM199	-2.085	-2.113	-2.234	-2.257	-2.099	-2.246	0.017	-0.147
Q15056	EIF4H	-1.351	-1.342	-1.575	-1.592	-1.347	-1.583	0.007	-0.237
Q5T1C6	THEM4	0.747	0.722	0.801	0.753	0.734	0.777	0.286	0.043
O00139	KIF2A	-5.120	-5.177	-5.234	-5.175	-5.149	-5.204	0.307	-0.056
Q9BVG4	PBDC1	4.354	4.249	4.346	4.268	4.302	4.307	0.945	0.005
Q14563	SEMA3A	1.260	1.139	0.780	0.865	1.199	0.823	0.046	-0.377
Q8TB96	ITFG1	-0.491	-0.552	-0.381	-0.514	-0.522	-0.448	0.453	0.074
Q4G0S4	CYP27C1	-2.773	-2.919	-3.141	-3.216	-2.846	-3.178	0.088	-0.332
Q9H211	CDT1	-2.962	-2.805	-3.057	-2.984	-2.883	-3.021	0.302	-0.137
Q14657	LAGE3	-0.337	-0.202	-0.591	-0.225	-0.269	-0.408	0.586	-0.138
Q8TB36	GDAP1	-2.289	-1.927	-2.152	-2.173	-2.108	-2.162	0.814	-0.054

Q96EY1	DNAJA3	-2.442	-2.414	-2.337	-2.371	-2.428	-2.354	0.084	0.074
P52895	AKR1C2	-4.337	-4.351	-3.308	-4.483	-4.344	-3.896	0.585	0.448
Q9H5V7	IKZF5	-0.954	-1.012	-0.931	-0.887	-0.983	-0.909	0.191	0.073
Q14254	FLOT2	3.355	3.284	3.306	3.270	3.319	3.288	0.536	-0.032
P37198	NUP62	2.841	2.876	2.862	2.861	2.858	2.862	0.872	0.004
Q13685	AAMP	1.621	1.670	1.694	1.644	1.646	1.669	0.569	0.024
Q5BJF2	TMEM97	0.759	1.011	0.735	0.565	0.885	0.650	0.279	-0.234
O95848	NUDT14	-0.697	-0.683	-0.418	-0.630	-0.690	-0.524	0.360	0.167
Q96CN9	GCC1	1.121	1.079	1.039	1.120	1.100	1.079	0.709	-0.020
Q96AT1	KIAA1143	1.171	1.116	1.072	1.040	1.143	1.056	0.141	-0.087
O75864	PPP1R37	-0.475	-0.497	-0.575	-0.549	-0.486	-0.562	0.049	-0.076
Q9HB21	PLEKHA1	-3.595	-3.290	-3.345	-3.229	-3.443	-3.287	0.485	0.156
Q16204	CCDC6	3.099	3.109	3.101	3.059	3.104	3.080	0.449	-0.024
O00189	AP4M1	-0.781	-0.681	-0.845	-0.739	-0.731	-0.792	0.489	-0.061
P57735	RAB25	-1.390	-1.044	-1.143	-1.027	-1.217	-1.085	0.582	0.132
P61129	ZC3H6	-1.973	-2.020	-1.559	-1.563	-1.996	-1.561	0.033	0.436
Q9Y666	SLC12A7	0.551	0.520	0.649	0.610	0.536	0.629	0.068	0.094
P62699	YPEL5	-0.803	-1.100	-0.970	-0.987	-0.951	-0.979	0.884	-0.027
P49736	MCM2	5.569	5.510	5.412	5.384	5.540	5.398	0.085	-0.142
Q9BT22	ALG1	-3.124	-2.828	-2.914	-2.884	-2.976	-2.899	0.693	0.077
Q9UBI9	HECA	-4.161	-4.242	-4.064	-4.182	-4.201	-4.123	0.399	0.078
Q9UDR5	AASS	-2.664	-2.744	-2.610	-2.565	-2.704	-2.588	0.158	0.116
Q9ULG1	INO80	0.388	0.386	0.394	0.370	0.387	0.382	0.732	-0.005
P62328	TMSB4X	3.445	3.312	3.056	3.018	3.379	3.037	0.101	-0.342
Q99942	RNF5	-1.052	-1.429	-1.026	-1.395	-1.241	-1.210	0.919	0.030
Q5VIR6	VPS53	0.423	0.451	0.295	0.376	0.437	0.335	0.212	-0.102
O94829	IPO13	-0.223	-0.290	-0.298	-0.294	-0.257	-0.296	0.445	-0.039
Q7L7X3	TAOK1	-0.042	0.028	-0.001	0.048	-0.007	0.024	0.550	0.031
O60890	OPHN1	-1.143	-1.237	-1.312	-1.267	-1.190	-1.289	0.243	-0.099
Q68DQ2	CRYBG3	-1.768	-1.713	-1.588	-1.537	-1.740	-1.563	0.043	0.178
Q14050	COL9A3	-4.408	-3.997	-4.105	-3.911	-4.203	-4.008	0.512	0.194
O75380	NDUFS6	2.028	1.986	2.071	2.087	2.007	2.079	0.145	0.072
O43324	EEF1E1	0.821	0.657	0.694	0.777	0.739	0.736	0.978	-0.003
Q8NEZ5	FBXO22	-1.593	-1.707	-1.814	-1.738	-1.650	-1.776	0.226	-0.126
P06702	S100A9	-2.765	-2.715	-2.600	-2.487	-2.740	-2.543	0.137	0.197

P30837	ALDH1B1	3.965	3.863	4.094	4.031	3.914	4.062	0.158	0.148
Q00169	PITPNA	2.054	1.994	2.004	1.947	2.024	1.975	0.360	-0.049
O95249	GOSR1	-3.065	-2.952	-2.791	-2.947	-3.008	-2.869	0.297	0.139
P15954	COX7C	-0.657	-0.873	-0.714	-0.889	-0.765	-0.801	0.822	-0.036
P02656	APOC3	-3.731	-3.725	-3.291	-3.234	-3.728	-3.262	0.037	0.465
Q9BV86	NTMT1	0.657	0.529	0.678	0.502	0.593	0.590	0.978	-0.003
Q9UNP9	PPIE	-0.661	-0.815	-0.689	-0.711	-0.738	-0.700	0.712	0.038
O60220	TIMM8A	0.130	0.044	0.167	0.149	0.087	0.158	0.334	0.071
O15294	OGT	-2.249	-2.250	-2.352	-2.337	-2.249	-2.344	0.048	-0.095
P49902	NT5C2	-0.383	-0.291	-0.431	-0.409	-0.337	-0.420	0.311	-0.083
P30414	NKTR	0.430	0.308	0.413	0.386	0.369	0.400	0.702	0.031
Q9H5Q4	TFB2M	2.131	2.132	2.141	2.100	2.131	2.121	0.706	-0.010
Q9Y6C9	MTCH2	2.689	2.570	2.688	2.651	2.629	2.670	0.618	0.040
O00151	PDLIM1	1.830	1.954	2.141	2.038	1.892	2.089	0.137	0.198
P43686	PSMC4	0.094	-0.002	0.094	0.050	0.046	0.072	0.693	0.026
Q9Y2S6	TMA7	0.418	0.351	0.487	0.348	0.384	0.418	0.720	0.034
P37059	HSD17B2	-3.543	-3.390	-3.606	-3.228	-3.466	-3.417	0.840	0.050
Q92887	ABCC2	-1.271	-1.331	-1.659	-1.621	-1.301	-1.640	0.018	-0.339
Q14320	FAM50A	2.374	2.310	2.515	2.461	2.342	2.488	0.076	0.146
P51808	DYNLT3	-2.886	-3.051	-3.003	-3.138	-2.969	-3.070	0.442	-0.102
Q9Y5N6	ORC6	-0.646	-0.735	-0.925	-1.024	-0.690	-0.974	0.051	-0.284
Q14204	DYNC1H1	8.075	8.033	8.091	8.055	8.054	8.073	0.567	0.019
Q01995	TAGLN	2.234	2.090	2.362	2.276	2.162	2.319	0.230	0.157
O75152	ZC3H11A	2.373	2.305	2.495	2.435	2.339	2.465	0.110	0.126
P16455	MGMT	2.843	2.704	2.727	2.658	2.774	2.692	0.437	-0.081
Q9P2F8	SIPA1L2	-3.634	-3.699	-3.845	-3.711	-3.666	-3.778	0.315	-0.112
O95881	TXNDC12	3.101	2.908	3.174	3.008	3.005	3.091	0.568	0.086
Q6P4A7	SFXN4	-2.933	-3.067	-2.727	-2.649	-3.000	-2.688	0.081	0.312
Q92520	FAM3C	2.263	2.283	2.367	2.249	2.273	2.308	0.658	0.035
Q9BUF5	TUBB6	3.312	3.194	3.287	3.223	3.253	3.255	0.982	0.002
Q8TCD5	NT5C	1.155	1.530	1.448	1.449	1.342	1.449	0.671	0.106
O14656	TOR1A	0.965	0.999	0.985	0.989	0.982	0.987	0.827	0.005
Q8TAP9	MPLKIP	-2.862	-2.991	-2.831	-2.910	-2.927	-2.870	0.548	0.056
Q5T200	ZC3H13	-1.456	-1.640	-1.605	-1.605	-1.548	-1.605	0.649	-0.057
O95674	CDS2	-0.818	-0.762	-0.796	-0.658	-0.790	-0.727	0.525	0.063

O60832	DKC1	1.689	1.617	1.853	1.772	1.653	1.812	0.100	0.159
Q6IC98	GRAMD4	-1.661	-1.544	-1.475	-1.456	-1.603	-1.466	0.249	0.137
Q92673	SORL1	-2.840	-3.010	-2.893	-2.980	-2.925	-2.936	0.922	-0.011
P53041	PPP5C	3.839	3.856	3.807	3.810	3.848	3.808	0.125	-0.039
P29144	TPP2	4.146	4.194	4.128	4.152	4.170	4.140	0.416	-0.030
P18283	GPX2	-0.644	-0.143	-0.628	-0.576	-0.394	-0.602	0.558	-0.208
Q8N5I2	ARRDC1	-0.521	-0.626	-0.647	-0.629	-0.574	-0.638	0.429	-0.065
Q8NCF5	NFATC2IP	0.420	0.401	0.348	0.304	0.411	0.326	0.126	-0.085
P12236	SLC25A6	2.325	2.480	2.471	2.734	2.403	2.603	0.345	0.200
P04183	TK1	2.098	2.104	1.842	1.926	2.101	1.884	0.121	-0.217
P48730	CSNK1D	-4.530	-4.606	-4.432	-4.749	-4.568	-4.590	0.912	-0.022
Q6SPF0	SAMD1	0.117	0.134	0.119	0.106	0.125	0.112	0.359	-0.013
P20042	EIF2S2	5.282	5.252	5.291	5.218	5.267	5.255	0.795	-0.012
O43491	EPB41L2	0.931	0.792	0.519	0.534	0.862	0.527	0.127	-0.335
Q6Y2X3	DNAJC14	-3.035	-2.955	-2.911	-2.859	-2.995	-2.885	0.167	0.110
P27707	DCK	1.997	2.048	2.084	2.015	2.022	2.049	0.599	0.027
Q9ULW0	TPX2	-2.173	-2.101	-2.045	-2.158	-2.137	-2.101	0.654	0.036
O43264	ZW10	0.391	0.376	0.305	0.366	0.384	0.336	0.352	-0.048
Q6PKG0	LARP1	1.085	1.056	1.071	1.078	1.071	1.075	0.829	0.004
O60518	RANBP6	0.341	0.292	0.340	0.339	0.316	0.340	0.516	0.023
Q01850	CDR2	-3.462	-3.541	-3.674	-3.601	-3.501	-3.637	0.129	-0.136
P13667	PDIA4	6.527	6.453	6.421	6.398	6.490	6.410	0.253	-0.080
O60244	MED14	1.593	1.561	1.563	1.576	1.577	1.569	0.713	-0.008
Q96S82	UBL7	-0.419	-0.350	-0.530	-0.406	-0.384	-0.468	0.385	-0.084
Q9NV96	TMEM30A	-3.827	-3.838	-3.746	-3.738	-3.833	-3.742	0.007	0.090
P52597	HNRNPF	4.645	4.625	4.743	4.702	4.635	4.722	0.102	0.087
Q96RS0	TGS1	1.012	0.977	0.995	0.998	0.994	0.997	0.917	0.002
Q53H80	AKIRIN2	-3.104	-3.272	-3.223	-3.249	-3.188	-3.236	0.671	-0.048
P31260	HOXA10	-5.221	-5.006	-5.065	-4.963	-5.113	-5.014	0.522	0.099
Q9BVM2	DPCD	1.434	1.358	1.199	1.191	1.396	1.195	0.115	-0.201
Q9NZD2	GLTP	-0.348	-0.388	-0.312	-0.378	-0.368	-0.345	0.616	0.023
Q6P1M0	SLC27A4	-0.362	-0.375	-0.231	-0.142	-0.368	-0.187	0.145	0.182
O95819	MAP4K4	-3.551	-3.414	-3.400	-3.469	-3.482	-3.434	0.614	0.048
P17174	GOT1	1.223	1.376	1.450	1.542	1.299	1.496	0.187	0.196
Q9H9B4	SFXN1	3.286	3.264	3.282	3.285	3.275	3.284	0.588	0.008

Q07021	C1QBP	5.417	5.419	5.321	5.413	5.418	5.367	0.466	-0.051
Q8WVX9	FAR1	1.255	1.096	1.238	1.160	1.175	1.199	0.824	0.023
Q9UK59	DBR1	1.616	1.641	1.540	1.575	1.629	1.558	0.092	-0.071
Q9Y4K4	MAP4K5	0.752	0.816	0.645	0.636	0.784	0.641	0.137	-0.143
Q6NXT1	ANKRD54	-2.990	-3.065	-2.888	-2.765	-3.028	-2.827	0.131	0.201
Q8IV50	LYSMD2	-1.881	-1.880	-1.848	-1.775	-1.880	-1.812	0.311	0.069
Q3T906	GNPTAB	-1.351	-1.325	-1.340	-1.280	-1.338	-1.310	0.518	0.028
Q14191	WRN	1.053	1.080	1.134	1.138	1.067	1.136	0.117	0.069
Q53FA7	TP53I3	2.217	2.135	2.135	2.157	2.176	2.146	0.594	-0.030
Q96JJ7	TMX3	2.202	2.159	2.092	2.120	2.181	2.106	0.119	-0.074
Q13509	TUBB3	0.602	0.528	0.629	0.673	0.565	0.651	0.213	0.086
Q6P161	MRPL54	1.662	1.502	1.531	1.480	1.582	1.506	0.507	-0.077
P13747	HLA	-0.824	-0.836	-0.527	-0.580	-0.830	-0.553	0.051	0.276
Q9H300	PARL	-2.351	-2.292	-2.121	-2.043	-2.322	-2.082	0.045	0.240
Q05D32	CTDSPL2	-3.754	-3.750	-3.871	-3.899	-3.752	-3.885	0.062	-0.133
P17020	ZNF16	-2.608	-2.524	-2.648	-2.436	-2.566	-2.542	0.861	0.024
Q15075	EEA1	5.387	5.296	5.416	5.407	5.341	5.412	0.363	0.071
Q8NDX6	ZNF740	-1.619	-1.530	-1.576	-1.434	-1.574	-1.505	0.507	0.069
Q13526	PIN1	3.429	3.426	3.349	3.319	3.427	3.334	0.096	-0.094
Q9HAC8	UBTD1	-0.330	-0.229	-0.403	-0.273	-0.280	-0.338	0.553	-0.058
P48552	NRIP1	-4.662	-4.396	-4.951	-4.752	-4.529	-4.851	0.202	-0.322
Q02487	DSC2	-3.993	-3.369	-3.618	-3.601	-3.681	-3.610	0.857	0.071
Q96GQ5	RUSF1	0.149	0.061	0.131	0.144	0.105	0.138	0.594	0.032
Q9H477	RBKS	-7.308	-6.911	-7.869	-7.429	-7.110	-7.649	0.212	-0.540
P61587	RND3	-2.862	-2.794	-3.006	-3.003	-2.828	-3.005	0.121	-0.177
Q99460	PSMD1	1.192	1.275	1.086	1.238	1.234	1.162	0.516	-0.072
P40429	RPL13A	4.990	4.838	4.873	4.840	4.914	4.857	0.588	-0.057
Q13637	RAB32	1.800	1.692	1.821	1.772	1.746	1.796	0.516	0.050
Q8IYR2	SMYD4	-1.539	-1.470	-1.589	-1.497	-1.505	-1.543	0.578	-0.039
O95478	NSA2	2.758	2.727	2.865	2.853	2.743	2.859	0.057	0.116
Q96LD8	SENP8	-2.240	-2.056	-2.125	-2.131	-2.148	-2.128	0.862	0.020
Q9H777	ELAC1	-1.467	-1.444	-1.476	-1.458	-1.455	-1.467	0.506	-0.012
Q99442	SEC62	1.751	1.663	1.856	1.763	1.707	1.809	0.249	0.103
P07951	TPM2	1.481	1.469	1.201	1.167	1.475	1.184	0.021	-0.291
Q96EN8	MOCOS	-4.120	-3.931	-3.921	-3.677	-4.026	-3.799	0.286	0.227

Q9Y487	ATP6V0A2	-0.446	-0.433	-0.385	-0.297	-0.440	-0.341	0.262	0.098
Q9H082	RAB33B	-1.638	-1.809	-1.751	-1.681	-1.724	-1.716	0.945	0.008
Q9BS31	ZNF649	-2.760	-2.842	-2.822	-2.920	-2.801	-2.871	0.395	-0.069
Q14457	BECN1	1.143	1.110	1.139	1.153	1.127	1.146	0.431	0.020
P01116	KRAS	-2.141	-0.962	-0.538	-1.345	-1.551	-0.942	0.493	0.610
Q16181	SEPTIN7	-1.233	-1.215	-1.399	-1.122	-1.224	-1.260	0.836	-0.036
Q5TBB1	RNASEH2B	-0.060	-0.077	-0.035	-0.069	-0.069	-0.052	0.495	0.017
Q08379	GOLGA2	-0.157	-0.129	-0.024	-0.093	-0.143	-0.058	0.215	0.084
Q9NXV6	CDKN2AIP	1.811	1.751	1.825	1.806	1.781	1.815	0.452	0.034
P46782	RPS5	4.628	4.598	4.597	4.642	4.613	4.619	0.840	0.006
O00622	CCN1	1.573	1.487	0.982	1.045	1.530	1.013	0.014	-0.517
Q9H9Y2	RPF1	1.048	0.926	1.090	1.043	0.987	1.066	0.404	0.079
Q9UHJ6	SHPK	-0.156	-0.389	-0.295	-0.388	-0.272	-0.342	0.660	-0.069
Q99633	PRPF18	-1.149	-1.161	-1.248	-1.165	-1.155	-1.206	0.429	-0.051
O75312	ZPR1	3.195	3.187	3.216	3.187	3.191	3.201	0.606	0.010
Q9Y6X5	ENPP4	-1.476	-1.012	-1.465	-1.243	-1.244	-1.354	0.726	-0.109
Q96QK1	VPS35	4.935	4.863	4.807	4.799	4.899	4.803	0.225	-0.096
Q9NWV8	BABAM1	1.086	1.234	1.101	1.192	1.160	1.146	0.894	-0.013
Q15427	SF3B4	0.170	-0.025	0.214	0.110	0.072	0.162	0.524	0.090
P42695	NCAPD3	0.258	0.239	-0.016	0.092	0.248	0.038	0.151	-0.210
P01008	SERPINC1	0.506	0.627	0.701	0.696	0.566	0.699	0.272	0.132
O75113	N4BP1	1.431	1.336	1.394	1.357	1.384	1.376	0.897	-0.008
Q9BZD4	NUF2	1.205	1.150	0.864	0.932	1.178	0.898	0.026	-0.280
Q5W0U4	BSPRY	-2.429	-1.954	-2.263	-2.174	-2.192	-2.218	0.929	-0.027
Q96MD2	KICS2	-1.973	-2.095	-1.968	-2.077	-2.034	-2.023	0.904	0.011
Q9NSD9	FARSB	4.540	4.476	4.510	4.507	4.508	4.509	0.981	0.001
Q969E8	TSR2	-0.675	-0.660	-0.610	-0.715	-0.668	-0.662	0.939	0.005
O94768	STK17B	-2.529	-2.270	-2.652	-2.198	-2.400	-2.425	0.934	-0.025
P11498	PC	2.113	2.085	2.098	2.076	2.099	2.087	0.581	-0.012
Q8WUH6	TMEM263	-1.855	-1.787	-2.033	-1.942	-1.821	-1.987	0.109	-0.166
Q9Y3Q3	TMED3	-3.192	-3.163	-3.086	-3.103	-3.178	-3.094	0.055	0.083
Q8N488	RYBP	-1.879	-1.910	-1.965	-1.900	-1.894	-1.933	0.436	-0.038
Q9NRY4	ARHGAP35	2.648	2.675	2.579	2.617	2.662	2.598	0.122	-0.064
Q6UWW8	CES3	-2.708	-2.819	-2.756	-2.648	-2.763	-2.702	0.509	0.062
Q86V81	ALYREF	4.464	4.448	4.420	4.383	4.456	4.402	0.170	-0.054

Q00839	HNRNPU	2.866	2.820	2.935	2.851	2.843	2.893	0.437	0.049
P62745	RHOB	-1.711	-1.496	-1.619	-1.398	-1.603	-1.509	0.603	0.094
Q9Y6W5	WASF2	0.657	0.623	0.600	0.684	0.640	0.642	0.976	0.002
Q8IZ13	ZBED8	-1.353	-1.343	-1.564	-1.397	-1.348	-1.481	0.355	-0.133
Q92572	AP3S1	1.843	1.812	1.800	1.731	1.827	1.766	0.292	-0.062
Q9Y224	RTRAF	3.893	3.816	3.820	3.779	3.854	3.800	0.371	-0.055
Q9H2D1	SLC25A32	-1.636	-1.571	-1.711	-1.704	-1.603	-1.708	0.189	-0.104
P61769	B2M	0.554	0.564	0.995	0.800	0.559	0.898	0.178	0.339
Q15334	LLGL1	1.854	1.873	1.794	1.772	1.864	1.783	0.032	-0.081
Q9BX63	BRIP1	-3.613	-3.626	-3.956	-4.012	-3.619	-3.984	0.038	-0.364
Q9H6R0	DHX33	0.710	0.653	0.776	0.848	0.682	0.812	0.113	0.130
O75127	PTCD1	0.769	0.850	0.699	0.859	0.810	0.779	0.774	-0.031
P49589	CARS1	-0.232	-0.401	-0.211	-0.170	-0.316	-0.190	0.365	0.126
P04731	MT1A	-3.491	-3.654	-3.689	-3.740	-3.573	-3.715	0.313	-0.142
Q13547	HDAC1	3.575	3.616	3.446	3.405	3.595	3.425	0.028	-0.170
P03915	MT	-0.303	-0.339	-0.384	-0.290	-0.321	-0.337	0.790	-0.016
Q8NFC6	BOD1L1	2.575	2.561	2.652	2.643	2.568	2.647	0.018	0.079
Q9H270	VPS11	2.127	2.050	2.103	2.076	2.088	2.090	0.978	0.001
P07203	GPX1	3.040	3.121	3.164	3.065	3.080	3.115	0.646	0.034
Q5VV42	CDKAL1	-2.308	-2.438	-2.514	-2.463	-2.373	-2.489	0.302	-0.116
O43482	OIP5	-1.875	-1.883	-2.186	-2.019	-1.879	-2.103	0.228	-0.223
Q5BKZ1	ZNF326	1.862	1.782	1.825	1.826	1.822	1.826	0.944	0.004
P09525	ANXA4	0.721	0.761	1.017	0.859	0.741	0.938	0.227	0.197
Q5VUB5	FAM171A1	-2.242	-2.388	-2.491	-2.392	-2.315	-2.442	0.305	-0.126
P49005	POLD2	1.813	1.967	1.735	1.944	1.890	1.840	0.739	-0.050
Q92667	AKAP1	-0.810	-0.906	-0.761	-0.787	-0.858	-0.774	0.316	0.084
P05412	JUN	-0.303	-0.437	-0.552	-0.634	-0.370	-0.593	0.130	-0.223
Q86SF2	GALNT7	0.452	0.515	0.396	0.324	0.483	0.360	0.123	-0.124
Q9UKV5	AMFR	-1.546	-1.509	-1.401	-1.293	-1.527	-1.347	0.156	0.180
P10398	ARAF	0.225	0.234	0.175	0.248	0.230	0.212	0.712	-0.018
P18146	EGR1	0.063	0.020	0.135	0.082	0.042	0.108	0.198	0.067
Q5HYI8	RABL3	1.048	1.006	1.211	1.201	1.027	1.206	0.063	0.179
Q9Y672	ALG6	0.518	0.410	0.364	0.401	0.464	0.383	0.357	-0.081
P80188	LCN2	-3.043	-2.889	-2.850	-3.472	-2.966	-3.161	0.643	-0.195
Q9NRG4	SMYD2	-2.075	-2.055	-2.119	-2.123	-2.065	-2.121	0.099	-0.056

P62277	RPS13	5.580	5.619	5.581	5.579	5.599	5.580	0.498	-0.019
Q9Y2Q9	MRPS28	2.003	2.036	1.944	1.911	2.019	1.927	0.059	-0.092
Q8IVL6	P3H3	0.621	0.514	0.128	0.275	0.567	0.201	0.065	-0.366
Q14980	NUMA1	-0.994	-1.049	-0.872	-1.020	-1.022	-0.946	0.485	0.076
Q8N2Z9	CENPS	-3.076	-3.033	-3.250	-3.164	-3.055	-3.207	0.125	-0.152
O43920	NDUFS5	0.797	0.688	0.796	0.856	0.743	0.826	0.340	0.083
Q06830	PRDX1	6.990	6.902	6.841	6.846	6.946	6.843	0.258	-0.102
Q9NX00	TMEM160	-1.575	-1.699	-1.721	-1.525	-1.637	-1.623	0.915	0.014
Q96S59	RANBP9	-1.654	-1.651	-1.726	-1.618	-1.652	-1.672	0.778	-0.019
Q8N543	OGFOD1	0.811	0.598	0.865	0.661	0.704	0.763	0.728	0.059
Q9NVT9	ARMC1	0.468	0.439	0.543	0.502	0.454	0.522	0.123	0.069
Q96MH2	HEXIM2	-1.741	-1.759	-1.806	-1.710	-1.750	-1.758	0.901	-0.008
Q8ND90	PNMA1	-2.211	-2.142	-2.041	-2.038	-2.177	-2.040	0.156	0.137
Q8TB40	ABHD4	-2.970	-3.057	-2.786	-2.842	-3.014	-2.814	0.078	0.200
Q15819	UBE2V2	1.989	1.969	2.043	2.019	1.979	2.031	0.081	0.052
P51948	MNAT1	-3.290	-3.368	-3.241	-3.093	-3.329	-3.167	0.232	0.162
O00217	NDUFS8	2.308	2.226	2.343	2.318	2.267	2.331	0.345	0.064
O75691	UTP20	4.771	4.683	4.837	4.790	4.727	4.813	0.264	0.086
Q8TEA1	NSUN6	0.493	0.480	0.407	0.379	0.487	0.393	0.059	-0.094
Q96H79	ZC3HAV1L	1.569	1.386	1.496	1.411	1.477	1.453	0.842	-0.024
Q8WVJ2	NUDCD2	1.363	1.264	1.350	1.227	1.313	1.289	0.785	-0.025
Q96K37	SLC35E1	-2.472	-2.481	-2.763	-2.598	-2.477	-2.681	0.245	-0.204
P48444	ARCN1	2.585	2.628	2.423	2.432	2.607	2.427	0.065	-0.180
Q9BY89	KIAA1671	1.339	1.352	1.343	1.370	1.346	1.357	0.554	0.011
O15160	POLR1C	-3.300	-3.357	-3.400	-3.431	-3.328	-3.415	0.154	-0.087
O43422	THAP12	-1.788	-1.715	-1.832	-1.788	-1.751	-1.810	0.326	-0.058
P18031	PTPN1	3.352	3.586	3.452	3.472	3.469	3.462	0.963	-0.007
Q9NU19	TBC1D22B	-1.719	-1.652	-1.784	-1.525	-1.685	-1.654	0.851	0.031
P10155	RO60	-1.728	-1.650	-1.784	-1.786	-1.689	-1.785	0.247	-0.096
P41567	EIF1	-0.645	-0.486	-0.595	-0.425	-0.565	-0.510	0.681	0.055
Q02543	RPL18A	5.302	5.287	5.264	5.217	5.294	5.240	0.238	-0.054
Q09666	AHNAK	9.401	9.382	9.340	9.279	9.391	9.309	0.203	-0.082
Q8N5M1	ATPAF2	-0.044	0.128	-0.207	-0.029	0.042	-0.118	0.327	-0.160
P48507	GCLM	0.474	0.634	0.948	0.815	0.554	0.882	0.091	0.328
O14745	SLC9A3R1	2.992	3.006	3.023	2.998	2.999	3.011	0.530	0.011

O00512	BCL9	-1.584	-1.564	-1.685	-1.634	-1.574	-1.659	0.146	-0.085
Q14674	ESPL1	-2.660	-2.703	-2.704	-2.615	-2.682	-2.659	0.711	0.022
Q15018	ABRAXAS2	1.872	1.921	1.863	1.908	1.897	1.885	0.764	-0.011
P61009	SPCS3	1.607	1.702	1.451	1.539	1.655	1.495	0.133	-0.160
Q96MS0	ROBO3	-4.538	-4.799	-4.299	-4.364	-4.669	-4.331	0.219	0.337
Q9HC16	APOBEC3G	-1.120	-1.235	-1.453	-1.356	-1.178	-1.405	0.098	-0.227
Q92830	KAT2A	-1.729	-1.707	-1.626	-1.522	-1.718	-1.574	0.207	0.144
Q5TF39	MFSD4B	-2.177	-2.193	-2.151	-2.224	-2.185	-2.188	0.952	-0.003
Q2M1P5	KIF7	-2.638	-2.568	-2.660	-2.611	-2.603	-2.635	0.538	-0.032
Q712K3	UBE2R2	-0.630	-0.599	-0.344	-0.419	-0.615	-0.381	0.067	0.233
Q9H936	SLC25A22	2.971	2.823	3.070	2.930	2.897	3.000	0.420	0.103
A6NGR9	MROH6	-4.206	-3.828	-4.177	-4.033	-4.017	-4.105	0.725	-0.088
P54577	YARS1	6.333	6.347	6.398	6.352	6.340	6.375	0.351	0.035
Q8TF42	UBASH3B	-1.297	-1.025	-1.161	-0.897	-1.161	-1.029	0.558	0.132
Q9Y6H3	ATP23	-2.139	-2.092	-2.242	-2.147	-2.116	-2.195	0.315	-0.079
Q9C073	FAM117A	-3.947	-3.972	-3.954	-3.772	-3.959	-3.863	0.481	0.096
P12814	ACTN1	-0.955	-0.743	-0.664	-0.847	-0.849	-0.756	0.574	0.094
Q5TA31	RNF187	-3.247	-3.156	-3.133	-2.928	-3.202	-3.030	0.316	0.172
Q9NXV2	KCTD5	-1.852	-1.853	-1.820	-1.848	-1.853	-1.834	0.401	0.019
Q92560	BAP1	-1.029	-1.084	-1.055	-1.021	-1.056	-1.038	0.637	0.018
Q8NHZ8	CDC26	0.428	0.436	0.241	0.283	0.432	0.262	0.070	-0.170
P11233	RALA	2.426	2.317	2.670	2.463	2.371	2.566	0.276	0.195
P22059	OSBP	3.861	3.873	3.946	3.811	3.867	3.879	0.889	0.012
Q9BRJ2	MRPL45	2.659	2.577	2.654	2.644	2.618	2.649	0.585	0.031
P20585	MSH3	2.602	2.563	2.487	2.525	2.583	2.506	0.108	-0.077
A6NJ78	METTL15	-1.038	-0.980	-1.095	-1.090	-1.009	-1.093	0.211	-0.083
Q9H4H8	FAM83D	-2.143	-1.989	-2.317	-2.232	-2.066	-2.274	0.176	-0.208
Q9P2D3	HEATR5B	-2.198	-2.466	-2.581	-2.582	-2.332	-2.581	0.314	-0.249
Q9Y4B4	RAD54L2	-2.010	-2.010	-2.131	-1.911	-2.010	-2.021	0.934	-0.011
P78524	DENND2B	-3.546	-3.704	-3.588	-3.527	-3.625	-3.557	0.543	0.068
P48556	PSMD8	4.537	4.450	4.525	4.429	4.494	4.477	0.823	-0.017
Q13619	CUL4A	-0.217	-0.338	-0.168	-0.347	-0.278	-0.257	0.870	0.020
Q86W50	METTL16	0.606	0.647	0.636	0.523	0.627	0.580	0.552	-0.047
P31749	AKT1	0.032	-0.001	-0.121	-0.149	0.016	-0.135	0.022	-0.151
Q7KZ85	SUPT6H	2.632	2.611	2.586	2.556	2.622	2.571	0.124	-0.051

Q12769	NUP160	2.925	2.871	2.886	2.880	2.898	2.883	0.682	-0.015
Q92889	ERCC4	-0.889	-0.880	-0.884	-0.880	-0.885	-0.882	0.672	0.003
Q9Y6D6	ARFGEF1	2.126	2.220	2.155	2.208	2.173	2.181	0.896	0.008
Q9NVC6	MED17	1.370	1.329	1.332	1.321	1.350	1.326	0.447	-0.023
Q8N8E3	CEP112	-2.060	-1.825	-2.260	-1.865	-1.943	-2.062	0.665	-0.120
Q96QR8	PURB	3.164	3.091	3.228	3.212	3.128	3.220	0.225	0.092
O43676	NDUFB3	1.330	1.198	1.454	1.365	1.264	1.410	0.226	0.146
Q9H4F1	ST6GALNAC4	-3.785	-3.612	-3.856	-3.460	-3.699	-3.658	0.876	0.040
P19823	ITIH2	-1.143	-1.373	-1.195	-1.415	-1.258	-1.305	0.796	-0.047
O95166	GABARAP	-1.806	-1.893	-1.710	-1.755	-1.850	-1.732	0.180	0.118
P46940	IQGAP1	6.990	6.982	7.042	6.961	6.986	7.002	0.766	0.016
E9PRG8	C11orf98	2.394	2.377	2.450	2.510	2.386	2.480	0.173	0.094
Q86VM9	ZC3H18	2.995	2.881	2.953	2.909	2.938	2.931	0.924	-0.007
P48147	PREP	4.451	4.408	4.405	4.395	4.429	4.400	0.401	-0.029
Q92499	DDX1	-2.208	-2.314	-2.246	-2.249	-2.261	-2.248	0.845	0.013
Q9H900	ZWILCH	-1.045	-0.951	-1.283	-1.048	-0.998	-1.165	0.369	-0.168
O60841	EIF5B	5.296	5.222	5.281	5.236	5.259	5.258	0.992	-0.001
O60231	DHX16	3.205	3.177	3.118	3.128	3.191	3.123	0.106	-0.068
O15014	ZNF609	0.128	0.059	0.128	0.036	0.094	0.082	0.857	-0.012
O00221	NFKBIE	-2.096	-2.093	-2.179	-2.014	-2.095	-2.096	0.988	-0.002
Q9BYE7	PCGF6	-2.822	-2.720	-2.755	-2.776	-2.771	-2.766	0.938	0.005
O15061	SYNM	-2.919	-3.074	-3.211	-3.112	-2.997	-3.162	0.236	-0.165
Q9BYD6	MRPL1	2.639	2.581	2.657	2.682	2.610	2.669	0.252	0.059
P61225	RAP2B	4.072	3.958	4.002	3.961	4.015	3.981	0.659	-0.034
Q92945	KHSRP	5.647	5.578	5.588	5.595	5.612	5.591	0.654	-0.021
Q8WU90	ZC3H15	4.009	3.872	3.947	3.847	3.940	3.897	0.665	-0.043
P49459	UBE2A	0.909	0.872	0.959	0.974	0.891	0.966	0.114	0.076
Q8IU81	IRF2BP1	1.696	1.733	1.645	1.695	1.714	1.670	0.295	-0.045
O75449	KATNA1	-0.467	-0.573	-0.505	-0.501	-0.520	-0.503	0.803	0.017
Q96FI4	NEIL1	-3.509	-3.392	-3.558	-3.406	-3.450	-3.482	0.776	-0.031
Q12789	GTF3C1	-4.577	-4.496	-4.612	-4.519	-4.537	-4.565	0.686	-0.029
Q96KP4	CNDP2	1.846	1.986	1.983	1.976	1.916	1.980	0.530	0.064
Q16763	UBE2S	0.017	0.026	0.353	0.363	0.021	0.358	0.000	0.337
P26572	MGAT1	-0.441	-0.420	-0.459	-0.383	-0.430	-0.421	0.845	0.009
P0C7U0	ELFN1	-2.021	-1.878	-1.937	-1.742	-1.950	-1.840	0.467	0.110

Q8TDB6	DTX3L	1.400	1.341	1.839	1.793	1.370	1.816	0.009	0.445
O14561	NDUFAB1	1.313	1.376	1.393	1.289	1.345	1.341	0.963	-0.003
Q13287	NMI	1.081	0.956	1.530	1.442	1.019	1.486	0.033	0.467
P31321	PRKAR1B	-1.741	-1.726	-2.090	-2.046	-1.733	-2.068	0.024	-0.335
Q9HD45	TM9SF3	2.272	2.353	2.253	2.236	2.312	2.244	0.329	-0.068
O94898	LRIG2	-3.916	-3.952	-4.110	-3.891	-3.934	-4.000	0.652	-0.066
Q86TX2	ACOT1	-3.887	-3.702	-3.667	-3.624	-3.795	-3.646	0.344	0.149
P62879	GNB2	0.870	0.847	0.784	0.819	0.858	0.802	0.136	-0.057
Q8TEQ6	GEMIN5	4.729	4.671	4.558	4.549	4.700	4.554	0.118	-0.146
Q9UHA3	RSL24D1	-0.251	-0.116	-0.087	-0.037	-0.183	-0.062	0.300	0.121
P16401	H1	6.888	6.685	6.942	6.741	6.786	6.842	0.735	0.055
P39023	RPL3	6.182	6.114	6.197	6.165	6.148	6.181	0.499	0.033
O43865	AHCYL1	2.327	2.305	2.229	2.207	2.316	2.218	0.024	-0.098
Q6NUK4	REEP3	-3.604	-3.163	-3.041	-3.374	-3.383	-3.207	0.594	0.176
O15305	PMM2	2.414	2.482	2.573	2.544	2.448	2.558	0.151	0.110
Q6GMV3	PTRHD1	1.488	1.353	1.463	1.467	1.420	1.465	0.628	0.045
Q00535	CDK5	-1.848	-1.671	-2.025	-1.891	-1.760	-1.958	0.226	-0.198
Q9Y657	SPIN1	0.788	0.809	0.808	0.834	0.798	0.821	0.312	0.023
Q9H1D9	POLR3F	0.780	0.896	0.812	0.926	0.838	0.869	0.738	0.031
Q96DY7	MTBP	-2.678	-2.557	-2.849	-2.552	-2.618	-2.700	0.678	-0.083
Q9P2S5	WRAP73	-2.298	-2.219	-2.326	-2.229	-2.259	-2.277	0.793	-0.019
Q8WWQ0	PHIP	2.309	2.259	2.273	2.258	2.284	2.266	0.592	-0.018
Q765P7	MTSS2	-2.199	-2.248	-1.956	-1.985	-2.224	-1.970	0.022	0.253
Q9BSE5	AGMAT	-2.121	-1.694	-2.183	-1.910	-1.907	-2.046	0.646	-0.139
Q9Y6A9	SPCS1	0.188	0.180	0.118	-0.005	0.184	0.056	0.286	-0.128
Q92797	SYMPK	3.402	3.443	3.415	3.443	3.422	3.429	0.825	0.006
P06703	S100A6	4.740	4.722	4.135	4.115	4.731	4.125	0.001	-0.606
Q9UJX3	ANAPC7	-2.928	-2.967	-2.797	-2.876	-2.947	-2.837	0.174	0.111
P17275	JUNB	2.209	2.103	2.566	2.470	2.156	2.518	0.038	0.362
P61927	RPL37	2.464	2.346	2.494	2.398	2.405	2.446	0.644	0.041
Q9Y4K1	CRYBG1	-1.006	-0.655	-0.737	-0.944	-0.831	-0.841	0.966	-0.010
P49419	ALDH7A1	2.165	2.195	2.226	2.334	2.180	2.280	0.300	0.100
O43301	HSPA12A	-0.262	-0.238	-0.352	-0.270	-0.250	-0.311	0.363	-0.061
Q96C57	CUSTOS	1.466	1.394	1.383	1.407	1.430	1.395	0.500	-0.035
Q969G5	CAVIN3	0.324	0.508	0.586	0.389	0.416	0.488	0.647	0.072

Q9BWU0	SLC4A1AP	1.997	2.133	1.880	1.891	2.065	1.886	0.228	-0.179
P11172	UMPS	3.382	3.372	3.317	3.334	3.377	3.325	0.054	-0.051
Q0PNE2	ELP6	-0.883	-0.976	-0.979	-0.989	-0.929	-0.984	0.448	-0.055
Q6PI48	DARS2	4.457	4.364	4.582	4.516	4.411	4.549	0.148	0.139
Q9UJZ1	STOML2	1.094	1.014	1.295	1.304	1.054	1.299	0.099	0.245
Q86TC9	MYPN	-0.995	-1.259	-1.291	-1.434	-1.127	-1.363	0.292	-0.236
O15239	NDUFA1	-2.391	-2.533	-2.114	-2.194	-2.462	-2.154	0.091	0.307
Q9BPX3	NCAPG	3.134	3.132	2.934	2.941	3.133	2.938	0.004	-0.196
Q96K80	ZC3H10	-5.285	-5.055	-5.052	-5.196	-5.170	-5.124	0.773	0.046
Q9UKT7	FBXL3	-4.592	-4.377	-4.548	-4.526	-4.484	-4.537	0.712	-0.052
P02649	APOE	0.160	0.125	0.332	0.303	0.143	0.318	0.018	0.175
Q9Y265	RUVBL1	3.386	3.378	3.278	3.223	3.382	3.250	0.125	-0.131
O95857	TSPAN13	-4.075	-4.228	-3.968	-4.193	-4.152	-4.081	0.659	0.071
Q9NWV4	CZIB	3.048	3.067	3.044	3.089	3.057	3.067	0.753	0.009
Q9H999	PANK3	-1.834	-2.001	-1.856	-1.833	-1.917	-1.845	0.544	0.073
Q96C36	PYCR2	3.108	3.114	3.138	3.141	3.111	3.139	0.020	0.028
P0DI83	RAB34	-2.006	-2.083	-1.882	-1.934	-2.044	-1.908	0.115	0.136
P63092	GNAS	-2.427	-2.411	-2.490	-2.411	-2.419	-2.451	0.569	-0.031
P60983	GMFB	2.050	2.032	1.904	2.012	2.041	1.958	0.360	-0.083
P12694	BCKDHA	-1.155	-1.100	-1.160	-1.106	-1.127	-1.133	0.897	-0.006
Q01415	GALK2	-4.004	-4.042	-3.925	-3.922	-4.023	-3.924	0.119	0.099
Q8TF01	PNISR	1.210	1.036	1.123	1.082	1.123	1.102	0.853	-0.021
Q9UEE9	CFDP1	1.248	0.914	1.103	0.858	1.081	0.981	0.679	-0.100
Q8IYS1	PM20D2	1.567	1.465	1.691	1.669	1.516	1.680	0.179	0.164
Q86Y39	NDUFA11	0.675	0.482	0.602	0.729	0.579	0.666	0.541	0.087
Q9H1B7	IRF2BPL	0.698	0.678	0.712	0.692	0.688	0.702	0.406	0.014
Q9P2M4	TBC1D14	-4.295	-4.302	-4.011	-4.048	-4.299	-4.029	0.038	0.269
Q9UL42	PNMA2	0.430	0.336	0.043	0.128	0.383	0.085	0.044	-0.298
Q99805	TM9SF2	2.065	2.108	2.082	2.027	2.087	2.054	0.459	-0.032
Q9NQT8	KIF13B	1.184	1.385	1.231	1.263	1.285	1.247	0.773	-0.037
P30043	BLVRB	4.048	4.090	4.140	4.146	4.069	4.143	0.170	0.074
Q15269	PWP2	4.049	3.924	4.065	4.017	3.987	4.041	0.537	0.055
P13489	RNH1	4.878	4.792	4.896	4.917	4.835	4.907	0.332	0.072
P18887	XRCC1	2.972	3.001	2.942	2.950	2.987	2.946	0.207	-0.041
Q9BYD3	MRPL4	-1.006	-1.071	-0.996	-1.037	-1.039	-1.017	0.640	0.022

P42696	RBM34	2.032	1.968	1.968	2.014	2.000	1.991	0.847	-0.009
P53611	RABGGTB	1.655	1.602	1.506	1.504	1.629	1.505	0.134	-0.123
Q9Y2G5	POFUT2	-0.186	-0.347	-0.218	-0.256	-0.266	-0.237	0.781	0.029
Q8NDT2	RBM15B	-0.403	-0.386	-0.389	-0.385	-0.394	-0.387	0.537	0.007
Q16832	DDR2	-1.226	-1.247	-0.810	-0.719	-1.237	-0.764	0.049	0.472
Q9H488	POFUT1	2.299	2.349	2.232	2.297	2.324	2.265	0.293	-0.059
Q9C005	DPY30	-2.586	-2.577	-2.328	-2.312	-2.581	-2.320	0.004	0.261
Q9UIM3	FKBPL	-1.980	-2.000	-1.885	-1.860	-1.990	-1.873	0.021	0.117
O00534	VWA5A	-3.064	-3.096	-2.546	-2.635	-3.080	-2.590	0.037	0.490
Q96C19	EFHD2	3.015	2.992	3.055	2.963	3.004	3.009	0.932	0.005
O95396	MOCS3	0.856	0.839	0.809	0.784	0.848	0.797	0.094	-0.051
Q9BYW2	SETD2	-0.570	-0.645	-0.722	-0.691	-0.608	-0.707	0.192	-0.099
P27816	MAP4	0.062	-0.048	0.008	-0.003	0.007	0.002	0.945	-0.005
Q3B726	POLR1F	0.535	0.564	0.552	0.484	0.550	0.518	0.513	-0.032
Q9NQY0	BIN3	-0.519	-0.591	-0.987	-0.840	-0.555	-0.914	0.082	-0.359
P20645	M6PR	1.901	1.812	1.942	1.862	1.857	1.902	0.525	0.046
Q96F10	SAT2	-2.826	-3.061	-2.766	-2.628	-2.944	-2.697	0.240	0.247
P08670	VIM	7.436	7.288	7.360	7.351	7.362	7.356	0.947	-0.006
Q9NPR9	GPR108	-1.879	-2.081	-1.965	-2.092	-1.980	-2.029	0.730	-0.049
Q9Y3D3	MRPS16	-0.643	-0.259	-0.516	-0.322	-0.451	-0.419	0.899	0.032
P12277	CKB	0.477	0.935	0.659	0.615	0.706	0.637	0.813	-0.069
P04843	RPN1	5.463	5.397	5.434	5.385	5.430	5.410	0.674	-0.020
Q9H4Z3	PCIF1	0.528	0.568	0.518	0.532	0.548	0.525	0.449	-0.023
Q9NRP0	OSTC	-0.053	-0.027	-0.296	0.038	-0.040	-0.129	0.689	-0.089
P27694	RPA1	4.950	4.890	4.740	4.698	4.920	4.719	0.041	-0.201
Q9BUQ8	DDX23	4.496	4.465	4.565	4.503	4.481	4.534	0.302	0.053
Q9Y6B6	SAR1B	1.683	1.660	1.610	1.516	1.672	1.563	0.244	-0.109
Q8N2H4	SYS1	-2.113	-2.221	-2.121	-2.175	-2.167	-2.148	0.796	0.019
P62829	RPL23	6.506	6.510	6.428	6.485	6.508	6.456	0.321	-0.052
Q96FZ7	CHMP6	0.016	-0.021	0.071	-0.026	-0.003	0.023	0.695	0.025
O76031	CLPX	3.424	3.389	3.380	3.343	3.407	3.362	0.219	-0.045
Q8NCC3	PLA2G15	-1.452	-1.628	-1.664	-1.682	-1.540	-1.673	0.370	-0.133
Q9NTJ5	SACM1L	-0.370	-0.243	-0.287	-0.227	-0.307	-0.257	0.580	0.049
Q9BYB4	GNB1L	-0.652	-0.669	-0.714	-0.761	-0.660	-0.737	0.158	-0.077
Q8NFP9	NBEA	-2.296	-2.359	-2.451	-2.367	-2.327	-2.409	0.272	-0.081

Q8IWL3	HSCB	0.065	-0.025	0.076	0.089	0.020	0.083	0.394	0.062
P34059	GALNS	1.602	1.453	1.542	1.505	1.528	1.524	0.965	-0.004
Q5TEC3	ZNF697	-5.025	-5.134	-4.809	-5.049	-5.080	-4.929	0.412	0.151
Q9BVK6	TMED9	3.845	3.838	3.798	3.814	3.842	3.806	0.097	-0.035
Q92547	TOPBP1	1.526	1.499	1.608	1.432	1.513	1.520	0.948	0.007
P51688	SGSH	-0.356	-0.285	-0.275	-0.176	-0.320	-0.225	0.272	0.095
Q16584	MAP3K11	-4.644	-4.748	-4.862	-4.791	-4.696	-4.827	0.190	-0.131
Q96HR9	REEP6	-1.725	-1.737	-1.701	-1.666	-1.731	-1.683	0.190	0.048
Q9BXB5	OSBPL10	-3.054	-3.235	-2.890	-3.123	-3.145	-3.007	0.453	0.138
Q9UHY1	NRBP1	2.294	2.249	2.155	2.191	2.272	2.173	0.081	-0.099
Q96AQ8	MCUR1	0.893	0.949	0.919	1.042	0.921	0.980	0.501	0.060
Q2TB10	ZNF800	1.777	1.728	1.863	1.815	1.752	1.839	0.126	0.087
P17980	PSMC3	4.665	4.638	4.662	4.617	4.652	4.639	0.697	-0.012
Q16543	CDC37	5.806	5.724	5.698	5.665	5.765	5.681	0.259	-0.084
P40227	CCT6A	1.546	1.557	1.596	1.582	1.552	1.589	0.060	0.037
P08240	SRPRA	-2.037	-2.301	-1.961	-2.294	-2.169	-2.128	0.866	0.041
O94830	DDHD2	-0.036	-0.171	-0.167	-0.177	-0.104	-0.172	0.495	-0.068
Q96MV1	TLCD4	-3.473	-3.327	-3.199	-3.202	-3.400	-3.201	0.223	0.199
Q9H501	ESF1	3.572	3.537	3.524	3.511	3.555	3.517	0.251	-0.037
P27348	YWHAQ	4.706	4.582	4.566	4.493	4.644	4.530	0.280	-0.114
Q9H1C7	CYSTM1	-1.599	-1.754	-1.408	-1.512	-1.677	-1.460	0.163	0.217
Q9UKA4	AKAP11	-1.706	-1.785	-1.929	-1.851	-1.746	-1.890	0.122	-0.145
Q9BTU6	PI4K2A	1.953	1.893	1.887	1.883	1.923	1.885	0.423	-0.038
Q00978	IRF9	0.021	0.021	0.463	0.425	0.021	0.444	0.029	0.423
P09874	PARP1	7.238	7.182	7.222	7.184	7.210	7.203	0.850	-0.007
Q96MG7	NSMCE3	0.686	0.607	0.629	0.563	0.647	0.596	0.435	-0.051
Q96LL9	DNAJC30	-1.023	-1.086	-1.134	-1.092	-1.054	-1.113	0.278	-0.059
P04632	CAPNS1	1.999	1.970	2.033	2.020	1.984	2.027	0.170	0.042
Q9H2Y7	ZNF106	-1.904	-1.998	-1.943	-2.003	-1.951	-1.973	0.738	-0.022
Q8NBJ5	COLGALT1	4.200	4.116	4.235	4.239	4.158	4.237	0.312	0.079
Q9Y4C1	KDM3A	1.991	1.934	2.050	1.965	1.962	2.007	0.486	0.045
O60487	MPZL2	-4.128	-4.127	-4.101	-3.954	-4.127	-4.027	0.404	0.100
P52756	RBM5	1.259	1.232	1.270	1.251	1.246	1.261	0.476	0.015
P62312	LSM6	2.497	2.373	2.604	2.540	2.435	2.572	0.230	0.137
Q68E01	INTS3	-3.676	-3.637	-3.748	-3.724	-3.656	-3.736	0.093	-0.079

Q9C0B7	TANGO6	1.138	1.042	0.990	0.995	1.090	0.992	0.290	-0.098
P14550	AKR1A1	3.414	3.405	3.516	3.515	3.410	3.515	0.026	0.106
P25787	PSMA2	3.420	3.314	3.391	3.369	3.367	3.380	0.843	0.013
P50895	BCAM	1.266	1.471	1.589	1.696	1.368	1.643	0.181	0.274
Q96HW7	INTS4	0.784	0.825	0.770	0.797	0.804	0.784	0.504	-0.021
Q9UHV7	MED13	0.415	0.358	0.428	0.416	0.386	0.422	0.422	0.036
Q9H5H4	ZNF768	0.608	0.574	0.726	0.742	0.591	0.734	0.041	0.143
Q9P0R6	GSKIP	-3.523	-3.428	-3.500	-3.468	-3.476	-3.484	0.894	-0.008
P49756	RBM25	2.836	2.675	2.806	2.691	2.755	2.749	0.953	-0.007
Q9NS86	LANCL2	1.558	1.581	1.678	1.615	1.569	1.647	0.213	0.077
Q9H0F6	SHARPIN	-4.056	-4.124	-3.859	-4.129	-4.090	-3.994	0.607	0.096
Q9UHK0	NUFIP1	-1.512	-1.439	-1.471	-1.484	-1.476	-1.477	0.970	-0.002
O14493	CLDN4	-3.221	-3.081	-3.170	-3.232	-3.151	-3.201	0.605	-0.050
Q5VUA4	ZNF318	0.254	0.078	0.185	0.071	0.166	0.128	0.756	-0.038
Q9H116	GZF1	-2.107	-2.045	-2.256	-1.967	-2.076	-2.112	0.847	-0.036
Q9GZZ1	NAA50	1.666	1.757	1.477	1.520	1.712	1.498	0.090	-0.213
O00193	SMAP	2.640	2.564	2.778	2.773	2.602	2.776	0.137	0.174
Q02539	H1	-1.657	-0.506	-1.403	-1.417	-1.082	-1.410	0.670	-0.328
Q7Z460	CLASP1	-3.113	-3.169	-3.124	-2.979	-3.141	-3.052	0.422	0.089
Q9NUQ7	UFSP2	1.356	1.339	1.386	1.424	1.348	1.405	0.167	0.057
Q8N3E9	PLCD3	-0.077	0.000	-0.068	0.026	-0.038	-0.021	0.804	0.017
Q9BYG3	NIFK	3.468	3.488	3.552	3.545	3.478	3.549	0.070	0.071
P09601	HMOX1	-0.744	-0.867	-0.424	-0.500	-0.805	-0.462	0.059	0.344
Q9NXZ2	DDX43	-1.052	-1.170	-0.705	-0.988	-1.111	-0.846	0.285	0.264
P16403	H1	4.497	4.508	4.422	4.369	4.503	4.396	0.144	-0.107
Q9UBQ6	EXTL2	-2.811	-2.955	-3.200	-2.904	-2.883	-3.052	0.444	-0.169
Q9BRT3	MIEN1	-0.123	-0.250	-0.038	-0.028	-0.187	-0.033	0.250	0.153
Q6ZU65	UBN2	-2.120	-2.051	-2.130	-2.056	-2.086	-2.093	0.890	-0.008
O60725	ICMT	-0.245	-0.329	-0.330	-0.428	-0.287	-0.379	0.291	-0.092
Q9NYQ6	CELSR1	-2.593	-2.592	-2.557	-2.438	-2.592	-2.497	0.356	0.095
O00148	DDX39A	0.499	-0.011	0.393	-0.018	0.244	0.187	0.879	-0.057
Q9P265	DIP2B	2.048	2.006	2.053	2.072	2.027	2.062	0.311	0.036
Q9P0P8	MTRES1	0.865	0.917	0.824	0.975	0.891	0.899	0.931	0.008
Q12899	TRIM26	0.711	0.765	0.599	0.593	0.738	0.596	0.114	-0.142
Q69YL0	NCBP2AS2	-0.561	-0.598	-0.810	-0.764	-0.579	-0.787	0.023	-0.208

O43143	DHX15	5.286	5.250	5.241	5.220	5.268	5.230	0.247	-0.037
P53816	PLAAT3	-2.943	-2.865	-2.988	-3.042	-2.904	-3.015	0.160	-0.111
Q9NSY1	BMP2K	-2.073	-1.960	-2.158	-1.868	-2.016	-2.013	0.987	0.003
Q99733	NAP1L4	-5.277	-4.937	-5.262	-4.777	-5.107	-5.019	0.798	0.088
O43396	TXNL1	3.402	3.323	3.250	3.236	3.362	3.243	0.195	-0.119
Q9P0T7	TMEM9	-2.614	-2.800	-2.805	-2.783	-2.707	-2.794	0.522	-0.087
O14618	CCS	2.116	2.144	2.027	2.103	2.130	2.065	0.317	-0.064
Q15363	TMED2	3.403	3.369	3.356	3.354	3.386	3.355	0.317	-0.031
Q9Y4U1	MMACHC	-1.409	-1.569	-1.716	-1.617	-1.489	-1.666	0.225	-0.177
O60476	MAN1A2	-1.147	-1.349	-1.256	-1.357	-1.248	-1.306	0.674	-0.058
A1L188	NDUFAF8	-3.494	-3.484	-3.642	-3.626	-3.489	-3.634	0.008	-0.145
Q96KM6	ZNF512B	1.106	1.073	1.106	1.069	1.090	1.087	0.941	-0.002
Q9GZR2	REXO4	2.839	2.832	2.952	2.899	2.836	2.925	0.177	0.090
P14927	UQCRB	1.430	1.485	1.333	1.414	1.457	1.374	0.246	-0.084
P30050	RPL12	6.002	5.983	6.024	5.986	5.992	6.005	0.618	0.013
Q92922	SMARCC1	1.905	2.103	1.909	1.890	2.004	1.900	0.483	-0.104
Q9H0B6	KLC2	-0.913	-0.946	-1.052	-1.072	-0.929	-1.062	0.034	-0.133
Q8NFW8	CMAS	2.388	2.162	2.541	2.444	2.275	2.493	0.276	0.218
Q14738	PPP2R5D	-0.133	0.028	-0.128	-0.099	-0.053	-0.114	0.587	-0.061
P24539	ATP5PB	3.717	3.680	3.707	3.743	3.699	3.725	0.409	0.026
O14495	PLPP3	-0.650	-0.859	-0.432	-0.461	-0.754	-0.446	0.202	0.308
P78357	CNTNAP1	-0.030	-0.116	0.052	0.122	-0.073	0.087	0.107	0.160
P14625	HSP90B1	7.229	7.211	7.247	7.190	7.220	7.219	0.960	-0.002
Q96CB8	INTS12	0.655	0.710	0.712	0.722	0.682	0.717	0.420	0.035
Q69YI7	NAIF1	-5.469	-5.460	-5.363	-5.453	-5.464	-5.408	0.424	0.057
Q9H3K2	GHITM	2.927	2.938	3.194	3.133	2.933	3.164	0.075	0.231
P35579	MYH9	5.426	5.522	5.507	5.384	5.474	5.445	0.752	-0.029
P25815	S100P	0.425	1.092	0.900	0.445	0.758	0.672	0.853	-0.086
P17481	HOXB8	-0.501	-0.582	-0.807	-0.796	-0.541	-0.801	0.094	-0.260
Q8IZL2	MAML2	-3.765	-3.786	-3.801	-3.675	-3.775	-3.738	0.657	0.038
Q68CQ7	GLT8D1	-1.488	-1.467	-1.353	-1.361	-1.477	-1.357	0.033	0.120
Q9NUP9	LIN7C	0.778	0.700	0.636	0.624	0.739	0.630	0.212	-0.109
Q05655	PRKCD	-3.877	-3.697	-3.591	-3.733	-3.787	-3.662	0.392	0.125
Q9H2W6	MRPL46	2.205	2.312	2.203	2.230	2.258	2.216	0.575	-0.042
P49770	EIF2B2	2.426	2.461	2.345	2.437	2.443	2.391	0.448	-0.052

O43761	SYNGR3	-4.580	-4.736	-4.391	-4.266	-4.658	-4.328	0.086	0.330
Q8TF65	GIPC2	-4.616	-4.236	-4.573	-4.530	-4.426	-4.551	0.628	-0.125
Q9H6P5	TASP1	-1.744	-1.840	-1.606	-1.765	-1.792	-1.686	0.392	0.106
Q8IUC6	TICAM1	-4.321	-4.329	-4.237	-4.257	-4.325	-4.247	0.048	0.078
Q9Y4P3	TBL2	3.905	3.861	3.918	3.897	3.883	3.908	0.456	0.025
Q8N490	PNKD	-3.322	-3.311	-3.291	-3.287	-3.316	-3.289	0.086	0.027
Q9UN76	SLC6A14	-3.342	-3.462	-3.044	-3.302	-3.402	-3.173	0.297	0.229
Q5SZD1	C6orf141	-7.638	-7.134	-7.363	-7.000	-7.386	-7.181	0.583	0.205
Q96A49	SYAP1	3.933	3.816	3.742	3.750	3.874	3.746	0.270	-0.128
Q9Y624	F11R	-2.534	-1.720	-2.256	-2.326	-2.127	-2.291	0.756	-0.164
Q9BSH4	TACO1	1.842	1.722	1.716	1.728	1.782	1.722	0.501	-0.060
P61457	PCBD1	1.974	1.940	1.927	1.955	1.957	1.941	0.549	-0.016
Q96RQ1	ERGIC2	0.758	0.772	0.826	0.826	0.765	0.826	0.074	0.061
P34096	RNASE4	-3.974	-3.952	-4.177	-3.988	-3.963	-4.083	0.423	-0.120
Q9H8M7	MINDY3	-1.623	-1.718	-1.730	-1.697	-1.671	-1.714	0.525	-0.043
Q8IWE4	DCUN1D3	-1.417	-1.189	-1.439	-1.155	-1.303	-1.297	0.978	0.006
Q8N1G0	ZNF687	-0.544	-0.653	-0.520	-0.634	-0.598	-0.577	0.810	0.022
Q9P2Y5	UVRAG	1.064	1.008	0.996	0.983	1.036	0.989	0.334	-0.047
Q96BX8	MOB3A	0.844	0.775	0.553	0.637	0.810	0.595	0.062	-0.215
Q15386	UBE3C	2.665	2.649	2.552	2.592	2.657	2.572	0.114	-0.084
Q9Y6I9	TEX264	0.288	0.239	-0.047	-0.100	0.263	-0.073	0.012	-0.336
Q7Z3V4	UBE3B	-1.271	-1.341	-1.123	-1.154	-1.306	-1.139	0.090	0.167
Q96A26	FAM162A	2.877	2.727	2.803	2.779	2.802	2.791	0.909	-0.011
Q96CS3	FAF2	3.476	3.477	3.438	3.457	3.476	3.448	0.206	-0.028
Q9C0E2	XPO4	1.753	1.777	1.663	1.745	1.765	1.704	0.363	-0.061
Q9Y580	RBM7	0.546	0.435	0.605	0.584	0.490	0.595	0.303	0.104
Q9C0J8	WDR33	2.020	2.079	2.035	2.031	2.049	2.033	0.679	-0.016
Q8N5I9	NOPCHAP1	1.400	1.206	1.256	1.318	1.303	1.287	0.897	-0.016
P83731	RPL24	6.552	6.386	6.475	6.390	6.469	6.432	0.742	-0.037
Q96EB6	SIRT1	0.497	0.535	0.592	0.659	0.516	0.626	0.136	0.109
Q9NRR5	UBQLN4	0.537	0.480	0.480	0.479	0.508	0.479	0.495	-0.029
O94888	UBXN7	2.724	2.641	2.650	2.688	2.683	2.669	0.799	-0.014
P82664	MRPS10	1.494	1.706	1.536	1.525	1.600	1.530	0.630	-0.070
Q9P1F3	ABRACL	1.504	1.608	1.567	1.575	1.556	1.571	0.817	0.015
Q96G03	PGM2	2.980	3.075	2.986	2.975	3.027	2.980	0.503	-0.047

O43818	RRP9	2.448	2.449	2.456	2.461	2.449	2.459	0.115	0.010
Q8N5M4	TTC9C	-0.381	-0.371	-0.415	-0.368	-0.376	-0.391	0.630	-0.015
Q9NR50	EIF2B3	0.276	0.148	0.295	0.154	0.212	0.224	0.910	0.012
O75911	DHRS3	-3.512	-3.297	-3.282	-3.267	-3.404	-3.275	0.441	0.130
Q9Y383	LUC7L2	1.961	1.897	2.062	2.042	1.929	2.052	0.138	0.123
Q9Y2R4	DDX52	2.460	2.469	2.467	2.439	2.465	2.453	0.552	-0.012
Q86WC4	OSTM1	-0.811	-0.864	-0.753	-0.832	-0.838	-0.793	0.459	0.045
Q86WK9	PAQR7	-2.969	-3.180	-3.390	-3.415	-3.075	-3.402	0.195	-0.327
Q8N0U4	FAM185A	-3.712	-3.765	-3.698	-3.873	-3.739	-3.786	0.688	-0.047
Q9UDX5	MTFP1	-0.523	-0.506	-0.411	-0.341	-0.515	-0.376	0.140	0.139
Q6SJ93	FAM111B	-3.393	-3.533	-3.888	-3.781	-3.463	-3.834	0.058	-0.372
P16035	TIMP2	0.193	0.221	0.160	0.199	0.207	0.180	0.381	-0.027
Q15646	OASL	-1.098	-0.860	-0.193	-0.074	-0.979	-0.133	0.048	0.846
O15063	GARRE1	-2.644	-2.608	-2.560	-2.432	-2.626	-2.496	0.272	0.130
O15498	YKT6	1.532	1.324	1.476	1.320	1.428	1.398	0.843	-0.030
Q86WR7	PROSER2	1.387	0.896	4.914	4.789	1.142	4.852	0.032	3.710
Q14692	BMS1	3.545	3.517	3.504	3.553	3.531	3.528	0.937	-0.003
O14578	CIT	0.187	0.023	-0.272	-0.460	0.105	-0.366	0.065	-0.471
Q9UHD2	TBK1	1.902	1.878	1.876	1.874	1.890	1.875	0.431	-0.015
P06865	HEXA	2.159	2.199	2.429	2.437	2.179	2.433	0.043	0.254
Q5VWQ0	RSBN1	-0.140	-0.194	-0.221	-0.125	-0.167	-0.173	0.927	-0.006
Q5SYE7	NHSL1	-3.941	-4.035	-3.945	-4.173	-3.988	-4.059	0.647	-0.071
Q9BRX5	GINS3	-2.152	-2.226	-2.427	-2.557	-2.189	-2.492	0.081	-0.303
Q8WUU5	GATAD1	-2.810	-2.914	-2.790	-2.820	-2.862	-2.805	0.461	0.057
Q15393	SF3B3	3.283	3.323	3.194	3.295	3.303	3.244	0.441	-0.059
P26022	PTX3	0.967	0.812	0.471	0.570	0.889	0.520	0.074	-0.369
P48735	IDH2	-2.384	-2.453	-2.328	-2.421	-2.418	-2.375	0.534	0.044
O15120	AGPAT2	-0.428	-0.579	-0.563	-0.571	-0.503	-0.567	0.551	-0.064
Q6P4Q7	CNNM4	-1.210	-1.091	-1.064	-1.016	-1.150	-1.040	0.287	0.110
O15228	GNPAT	-0.290	-0.476	-0.504	-0.618	-0.383	-0.561	0.271	-0.177
Q8N442	GUF1	-0.059	-0.073	0.012	0.014	-0.066	0.013	0.048	0.079
Q9H1K0	RBSN	-1.062	-1.126	-1.048	-1.125	-1.094	-1.087	0.899	0.007
O75509	TNFRSF21	-2.937	-2.832	-2.788	-2.534	-2.884	-2.661	0.303	0.224
Q9NRG7	SDR39U1	0.138	0.037	0.265	0.158	0.088	0.212	0.234	0.124
Q4FZB7	KMT5B	-4.358	-4.580	-4.294	-4.198	-4.469	-4.246	0.262	0.223

Q7Z6K3	PTAR1	-1.091	-1.023	-1.153	-1.045	-1.057	-1.099	0.594	-0.042
O60678	PRMT3	-0.240	-0.326	-0.354	-0.440	-0.283	-0.397	0.201	-0.114
Q5T7V8	GORAB	-2.213	-2.201	-2.301	-2.149	-2.207	-2.225	0.850	-0.018
O60264	SMARCA5	4.807	4.779	4.833	4.775	4.793	4.804	0.774	0.011
Q9H3N1	TMX1	3.606	3.662	3.510	3.496	3.634	3.503	0.119	-0.131
P28482	MAPK1	3.968	3.989	4.018	3.876	3.978	3.947	0.737	-0.031
P17026	ZNF22	0.072	-0.169	0.084	-0.155	-0.048	-0.035	0.945	0.013
Q9UQE7	SMC3	5.038	5.014	5.012	4.916	5.026	4.964	0.415	-0.062
Q9BRP1	PDCD2L	0.909	0.857	0.809	0.846	0.883	0.827	0.238	-0.056
Q99985	SEMA3C	-1.804	-1.791	-1.927	-1.963	-1.797	-1.945	0.053	-0.148
O75323	NIPSNAP2	2.772	2.592	2.843	2.702	2.682	2.773	0.515	0.090
P46779	RPL28	2.876	2.832	3.027	2.891	2.854	2.959	0.350	0.105
Q9Y3E0	GOLT1B	0.198	0.057	0.296	0.247	0.127	0.272	0.265	0.144
O15551	CLDN3	-1.744	-1.049	-1.553	-1.608	-1.397	-1.580	0.691	-0.184
Q5XKP0	MICOS13	2.531	2.396	2.578	2.525	2.463	2.552	0.399	0.088
P43307	SSR1	0.296	0.317	0.285	0.222	0.307	0.254	0.324	-0.053
Q02878	RPL6	7.432	7.339	7.439	7.408	7.385	7.424	0.556	0.038
P40123	CAP2	-3.653	-3.754	-3.543	-3.669	-3.704	-3.606	0.355	0.098
Q9Y2K3	MYH15	-2.946	-3.037	-3.000	-2.837	-2.991	-2.919	0.536	0.073
P41743	PRKCI	1.066	1.035	0.935	0.922	1.050	0.928	0.047	-0.122
Q15942	ZYX	1.115	0.997	1.141	1.067	1.056	1.104	0.570	0.048
Q02446	SP4	-2.659	-2.647	-2.615	-2.715	-2.653	-2.665	0.853	-0.012
Q9H0N5	PCBD2	-0.360	-0.213	-0.246	-0.261	-0.286	-0.253	0.732	0.033
O95183	VAMP5	-1.407	-1.492	-1.591	-1.558	-1.450	-1.574	0.175	-0.125
Q6P2P2	PRMT9	-3.090	-3.165	-2.840	-2.721	-3.128	-2.780	0.054	0.347
O15344	MID1	-2.881	-2.380	-2.748	-2.715	-2.630	-2.731	0.755	-0.101
Q9C002	NMES1	-2.457	-2.499	-2.505	-2.542	-2.478	-2.523	0.250	-0.045
Q8TF47	ZFP90	-2.016	-2.086	-2.024	-2.025	-2.051	-2.024	0.580	0.027
P83436	COG7	1.099	1.035	0.984	0.998	1.067	0.991	0.242	-0.075
Q9H1B5	XYLT2	-0.443	-0.536	-0.624	-0.601	-0.490	-0.613	0.214	-0.123
Q7Z7L1	SLFN11	-1.129	-0.743	-0.907	-0.859	-0.936	-0.883	0.830	0.053
Q9BY77	POLDIP3	-2.456	-2.564	-2.449	-2.626	-2.510	-2.538	0.819	-0.028
P18669	PGAM1	6.086	6.092	5.990	6.043	6.089	6.016	0.221	-0.073
Q9H425	C1orf198	-2.981	-3.004	-3.285	-2.889	-2.992	-3.087	0.716	-0.095
Q6P1X5	TAF2	1.510	1.483	1.530	1.544	1.496	1.537	0.151	0.041

Q96Q83	ALKBH3	-3.909	-3.835	-3.893	-3.636	-3.872	-3.765	0.555	0.107
O95528	SLC2A10	-3.329	-3.226	-3.332	-3.125	-3.277	-3.228	0.726	0.049
Q6ZSJ8	C1orf122	-1.732	-1.799	-1.706	-1.645	-1.765	-1.676	0.187	0.090
Q99447	PCYT2	-5.404	-4.649	-5.558	-5.388	-5.026	-5.473	0.441	-0.447
Q8WZA9	IRGQ	1.349	1.360	1.111	1.144	1.354	1.128	0.030	-0.227
Q9BU70	TRMO	-3.733	-3.725	-3.882	-3.683	-3.729	-3.782	0.688	-0.053
P11216	PYGB	7.750	7.652	7.472	7.464	7.701	7.468	0.130	-0.233
O43290	SART1	3.871	3.885	3.873	3.851	3.878	3.862	0.367	-0.016
Q9UEU0	VTI1B	-0.954	-0.987	-0.940	-1.031	-0.970	-0.985	0.800	-0.015
P62888	RPL30	6.053	6.032	5.988	5.947	6.043	5.968	0.119	-0.075
Q9Y3L5	RAP2C	-2.632	-2.530	-2.290	-2.223	-2.581	-2.257	0.046	0.324
O95232	LUC7L3	2.577	2.553	2.588	2.606	2.565	2.597	0.183	0.032
Q8IYB7	DIS3L2	0.526	0.503	0.202	0.550	0.514	0.376	0.573	-0.138
P24844	MYL9	-2.620	-2.716	-2.919	-3.070	-2.668	-2.994	0.086	-0.326
Q13523	PRPF4B	3.345	3.223	3.361	3.252	3.284	3.307	0.810	0.022
Q15388	TOMM20	1.992	2.016	1.968	1.943	2.004	1.956	0.105	-0.049
Q8NC96	NECAP1	0.554	0.405	0.383	0.310	0.479	0.346	0.294	-0.133
O43813	LANCL1	2.380	2.396	2.422	2.337	2.388	2.379	0.872	-0.009
P00450	CP	-4.149	-4.287	-3.868	-4.205	-4.218	-4.037	0.467	0.181
P01033	TIMP1	0.394	0.263	0.284	0.385	0.329	0.334	0.953	0.006
Q9H910	JPT2	-4.997	-5.220	-5.274	-5.127	-5.109	-5.201	0.572	-0.092
Q99717	SMAD5	-1.266	-1.312	-1.399	-1.392	-1.289	-1.395	0.129	-0.106
P16402	H1	5.561	5.635	5.579	5.572	5.598	5.575	0.645	-0.023
Q9BVC5	C2orf49	-1.412	-1.384	-1.518	-1.400	-1.398	-1.459	0.486	-0.061
Q9NR09	BIRC6	3.908	3.839	3.792	3.783	3.873	3.787	0.235	-0.086
P28070	PSMB4	3.389	3.262	3.328	3.149	3.326	3.239	0.520	-0.087
O95613	PCNT	-2.213	-2.422	-2.292	-2.471	-2.318	-2.382	0.687	-0.064
Q9NYS0	NKIRAS1	-5.721	-5.685	-5.812	-5.866	-5.703	-5.839	0.066	-0.136
Q92504	SLC39A7	-1.145	-1.104	-0.956	-0.960	-1.124	-0.958	0.075	0.166
Q96S99	PLEKHF1	-1.381	-1.383	-1.650	-1.699	-1.382	-1.675	0.052	-0.293
Q96AY4	TTC28	0.694	0.538	0.724	0.697	0.616	0.710	0.436	0.094
O95274	LYPD3	-1.415	-1.370	-1.476	-1.388	-1.393	-1.432	0.531	-0.039
Q9Y4L5	RNF115	-4.292	-4.259	-4.402	-4.287	-4.275	-4.345	0.433	-0.069
Q8N9T8	KRI1	3.455	3.354	3.397	3.386	3.405	3.392	0.840	-0.013
Q2PZI1	DPY19L1	-2.156	-1.988	-1.901	-2.117	-2.072	-2.009	0.694	0.063

Q96E52	OMA1	-0.866	-1.127	-0.922	-1.077	-0.996	-1.000	0.986	-0.003
P27708	CAD	6.242	6.212	6.095	6.119	6.227	6.107	0.027	-0.121
Q9Y262	EIF3L	-2.471	-2.162	-2.652	-2.214	-2.317	-2.433	0.711	-0.116
Q8WX92	NELFB	-2.979	-2.940	-2.914	-2.820	-2.959	-2.867	0.273	0.092
Q7Z7E8	UBE2Q1	-0.748	-0.723	-0.787	-0.734	-0.735	-0.760	0.506	-0.025
P51946	CCNH	1.878	1.884	1.901	1.943	1.881	1.922	0.299	0.041
O95154	AKR7A3	-4.451	-3.986	-4.452	-4.105	-4.219	-4.278	0.858	-0.059
Q9NR77	PXMP2	0.529	0.668	0.498	0.610	0.599	0.554	0.669	-0.045
Q8IVS2	MCAT	1.472	1.498	1.462	1.493	1.485	1.478	0.750	-0.007
Q12986	NFX1	-1.810	-1.817	-1.806	-1.973	-1.814	-1.889	0.530	-0.076
Q9NUW8	TDP1	-1.143	-1.572	-1.716	-1.662	-1.357	-1.689	0.362	-0.331
Q9Y6X4	FAM169A	-1.958	-1.824	-1.874	-1.834	-1.891	-1.854	0.677	0.037
Q9NU23	LYRM2	-0.802	-0.797	-0.942	-0.891	-0.800	-0.916	0.134	-0.116
Q13572	ITPK1	-0.570	-0.390	-0.677	-0.616	-0.480	-0.646	0.295	-0.166
O00401	WASL	1.037	1.047	1.150	1.103	1.042	1.126	0.159	0.085
P48060	GLIPR1	-4.156	-4.009	-4.035	-3.963	-4.082	-3.999	0.450	0.083
P62875	POLR2L	0.545	0.414	0.452	0.380	0.480	0.416	0.506	-0.063
Q8N4J0	CARNMT1	-1.879	-1.863	-1.693	-1.811	-1.871	-1.752	0.288	0.119
Q13630	GFUS	2.690	2.813	2.774	2.757	2.751	2.765	0.859	0.014
P03886	MT	0.064	0.193	0.191	0.214	0.129	0.202	0.452	0.074
Q5SW79	CEP170	-0.375	-0.455	-0.327	-0.337	-0.415	-0.332	0.281	0.083
Q8NAF0	ZNF579	-0.930	-0.840	-1.000	-0.812	-0.885	-0.906	0.867	-0.021
A6ND36	FAM83G	1.547	1.506	1.563	1.527	1.527	1.545	0.562	0.019
P40938	RFC3	-1.475	-1.350	-1.749	-1.540	-1.412	-1.644	0.225	-0.232
P41212	ETV6	0.797	0.778	0.747	0.711	0.788	0.729	0.138	-0.059
Q01201	RELB	-1.188	-1.226	-0.924	-0.933	-1.207	-0.928	0.034	0.279
Q96NY9	MUS81	-1.559	-1.591	-1.607	-1.681	-1.575	-1.644	0.286	-0.069
P36578	RPL4	7.738	7.681	7.723	7.670	7.709	7.696	0.766	-0.013
Q9Y5S2	CDC42BPB	3.114	3.204	3.131	3.163	3.159	3.147	0.838	-0.012
O00264	PGRMC1	4.262	4.278	4.277	4.256	4.270	4.266	0.787	-0.004
A1L020	MEX3A	-2.965	-2.951	-3.070	-2.870	-2.958	-2.970	0.925	-0.012
Q3ZCN5	OTOGL	-6.298	-6.452	-6.270	-6.500	-6.375	-6.385	0.949	-0.010
O43189	PHF1	-2.381	-2.379	-2.140	-2.099	-2.380	-2.120	0.049	0.260
P07814	EPRS1	6.827	6.768	6.886	6.853	6.798	6.869	0.200	0.072
Q70J99	UNC13D	-4.257	-3.989	-4.195	-4.211	-4.123	-4.203	0.659	-0.080

P31150	GDI1	3.562	3.587	3.610	3.638	3.575	3.624	0.119	0.049
Q9HD67	MYO10	-2.173	-2.175	-2.174	-2.003	-2.174	-2.089	0.500	0.086
Q9BUP3	HTATIP2	-3.805	-3.687	-3.746	-3.705	-3.746	-3.725	0.790	0.020
P24468	NR2F2	-3.578	-3.628	-4.197	-4.008	-3.603	-4.102	0.101	-0.499
P45973	CBX5	1.992	2.092	1.968	1.954	2.042	1.961	0.348	-0.081
P61960	UFM1	1.275	1.203	1.351	1.285	1.239	1.318	0.248	0.079
P09382	LGALS1	5.841	5.855	5.794	5.688	5.848	5.741	0.287	-0.107
Q96BI1	SLC22A18	0.823	0.622	0.793	0.771	0.723	0.782	0.658	0.060
Q6ZUX7	LHFPL2	-2.435	-2.722	-2.447	-2.505	-2.579	-2.476	0.603	0.103
Q96PK6	RBM14	3.693	3.714	3.722	3.732	3.703	3.727	0.221	0.024
Q9Y276	BCS1L	2.263	2.143	2.253	2.276	2.203	2.264	0.490	0.061
P06576	ATP5F1B	7.289	7.223	7.294	7.289	7.256	7.292	0.472	0.036
P80297	MT1X	-2.918	-3.053	-2.414	-2.446	-2.986	-2.430	0.064	0.556
Q9NRF8	CTPS2	3.392	3.380	3.246	3.290	3.386	3.268	0.099	-0.118
O15116	LSM1	1.060	0.868	0.877	0.709	0.964	0.793	0.315	-0.171
Q9Y613	FHOD1	1.516	1.421	1.256	1.313	1.468	1.284	0.104	-0.184
Q12965	MYO1E	1.603	1.837	1.566	1.658	1.720	1.612	0.519	-0.108
Q9HCE3	ZNF532	-0.802	-0.613	-0.816	-0.620	-0.708	-0.718	0.948	-0.010
P11831	SRF	-1.703	-1.804	-1.631	-1.704	-1.754	-1.667	0.310	0.086
P36957	DLST	0.194	0.199	0.354	0.807	0.196	0.580	0.340	0.384
Q13601	KRR1	-1.384	-1.138	-1.131	-0.929	-1.261	-1.030	0.288	0.231
Q8WTS6	SETD7	-0.476	-0.422	-0.350	-0.274	-0.449	-0.312	0.113	0.137
Q49MG5	MAP9	-3.691	-3.587	-3.449	-3.456	-3.639	-3.453	0.173	0.186
O95630	STAMBP	0.827	0.800	0.868	0.772	0.813	0.820	0.919	0.006
P14854	COX6B1	2.733	2.755	2.649	2.669	2.744	2.659	0.030	-0.085
Q9UQB8	BAIAP2	-4.791	-3.962	-4.409	-4.515	-4.376	-4.462	0.871	-0.085
O43819	SCO2	-0.014	-0.138	-0.011	-0.115	-0.076	-0.063	0.891	0.013
Q6P2E9	EDC4	2.933	2.858	2.767	2.758	2.895	2.763	0.171	-0.133
Q96EV2	RBM33	1.401	1.308	1.356	1.323	1.355	1.340	0.804	-0.015
Q9Y324	FCF1	0.733	0.772	0.726	0.825	0.752	0.775	0.728	0.023
Q9Y3D9	MRPS23	2.528	2.565	2.472	2.476	2.547	2.474	0.156	-0.073
O96007	MOCS2	-0.684	-0.733	-0.830	-0.766	-0.709	-0.798	0.168	-0.089
O95202	LETM1	3.681	3.547	3.756	3.644	3.614	3.700	0.432	0.086
Q7L0Y3	TRMT10C	3.958	3.985	3.966	4.012	3.971	3.989	0.582	0.018
Q9Y4G2	PLEKHM1	-2.400	-2.435	-2.523	-2.353	-2.417	-2.438	0.849	-0.021

Q6UX04	CWC27	-0.735	-0.773	-0.854	-0.779	-0.754	-0.816	0.319	-0.062
Q08AM6	VAC14	2.726	2.751	2.786	2.758	2.739	2.772	0.218	0.034
Q96JB2	COG3	0.319	0.219	0.411	0.293	0.269	0.352	0.400	0.083
Q9Y305	ACOT9	-3.168	-3.278	-3.329	-3.140	-3.223	-3.234	0.927	-0.012
Q13442	PDAP1	4.324	4.231	4.364	4.249	4.278	4.307	0.734	0.029
P49643	PRIM2	1.113	1.123	0.874	0.923	1.118	0.898	0.061	-0.220
P61024	CKS1B	-0.884	-0.828	-1.120	-1.094	-0.856	-1.107	0.037	-0.252
P37837	TALDO1	6.944	6.840	6.933	6.895	6.892	6.914	0.745	0.022
Q9P2M7	CGN	0.744	1.016	0.702	0.941	0.880	0.822	0.778	-0.058
Q9H3M7	TXNIP	-0.249	-0.031	-0.623	-0.284	-0.140	-0.454	0.281	-0.313
O15397	IPO8	0.053	0.042	0.079	0.131	0.048	0.105	0.257	0.057
P62072	TIMM10	1.431	1.519	1.184	1.233	1.475	1.208	0.055	-0.267
Q86V48	LUZP1	-2.749	-2.693	-2.847	-2.776	-2.721	-2.811	0.190	-0.090
Q9UHF1	EGFL7	-2.665	-2.700	-2.601	-2.644	-2.682	-2.623	0.167	0.060
Q13232	NME3	-0.061	-0.136	-0.012	-0.031	-0.099	-0.021	0.276	0.077
P53999	SUB1	4.529	4.469	4.539	4.531	4.499	4.535	0.434	0.037
Q9NYL9	TMOD3	3.610	3.703	3.593	3.614	3.656	3.604	0.457	-0.053
P19387	POLR2C	0.949	0.937	0.972	0.978	0.943	0.975	0.068	0.032
Q8NBX0	SCCPDH	3.193	3.152	3.170	3.176	3.172	3.173	0.975	0.001
Q9ULC4	MCTS1	-0.538	-0.562	-0.554	-0.422	-0.550	-0.488	0.518	0.062
P07947	YES1	0.832	0.723	0.826	0.812	0.778	0.819	0.589	0.041
Q9UJC5	SH3BGRL2	-3.921	-2.310	-3.223	-3.402	-3.116	-3.312	0.848	-0.197
Q99666	RGPD5	-3.684	-3.688	-3.591	-3.568	-3.686	-3.580	0.064	0.106
O60831	PRAF2	1.546	1.378	1.558	1.513	1.462	1.536	0.540	0.073
Q9BSB4	ATG101	-0.669	-0.788	-0.562	-0.556	-0.728	-0.559	0.214	0.169
Q96I99	SUCLG2	1.377	1.286	1.554	1.553	1.332	1.553	0.129	0.222
Q68CQ4	UTP25	2.538	2.534	2.603	2.615	2.536	2.609	0.037	0.073
Q5JVS0	HABP4	-0.596	-0.762	-0.719	-0.715	-0.679	-0.717	0.729	-0.038
O43824	GTPBP6	1.770	1.698	1.761	1.773	1.734	1.767	0.525	0.033
O43572	AKAP10	0.346	0.320	0.257	0.250	0.333	0.253	0.080	-0.080
Q9ULC6	PADI1	-4.655	-4.520	-4.406	-4.601	-4.588	-4.503	0.561	0.084
P35222	CTNNB1	1.249	1.624	1.453	1.376	1.437	1.414	0.924	-0.023
Q8N1B4	VPS52	-2.580	-2.279	-2.481	-1.999	-2.430	-2.240	0.585	0.189
O75362	ZNF217	2.657	2.577	2.447	2.462	2.617	2.454	0.141	-0.163
Q17RN3	FAM98C	-1.190	-1.258	-1.380	-1.412	-1.224	-1.396	0.081	-0.172

O15514	POLR2D	-1.242	-1.264	-1.351	-1.327	-1.253	-1.339	0.036	-0.086
Q86VQ6	TXNRD3	-0.304	-0.485	-0.326	-0.283	-0.395	-0.304	0.494	0.091
Q14686	NCOA6	-0.412	-0.413	-0.521	-0.542	-0.412	-0.531	0.053	-0.119
Q9BSD7	NTPCR	1.213	1.188	1.209	1.237	1.201	1.223	0.358	0.022
P52848	NDST1	-3.132	-3.253	-3.332	-3.196	-3.193	-3.264	0.517	-0.071
Q00765	REEP5	2.976	2.994	3.192	3.103	2.985	3.147	0.158	0.162
O43896	KIF1C	1.321	1.208	1.256	1.260	1.264	1.258	0.932	-0.006
Q5SRI9	MANEA	-1.913	-1.874	-1.831	-1.735	-1.893	-1.783	0.229	0.110
Q8NFH3	NUP43	1.055	1.060	1.070	0.980	1.058	1.025	0.602	-0.033
P48200	IREB2	-1.128	-1.138	-0.854	-0.836	-1.133	-0.845	0.004	0.288
O95571	ETHE1	2.136	2.315	2.271	2.318	2.225	2.295	0.578	0.070
Q16540	MRPL23	1.791	1.759	1.648	1.663	1.775	1.655	0.046	-0.120
P07205	PGK2	-0.037	-0.123	0.001	-0.068	-0.080	-0.033	0.492	0.046
O14964	HGS	-1.191	-1.322	-1.278	-1.463	-1.256	-1.371	0.430	-0.114
O00462	MANBA	-1.160	-1.215	-1.154	-1.119	-1.188	-1.137	0.282	0.051
P27695	APEX1	4.601	4.632	4.572	4.582	4.617	4.577	0.213	-0.039
Q9BWT7	CARD10	-4.372	-4.468	-4.332	-4.431	-4.420	-4.381	0.631	0.039
P42025	ACTR1B	0.107	0.268	0.161	0.174	0.187	0.168	0.847	-0.020
Q8NHP6	MOSPD2	-1.243	-1.195	-1.127	-1.107	-1.219	-1.117	0.107	0.102
Q13011	ECH1	4.854	4.778	5.053	4.968	4.816	5.011	0.077	0.195
O76027	ANXA9	-2.608	-2.677	-2.799	-2.877	-2.642	-2.838	0.066	-0.196
Q8IXZ2	ZC3H3	-1.915	-1.952	-2.017	-1.904	-1.934	-1.960	0.718	-0.027
Q9UI95	MAD2L2	-0.135	-0.360	-0.125	-0.228	-0.248	-0.176	0.643	0.071
Q6I9Y2	THOC7	1.645	1.544	1.606	1.640	1.594	1.623	0.675	0.028
Q9BVC3	DSCC1	-0.188	-0.078	-0.353	-0.253	-0.133	-0.303	0.151	-0.170
Q8NHG7	SVIP	-3.587	-2.759	-3.455	-2.951	-3.173	-3.203	0.957	-0.030
Q9NQZ2	UTP3	2.092	2.055	2.201	2.198	2.074	2.199	0.090	0.126
Q6P2Q9	PRPF8	6.568	6.502	6.526	6.479	6.535	6.502	0.514	-0.033
Q9UKK9	NUDT5	3.855	3.763	3.739	3.686	3.809	3.713	0.241	-0.096
Q15404	RSU1	0.636	0.658	0.636	0.569	0.647	0.603	0.400	-0.044
P10809	HSPD1	8.203	8.183	8.327	8.311	8.193	8.319	0.012	0.126
O14929	HAT1	2.025	2.005	1.808	1.808	2.015	1.808	0.031	-0.207
O43504	LAMTOR5	-1.075	-1.091	-0.933	-0.907	-1.083	-0.920	0.016	0.164
Q9Y6I4	USP3	-2.472	-2.472	-2.401	-2.325	-2.472	-2.363	0.213	0.109
Q5TC12	ATPAF1	-0.569	-0.702	-0.630	-0.590	-0.635	-0.610	0.771	0.025

Q13347	EIF3I	4.595	4.549	4.431	4.471	4.572	4.451	0.059	-0.121
Q5T160	RARS2	1.560	1.525	1.503	1.531	1.542	1.517	0.383	-0.025
P12268	IMPDH2	5.680	5.613	5.495	5.505	5.647	5.500	0.135	-0.147
O15327	INPP4B	0.539	0.468	0.208	0.224	0.503	0.216	0.067	-0.287
P20962	PTMS	1.628	1.565	1.742	1.708	1.596	1.725	0.100	0.129
Q9BWJ5	SF3B5	-0.429	-0.429	-0.527	-0.451	-0.429	-0.489	0.357	-0.061
Q9BT23	LIMD2	-0.404	-0.495	-0.558	-0.533	-0.449	-0.545	0.264	-0.096
Q9H4G4	GLIPR2	-2.342	-2.238	-2.290	-2.280	-2.290	-2.285	0.939	0.005
Q9H6Z4	RANBP3	-2.618	-2.572	-2.652	-2.663	-2.595	-2.657	0.206	-0.063
O75369	FLNB	-0.225	-0.249	0.153	-0.580	-0.237	-0.213	0.959	0.024
Q5W0B1	OBI1	-0.330	-0.304	-0.326	-0.258	-0.317	-0.292	0.591	0.025
Q2KHR3	QSER1	-1.978	-2.029	-2.362	-2.256	-2.003	-2.309	0.067	-0.306
P10515	DLAT	4.729	4.531	4.807	4.684	4.630	4.746	0.443	0.116
P60059	SEC61G	-2.682	-2.821	-2.862	-3.014	-2.752	-2.938	0.213	-0.186
Q8WTS1	ABHD5	-2.527	-2.560	-2.495	-2.384	-2.544	-2.440	0.294	0.104
Q99685	MGLL	-4.899	-5.025	-4.933	-5.089	-4.962	-5.011	0.675	-0.049
O60308	CEP104	-4.366	-4.288	-4.133	-3.894	-4.327	-4.013	0.206	0.314
P49585	PCYT1A	2.326	2.201	2.377	2.298	2.263	2.338	0.436	0.075
Q9H2H9	SLC38A1	0.908	0.890	1.073	1.061	0.899	1.067	0.007	0.168
Q15102	PAFAH1B3	3.316	3.356	3.339	3.319	3.336	3.329	0.789	-0.007
Q9UKL0	RCOR1	2.812	2.750	2.703	2.705	2.781	2.704	0.241	-0.077
Q8IZW8	TNS4	0.678	1.221	0.845	0.992	0.950	0.919	0.929	-0.031
P61011	SRP54	-1.825	-1.968	-2.098	-2.156	-1.896	-2.127	0.155	-0.230
O43167	ZBTB24	-3.482	-3.532	-3.249	-3.167	-3.507	-3.208	0.038	0.299
Q7Z5L9	IRF2BP2	-2.810	-2.735	-2.840	-2.743	-2.773	-2.791	0.791	-0.019
Q8NB66	UNC13C	-5.498	-5.673	-5.537	-5.467	-5.585	-5.502	0.509	0.083
P20290	BTF3	2.668	2.600	2.786	2.610	2.634	2.698	0.597	0.064
Q15773	MLF2	0.954	0.882	1.057	1.054	0.918	1.055	0.164	0.137
Q8N0U8	VKORC1L1	0.426	0.489	0.539	0.545	0.457	0.542	0.223	0.084
O15069	NACAD	-6.544	-6.138	-5.936	-6.284	-6.341	-6.110	0.481	0.231
Q9H6H4	REEP4	-0.742	-0.713	-0.720	-0.648	-0.727	-0.684	0.429	0.043
O15264	MAPK13	-2.003	-1.849	-1.924	-1.928	-1.926	-1.926	1.000	0.000
P28347	TEAD1	-3.590	-3.501	-3.699	-3.610	-3.545	-3.655	0.227	-0.109
O95833	CLIC3	-1.871	-1.543	-1.510	-1.570	-1.707	-1.540	0.491	0.167
P31146	CORO1A	1.079	0.978	0.707	0.761	1.029	0.734	0.061	-0.295

Q96CT7	CCDC124	3.177	3.019	3.113	3.038	3.098	3.075	0.827	-0.023
Q96KR1	ZFR	4.007	3.969	4.149	4.086	3.988	4.118	0.097	0.130
Q96JQ2	CLMN	0.739	0.795	0.773	0.735	0.767	0.754	0.747	-0.013
Q9BS40	LXN	-1.257	-1.063	-0.477	-1.204	-1.160	-0.840	0.537	0.319
Q8TA86	RP9	-0.883	-0.820	-0.848	-0.728	-0.852	-0.788	0.473	0.063
Q96JN0	LCOR	0.163	0.149	0.112	0.148	0.156	0.130	0.375	-0.026
Q9UBV8	PEF1	0.719	0.587	0.706	0.760	0.653	0.733	0.425	0.080
Q5VZK9	CARMIL1	-4.853	-4.385	-4.535	-4.640	-4.619	-4.587	0.915	0.032
Q14739	LBR	3.152	3.249	3.209	3.088	3.201	3.148	0.572	-0.052
P18085	ARF4	3.248	3.360	3.301	3.156	3.304	3.229	0.502	-0.075
Q8IXQ3	C9orf40	-2.493	-2.399	-2.486	-2.283	-2.446	-2.385	0.659	0.061
Q9BXP2	SLC12A9	-0.109	-0.200	-0.178	-0.269	-0.154	-0.224	0.393	-0.069
Q76L83	ASXL2	-2.190	-2.156	-2.144	-1.993	-2.173	-2.069	0.392	0.104
P52788	SMS	3.132	3.097	2.946	2.955	3.114	2.951	0.052	-0.164
Q14CS0	UBXN2B	-1.856	-1.820	-1.918	-1.913	-1.838	-1.916	0.142	-0.077
Q9BTX7	TTPAL	-2.560	-2.536	-2.524	-2.439	-2.548	-2.482	0.349	0.066
P84243	H3	1.214	1.168	1.449	1.370	1.191	1.410	0.061	0.218
Q7Z3T8	ZFYVE16	0.354	0.295	0.203	0.279	0.324	0.241	0.233	-0.083
Q92995	USP13	-3.084	-3.328	-3.183	-3.163	-3.206	-3.173	0.834	0.032
Q9NQS1	AVEN	-3.452	-3.289	-3.457	-3.400	-3.371	-3.429	0.604	-0.058
Q96AJ1	CLUAP1	-2.406	-2.634	-2.610	-2.582	-2.520	-2.596	0.625	-0.076
P18440	NAT1	-3.596	-3.572	-3.379	-3.621	-3.584	-3.500	0.615	0.083
Q9H0C8	ILKAP	3.560	3.433	3.423	3.334	3.497	3.378	0.283	-0.118
Q8WTW3	COG1	1.551	1.674	1.513	1.651	1.613	1.582	0.773	-0.031
P29992	GNA11	0.995	0.860	0.849	0.786	0.927	0.817	0.324	-0.110
Q14207	NPAT	0.045	0.018	-0.148	-0.101	0.031	-0.124	0.047	-0.156
Q9UJT0	TUBE1	-1.199	-1.270	-0.956	-0.786	-1.235	-0.871	0.108	0.363
Q9C075	KRT23	-0.977	-0.313	-0.868	-0.846	-0.645	-0.857	0.637	-0.213
P25208	NFYB	-2.895	-2.721	-2.887	-2.896	-2.808	-2.891	0.514	-0.083
P08473	MME	-3.279	-3.175	-3.106	-3.200	-3.227	-3.153	0.406	0.073
O95210	STBD1	-0.235	-0.214	-0.004	0.092	-0.225	0.044	0.101	0.269
Q9P206	KIAA1522	-2.089	-1.960	-1.925	-1.811	-2.024	-1.868	0.213	0.156
O60513	B4GALT4	-3.177	-3.710	-3.313	-3.757	-3.444	-3.535	0.818	-0.091
Q567U6	CCDC93	1.679	1.847	1.788	1.914	1.763	1.851	0.497	0.088
P52565	ARHGDIA	5.222	5.187	5.187	5.124	5.204	5.156	0.336	-0.049

Q15208	STK38	1.661	1.608	1.639	1.669	1.635	1.654	0.596	0.020
Q9BYD2	MRPL9	2.668	2.662	2.685	2.737	2.665	2.711	0.321	0.047
P62269	RPS18	6.096	6.040	6.081	6.049	6.068	6.065	0.930	-0.003
Q9NUL7	DDX28	1.233	1.230	1.211	1.283	1.231	1.247	0.732	0.016
O95455	TGDS	-2.136	-2.142	-2.131	-2.056	-2.139	-2.093	0.438	0.045
Q9UL46	PSME2	3.706	3.716	3.643	3.666	3.711	3.655	0.090	-0.056
P30876	POLR2B	4.146	4.089	4.141	4.094	4.118	4.117	0.995	0.000
Q7Z2W9	MRPL21	-0.745	-0.579	-0.711	-0.668	-0.662	-0.689	0.800	-0.027
Q9H0G5	NSRP1	1.765	1.806	1.758	1.812	1.785	1.785	0.989	-0.001
Q8N1G4	LRRC47	4.765	4.686	4.817	4.770	4.726	4.794	0.305	0.068
P08574	CYC1	3.735	3.825	3.909	3.842	3.780	3.876	0.242	0.095
Q2TAA2	IAH1	0.566	0.476	0.766	0.669	0.521	0.718	0.098	0.196
O00442	RTCA	-2.356	-2.490	-2.498	-2.357	-2.423	-2.428	0.966	-0.005
O43542	XRCC3	-2.525	-2.595	-2.948	-2.757	-2.560	-2.852	0.168	-0.292
P00441	SOD1	5.064	5.016	5.073	5.100	5.040	5.086	0.264	0.047
Q96D53	COQ8B	-2.429	-2.478	-2.587	-2.503	-2.453	-2.545	0.228	-0.092
O15066	KIF3B	0.031	-0.137	-0.156	-0.012	-0.053	-0.084	0.805	-0.031
Q96ES7	SGF29	-0.151	-0.143	-0.157	-0.074	-0.147	-0.116	0.589	0.031
Q9H0K6	PUS7L	-1.521	-1.324	-1.853	-1.752	-1.423	-1.803	0.112	-0.380
Q86WR0	CCDC25	-0.603	-0.675	-0.749	-0.821	-0.639	-0.785	0.103	-0.146
Q9NPE3	NOP10	1.644	1.669	1.807	1.771	1.656	1.789	0.033	0.133
Q8N1G2	CMTR1	3.230	3.188	3.116	3.120	3.209	3.118	0.140	-0.091
Q92597	NDRG1	-2.692	-2.912	-2.606	-2.104	-2.802	-2.355	0.296	0.447
Q14108	SCARB2	2.010	1.922	2.276	2.013	1.966	2.145	0.388	0.179
Q3SY69	ALDH1L2	-0.894	-1.037	-1.300	-1.290	-0.966	-1.295	0.136	-0.329
Q96T83	SLC9A7	-0.564	-0.518	-0.856	-0.640	-0.541	-0.748	0.297	-0.207
P47755	CAPZA2	2.202	2.218	1.987	2.113	2.210	2.050	0.233	-0.161
Q9H147	DNTTIP1	1.344	1.378	1.227	1.238	1.361	1.233	0.063	-0.128
P23610	F8A1	-0.754	-0.868	-0.747	-0.791	-0.811	-0.769	0.592	0.042
P51659	HSD17B4	1.963	1.844	1.913	1.874	1.904	1.894	0.892	-0.010
Q8N2Y8	RUSC2	-3.480	-3.560	-3.328	-3.302	-3.520	-3.315	0.096	0.205
Q07955	SRSF1	-0.156	-0.323	-0.186	-0.210	-0.240	-0.198	0.703	0.042
P27635	RPL10	5.437	5.428	5.467	5.554	5.432	5.511	0.321	0.078
Q9BRJ7	NUDT16L1	-0.568	-0.651	-0.927	-0.777	-0.610	-0.852	0.138	-0.242
P00367	GLUD1	4.025	4.087	4.400	4.400	4.056	4.400	0.057	0.344

P11387	TOP1	6.706	6.674	6.737	6.673	6.690	6.705	0.736	0.015
Q96IZ0	PAWR	0.290	0.267	0.190	0.138	0.279	0.164	0.102	-0.115
Q8IY18	SMC5	2.535	2.492	2.460	2.439	2.513	2.450	0.164	-0.064
Q9UN37	VPS4A	2.882	2.831	2.825	2.891	2.856	2.858	0.977	0.001
Q86YH6	PDSS2	-0.281	-0.326	-0.377	-0.403	-0.303	-0.390	0.107	-0.087
Q8N129	CNPY4	-2.653	-2.675	-2.563	-2.644	-2.664	-2.603	0.358	0.061
Q8WVE0	EEF1AKMT1	-1.727	-1.830	-1.769	-1.834	-1.778	-1.802	0.742	-0.023
Q9NYJ8	TAB2	-3.279	-3.327	-3.225	-3.232	-3.303	-3.228	0.190	0.074
P30533	LRPAP1	2.099	2.117	2.147	2.141	2.108	2.144	0.129	0.036
Q9BR11	ZSWIM1	-4.928	-5.094	-4.915	-4.934	-5.011	-4.924	0.483	0.087
Q15628	TRADD	-1.052	-1.221	-1.353	-1.243	-1.137	-1.298	0.271	-0.161
Q5T3J3	LRIF1	-0.889	-0.966	-0.855	-0.856	-0.927	-0.855	0.312	0.072
Q8WUY1	THEM6	1.705	1.720	1.474	1.421	1.712	1.448	0.048	-0.265
P49720	PSMB3	5.086	4.930	4.928	4.771	5.008	4.849	0.289	-0.159
Q14CZ7	FASTKD3	-1.310	-1.331	-1.448	-1.354	-1.320	-1.401	0.324	-0.081
Q99575	POP1	3.164	3.161	3.248	3.174	3.162	3.211	0.413	0.049
P54760	EPHB4	-0.202	-0.217	-0.337	-0.235	-0.210	-0.286	0.368	-0.077
P54198	HIRA	-1.740	-1.668	-1.719	-1.777	-1.704	-1.748	0.445	-0.044
O95810	CAVIN2	3.126	2.983	2.767	2.731	3.055	2.749	0.130	-0.305
Q6PCB5	RSBN1L	-1.381	-1.423	-1.492	-1.621	-1.402	-1.556	0.229	-0.154
Q2TAL8	QRICH1	2.537	2.522	2.506	2.469	2.530	2.488	0.232	-0.042
Q16829	DUSP7	-5.341	-5.182	-5.058	-4.861	-5.262	-4.960	0.145	0.302
Q5TAQ9	DCAF8	0.949	0.913	1.002	0.995	0.931	0.998	0.156	0.067
Q9Y2L1	DIS3	0.414	0.308	0.376	0.357	0.361	0.367	0.935	0.005
Q99674	CGREF1	-1.257	-1.376	-1.350	-1.325	-1.316	-1.338	0.777	-0.022
O95873	C6orf47	-1.741	-1.630	-1.879	-1.790	-1.686	-1.834	0.177	-0.149
Q9Y6M9	NDUFB9	1.597	1.582	1.632	1.711	1.589	1.671	0.278	0.082
Q8N9L9	ACOT4	-4.749	-4.369	-4.804	-4.403	-4.559	-4.604	0.886	-0.045
P61353	RPL27	4.484	4.660	4.491	4.663	4.572	4.577	0.972	0.005
Q9Y385	UBE2J1	-2.260	-2.316	-2.245	-2.339	-2.288	-2.292	0.949	-0.004
Q93083	MT1L	-1.345	-1.430	-1.331	-1.351	-1.388	-1.341	0.464	0.047
P41091	EIF2S3	3.250	3.196	3.378	3.256	3.223	3.317	0.343	0.094
P50416	CPT1A	-3.677	-3.597	-3.579	-3.583	-3.637	-3.581	0.392	0.056
Q8IYQ7	THNSL1	2.119	2.164	2.150	2.175	2.142	2.162	0.522	0.021
Q02880	TOP2B	-3.842	-3.870	-3.683	-3.657	-3.856	-3.670	0.011	0.186

Q14562	DHX8	2.087	2.030	2.037	2.035	2.058	2.036	0.575	-0.023
Q9Y6K5	OAS3	0.327	0.544	0.752	0.797	0.435	0.775	0.185	0.339
Q13769	THOC5	2.711	2.648	2.691	2.710	2.680	2.700	0.634	0.020
Q13283	G3BP1	4.543	4.588	4.433	4.460	4.566	4.447	0.064	-0.119
P61586	RHOA	3.037	3.033	3.095	3.019	3.035	3.057	0.669	0.022
O15230	LAMA5	-0.056	0.080	-0.025	0.022	0.012	-0.001	0.878	-0.013
P02794	FTH1	6.035	5.816	6.153	6.049	5.926	6.101	0.327	0.175
Q53S58	TMEM177	0.335	0.293	0.508	0.523	0.314	0.516	0.045	0.202
Q9H9S5	FKRP	-3.453	-3.499	-3.502	-3.415	-3.476	-3.459	0.773	0.017
Q99676	ZNF184	-4.320	-4.258	-4.302	-4.347	-4.289	-4.324	0.463	-0.035
P15121	AKR1B1	1.170	1.355	1.518	1.131	1.263	1.324	0.809	0.062
Q06546	GABPA	2.108	2.074	2.131	2.136	2.091	2.134	0.241	0.043
Q92506	HSD17B8	-1.038	-0.949	-0.901	-0.886	-0.994	-0.894	0.260	0.100
P11279	LAMP1	1.409	1.471	1.597	1.433	1.440	1.515	0.522	0.075
A5D8V6	VPS37C	-0.205	-0.354	-0.404	-0.367	-0.280	-0.385	0.381	-0.106
Q13901	C1D	0.640	0.348	0.555	0.311	0.494	0.433	0.780	-0.061
Q15046	KARS1	2.596	2.561	2.399	2.511	2.578	2.455	0.251	-0.123
Q8WXI9	GATAD2B	2.382	2.523	2.353	2.425	2.453	2.389	0.530	-0.064
Q9H2P0	ADNP	3.678	3.702	3.639	3.624	3.690	3.632	0.070	-0.058
Q8N0Z3	SPICE1	-2.674	-2.695	-2.944	-2.905	-2.684	-2.924	0.020	-0.240
P16260	SLC25A16	-1.276	-1.269	-1.468	-1.067	-1.272	-1.268	0.986	0.004
Q9BQE3	TUBA1C	3.128	3.175	3.004	3.033	3.152	3.019	0.059	-0.133
O15511	ARPC5	1.319	1.221	1.359	1.359	1.270	1.359	0.320	0.089
P10074	ZBTB48	-2.538	-2.545	-2.613	-2.536	-2.542	-2.574	0.555	-0.032
P02792	FTL	5.321	5.231	5.255	5.279	5.276	5.267	0.882	-0.008
Q15369	ELOC	0.003	0.065	0.068	0.103	0.034	0.085	0.313	0.052
Q9H8H0	NOL11	2.690	2.633	2.595	2.686	2.661	2.641	0.747	-0.021
P11182	DBT	2.850	2.811	2.962	2.979	2.830	2.970	0.054	0.140
Q92575	UBXN4	2.973	2.909	2.901	2.862	2.941	2.882	0.276	-0.059
Q9Y388	RBMX2	1.268	1.333	1.263	1.357	1.300	1.310	0.885	0.010
Q8IV36	HID1	-5.887	-5.614	-5.650	-5.758	-5.751	-5.704	0.793	0.047
O14981	BTAF1	2.005	2.015	1.948	2.000	2.010	1.974	0.386	-0.036
Q99618	CDCA3	-1.714	-1.659	-1.913	-1.912	-1.686	-1.913	0.076	-0.227
Q9UGU5	HMGXB4	-0.065	-0.009	0.033	0.078	-0.037	0.056	0.129	0.092
Q13882	PTK6	-3.828	-3.938	-4.321	-3.987	-3.883	-4.154	0.334	-0.271

Q5SRE5	NUP188	0.090	0.227	0.080	0.056	0.158	0.068	0.408	-0.090
Q9GZN1	ACTR6	-4.513	-4.427	-4.643	-4.480	-4.470	-4.561	0.454	-0.091
Q9P021	CRIPT	-3.574	-3.472	-4.015	-3.520	-3.523	-3.768	0.500	-0.245
P84098	RPL19	4.389	4.304	4.441	4.391	4.347	4.416	0.323	0.069
Q96DI7	SNRNP40	-0.659	-0.926	-0.724	-1.012	-0.792	-0.868	0.736	-0.076
Q8IY26	PLPP6	-2.206	-2.298	-2.217	-2.048	-2.252	-2.132	0.370	0.120
P13804	ETFA	3.042	3.081	3.192	3.114	3.062	3.153	0.215	0.091
Q7L211	ABHD13	-1.860	-2.849	-2.729	-2.439	-2.355	-2.584	0.725	-0.229
Q14247	CTTN	1.209	1.148	1.305	1.245	1.178	1.275	0.152	0.097
Q92905	COPS5	2.729	2.681	2.529	2.533	2.705	2.531	0.086	-0.174
Q8TDD1	DDX54	-4.136	-3.928	-4.069	-3.822	-4.032	-3.946	0.646	0.087
O75915	ARL6IP5	1.315	1.327	1.454	1.327	1.321	1.391	0.468	0.070
P15408	FOSL2	-3.160	-3.152	-2.967	-2.925	-3.156	-2.946	0.057	0.210
Q9GZQ3	COMMD5	-0.568	-0.561	-0.507	-0.577	-0.564	-0.542	0.643	0.022
Q5T5U3	ARHGAP21	0.067	0.103	-0.106	-0.085	0.085	-0.095	0.023	-0.180
O95400	CD2BP2	1.854	1.661	1.844	1.759	1.757	1.802	0.730	0.044
Q12834	CDC20	-0.217	-0.178	-0.301	-0.275	-0.198	-0.288	0.073	-0.090
P03928	MT	0.057	0.052	-0.006	0.055	0.055	0.025	0.506	-0.030
Q14571	ITPR2	0.570	0.724	0.576	0.644	0.647	0.610	0.716	-0.037
Q99958	FOXC2	-4.721	-4.643	-4.885	-4.778	-4.682	-4.832	0.165	-0.149
Q99848	EBNA1BP2	3.711	3.648	3.768	3.752	3.680	3.760	0.222	0.080
P62241	RPS8	6.293	6.278	6.250	6.261	6.286	6.255	0.091	-0.030
Q9NXE8	CWC25	0.213	0.117	0.162	0.064	0.165	0.113	0.524	-0.053
P62424	RPL7A	6.369	6.284	6.447	6.370	6.326	6.408	0.288	0.082
Q8WV41	SNX33	-0.786	-0.869	-0.775	-0.833	-0.827	-0.804	0.687	0.024
Q9UHB7	AFF4	-0.308	-0.421	-0.454	-0.404	-0.365	-0.429	0.447	-0.064
Q92968	PEX13	-0.370	-0.495	-0.283	-0.343	-0.432	-0.313	0.273	0.119
O43505	B4GAT1	-1.042	-1.064	-1.296	-1.234	-1.053	-1.265	0.064	-0.212
P20393	NR1D1	-2.296	-2.261	-2.448	-2.383	-2.278	-2.415	0.096	-0.137
Q9NVP1	DDX18	4.576	4.644	4.627	4.611	4.610	4.619	0.836	0.009
Q9Y6B7	AP4B1	-1.068	-1.095	-1.110	-1.140	-1.082	-1.125	0.165	-0.044
P55199	ELL	0.611	0.561	0.511	0.472	0.586	0.491	0.103	-0.095
P27338	MAOB	-2.786	-2.138	-2.651	-2.402	-2.462	-2.526	0.878	-0.064
Q9NX01	TXNL4B	0.153	0.057	0.313	0.302	0.105	0.307	0.144	0.202
Q9P2K6	KLHL42	-0.839	-0.751	-0.938	-0.740	-0.795	-0.839	0.736	-0.045

Q5K651	SAMD9	0.349	0.715	0.655	0.698	0.532	0.677	0.574	0.145
P09429	HMGB1	4.239	4.338	4.212	4.168	4.289	4.190	0.263	-0.099
O95777	LSM8	1.151	1.078	1.274	1.222	1.115	1.248	0.109	0.134
Q13643	FHL3	-0.328	-0.250	-0.264	-0.287	-0.289	-0.275	0.788	0.014
Q15286	RAB35	1.602	1.643	1.714	1.684	1.623	1.699	0.103	0.076
Q2TAY7	SMU1	2.349	2.333	2.424	2.419	2.341	2.422	0.043	0.081
Q5SR56	MFSD14B	-3.755	-3.737	-3.824	-3.458	-3.746	-3.641	0.667	0.105
P17655	CAPN2	2.243	2.062	2.281	2.213	2.153	2.247	0.479	0.094
Q9UBV7	B4GALT7	-1.546	-1.581	-1.743	-1.686	-1.563	-1.714	0.066	-0.151
P10109	FDX1	0.142	0.127	0.010	0.016	0.135	0.013	0.020	-0.122
Q93034	CUL5	3.316	3.240	3.316	3.290	3.278	3.303	0.628	0.025
P50579	METAP2	-2.591	-2.647	-2.584	-2.635	-2.619	-2.609	0.822	0.010
P35568	IRS1	1.500	1.478	1.422	1.464	1.489	1.443	0.226	-0.046
Q96LW7	CARD19	-6.768	-7.098	-6.821	-6.870	-6.933	-6.846	0.689	0.087
Q9P0I2	EMC3	-2.483	-2.680	-2.151	-2.445	-2.582	-2.298	0.267	0.284
Q13485	SMAD4	0.672	0.567	0.519	0.482	0.619	0.500	0.237	-0.119
P42356	PI4KA	1.290	1.202	1.201	1.111	1.246	1.156	0.289	-0.090
O00757	FBP2	-5.126	-4.390	-4.654	-4.873	-4.758	-4.764	0.991	-0.006
O43426	SYNJ1	-2.599	-2.750	-2.656	-2.700	-2.675	-2.678	0.970	-0.004
O95140	MFN2	0.245	0.219	0.342	0.364	0.232	0.353	0.021	0.121
Q8WZ82	OVCA2	0.554	0.481	0.403	0.434	0.517	0.418	0.190	-0.099
P61968	LMO4	-2.967	-3.003	-2.742	-2.685	-2.985	-2.714	0.025	0.271
P61313	RPL15	3.848	3.720	3.886	3.779	3.784	3.832	0.624	0.048
Q9NR45	NANS	4.134	4.128	4.236	4.208	4.131	4.222	0.084	0.092
Q9Y6X8	ZHX2	-0.322	-0.125	-0.331	-0.112	-0.223	-0.222	0.992	0.002
O14828	SCAMP3	-0.440	-0.657	-0.638	-0.839	-0.548	-0.738	0.330	-0.190
Q6UB35	MTHFD1L	3.353	3.402	3.382	3.346	3.377	3.364	0.712	-0.013
Q8IZT6	ASPM	-1.699	-1.705	-2.018	-1.882	-1.702	-1.950	0.170	-0.248
O75396	SEC22B	2.346	2.247	2.253	2.273	2.296	2.263	0.621	-0.033
Q9Y6J0	CABIN1	-6.044	-5.905	-6.051	-5.751	-5.975	-5.901	0.715	0.073
P08758	ANXA5	4.450	4.761	4.769	4.698	4.605	4.734	0.558	0.128
Q9Y263	PLAA	3.860	3.764	3.829	3.756	3.812	3.792	0.778	-0.020
Q7Z4Q2	HEATR3	1.423	1.313	1.463	1.389	1.368	1.426	0.485	0.058
P58317	ZNF121	-1.351	-1.361	-1.334	-1.253	-1.356	-1.294	0.364	0.063
Q9Y3A5	SBDS	4.424	4.346	4.479	4.428	4.385	4.453	0.300	0.068

Q6DKK2	TTC19	0.710	0.533	0.817	0.628	0.622	0.723	0.517	0.101
Q9NYP9	MIS18A	0.064	-0.077	-0.216	-0.379	-0.006	-0.297	0.116	-0.291
Q9NQA3	WASH6P	-2.948	-2.965	-2.826	-2.949	-2.957	-2.887	0.459	0.069
Q86US8	SMG6	0.973	0.938	0.802	0.860	0.956	0.831	0.088	-0.124
Q9Y4K3	TRAF6	-1.582	-1.654	-1.670	-1.564	-1.618	-1.617	0.984	0.002
Q96LD4	TRIM47	-0.510	-0.442	-0.303	-0.365	-0.476	-0.334	0.092	0.142
Q969X1	TMBIM1	-2.351	-2.365	-2.315	-2.266	-2.358	-2.290	0.201	0.068
Q9UNQ2	DIMT1	2.783	2.742	2.739	2.741	2.763	2.740	0.464	-0.023
Q9HCS7	XAB2	2.927	2.886	2.919	2.934	2.906	2.926	0.499	0.020
P98088	MUC5AC	-0.848	-0.887	-0.648	-0.776	-0.867	-0.712	0.227	0.155
Q12996	CSTF3	3.128	3.113	3.137	3.133	3.121	3.135	0.291	0.014
Q9BVC6	TMEM109	2.910	2.843	2.893	2.942	2.877	2.917	0.438	0.041
Q9NVJ2	ARL8B	0.284	0.181	0.282	0.190	0.233	0.236	0.965	0.003
Q96KN1	LRATD2	-0.785	-0.304	-0.433	-0.395	-0.545	-0.414	0.684	0.131
Q03701	CEBPZ	3.899	3.846	3.913	3.925	3.873	3.919	0.322	0.047
P98175	RBM10	-1.906	-1.972	-2.121	-1.938	-1.939	-2.030	0.493	-0.091
Q9NZZ3	CHMP5	-2.025	-2.068	-2.206	-1.907	-2.046	-2.057	0.957	-0.010
O43929	ORC4	-1.517	-1.576	-1.648	-1.921	-1.546	-1.785	0.320	-0.238
Q9UKJ3	GPATCH8	0.142	0.127	0.038	0.108	0.135	0.073	0.322	-0.061
Q96TC7	RMDN3	0.612	0.449	0.652	0.785	0.530	0.718	0.223	0.188
Q14181	POLA2	0.284	0.240	0.015	0.064	0.262	0.039	0.022	-0.223
Q9UJK0	TSR3	-1.750	-1.606	-1.701	-1.412	-1.678	-1.557	0.554	0.121
P53582	METAP1	2.462	2.410	2.442	2.409	2.436	2.425	0.779	-0.010
Q9H3K6	BOLA2	1.707	1.759	1.627	1.658	1.733	1.643	0.119	-0.091
P10620	MGST1	-0.237	-0.288	-0.285	-0.287	-0.262	-0.286	0.525	-0.023
P50454	SERPINH1	5.538	5.494	5.544	5.542	5.516	5.543	0.433	0.027
Q8NHP8	PLBD2	-2.096	-2.109	-2.031	-2.028	-2.102	-2.029	0.044	0.073
Q9NQ88	TIGAR	1.775	1.718	1.749	1.718	1.746	1.734	0.745	-0.013
Q15072	ZNF146	-2.711	-2.657	-2.490	-2.548	-2.684	-2.519	0.053	0.165
O00425	IGF2BP3	5.392	5.296	5.256	5.218	5.344	5.237	0.237	-0.107
Q8IUS5	EPHX4	-3.481	-3.610	-3.588	-3.537	-3.545	-3.562	0.841	-0.017
Q96C28	ZNF707	-4.759	-4.872	-4.853	-4.687	-4.816	-4.770	0.699	0.046
P29084	GTF2E2	2.821	2.768	2.799	2.822	2.795	2.810	0.661	0.016
Q9Y5Z4	HEBP2	-1.592	-1.404	-1.669	-1.620	-1.498	-1.644	0.352	-0.146
Q5QJE6	DNTTIP2	3.779	3.746	3.870	3.838	3.762	3.854	0.058	0.092

P63208	SKP1	1.891	1.360	2.016	1.584	1.626	1.800	0.663	0.174
Q9NVV9	THAP1	-3.713	-3.647	-3.621	-3.747	-3.680	-3.684	0.959	-0.004
P49755	TMED10	3.386	3.233	3.422	3.254	3.310	3.338	0.828	0.028
Q9P219	CCDC88C	-0.938	-0.980	-1.155	-1.010	-0.959	-1.083	0.322	-0.124
Q86WG5	SBF2	-5.358	-5.193	-5.532	-5.566	-5.276	-5.549	0.175	-0.273
P80404	ABAT	-3.371	-3.538	-3.436	-3.565	-3.454	-3.501	0.706	-0.046
Q8WWH5	TRUB1	2.564	2.531	2.497	2.507	2.547	2.502	0.201	-0.045
P26885	FKBP2	1.605	1.540	1.643	1.547	1.572	1.595	0.741	0.022
Q02127	DHODH	2.109	2.092	2.077	2.126	2.101	2.101	0.981	0.001
Q96EH3	MALSU1	-0.140	-0.151	-0.190	-0.147	-0.145	-0.169	0.464	-0.023
Q9UBG0	MRC2	0.642	0.470	0.769	0.748	0.556	0.758	0.252	0.202
Q16531	DDB1	4.946	4.972	4.879	4.880	4.959	4.880	0.105	-0.079
Q15022	SUZ12	1.729	1.649	1.572	1.588	1.689	1.580	0.214	-0.109
P99999	CYCS	5.614	5.652	5.746	5.738	5.633	5.742	0.097	0.108
P51805	PLXNA3	-2.249	-2.408	-2.278	-2.330	-2.329	-2.304	0.812	0.024
Q9NS37	CREBZF	-3.060	-2.929	-3.072	-2.954	-2.995	-3.013	0.855	-0.018
Q9BT92	TCHP	-2.556	-2.565	-2.549	-2.597	-2.560	-2.573	0.691	-0.013
Q96EW2	HSPBAP1	-2.464	-2.512	-2.305	-2.161	-2.488	-2.233	0.149	0.255
O43660	PLRG1	-0.748	-0.819	-0.606	-0.716	-0.783	-0.661	0.224	0.122
Q8IV48	ERI1	2.920	2.852	2.645	2.659	2.886	2.652	0.082	-0.234
P83916	CBX1	2.701	2.567	2.770	2.624	2.634	2.697	0.591	0.063
Q13506	NAB1	-4.218	-3.907	-3.976	-3.968	-4.063	-3.972	0.664	0.091
Q9HBL7	PLGRKT	2.032	1.944	2.075	2.106	1.988	2.090	0.229	0.102
Q8IY81	FTSJ3	3.819	3.833	3.930	3.867	3.826	3.898	0.247	0.072
Q8TF44	C2CD4C	-3.063	-3.192	-3.045	-3.156	-3.128	-3.101	0.780	0.027
Q99829	CPNE1	1.074	1.667	1.394	1.325	1.370	1.360	0.977	-0.011
Q86VW0	SESTD1	-3.613	-3.457	-3.559	-3.375	-3.535	-3.467	0.631	0.068
Q9NPF5	DMAP1	1.335	1.334	1.338	1.324	1.335	1.331	0.665	-0.004
Q8N3C7	CLIP4	-4.584	-4.163	-4.338	-4.020	-4.373	-4.179	0.543	0.194
Q9HB40	SCPEP1	1.191	1.136	1.106	1.112	1.164	1.109	0.293	-0.055
O76071	CIAO1	1.400	1.361	1.342	1.292	1.380	1.317	0.189	-0.064
Q86YS6	RAB43	-0.398	-0.518	-0.208	-0.200	-0.458	-0.204	0.146	0.255
Q9NRH1	YAE1	-3.431	-3.474	-3.595	-3.605	-3.452	-3.600	0.077	-0.147
Q9Y4A0	JRKL	-4.666	-4.215	-4.558	-4.346	-4.441	-4.452	0.969	-0.011
Q9BV19	C1orf50	-0.895	-0.988	-1.030	-0.916	-0.942	-0.973	0.711	-0.032

P29966	MARCKS	0.042	0.712	1.523	1.156	0.377	1.339	0.163	0.962
P50148	GNAQ	-0.853	-0.726	-0.643	-0.622	-0.790	-0.633	0.237	0.157
Q7L2H7	EIF3M	3.637	3.639	3.389	3.439	3.638	3.414	0.070	-0.224
Q9GZY4	COA1	-0.156	-0.214	-0.043	-0.063	-0.185	-0.053	0.108	0.133
P82979	SARNP	3.498	3.406	3.690	3.588	3.452	3.639	0.114	0.187
P62857	RPS28	3.892	3.780	3.837	3.901	3.836	3.869	0.669	0.033
O95373	IPO7	5.173	5.102	5.038	4.983	5.138	5.010	0.113	-0.128
Q9Y6J9	TAF6L	0.124	0.050	0.119	0.074	0.087	0.097	0.845	0.010
Q9NPD3	EXOSC4	1.505	1.441	1.419	1.454	1.473	1.437	0.451	-0.036
O95544	NADK	-4.297	-4.063	-4.155	-4.174	-4.180	-4.165	0.918	0.015
Q9NWW5	CLN6	-4.262	-4.096	-4.221	-4.163	-4.179	-4.192	0.902	-0.013
O76094	SRP72	0.080	0.010	-0.063	-0.053	0.045	-0.058	0.203	-0.103
O60826	CCDC22	1.300	1.362	1.381	1.412	1.331	1.397	0.242	0.066
Q9HCU9	BRMS1	-1.264	-1.339	-1.377	-1.419	-1.301	-1.398	0.186	-0.096
Q8N6S5	ARL6IP6	-0.643	-0.775	-0.614	-0.611	-0.709	-0.613	0.382	0.096
Q8WVC0	LEO1	0.229	0.263	0.119	0.097	0.246	0.108	0.032	-0.138
Q9NWB7	IFT57	-3.757	-3.817	-3.658	-3.744	-3.787	-3.701	0.257	0.086
O15382	BCAT2	0.514	0.402	0.658	0.650	0.458	0.654	0.177	0.196
Q7Z7K0	CMC1	-0.265	-0.302	-0.256	-0.301	-0.284	-0.278	0.868	0.006
O75976	CPD	0.141	0.266	0.124	0.223	0.203	0.174	0.748	-0.030
P48643	CCT5	4.873	4.790	4.886	4.776	4.831	4.831	0.999	0.000
P49441	INPP1	0.155	0.241	0.333	0.300	0.198	0.316	0.187	0.119
Q8IVH4	MMAA	-1.141	-1.121	-0.972	-0.912	-1.131	-0.942	0.075	0.189
Q9HAB8	PPCS	-0.624	-0.559	-0.564	-0.520	-0.592	-0.542	0.345	0.050
Q9BSM1	PCGF1	-2.590	-2.527	-2.611	-2.537	-2.559	-2.574	0.782	-0.015
P36952	SERPINB5	-1.227	-0.072	-0.671	-0.746	-0.650	-0.709	0.935	-0.059
Q9Y2S7	POLDIP2	3.336	3.332	3.283	3.248	3.334	3.266	0.154	-0.069
Q8N183	NDUFAF2	4.006	3.900	4.054	4.003	3.953	4.028	0.369	0.076
Q7LBC6	KDM3B	0.647	0.636	0.513	0.506	0.641	0.510	0.005	-0.131
Q96QT4	TRPM7	-2.913	-3.039	-2.938	-2.920	-2.976	-2.929	0.592	0.047
Q5JU69	TOR2A	-2.488	-2.659	-2.535	-2.559	-2.574	-2.547	0.809	0.027
A8MSI8	LYRM9	-3.459	-3.550	-3.345	-3.370	-3.504	-3.358	0.170	0.147
P49069	CAMLG	0.418	0.357	0.312	0.352	0.388	0.332	0.285	-0.055
Q8TAD8	SNIP1	0.724	0.691	0.764	0.738	0.707	0.751	0.175	0.043
P09884	POLA1	2.096	2.091	1.829	1.862	2.094	1.846	0.038	-0.248

Q86TU7	SETD3	0.822	0.861	0.800	0.780	0.841	0.790	0.183	-0.051
P54136	RARS1	2.389	2.274	2.403	2.279	2.331	2.341	0.920	0.010
Q96F63	CCDC97	-0.224	-0.235	-0.410	-0.401	-0.230	-0.406	0.002	-0.176
Q5HYJ3	FAM76B	-1.746	-2.063	-1.815	-1.960	-1.905	-1.888	0.934	0.017
O14925	TIMM23	1.401	1.324	1.286	1.325	1.363	1.305	0.352	-0.058
Q01105	SET	1.184	1.126	1.164	1.149	1.155	1.156	0.967	0.001
Q8N5C6	SRBD1	0.768	0.785	0.637	0.609	0.777	0.623	0.020	-0.154
Q9BSV6	TSEN34	0.240	0.277	0.037	0.149	0.259	0.093	0.182	-0.165
Q86SE9	PCGF5	-4.399	-4.694	-4.658	-4.750	-4.547	-4.704	0.472	-0.157
P0DPB6	POLR1D	0.224	0.214	0.274	0.240	0.219	0.257	0.246	0.038
Q9NV35	NUDT15	-0.913	-1.081	-1.103	-1.155	-0.997	-1.129	0.346	-0.132
Q9NWS0	PIH1D1	1.090	1.035	0.959	0.987	1.063	0.973	0.140	-0.090
Q9NRY2	INIP	-2.234	-2.211	-2.467	-2.283	-2.223	-2.375	0.343	-0.152
Q969Z0	TBRG4	1.857	1.883	1.732	1.842	1.870	1.787	0.360	-0.083
Q06124	PTPN11	-2.416	-2.510	-2.500	-2.478	-2.463	-2.489	0.676	-0.026
Q8N3R9	PALS1	-3.378	-3.192	-3.447	-3.208	-3.285	-3.327	0.806	-0.043
O43592	XPOT	3.455	3.494	3.437	3.506	3.474	3.472	0.957	-0.002
Q8N1F8	STK11IP	0.252	0.217	0.318	0.256	0.235	0.287	0.310	0.052
Q9H4M9	EHD1	3.714	3.730	3.740	3.633	3.722	3.686	0.623	-0.036
Q96LX8	ZNF597	-3.276	-3.455	-3.131	-3.411	-3.366	-3.271	0.635	0.095
Q32P41	TRMT5	2.414	2.463	2.289	2.484	2.439	2.387	0.689	-0.052
Q9UL25	RAB21	1.925	2.032	1.941	1.905	1.979	1.923	0.479	-0.056
O75475	PSIP1	3.366	3.252	3.225	3.141	3.309	3.183	0.228	-0.126
Q9BV20	MRI1	1.124	1.044	1.015	1.062	1.084	1.039	0.449	-0.045
Q5H9R4	ARMCX4	-6.819	-6.595	-6.539	-6.553	-6.707	-6.546	0.386	0.161
O00212	RHOD	-1.670	-1.598	-1.577	-1.532	-1.634	-1.554	0.226	0.080
O43502	RAD51C	-2.488	-2.965	-2.811	-3.210	-2.726	-3.010	0.460	-0.284
P39687	ANP32A	3.333	3.435	3.427	3.377	3.384	3.402	0.788	0.018
P46778	RPL21	4.089	4.091	4.130	4.146	4.090	4.138	0.102	0.048
Q15032	R3HDM1	-3.164	-3.274	-3.522	-3.433	-3.219	-3.478	0.072	-0.258
O60287	URB1	3.171	3.114	3.149	3.144	3.143	3.146	0.919	0.004
Q08170	SRSF4	3.636	3.518	3.534	3.602	3.577	3.568	0.912	-0.009
O60306	AQR	2.954	2.963	2.854	2.886	2.959	2.870	0.094	-0.089
Q9NRX2	MRPL17	2.954	2.946	2.902	2.929	2.950	2.916	0.214	-0.034
Q9P031	CCDC59	1.212	1.107	1.160	1.173	1.159	1.167	0.913	0.007

Q06945	SOX4	-8.004	-7.302	-7.803	-7.993	-7.653	-7.898	0.611	-0.245
Q6UWZ7	ABRAXAS1	-2.210	-2.294	-2.377	-2.350	-2.252	-2.363	0.203	-0.112
Q9P275	USP36	2.701	2.594	2.788	2.752	2.648	2.770	0.239	0.122
P62877	RBX1	0.692	0.661	0.529	0.563	0.676	0.546	0.030	-0.130
Q9Y5Y6	ST14	-2.414	-2.054	-2.511	-2.320	-2.234	-2.416	0.491	-0.182
Q6PH81	C16orf87	-2.257	-2.323	-2.288	-2.377	-2.290	-2.333	0.528	-0.042
P06733	ENO1	7.214	7.138	7.224	7.171	7.176	7.197	0.698	0.021
O00487	PSMD14	3.749	3.812	3.664	3.708	3.781	3.686	0.145	-0.094
Q96CP2	FLYWCH2	2.709	2.669	2.752	2.829	2.689	2.790	0.186	0.101
Q9Y6W3	CAPN7	0.843	0.750	0.722	0.758	0.796	0.740	0.420	-0.057
Q6XZF7	DNMBP	2.197	2.153	1.829	1.857	2.175	1.843	0.011	-0.332
P23381	WARS1	3.775	3.739	4.084	4.108	3.757	4.096	0.007	0.339
Q9UJF2	RASAL2	-3.645	-3.596	-2.997	-2.853	-3.620	-2.925	0.045	0.695
Q9NS98	SEMA3G	-5.158	-5.295	-5.207	-5.073	-5.226	-5.140	0.463	0.086
P24928	POLR2A	3.829	3.767	3.808	3.773	3.798	3.791	0.858	-0.008
P11717	IGF2R	4.673	4.588	4.832	4.826	4.631	4.829	0.133	0.198
Q13576	IQGAP2	1.081	1.008	0.452	0.483	1.044	0.467	0.019	-0.577
Q9H1Y0	ATG5	-0.470	-0.439	-0.595	-0.600	-0.454	-0.598	0.060	-0.143
Q9UGT4	SUSD2	-4.532	-4.374	-4.434	-4.463	-4.453	-4.448	0.965	0.004
P17812	CTPS1	3.600	3.553	3.464	3.498	3.576	3.481	0.090	-0.095
Q9Y4G6	TLN2	-1.988	-2.000	-2.059	-1.871	-1.994	-1.965	0.811	0.029
P09972	ALDOC	1.688	1.751	1.711	1.719	1.719	1.715	0.912	-0.004
P08779	KRT16	-2.222	-2.193	-3.306	-3.261	-2.208	-3.283	0.002	-1.076
Q8WXX5	DNAJC9	3.805	3.806	3.736	3.712	3.806	3.724	0.091	-0.082
Q16186	ADRM1	2.031	2.006	1.998	1.977	2.018	1.988	0.206	-0.031
Q96EG3	ZNF837	-5.545	-5.507	-5.584	-5.403	-5.526	-5.494	0.781	0.033
Q9NUD5	ZCCHC3	-1.718	-1.772	-1.744	-1.769	-1.745	-1.756	0.752	-0.011
Q8TD57	DNAH3	-4.449	-4.343	-4.143	-4.058	-4.396	-4.101	0.053	0.295
P24592	IGFBP6	-0.945	-1.107	-1.353	-1.342	-1.026	-1.347	0.156	-0.321
Q96EM0	L3HYPDH	-0.892	-0.890	-1.137	-1.087	-0.891	-1.112	0.071	-0.221
O14562	UBFD1	2.421	2.314	2.283	2.284	2.368	2.283	0.358	-0.084
O95989	NUDT3	1.851	1.656	1.910	1.776	1.754	1.843	0.538	0.090
Q5U5Q3	MEX3C	-2.947	-3.046	-3.088	-2.980	-2.997	-3.034	0.662	-0.037
Q9BWT6	MND1	-1.086	-1.011	-1.249	-1.206	-1.048	-1.227	0.078	-0.179
P19075	TSPAN8	-2.833	-2.468	-2.906	-2.941	-2.650	-2.924	0.373	-0.274

Q9BW85	YJU2	0.146	0.134	0.120	0.158	0.140	0.139	0.955	-0.001
P42694	HELZ	-1.337	-1.361	-1.508	-1.441	-1.349	-1.474	0.137	-0.125
Q3MII6	TBC1D25	-3.125	-3.215	-3.077	-3.138	-3.170	-3.107	0.385	0.063
O43752	STX6	0.667	0.723	0.579	0.732	0.695	0.656	0.701	-0.039
O60930	RNASEH1	-0.379	-0.507	-0.402	-0.387	-0.443	-0.395	0.588	0.048
Q14573	ITPR3	4.737	4.726	4.837	4.814	4.732	4.826	0.035	0.094
O43719	HTATSF1	3.228	3.234	3.118	3.116	3.231	3.117	0.006	-0.114
P20338	RAB4A	-0.111	-0.191	-0.502	-0.363	-0.151	-0.433	0.099	-0.282
Q8TED0	UTP15	0.708	0.845	0.800	0.911	0.776	0.855	0.466	0.079
P42765	ACAA2	4.604	4.629	4.816	4.804	4.617	4.810	0.016	0.194
Q9NXC5	MIOS	0.574	0.438	0.626	0.512	0.506	0.569	0.552	0.063
O60911	CTSV	-2.775	-2.870	-3.089	-3.065	-2.823	-3.077	0.101	-0.254
O94886	TMEM63A	-0.484	-0.299	-0.367	-0.197	-0.392	-0.282	0.475	0.110
Q9Y450	HBS1L	-2.176	-2.156	-2.174	-2.168	-2.166	-2.171	0.714	-0.005
Q92535	PIGC	-4.270	-4.240	-4.236	-4.028	-4.255	-4.132	0.445	0.123
Q9BUT9	MCRIP2	-4.142	-4.059	-3.045	-3.097	-4.101	-3.071	0.005	1.030
Q9UL63	MKLN1	-2.251	-2.577	-2.320	-2.435	-2.414	-2.377	0.861	0.037
Q96S66	CLCC1	-0.248	-0.326	-0.434	-0.420	-0.287	-0.427	0.163	-0.140
P50336	PPOX	0.867	0.761	0.987	1.005	0.814	0.996	0.171	0.182
Q12905	ILF2	5.623	5.522	5.617	5.565	5.572	5.591	0.781	0.019
Q96KQ7	EHMT2	-3.831	-3.813	-3.967	-3.976	-3.822	-3.971	0.013	-0.149
O94903	PLPBP	3.816	3.881	3.845	3.836	3.849	3.840	0.840	-0.008
Q8WYA0	IFT81	-1.073	-1.137	-1.144	-1.127	-1.105	-1.136	0.508	-0.031
P34931	HSPA1L	-1.538	-1.588	-1.481	-1.441	-1.563	-1.461	0.090	0.102
Q6ZW49	PAXIP1	-2.182	-1.918	-2.221	-1.937	-2.050	-2.079	0.896	-0.029
O15550	KDM6A	-2.304	-2.086	-2.226	-2.024	-2.195	-2.125	0.684	0.070
Q9H7B4	SMYD3	-1.744	-1.958	-2.019	-2.113	-1.851	-2.066	0.262	-0.215
P07305	H1	-3.488	-3.354	-3.178	-3.001	-3.421	-3.089	0.105	0.332
O75764	TCEA3	-4.420	-4.292	-4.324	-4.396	-4.356	-4.360	0.963	-0.004
Q6UW78	UQCC3	-1.376	-1.343	-1.269	-1.384	-1.359	-1.326	0.668	0.033
Q86T24	ZBTB33	-1.275	-1.344	-1.274	-1.320	-1.310	-1.297	0.795	0.012
P22681	CBL	2.086	2.005	2.055	2.086	2.046	2.070	0.653	0.025
Q9NPI8	FANCF	-4.090	-4.229	-4.146	-4.011	-4.159	-4.078	0.492	0.081
Q9BTE3	MCMBP	-2.809	-2.802	-2.930	-2.880	-2.805	-2.905	0.152	-0.099
Q14119	VEZF1	0.130	0.099	0.174	0.121	0.115	0.148	0.414	0.033

Q9H8H3	METTL7A	-5.459	-5.258	-5.228	-5.401	-5.358	-5.314	0.772	0.044
Q9BY42	RTF2	0.862	1.032	0.935	1.045	0.947	0.990	0.717	0.043
Q01082	SPTBN1	0.554	0.786	0.657	0.605	0.670	0.631	0.795	-0.039
Q7L1T6	CYB5R4	-0.901	-0.945	-0.803	-0.860	-0.923	-0.831	0.134	0.092
Q9GZR7	DDX24	-1.575	-1.589	-1.540	-1.619	-1.582	-1.580	0.967	0.002
O94973	AP2A2	-2.497	-2.540	-2.525	-2.399	-2.519	-2.462	0.527	0.057
Q8N5K1	CISD2	1.878	1.918	1.960	2.005	1.898	1.982	0.109	0.085
Q9Y2R0	COA3	0.951	0.961	0.926	0.706	0.956	0.816	0.423	-0.140
Q9H7P6	MVB12B	-7.089	-6.562	-6.639	-6.692	-6.826	-6.666	0.652	0.160
P62979	RPS27A	3.265	3.352	3.354	3.380	3.309	3.367	0.398	0.058
Q7Z4R8	C6orf120	-2.231	-2.317	-2.231	-2.296	-2.274	-2.263	0.861	0.011
Q96EQ0	SGTB	-0.429	-0.640	-0.281	-0.327	-0.535	-0.304	0.261	0.231
Q14697	GANAB	0.147	0.176	0.040	0.108	0.161	0.074	0.198	-0.087
Q15061	WDR43	3.445	3.395	3.446	3.478	3.420	3.462	0.312	0.042
O43776	NARS1	5.688	5.577	5.651	5.618	5.633	5.634	0.979	0.002
Q9UK39	NOCT	-1.823	-1.894	-1.984	-1.830	-1.859	-1.907	0.645	-0.048
P58107	EPPK1	2.444	2.963	2.628	2.645	2.704	2.637	0.839	-0.067
P26583	HMGB2	5.567	5.617	5.697	5.637	5.592	5.667	0.197	0.075
Q8NHG8	ZNRF2	-0.900	-0.974	-0.895	-0.963	-0.937	-0.929	0.886	0.008
O94885	SASH1	-2.778	-2.722	-2.552	-2.524	-2.750	-2.538	0.043	0.212
Q9Y5J7	TIMM9	1.166	1.101	0.760	0.887	1.134	0.824	0.080	-0.310
P22087	FBL	5.021	5.017	5.078	5.103	5.019	5.090	0.102	0.072
Q86Y13	DZIP3	-2.332	-2.391	-2.330	-2.285	-2.361	-2.307	0.293	0.054
P06400	RB1	2.692	2.793	2.527	2.561	2.743	2.544	0.131	-0.199
Q32MZ4	LRRFIP1	1.103	1.053	1.289	1.061	1.078	1.175	0.549	0.097
Q9BQP7	MGME1	1.204	1.286	1.161	1.151	1.245	1.156	0.271	-0.089
Q9Y6R0	NUMBL	1.293	1.222	1.406	1.387	1.258	1.396	0.143	0.139
Q92747	ARPC1A	-0.494	-0.474	-0.394	-0.541	-0.484	-0.468	0.859	0.017
Q96FQ6	S100A16	0.515	0.418	0.364	0.136	0.466	0.250	0.278	-0.217
P61020	RAB5B	-0.827	-0.823	-0.712	-0.603	-0.825	-0.658	0.200	0.168
Q96HY7	DHTKD1	3.375	3.382	3.342	3.311	3.379	3.327	0.166	-0.052
Q86X02	CDR2L	-1.337	-1.430	-1.338	-1.312	-1.383	-1.325	0.418	0.058
Q14137	BOP1	-0.569	-0.474	-0.706	-0.451	-0.521	-0.579	0.733	-0.058
Q7Z478	DHX29	3.529	3.440	3.360	3.318	3.484	3.339	0.144	-0.145
P50238	CRIP1	-2.213	-1.475	-1.920	-1.558	-1.844	-1.739	0.829	0.105

P55809	OXCT1	3.247	3.251	3.165	3.197	3.249	3.181	0.142	-0.068
Q13217	DNAJC3	2.815	2.842	2.891	2.871	2.829	2.881	0.100	0.052
Q99623	PHB2	2.470	2.396	2.607	2.610	2.433	2.609	0.132	0.176
Q8N3Z6	ZCCHC7	-2.133	-2.071	-2.074	-1.970	-2.102	-2.022	0.339	0.080
Q92698	RAD54L	-1.606	-1.527	-1.812	-1.601	-1.567	-1.707	0.396	-0.140
Q96DA6	DNAJC19	-1.597	-1.659	-1.554	-1.621	-1.628	-1.587	0.469	0.041
Q9BR61	ACBD6	1.017	0.902	0.848	0.770	0.960	0.809	0.179	-0.151
Q15059	BRD3	-0.570	-0.520	-0.488	-0.582	-0.545	-0.535	0.874	0.010
Q9NX20	MRPL16	2.468	2.479	2.437	2.498	2.473	2.468	0.882	-0.006
P05129	PRKCG	-4.553	-4.627	-4.708	-4.636	-4.590	-4.672	0.253	-0.082
P41162	ETV3	-2.114	-2.126	-2.110	-1.974	-2.120	-2.042	0.458	0.078
Q9Y3A2	UTP11	1.473	1.462	1.488	1.495	1.467	1.491	0.090	0.024
P49915	GMPS	-0.045	0.072	-0.349	-0.134	0.014	-0.241	0.209	-0.255
P40222	TXLNA	3.673	3.592	3.601	3.592	3.632	3.597	0.540	-0.036
Q9BUM1	G6PC3	-3.110	-3.232	-2.983	-3.132	-3.171	-3.058	0.364	0.114
Q9NX24	NHP2	1.659	1.732	1.784	1.834	1.695	1.809	0.140	0.114
Q9HBM6	TAF9B	-0.684	-0.664	-0.807	-0.814	-0.674	-0.810	0.028	-0.136
Q69YN4	VIRMA	-1.951	-2.246	-2.039	-2.224	-2.098	-2.132	0.869	-0.033
Q9NUA8	ZBTB40	-1.879	-1.972	-2.017	-2.051	-1.925	-2.034	0.229	-0.109
Q9BZK7	TBL1XR1	1.730	1.670	1.642	1.563	1.700	1.602	0.196	-0.097
Q1ED39	KNOP1	0.560	0.571	0.514	0.641	0.565	0.577	0.882	0.012
Q13362	PPP2R5C	-2.120	-1.983	-2.057	-2.047	-2.052	-2.052	0.999	0.000
Q96A35	MRPL24	2.374	2.278	2.279	2.342	2.326	2.311	0.820	-0.015
P82932	MRPS6	1.913	1.809	1.785	1.829	1.861	1.807	0.474	-0.055
O75146	HIP1R	0.115	0.175	0.159	0.287	0.145	0.223	0.424	0.078
Q7Z2T5	TRMT1L	1.583	1.503	1.623	1.621	1.543	1.622	0.298	0.079
Q15035	TRAM2	-2.334	-2.417	-2.473	-2.468	-2.376	-2.470	0.262	-0.095
Q13395	TARBP1	-0.959	-1.050	-1.038	-1.023	-1.004	-1.030	0.666	-0.026
Q53S33	BOLA3	0.687	0.620	0.573	0.581	0.654	0.577	0.259	-0.077
Q8NFU5	IPMK	-2.952	-3.026	-3.112	-3.078	-2.989	-3.095	0.171	-0.106
Q9P1U0	POLR1H	-1.183	-1.208	-1.248	-1.077	-1.196	-1.162	0.762	0.034
Q9NVE7	PANK4	2.733	2.720	2.618	2.652	2.726	2.635	0.083	-0.091
P51116	FXR2	2.779	2.727	2.858	2.869	2.753	2.864	0.136	0.110
Q9Y4W6	AFG3L2	5.160	5.050	5.239	5.220	5.105	5.230	0.256	0.125
P31930	UQCRC1	4.621	4.682	4.627	4.629	4.651	4.628	0.579	-0.023

Q63HN8	RNF213	-5.955	-5.873	-5.748	-5.873	-5.914	-5.810	0.318	0.104
Q6ZWJ1	STXBP4	-1.363	-1.551	-1.501	-1.696	-1.457	-1.598	0.407	-0.141
P49458	SRP9	3.225	3.257	3.121	3.079	3.241	3.100	0.037	-0.141
Q9NVP2	ASF1B	-1.656	-1.631	-1.791	-1.897	-1.643	-1.844	0.149	-0.200
Q8N465	D2HGDH	-1.395	-1.557	-1.519	-1.580	-1.476	-1.549	0.524	-0.073
Q92930	RAB8B	-3.034	-3.277	-3.069	-3.316	-3.156	-3.192	0.852	-0.037
Q9P266	JCAD	-1.927	-2.040	-2.005	-1.999	-1.983	-2.002	0.796	-0.019
Q8TAT5	NEIL3	-3.895	-4.020	-4.015	-4.036	-3.958	-4.026	0.470	-0.068
Q96EL2	MRPS24	-0.319	-0.456	-0.404	-0.508	-0.387	-0.456	0.511	-0.069
P35232	PHB	5.825	5.697	5.938	5.871	5.761	5.904	0.226	0.143
Q14974	KPNB1	2.626	2.507	2.517	2.517	2.567	2.517	0.556	-0.050
Q8NB91	FANCB	-1.150	-1.237	-1.337	-1.336	-1.193	-1.336	0.188	-0.143
Q07864	POLE	1.808	1.810	1.491	1.500	1.809	1.496	0.006	-0.313
A8MVW0	FAM171A2	-2.938	-3.044	-3.256	-3.082	-2.991	-3.169	0.249	-0.177
Q14353	GAMT	-1.208	-1.340	-1.513	-1.472	-1.274	-1.493	0.162	-0.219
P80723	BASP1	-3.200	-3.556	-2.884	-3.491	-3.378	-3.188	0.653	0.191
Q05DH4	FHIP1A	-2.686	-2.612	-2.657	-2.579	-2.649	-2.618	0.622	0.031
P53396	ACLY	0.948	0.868	0.871	0.689	0.908	0.780	0.374	-0.128
Q15651	HMGN3	-1.441	-1.449	-1.607	-1.560	-1.445	-1.583	0.098	-0.138
A8MQ03	CYSRT1	-3.219	-3.334	-3.279	-3.355	-3.276	-3.317	0.626	-0.040
P53801	PTTG1IP	-0.110	-0.113	0.071	0.056	-0.111	0.064	0.023	0.175
Q92541	RTF1	3.576	3.522	3.569	3.557	3.549	3.563	0.687	0.014
Q14493	SLBP	-1.592	-1.594	-1.542	-1.608	-1.593	-1.575	0.680	0.018
P42338	PIK3CB	1.113	0.955	1.148	0.972	1.034	1.060	0.844	0.026
P12109	COL6A1	0.271	0.230	0.421	0.539	0.251	0.480	0.131	0.230
Q9HCM7	FBRSL1	-3.287	-3.363	-3.269	-3.218	-3.325	-3.244	0.238	0.081
Q96SB3	PPP1R9B	2.211	2.067	2.015	1.956	2.139	1.986	0.248	-0.154
P04264	KRT1	2.826	2.771	2.076	2.084	2.799	2.080	0.021	-0.719
Q12824	SMARCB1	-3.023	-2.562	-2.970	-2.809	-2.792	-2.889	0.748	-0.097
Q92844	TANK	-0.465	-0.418	-0.379	-0.292	-0.441	-0.335	0.203	0.106
Q86W34	AMZ2	-1.337	-1.525	-1.318	-1.411	-1.431	-1.364	0.609	0.067
Q9GZT4	SRR	1.300	1.117	1.017	0.959	1.209	0.988	0.228	-0.221
O60499	STX10	0.140	-0.043	0.046	-0.144	0.048	-0.049	0.538	-0.097
Q9Y3E2	BOLA1	-2.803	-2.517	-2.189	-1.834	-2.660	-2.011	0.110	0.648
Q8TAK5	GABPB2	-1.996	-2.022	-2.079	-2.001	-2.009	-2.040	0.564	-0.032

Q01813	PFKP	0.543	0.471	0.537	0.460	0.507	0.499	0.885	-0.009
P98160	HSPG2	-1.815	-1.860	-1.901	-1.823	-1.837	-1.862	0.647	-0.025
Q8IWF6	DENND6A	-0.931	-1.031	-0.994	-1.032	-0.981	-1.013	0.638	-0.032
Q9NRM7	LATS2	-3.097	-3.266	-3.184	-3.234	-3.182	-3.209	0.799	-0.028
P21953	BCKDHB	2.156	2.142	2.104	2.138	2.149	2.121	0.333	-0.028
Q8WUK0	PTPMT1	0.487	0.331	0.288	0.237	0.409	0.262	0.291	-0.146
O15287	FANCG	-2.494	-2.580	-2.851	-2.766	-2.537	-2.808	0.046	-0.271
Q8IZM8	ZNF654	-1.991	-2.109	-2.064	-2.021	-2.050	-2.042	0.920	0.008
Q9Y5Q0	FADS3	-3.216	-3.258	-2.824	-2.888	-3.237	-2.856	0.016	0.380
O15355	PPM1G	4.775	4.786	4.724	4.717	4.781	4.721	0.019	-0.060
Q9NZN4	EHD2	-0.329	-0.176	-0.534	-0.396	-0.253	-0.465	0.178	-0.212
O94915	FRYL	2.366	2.281	2.301	2.259	2.323	2.280	0.488	-0.043
Q9P0B6	CCDC167	-1.217	-1.244	-1.401	-1.202	-1.231	-1.301	0.606	-0.071
Q9C0D3	ZYG11B	-0.867	-1.031	-0.916	-0.812	-0.949	-0.864	0.488	0.085
Q8NCJ5	SPRYD3	1.324	1.436	1.269	1.409	1.380	1.339	0.696	-0.041
Q9UG01	IFT172	-2.782	-2.823	-2.899	-2.805	-2.803	-2.852	0.478	-0.049
P35610	SOAT1	1.995	1.785	1.876	1.882	1.890	1.879	0.933	-0.011
O75845	SC5D	-1.637	-1.622	-1.392	-1.447	-1.629	-1.419	0.065	0.210
O43716	GATC	-2.804	-2.740	-2.870	-2.857	-2.772	-2.863	0.203	-0.091
Q8N2A8	PLD6	-6.626	-6.607	-6.636	-6.506	-6.617	-6.571	0.608	0.046
Q69YH5	CDCA2	0.390	0.309	0.225	0.227	0.350	0.226	0.202	-0.124
Q9BXL8	CDCA4	-3.806	-3.625	-3.852	-3.325	-3.716	-3.588	0.715	0.127
P18077	RPL35A	5.448	5.346	5.414	5.373	5.397	5.394	0.962	-0.003
Q96FN9	DTD2	-1.441	-1.386	-1.537	-1.290	-1.414	-1.413	0.998	0.000
Q8IWT6	LRRC8A	0.599	0.613	0.820	0.756	0.606	0.788	0.097	0.182
Q9BVW5	TIPIN	-1.637	-1.761	-2.024	-1.949	-1.699	-1.986	0.079	-0.287
O00519	FAAH	-1.758	-1.349	-1.525	-1.606	-1.553	-1.565	0.963	-0.012
Q99417	MYCBP	1.365	1.243	1.172	1.054	1.304	1.113	0.153	-0.191
Q9NXF8	ZDHHC7	-3.182	-3.140	-3.386	-3.345	-3.161	-3.366	0.020	-0.205
Q8TBZ6	TRMT10A	-1.999	-1.830	-1.697	-1.630	-1.915	-1.663	0.171	0.251
Q16576	RBBP7	-1.873	-1.990	-1.744	-1.940	-1.931	-1.842	0.531	0.090
Q9HCG1	ZNF160	-3.307	-3.301	-3.325	-3.265	-3.304	-3.295	0.819	0.009
Q8WV60	PTCD2	-0.428	-0.554	-0.550	-0.607	-0.491	-0.579	0.377	-0.087
P07942	LAMB1	1.223	1.277	0.903	0.957	1.250	0.930	0.014	-0.320
Q15691	MAPRE1	4.034	4.082	4.016	3.944	4.058	3.980	0.231	-0.078

P31153	MAT2A	3.488	3.441	3.497	3.431	3.464	3.464	0.991	-0.001
P58004	SESN2	0.581	0.514	0.570	0.609	0.548	0.590	0.417	0.042
Q53H96	PYCR3	-3.415	-3.292	-3.440	-3.396	-3.353	-3.418	0.475	-0.065
P62333	PSMC6	4.335	4.267	4.375	4.305	4.301	4.340	0.510	0.039
P62987	UBA52	1.648	1.626	1.518	1.490	1.637	1.504	0.022	-0.133
Q9BSK2	SLC25A33	-2.508	-2.648	-2.472	-2.537	-2.578	-2.505	0.474	0.073
Q9NYZ3	GTSE1	-0.372	-0.429	-0.403	-0.443	-0.401	-0.423	0.592	-0.022
Q9Y2T7	YBX2	-1.228	-1.039	-1.166	-1.107	-1.134	-1.136	0.983	-0.003
P47224	RABIF	0.068	0.108	-0.158	-0.020	0.088	-0.089	0.217	-0.177
Q9Y6K0	CEPT1	-0.181	-0.209	-0.174	-0.156	-0.195	-0.165	0.232	0.030
Q6GMV2	SMYD5	2.493	2.419	2.356	2.315	2.456	2.335	0.136	-0.121
Q8N4L2	PIP4P2	-3.435	-3.499	-3.577	-3.515	-3.467	-3.546	0.219	-0.079
Q9UHG0	DCDC2	-3.364	-3.060	-3.518	-3.254	-3.212	-3.386	0.481	-0.174
O14841	OPLAH	0.868	0.797	0.875	0.797	0.833	0.836	0.956	0.003
P82650	MRPS22	2.686	2.787	2.695	2.690	2.737	2.693	0.547	-0.044
Q96NT0	CCDC115	-3.784	-3.961	-4.159	-4.028	-3.872	-4.093	0.193	-0.221
Q9HCU5	PREB	1.982	1.885	2.068	2.042	1.933	2.055	0.223	0.122
Q9H8U3	ZFAND3	-1.733	-1.921	-1.594	-1.790	-1.827	-1.692	0.425	0.135
Q6NXR4	TTI2	1.139	1.122	0.995	1.053	1.131	1.024	0.144	-0.107
Q16719	KYNU	-1.326	-1.257	-0.896	-1.121	-1.292	-1.009	0.219	0.283
Q96PN7	TRERF1	-3.258	-3.050	-3.527	-3.192	-3.154	-3.359	0.425	-0.205
P14174	MIF	2.567	2.281	2.511	2.384	2.424	2.447	0.901	0.023
Q9UPX8	SHANK2	-3.651	-3.213	-3.698	-3.483	-3.432	-3.590	0.603	-0.158
Q9C004	SPRY4	1.379	1.323	1.302	1.418	1.351	1.360	0.908	0.009
P24311	COX7B	-1.106	-1.213	-1.198	-1.292	-1.159	-1.245	0.353	-0.086
Q2TB90	HKDC1	-0.695	-0.318	-0.529	-0.476	-0.506	-0.503	0.988	0.004
P38919	EIF4A3	4.867	4.760	4.889	4.749	4.813	4.819	0.954	0.006
Q9Y3Q8	TSC22D4	-0.713	-0.626	-0.451	-0.532	-0.669	-0.492	0.097	0.177
O95486	SEC24A	1.712	1.653	1.564	1.572	1.683	1.568	0.155	-0.115
Q96I25	RBM17	3.008	2.974	2.914	2.890	2.991	2.902	0.058	-0.089
P62258	YWHAE	6.067	5.988	6.244	6.171	6.028	6.207	0.079	0.180
O95801	TTC4	2.005	1.942	1.891	1.850	1.974	1.870	0.131	-0.103
Q8NAV1	PRPF38A	0.950	0.993	0.997	1.012	0.971	1.004	0.344	0.033
Q6DCA0	AMMECR1L	-2.010	-2.038	-2.016	-1.983	-2.024	-2.000	0.386	0.024
Q17RS7	GEN1	-2.644	-2.493	-2.622	-2.589	-2.569	-2.605	0.714	-0.037

Q9Y4E5	ZNF451	-5.951	-5.839	-5.932	-6.001	-5.895	-5.967	0.411	-0.071
Q9UBT2	UBA2	3.043	3.027	3.056	3.093	3.035	3.074	0.244	0.039
P51970	NDUFA8	3.024	3.057	3.094	3.137	3.041	3.115	0.120	0.074
Q9P0M9	MRPL27	0.517	0.487	0.519	0.487	0.502	0.503	0.950	0.002
Q3ZAQ7	VMA21	-1.666	-1.559	-1.515	-1.428	-1.613	-1.472	0.184	0.141
Q9H299	SH3BGRL3	4.053	4.111	3.975	3.912	4.082	3.944	0.084	-0.138
P61106	RAB14	4.002	4.085	3.893	3.861	4.043	3.877	0.121	-0.166
Q9P2I0	CPSF2	3.499	3.451	3.482	3.464	3.475	3.473	0.962	-0.001
Q9NX18	SDHAF2	0.521	0.458	-0.020	0.038	0.490	0.009	0.008	-0.481
Q13325	IFIT5	-1.605	-1.725	-1.590	-1.593	-1.665	-1.592	0.439	0.073
P48723	HSPA13	1.773	1.651	2.052	1.972	1.712	2.012	0.070	0.300
Q5T653	MRPL2	2.523	2.449	2.529	2.520	2.486	2.524	0.485	0.039
Q6NVY1	HIBCH	2.696	2.617	2.773	2.822	2.657	2.798	0.114	0.141
P19447	ERCC3	1.576	1.593	1.618	1.653	1.585	1.635	0.170	0.051
Q5HYK3	COQ5	3.277	3.197	3.332	3.286	3.237	3.309	0.285	0.072
Q53EU6	GPAT3	2.973	2.949	2.736	2.795	2.961	2.766	0.063	-0.196
Q8N1F7	NUP93	2.549	2.507	2.547	2.586	2.528	2.567	0.307	0.038
A0A2Z4LIS9	FOXO3B	-3.953	-4.215	-4.275	-4.288	-4.084	-4.282	0.371	-0.198
O95292	VAPB	2.054	2.188	2.034	2.014	2.121	2.024	0.383	-0.097
P54259	ATN1	-0.768	-0.878	-0.688	-0.691	-0.823	-0.689	0.249	0.134
O00750	PIK3C2B	-3.073	-3.008	-3.316	-2.935	-3.040	-3.126	0.732	-0.085
Q9NRY5	FAM114A2	0.750	0.621	0.673	0.648	0.685	0.661	0.767	-0.025
Q9Y2V2	CARHSP1	1.762	1.770	1.704	1.700	1.766	1.702	0.016	-0.064
Q5T8A7	PPP1R26	-3.433	-3.615	-3.549	-3.411	-3.524	-3.480	0.740	0.044
Q9NSB2	KRT84	-1.203	-1.306	-1.083	-1.149	-1.255	-1.116	0.173	0.139
O14641	DVL2	1.091	1.063	1.101	1.028	1.077	1.065	0.793	-0.013
P30405	PPIF	2.206	2.145	2.286	2.265	2.175	2.275	0.159	0.100
Q92643	PIGK	-0.449	-0.342	-0.469	-0.450	-0.396	-0.460	0.436	-0.064
Q9H3S4	TPK1	-3.390	-3.511	-3.555	-3.434	-3.451	-3.495	0.656	-0.044
P48047	ATP5PO	4.710	4.590	4.782	4.699	4.650	4.740	0.356	0.090
Q9ULX6	AKAP8L	-1.549	-1.595	-1.488	-1.489	-1.572	-1.489	0.172	0.083
Q6PK04	CCDC137	1.293	1.180	1.207	1.189	1.237	1.198	0.621	-0.038
Q9P032	NDUFAF4	3.861	3.753	3.890	3.852	3.807	3.871	0.432	0.064
P78318	IGBP1	2.079	2.027	2.108	2.057	2.053	2.082	0.504	0.030
Q96KP1	EXOC2	2.417	2.438	2.397	2.429	2.427	2.413	0.550	-0.014

P04004	VTN	0.446	0.277	1.082	0.966	0.361	1.024	0.031	0.663
Q8IV03	LURAP1L	-2.916	-2.836	-2.958	-2.787	-2.876	-2.872	0.976	0.003
Q9H2J4	PDCL3	1.414	1.357	1.312	1.284	1.386	1.298	0.152	-0.088
Q96FS4	SIPA1	0.935	0.838	0.930	0.955	0.887	0.942	0.447	0.056
Q96EY8	MMAB	1.443	1.447	1.550	1.565	1.445	1.558	0.032	0.113
P62140	PPP1CB	1.024	1.025	1.073	1.054	1.024	1.063	0.151	0.039
O14734	ACOT8	0.521	0.352	0.521	0.393	0.437	0.457	0.866	0.021
Q96NW4	ANKRD27	0.511	0.492	0.446	0.487	0.502	0.467	0.308	-0.035
Q96MW1	CCDC43	-1.118	-1.126	-1.088	-1.044	-1.122	-1.066	0.230	0.056
Q5T601	ADGRF1	-2.045	-1.876	-2.131	-1.758	-1.961	-1.945	0.948	0.016
Q9NUS5	AP5S1	-1.675	-1.757	-1.726	-1.617	-1.716	-1.671	0.586	0.044
O96013	PAK4	-1.110	-1.046	-1.027	-0.878	-1.078	-0.952	0.316	0.125
Q9Y256	RCE1	-0.465	-0.459	-0.555	-0.486	-0.462	-0.520	0.340	-0.058
Q96PE7	MCEE	-0.381	-0.340	-0.349	-0.325	-0.361	-0.337	0.447	0.024
Q8WUM0	NUP133	3.603	3.533	3.524	3.504	3.568	3.514	0.348	-0.054
Q9H4A6	GOLPH3	1.482	1.437	1.435	1.413	1.459	1.424	0.344	-0.035
Q7Z3B3	KANSL1	-1.034	-1.107	-1.073	-0.988	-1.070	-1.031	0.551	0.040
Q9P2E3	ZNFX1	0.587	0.591	0.693	0.714	0.589	0.703	0.050	0.114
Q96GC9	VMP1	-1.275	-1.383	-1.118	-1.306	-1.329	-1.212	0.417	0.117
O60507	TPST1	-2.282	-2.490	-2.188	-2.327	-2.386	-2.258	0.426	0.128
Q8WTX7	CASTOR1	-2.025	-2.204	-1.984	-1.905	-2.115	-1.945	0.278	0.170
Q92841	DDX17	0.307	0.315	0.320	0.313	0.311	0.316	0.428	0.005
O14965	AURKA	0.583	0.544	0.495	0.382	0.563	0.439	0.244	-0.125
P58546	MTPN	3.648	3.627	3.554	3.545	3.638	3.549	0.044	-0.088
Q96GQ7	DDX27	4.568	4.503	4.580	4.524	4.536	4.552	0.738	0.016
Q9Y6A5	TACC3	1.030	1.095	0.979	0.963	1.062	0.971	0.203	-0.092
O60669	SLC16A7	-6.147	-6.145	-5.863	-6.204	-6.146	-6.034	0.629	0.112
Q9Y2R5	MRPS17	1.906	1.827	1.748	1.713	1.866	1.730	0.137	-0.136
Q53HC9	EIPR1	1.950	1.904	1.877	1.857	1.927	1.867	0.196	-0.060
O76021	RSL1D1	5.005	5.012	5.106	5.070	5.009	5.088	0.130	0.079
P29597	TYK2	-0.536	-0.584	-0.668	-0.613	-0.560	-0.641	0.159	-0.081
A6NED2	RCCD1	-1.518	-1.463	-1.470	-1.510	-1.490	-1.490	0.991	0.000
P27105	STOM	-3.334	-3.413	-3.153	-3.172	-3.373	-3.163	0.101	0.211
O15067	PFAS	4.219	4.154	4.121	4.118	4.186	4.119	0.286	-0.067
Q15527	SURF2	0.941	0.983	0.927	0.925	0.962	0.926	0.333	-0.036

Q92879	CELF1	-1.598	-1.589	-1.772	-1.671	-1.594	-1.721	0.238	-0.127
O43707	ACTN4	5.062	5.350	4.986	4.878	5.206	4.932	0.282	-0.274
Q9UN81	L1RE1	1.387	1.546	1.480	1.433	1.467	1.457	0.921	-0.010
Q14118	DAG1	1.180	1.070	1.216	1.150	1.125	1.183	0.479	0.058
P40937	RFC5	-3.944	-4.020	-3.999	-4.174	-3.982	-4.087	0.430	-0.105
Q9Y3P9	RABGAP1	2.117	2.090	2.068	1.999	2.103	2.033	0.265	-0.070
Q96HP4	OXNAD1	-0.094	-0.162	-0.057	-0.050	-0.128	-0.054	0.269	0.074
P10588	NR2F6	-0.292	-0.454	-0.230	-0.152	-0.373	-0.191	0.226	0.182
Q12772	SREBF2	-3.221	-3.178	-3.239	-3.147	-3.200	-3.193	0.913	0.007
Q96BY7	ATG2B	0.734	0.711	0.576	0.555	0.723	0.565	0.010	-0.157
Q9NVI1	FANCI	0.602	0.755	0.512	0.481	0.678	0.496	0.242	-0.182
Q9UKI2	CDC42EP3	0.247	0.158	0.141	0.237	0.203	0.189	0.854	-0.014
P24666	ACP1	-7.273	-7.374	-7.647	-7.174	-7.323	-7.410	0.776	-0.087
Q7L592	NDUFAF7	-0.630	-0.704	-0.569	-0.564	-0.667	-0.566	0.224	0.101
Q03518	TAP1	0.747	0.622	0.943	0.906	0.685	0.925	0.139	0.240
P51795	CLCN5	-2.766	-2.881	-3.120	-3.030	-2.824	-3.075	0.082	-0.251
Q9UBS8	RNF14	-2.644	-2.692	-2.720	-2.754	-2.668	-2.737	0.154	-0.069
Q15428	SF3A2	1.865	1.820	1.946	1.877	1.843	1.912	0.254	0.069
Q7Z6M4	MTERF4	0.468	0.380	0.397	0.340	0.424	0.368	0.417	-0.056
Q8NEB9	PIK3C3	3.058	2.984	2.981	2.967	3.021	2.974	0.415	-0.047
Q9BWD1	ACAT2	-1.645	-1.422	-1.710	-1.685	-1.533	-1.697	0.378	-0.164
P05114	HMGN1	3.143	3.058	3.148	3.098	3.100	3.123	0.701	0.023
P17535	JUND	-1.842	-1.926	-1.734	-1.693	-1.884	-1.714	0.108	0.170
Q9Y2Z0	SUGT1	-3.508	-3.479	-3.532	-3.561	-3.493	-3.546	0.124	-0.053
P41223	BUD31	-3.300	-3.518	-3.165	-3.142	-3.409	-3.153	0.254	0.256
O95870	ABHD16A	-2.907	-2.878	-2.839	-2.849	-2.892	-2.844	0.157	0.049
Q6IA17	SIGIRR	-3.614	-3.502	-3.509	-3.525	-3.558	-3.517	0.599	0.041
Q96IX5	ATP5MK	0.305	0.187	0.268	0.217	0.246	0.242	0.961	-0.004
Q9ULF5	SLC39A10	-0.503	-0.567	-0.709	-0.703	-0.535	-0.706	0.115	-0.171
Q9HCN8	SDF2L1	0.154	0.165	0.064	-0.021	0.160	0.022	0.184	-0.138
O75794	CDC123	0.628	0.585	0.693	0.660	0.606	0.677	0.129	0.070
Q8TAM1	BBS10	-4.517	-4.735	-4.576	-4.608	-4.626	-4.592	0.809	0.034
P40261	NNMT	-1.327	-0.697	-0.657	-0.771	-1.012	-0.714	0.515	0.298
Q9BYV8	CEP41	0.122	-0.052	-0.121	-0.262	0.035	-0.191	0.186	-0.226
Q9NVM6	DNAJC17	-0.345	-0.485	-0.317	-0.357	-0.415	-0.337	0.457	0.078

Q86SZ2	TRAPPC6B	-0.445	-0.402	-0.634	-0.558	-0.424	-0.596	0.086	-0.172
Q8N9N2	ASCC1	-0.669	-0.778	-0.646	-0.757	-0.724	-0.702	0.804	0.022
Q9Y259	CHKB	-1.764	-1.785	-1.757	-1.740	-1.774	-1.749	0.201	0.026
Q9H0R4	HDHD2	0.169	0.126	0.094	0.038	0.148	0.066	0.153	-0.082
Q99570	PIK3R4	2.640	2.601	2.612	2.626	2.621	2.619	0.951	-0.002
A0FGR8	ESYT2	-2.523	-2.725	-2.660	-2.718	-2.624	-2.689	0.637	-0.065
O43772	SLC25A20	0.432	0.418	0.550	0.738	0.425	0.644	0.256	0.219
Q9BZE2	PUS3	0.593	0.597	0.525	0.593	0.595	0.559	0.485	-0.036
P60660	MYL6	-3.377	-3.204	-3.378	-3.358	-3.290	-3.368	0.536	-0.077
O95625	ZBTB11	0.881	0.890	0.847	0.879	0.886	0.863	0.381	-0.023
O75937	DNAJC8	4.556	4.622	4.385	4.466	4.589	4.425	0.093	-0.164
Q9BW62	KATNAL1	-2.254	-2.309	-2.322	-2.272	-2.281	-2.297	0.710	-0.016
A0PJW6	TMEM223	-2.115	-2.281	-2.170	-2.285	-2.198	-2.227	0.801	-0.030
P54886	ALDH18A1	-3.866	-3.860	-4.065	-3.698	-3.863	-3.881	0.937	-0.018
P55010	EIF5	4.832	4.727	4.781	4.663	4.779	4.722	0.543	-0.057
P47895	ALDH1A3	3.414	3.392	3.839	3.756	3.403	3.798	0.051	0.395
P62750	RPL23A	6.497	6.461	6.533	6.479	6.479	6.506	0.504	0.027
Q9Y3A4	RRP7A	-0.525	-0.466	-0.528	-0.444	-0.495	-0.486	0.877	0.009
O75191	XYLB	-2.970	-3.032	-3.071	-2.883	-3.001	-2.977	0.840	0.024
Q9NVV4	MTPAP	-2.945	-2.766	-2.839	-2.781	-2.856	-2.810	0.701	0.046
Q8N5N7	MRPL50	-1.774	-1.550	-1.814	-1.461	-1.662	-1.638	0.919	0.025
Q9P0S2	COX16	-1.243	-1.224	-1.352	-1.303	-1.234	-1.328	0.128	-0.094
Q8IVM0	CCDC50	-2.116	-2.182	-2.143	-2.221	-2.149	-2.182	0.592	-0.032
O75197	LRP5	-5.505	-5.589	-5.494	-5.851	-5.547	-5.673	0.608	-0.125
P27144	AK4	4.431	4.155	4.069	3.783	4.293	3.926	0.206	-0.367
Q9UMS4	PRPF19	5.111	5.129	5.138	5.099	5.120	5.118	0.945	-0.002
Q9H9Y4	GPN2	-1.291	-1.295	-1.461	-1.197	-1.293	-1.329	0.831	-0.036
P13051	UNG	-6.751	-6.876	-6.913	-7.630	-6.813	-7.271	0.418	-0.458
P42575	CASP2	-1.266	-1.222	-1.620	-1.530	-1.244	-1.575	0.048	-0.331
P46781	RPS9	6.659	6.576	6.500	6.459	6.617	6.479	0.141	-0.138
Q9P0U1	TOMM7	3.479	3.451	3.479	3.432	3.465	3.455	0.758	-0.010
P32455	GBP1	0.059	-0.215	0.492	0.059	-0.078	0.275	0.322	0.353
O15118	NPC1	1.162	1.111	1.360	1.276	1.137	1.318	0.087	0.181
P16989	YBX3	-1.685	-1.566	-1.601	-1.487	-1.625	-1.544	0.427	0.082
Q96FF9	CDCA5	-0.412	-0.353	-0.375	-0.410	-0.382	-0.393	0.799	-0.010

P31944	CASP14	-2.218	-2.076	-2.329	-2.079	-2.147	-2.204	0.740	-0.057
Q9H6W3	RIOX1	-0.792	-0.884	-0.832	-0.912	-0.838	-0.872	0.635	-0.034
Q9HAU8	RNPEPL1	-3.052	-3.141	-3.199	-3.125	-3.097	-3.162	0.379	-0.065
Q8WXI4	ACOT11	-4.927	-4.662	-5.087	-4.818	-4.794	-4.952	0.491	-0.158
O43823	AKAP8	2.697	2.674	2.710	2.706	2.686	2.708	0.298	0.022
Q96HJ9	FMC1	-2.807	-2.982	-2.742	-2.862	-2.895	-2.802	0.485	0.093
Q659A1	ICE2	-0.197	-0.144	-0.246	-0.171	-0.171	-0.208	0.509	-0.038
Q9NWX6	THG1L	0.543	0.703	0.639	0.617	0.623	0.628	0.961	0.005
Q92546	RGP1	-3.883	-3.900	-3.909	-3.945	-3.891	-3.927	0.262	-0.036
Q96EL3	MRPL53	1.497	1.445	1.673	1.480	1.471	1.576	0.464	0.106
Q8IWZ8	SUGP1	1.346	1.326	1.255	1.223	1.336	1.239	0.049	-0.097
P47985	UQCRFS1	-3.083	-2.985	-2.816	-2.768	-3.034	-2.792	0.082	0.242
Q8IXI1	RHOT2	1.149	1.206	1.226	1.223	1.178	1.224	0.349	0.047
Q92871	PMM1	0.890	0.807	0.864	0.776	0.848	0.820	0.687	-0.028
Q8N9Q2	SREK1IP1	-5.168	-5.305	-5.347	-5.289	-5.236	-5.318	0.430	-0.081
O95602	POLR1A	3.578	3.563	3.559	3.546	3.571	3.553	0.207	-0.018
Q10713	PMPCA	3.517	3.525	3.453	3.416	3.521	3.434	0.121	-0.086
Q16706	MAN2A1	1.543	1.539	1.697	1.715	1.541	1.706	0.030	0.165
Q9P0J7	KCMF1	-0.229	-0.360	-0.204	-0.285	-0.295	-0.245	0.592	0.050
P55268	LAMB2	-1.860	-1.807	-1.924	-1.764	-1.834	-1.844	0.918	-0.010
Q99638	RAD9A	-1.245	-1.305	-1.391	-1.353	-1.275	-1.372	0.136	-0.097
Q9UIW2	PLXNA1	0.336	0.306	0.331	0.349	0.321	0.340	0.409	0.019
Q92990	GLMN	1.179	1.124	1.116	1.105	1.152	1.111	0.366	-0.041
P33897	ABCD1	-0.269	-0.352	-0.157	-0.238	-0.310	-0.197	0.190	0.113
Q07065	CKAP4	4.599	4.539	4.820	4.774	4.569	4.797	0.030	0.228
Q8IZ69	TRMT2A	-0.436	-0.486	-0.286	-0.414	-0.461	-0.350	0.309	0.111
Q9Y2Z4	YARS2	4.266	4.131	4.221	4.156	4.199	4.189	0.910	-0.010
P28715	ERCC5	-1.024	-1.000	-1.004	-0.919	-1.012	-0.961	0.431	0.051
P19634	SLC9A1	-2.877	-3.015	-2.805	-2.878	-2.946	-2.841	0.345	0.105
Q9UNE7	STUB1	-0.321	-0.324	-0.753	-0.400	-0.323	-0.577	0.387	-0.254
P53634	CTSC	0.885	1.515	1.504	1.537	1.200	1.520	0.495	0.320
Q9H173	SIL1	0.758	0.684	0.831	0.781	0.721	0.806	0.218	0.085
Q99961	SH3GL1	1.332	1.203	1.168	1.123	1.267	1.146	0.289	-0.122
Q07889	SOS1	0.775	0.737	0.706	0.753	0.756	0.729	0.483	-0.026
P42566	EPS15	3.006	2.801	2.795	2.690	2.904	2.742	0.334	-0.161

Q5RKV6	EXOSC6	2.409	2.374	2.386	2.360	2.391	2.373	0.499	-0.018
Q92618	ZNF516	-1.804	-1.642	-1.716	-1.596	-1.723	-1.656	0.580	0.067
P06396	GSN	-2.039	-2.051	-1.973	-1.981	-2.045	-1.977	0.019	0.068
Q9BRT2	UQCC2	0.608	0.632	0.584	0.552	0.620	0.568	0.130	-0.052
Q9Y679	AUP1	-1.412	-1.433	-1.365	-1.324	-1.423	-1.344	0.114	0.078
Q96M96	FGD4	-2.013	-1.769	-1.894	-2.026	-1.891	-1.960	0.680	-0.069
Q9UKU7	ACAD8	-2.318	-2.414	-2.199	-2.294	-2.366	-2.247	0.218	0.119
O15254	ACOX3	-1.752	-1.783	-1.699	-1.538	-1.767	-1.618	0.309	0.149
Q9BQ67	GRWD1	2.769	2.632	2.748	2.614	2.700	2.681	0.860	-0.019
P54922	ADPRH	-1.991	-2.130	-1.888	-1.868	-2.061	-1.878	0.226	0.183
Q9NRD1	FBXO6	0.805	0.742	0.905	0.879	0.773	0.892	0.129	0.119
Q9P2N5	RBM27	3.042	2.969	3.058	3.045	3.005	3.051	0.421	0.046
Q7Z6M1	RABEPK	-0.797	-0.847	-0.864	-0.864	-0.822	-0.864	0.338	-0.043
Q8N9N8	EIF1AD	-0.045	0.155	0.189	0.063	0.055	0.126	0.619	0.071
Q96L58	B3GALT6	-1.381	-1.329	-1.485	-1.348	-1.355	-1.416	0.532	-0.061
Q86V87	FHIP2B	-2.789	-2.663	-2.873	-2.597	-2.726	-2.735	0.962	-0.009
Q9Y4Z0	LSM4	2.373	2.250	2.456	2.409	2.312	2.432	0.274	0.121
Q86V85	GPR180	-1.914	-1.945	-2.060	-2.001	-1.929	-2.031	0.130	-0.102
P49406	MRPL19	3.504	3.430	3.380	3.350	3.467	3.365	0.186	-0.102
Q9BZG8	DPH1	-3.276	-3.187	-3.263	-3.093	-3.231	-3.178	0.650	0.053
Q96BK5	PINX1	0.682	0.548	0.534	0.594	0.615	0.564	0.583	-0.051
Q96AX1	VPS33A	1.125	1.059	1.150	1.168	1.092	1.159	0.275	0.067
Q15464	SHB	-2.735	-2.595	-2.709	-2.584	-2.665	-2.646	0.861	0.019
Q96IU4	ABHD14B	0.056	0.020	-0.089	0.112	0.038	0.011	0.834	-0.027
Q5JTZ9	AARS2	1.971	1.937	1.979	2.011	1.954	1.995	0.221	0.041
Q96BD8	SKA1	-4.026	-4.038	-4.463	-4.132	-4.032	-4.297	0.355	-0.266
Q8NFI3	ENGASE	-1.487	-1.466	-1.619	-1.553	-1.477	-1.586	0.160	-0.110
Q8WVR3	TRAPPC14	-2.587	-2.693	-2.685	-2.695	-2.640	-2.690	0.514	-0.050
Q9NQZ5	STARD7	-0.370	-0.320	-0.257	-0.196	-0.345	-0.227	0.100	0.118
Q8NB46	ANKRD52	-0.392	-0.382	-0.659	-0.234	-0.387	-0.447	0.825	-0.060
Q96DG6	CMBL	3.471	3.306	3.490	3.423	3.388	3.456	0.556	0.068
Q9NUP7	TRMT13	-1.041	-0.920	-1.136	-0.989	-0.980	-1.063	0.481	-0.082
P82673	MRPS35	2.404	2.416	2.369	2.303	2.410	2.336	0.258	-0.074
Q8IV08	PLD3	0.613	0.639	0.761	0.934	0.626	0.848	0.230	0.222
O60216	RAD21	2.228	2.239	2.227	2.187	2.234	2.207	0.397	-0.027

Q9Y676	MRPS18B	1.451	1.493	1.388	1.390	1.472	1.389	0.159	-0.083
P50990	CCT8	4.338	4.223	4.339	4.253	4.280	4.296	0.850	0.016
O75083	WDR1	3.822	3.892	3.894	3.859	3.857	3.876	0.686	0.019
Q9BYN0	SRXN1	-0.283	-0.181	-0.278	-0.031	-0.232	-0.155	0.645	0.077
P83876	TXNL4A	0.541	0.481	0.435	0.317	0.511	0.376	0.219	-0.135
Q9H9H4	VPS37B	1.038	1.125	0.869	0.806	1.082	0.837	0.053	-0.244
Q13315	ATM	2.641	2.583	2.493	2.520	2.612	2.506	0.126	-0.105
Q9NZV1	CRIM1	-1.811	-1.875	-1.922	-1.980	-1.843	-1.951	0.131	-0.108
O14944	EREG	-0.083	-0.198	-0.236	-0.355	-0.141	-0.295	0.203	-0.155
P78316	NOP14	1.606	1.573	1.621	1.702	1.589	1.662	0.296	0.073
Q9NX74	DUS2	1.270	1.196	1.153	1.209	1.233	1.181	0.387	-0.052
P35249	RFC4	-0.163	-0.066	-0.402	-0.400	-0.114	-0.401	0.106	-0.287
Q12972	PPP1R8	-0.764	-0.695	-0.650	-0.738	-0.730	-0.694	0.595	0.036
Q9UBF8	PI4KB	-4.380	-4.539	-4.091	-4.122	-4.460	-4.106	0.130	0.353
O95872	GPANK1	-4.905	-4.973	-5.033	-4.734	-4.939	-4.884	0.776	0.055
O95067	CCNB2	0.423	0.437	0.344	0.376	0.430	0.360	0.099	-0.070
Q6P3S6	FBXO42	-0.769	-0.839	-0.795	-0.743	-0.804	-0.769	0.514	0.035
P51151	RAB9A	-0.529	-0.580	-0.630	-0.445	-0.554	-0.537	0.885	0.017
Q9BRU9	UTP23	0.408	0.365	0.501	0.445	0.386	0.473	0.143	0.087
Q8NCL4	GALNT6	-2.400	-2.322	-2.297	-2.137	-2.361	-2.217	0.291	0.144
Q6YN16	HSDL2	1.594	1.610	1.553	1.638	1.602	1.595	0.899	-0.007
Q6AI08	HEATR6	-0.087	-0.251	-0.112	-0.162	-0.169	-0.137	0.766	0.032
O15347	HMGB3	4.975	4.931	5.002	4.925	4.953	4.964	0.839	0.011
Q9Y546	LRRC42	0.084	0.094	-0.026	0.006	0.089	-0.010	0.080	-0.099
Q9UNH7	SNX6	0.081	-0.038	0.065	0.043	0.021	0.054	0.681	0.033
P53365	ARFIP2	-2.470	-2.497	-2.680	-2.518	-2.484	-2.599	0.386	-0.115
Q9BVM4	GGACT	-2.257	-2.339	-2.256	-2.154	-2.298	-2.205	0.299	0.093
Q9NQ50	MRPL40	1.947	1.845	1.965	1.937	1.896	1.951	0.465	0.055
O75503	CLN5	-3.688	-3.755	-3.852	-3.832	-3.722	-3.842	0.152	-0.120
Q9H061	TMEM126A	0.395	0.345	0.404	0.295	0.370	0.350	0.785	-0.020
Q9BQA1	WDR77	0.439	0.491	0.202	0.285	0.465	0.243	0.063	-0.222
O95825	CRYZL1	1.682	1.585	1.686	1.581	1.633	1.634	0.992	0.001
O60906	SMPD2	-2.407	-2.402	-2.368	-2.313	-2.405	-2.340	0.257	0.064
O14782	KIF3C	1.716	1.473	1.821	1.739	1.594	1.780	0.353	0.186
Q9UQ35	SRRM2	2.756	2.727	2.744	2.712	2.742	2.728	0.586	-0.014

P84085	ARF5	2.363	2.363	2.378	2.372	2.363	2.375	0.144	0.012
Q6P444	MTFR2	-1.825	-1.995	-1.968	-1.995	-1.910	-1.981	0.557	-0.071
Q9H223	EHD4	2.689	2.884	2.517	2.487	2.787	2.502	0.203	-0.285
Q9NTM9	CUTC	0.269	0.379	0.343	0.448	0.324	0.395	0.449	0.071
P17152	TMEM11	1.156	1.140	1.137	1.217	1.148	1.177	0.601	0.029
P05387	RPLP2	5.738	5.511	5.539	5.387	5.625	5.463	0.373	-0.161
Q96EX1	SMIM12	-0.712	-0.608	-0.831	-0.840	-0.660	-0.836	0.181	-0.175
P31323	PRKAR2B	1.832	1.651	1.915	1.946	1.742	1.930	0.278	0.188
Q15776	ZKSCAN8	-2.250	-2.196	-2.303	-2.167	-2.223	-2.235	0.891	-0.012
Q9NZC3	GDE1	-2.625	-2.654	-2.719	-2.570	-2.639	-2.644	0.957	-0.005
P62081	RPS7	5.126	5.058	5.111	5.024	5.092	5.068	0.708	-0.024
Q12874	SF3A3	4.496	4.492	4.558	4.519	4.494	4.538	0.261	0.044
P23193	TCEA1	2.747	2.857	2.972	3.089	2.802	3.030	0.106	0.229
Q92854	SEMA4D	-2.912	-2.823	-2.959	-2.967	-2.867	-2.963	0.274	-0.096
Q01469	FABP5	3.801	3.834	3.863	3.772	3.818	3.818	0.998	0.000
Q9BUL9	RPP25	-0.163	-0.095	-0.080	-0.133	-0.129	-0.107	0.654	0.023
Q96BY9	SARAF	-1.513	-1.489	-1.305	-1.373	-1.501	-1.339	0.103	0.162
Q5JPH6	EARS2	-3.227	-3.367	-3.371	-3.224	-3.297	-3.297	0.996	-0.001
P42166	TMPO	3.539	3.516	3.432	3.426	3.527	3.429	0.063	-0.098
Q7L4I2	RSRC2	-2.356	-2.317	-2.375	-2.219	-2.336	-2.297	0.704	0.039
P49354	FNTA	0.411	0.274	0.183	0.250	0.343	0.217	0.282	-0.126
Q5C9Z4	NOM1	2.180	2.081	2.218	2.189	2.130	2.204	0.362	0.073
O75164	KDM4A	-1.784	-1.786	-1.759	-1.767	-1.785	-1.763	0.118	0.022
P51587	BRCA2	-2.491	-2.560	-2.873	-2.437	-2.525	-2.655	0.658	-0.130
Q9BRX9	WDR83	0.099	0.037	0.001	0.061	0.068	0.031	0.489	-0.037
P10746	UROS	-0.265	-0.325	-0.287	-0.230	-0.295	-0.259	0.470	0.037
Q9BVL4	SELENOO	0.721	0.641	0.703	0.696	0.681	0.699	0.724	0.018
O43242	PSMD3	3.493	3.508	3.505	3.556	3.500	3.531	0.436	0.030
Q9H4I3	TRABD	-1.485	-1.673	-1.453	-1.726	-1.579	-1.590	0.954	-0.011
Q8WU76	SCFD2	-3.334	-3.299	-3.507	-3.258	-3.317	-3.382	0.690	-0.066
P50443	SLC26A2	-3.502	-3.448	-3.768	-3.667	-3.475	-3.718	0.082	-0.243
Q03188	CENPC	0.375	0.351	0.530	0.507	0.363	0.519	0.011	0.156
P15407	FOSL1	0.328	0.161	0.385	0.277	0.244	0.331	0.490	0.087
Q8IWX8	CHERP	4.413	4.313	4.363	4.284	4.363	4.324	0.604	-0.039
Q9NPE2	NGRN	-1.434	-1.428	-1.373	-1.242	-1.431	-1.307	0.311	0.123

P36551	CPOX	2.607	2.539	2.562	2.565	2.573	2.564	0.824	-0.010
Q8NCA5	FAM98A	0.974	0.937	0.859	0.906	0.955	0.882	0.142	-0.073
O15427	SLC16A3	1.719	1.736	1.668	1.592	1.728	1.630	0.222	-0.098
P62263	RPS14	5.279	5.167	5.323	5.123	5.223	5.223	0.999	0.000
Q9NWM8	FKBP14	0.348	0.238	0.152	0.140	0.293	0.146	0.225	-0.147
O95470	SGPL1	3.430	3.515	3.655	3.596	3.473	3.626	0.112	0.153
Q8IZ26	ZNF34	-3.395	-3.260	-3.269	-3.087	-3.328	-3.178	0.328	0.150
O95999	BCL10	1.980	1.937	2.141	2.142	1.959	2.141	0.074	0.183
Q5D862	FLG2	-3.903	-3.853	-4.296	-4.096	-3.878	-4.196	0.176	-0.318
Q9C0C2	TNKS1BP1	2.613	2.589	2.686	2.655	2.601	2.671	0.076	0.070
Q93052	LPP	2.023	2.058	2.120	2.088	2.040	2.104	0.119	0.063
P54253	ATXN1	-2.402	-2.319	-2.415	-2.347	-2.361	-2.381	0.740	-0.021
P22676	CALB2	3.108	3.296	4.455	4.345	3.202	4.400	0.016	1.198
Q969V5	MUL1	-0.372	-0.459	-0.376	-0.365	-0.416	-0.371	0.486	0.045
Q6P4F2	FDX2	-3.285	-3.210	-3.305	-3.492	-3.247	-3.398	0.329	-0.151
Q8N448	LNX2	-2.514	-2.385	-2.553	-2.345	-2.450	-2.449	0.997	0.001
Q99986	VRK1	3.444	3.449	3.170	3.166	3.446	3.168	0.000	-0.278
P47914	RPL29	3.822	3.804	3.789	3.803	3.813	3.796	0.289	-0.017
Q96BP3	PPWD1	1.353	1.298	1.274	1.191	1.325	1.233	0.222	-0.092
P10244	MYBL2	-4.442	-4.161	-4.367	-4.310	-4.302	-4.338	0.837	-0.037
P62380	TBPL1	-0.594	-0.628	-0.667	-0.731	-0.611	-0.699	0.172	-0.088
Q96HV5	TMEM41A	-0.201	-0.211	-0.204	-0.148	-0.206	-0.176	0.470	0.030
Q9UNA4	POLI	-2.847	-2.930	-3.096	-3.008	-2.889	-3.052	0.115	-0.164
Q13443	ADAM9	0.061	0.035	0.046	0.012	0.048	0.029	0.474	-0.019
Q9NUM4	TMEM106B	0.338	0.381	0.516	0.455	0.359	0.486	0.090	0.126
Q7Z7M9	GALNT5	-2.191	-1.900	-2.222	-2.035	-2.046	-2.128	0.688	-0.083
Q96FJ2	DYNLL2	2.621	2.664	2.940	2.802	2.642	2.871	0.161	0.229
Q8N8D1	PDCD7	-0.646	-0.714	-0.703	-0.609	-0.680	-0.656	0.724	0.024
Q6AI39	BICRAL	-0.907	-0.837	-0.939	-0.861	-0.872	-0.900	0.644	-0.028
Q8WUA4	GTF3C2	-0.412	-0.417	-0.415	-0.433	-0.415	-0.424	0.465	-0.010
Q9NPI7	KRCC1	-4.136	-3.943	-4.144	-3.725	-4.039	-3.934	0.709	0.105
Q05209	PTPN12	-0.296	-0.362	-0.380	-0.321	-0.329	-0.350	0.672	-0.022
Q9BX95	SGPP1	0.531	0.481	0.389	0.432	0.506	0.410	0.104	-0.096
P09211	GSTP1	5.627	5.603	5.602	5.541	5.615	5.572	0.371	-0.044
Q16394	EXT1	-2.633	-2.772	-2.674	-2.840	-2.703	-2.757	0.666	-0.054

P52888	THOP1	4.384	4.283	4.273	4.170	4.333	4.222	0.260	-0.112
P40121	CAPG	1.552	1.389	2.009	1.793	1.471	1.901	0.094	0.430
Q96AG4	LRRC59	5.565	5.418	5.563	5.434	5.492	5.498	0.952	0.007
Q96SL8	FIZ1	-4.290	-4.243	-4.408	-4.260	-4.267	-4.334	0.526	-0.067
P52429	DGKE	-0.102	-0.116	0.022	0.024	-0.109	0.023	0.029	0.132
Q9UGY1	NOL12	-1.540	-1.595	-1.648	-1.556	-1.568	-1.602	0.601	-0.034
Q9UMN6	KMT2B	0.796	0.779	0.753	0.834	0.787	0.794	0.901	0.006
O94817	ATG12	-0.417	-0.605	-0.478	-0.507	-0.511	-0.493	0.879	0.018
Q8TCG1	CIP2A	-0.377	-0.542	-0.603	-0.606	-0.460	-0.605	0.330	-0.145
Q99683	MAP3K5	-4.941	-4.670	-4.801	-4.696	-4.805	-4.748	0.750	0.057
Q9H4B6	SAV1	-1.058	-0.977	-1.062	-0.996	-1.017	-1.029	0.848	-0.011
Q8NFZ8	CADM4	-1.662	-1.590	-1.681	-1.627	-1.626	-1.654	0.606	-0.028
Q99496	RNF2	-2.155	-2.176	-1.988	-1.961	-2.166	-1.974	0.009	0.192
Q96HN2	AHCYL2	-1.234	-1.039	-1.458	-1.174	-1.137	-1.316	0.419	-0.179
P56277	CMC4	-0.753	-0.881	-0.805	-0.905	-0.817	-0.855	0.690	-0.038
Q9NQG6	MIEF1	-1.063	-1.147	-0.967	-0.996	-1.105	-0.982	0.177	0.123
O94766	B3GAT3	-3.894	-3.965	-3.968	-3.889	-3.930	-3.928	0.984	0.001
Q9HD34	LYRM4	1.101	1.068	1.047	1.105	1.085	1.076	0.826	-0.009
Q9C0B1	FTO	1.922	1.833	1.773	1.749	1.877	1.761	0.213	-0.116
Q7LGA3	HS2ST1	-1.408	-1.311	-1.543	-1.324	-1.359	-1.434	0.621	-0.074
Q9NVR0	KLHL11	-0.511	-0.545	-0.503	-0.514	-0.528	-0.508	0.447	0.019
Q14728	MFSD10	0.196	0.155	0.362	0.301	0.176	0.332	0.065	0.156
P62826	RAN	6.178	6.121	6.170	6.151	6.150	6.161	0.769	0.011
P60002	ELOF1	-2.167	-1.707	-1.865	-1.690	-1.937	-1.777	0.614	0.160
Q99952	PTPN18	-3.974	-4.033	-4.059	-4.005	-4.003	-4.032	0.546	-0.029
Q92828	CORO2A	0.072	0.149	0.273	0.282	0.110	0.277	0.139	0.167
Q07617	SPAG1	-0.272	-0.183	-0.051	-0.124	-0.228	-0.087	0.139	0.140
O75420	GIGYF1	0.883	0.771	0.863	0.835	0.827	0.849	0.757	0.023
P49247	RPIA	1.406	1.384	1.424	1.444	1.395	1.434	0.123	0.039
Q96KQ4	PPP1R13B	0.303	0.332	0.127	0.137	0.317	0.132	0.028	-0.185
Q5JUR7	TEX30	-1.121	-1.178	-1.106	-1.206	-1.149	-1.156	0.920	-0.007
O15050	TRANK1	-3.355	-3.354	-3.371	-3.285	-3.355	-3.328	0.650	0.026
O60927	PPP1R11	-1.356	-1.224	-1.274	-1.373	-1.290	-1.324	0.724	-0.034
P21589	NT5E	-1.813	-1.952	-1.767	-1.888	-1.883	-1.827	0.608	0.056
Q5VVJ2	MYSM1	-1.948	-2.104	-2.083	-2.178	-2.026	-2.131	0.390	-0.105

Q9UJJ9	GNPTG	-1.761	-1.771	-1.452	-1.576	-1.766	-1.514	0.152	0.252
Q9UBB5	MBD2	-2.234	-1.910	-2.200	-2.237	-2.072	-2.219	0.531	-0.147
Q9NQS3	NECTIN3	-4.789	-4.827	-4.750	-4.413	-4.808	-4.582	0.405	0.227
P68402	PAFAH1B2	-2.039	-1.833	-2.185	-1.790	-1.936	-1.988	0.844	-0.052
Q8TDP1	RNASEH2C	0.189	0.179	0.005	0.104	0.184	0.055	0.228	-0.129
P16083	NQO2	1.653	1.517	1.859	1.824	1.585	1.841	0.146	0.256
Q8WW35	DYNLT2B	-3.062	-3.107	-3.155	-3.063	-3.084	-3.109	0.692	-0.025
Q6P1N9	TATDN1	-1.372	-1.496	-1.503	-1.561	-1.434	-1.532	0.336	-0.098
Q9UHY7	ENOPH1	1.959	2.033	1.957	2.004	1.996	1.980	0.759	-0.016
Q9UIJ7	AK3	-1.475	-1.409	-1.190	-1.195	-1.442	-1.193	0.082	0.250
Q9UM82	SPATA2	-1.945	-1.877	-2.003	-1.942	-1.911	-1.972	0.310	-0.062
Q14696	MESD	1.856	2.028	1.825	1.784	1.942	1.804	0.345	-0.137
Q8TBA6	GOLGA5	-2.547	-2.600	-2.624	-2.673	-2.573	-2.649	0.171	-0.075
O43657	TSPAN6	-3.739	-2.801	-3.335	-3.304	-3.270	-3.319	0.933	-0.049
Q13185	CBX3	5.905	6.019	5.913	5.939	5.962	5.926	0.644	-0.036
P04844	RPN2	0.179	0.110	0.123	0.138	0.144	0.131	0.762	-0.014
Q13435	SF3B2	4.875	4.856	4.980	4.919	4.866	4.949	0.198	0.083
Q96CF2	CHMP4C	-1.387	-1.198	-1.285	-1.369	-1.293	-1.327	0.780	-0.035
Q53HL2	CDCA8	0.300	0.380	0.327	0.324	0.340	0.325	0.778	-0.015
Q9UKM7	MAN1B1	1.189	1.266	1.066	1.253	1.227	1.160	0.601	-0.068
O14503	BHLHE40	-0.706	-0.734	-0.707	-0.682	-0.720	-0.695	0.310	0.025
Q96DE5	ANAPC16	-1.241	-1.073	-0.963	-0.952	-1.157	-0.957	0.252	0.200
O15541	RNF113A	0.057	0.064	0.136	0.103	0.061	0.120	0.158	0.059
O95678	KRT75	-5.037	-5.117	-4.956	-4.976	-5.077	-4.966	0.205	0.111
Q9BX68	HINT2	1.761	1.683	1.711	1.606	1.722	1.658	0.439	-0.064
Q8IWJ2	GCC2	1.986	1.934	1.980	2.007	1.960	1.994	0.400	0.033
O60318	MCM3AP	1.716	1.666	1.808	1.773	1.691	1.790	0.098	0.100
Q7Z7L7	ZER1	-2.972	-2.882	-2.917	-2.761	-2.927	-2.839	0.451	0.088
P82933	MRPS9	2.542	2.574	2.528	2.551	2.558	2.539	0.446	-0.019
O14949	UQCRQ	3.782	3.739	3.722	3.701	3.761	3.711	0.223	-0.049
Q9NYY8	FASTKD2	-1.454	-1.440	-1.506	-1.690	-1.447	-1.598	0.346	-0.151
Q53GL7	PARP10	-2.673	-2.640	-2.403	-2.361	-2.656	-2.382	0.011	0.274
P61962	DCAF7	1.397	1.445	1.399	1.550	1.421	1.475	0.604	0.054
Q9H2C0	GAN	0.535	0.535	0.654	0.638	0.535	0.646	0.045	0.111
P54727	RAD23B	3.012	3.086	3.060	3.094	3.049	3.077	0.592	0.028

Q9UFC0	LRWD1	2.267	2.247	2.100	2.185	2.257	2.143	0.212	-0.114
Q8NBL1	POGLUT1	-0.436	-0.676	-0.568	-0.554	-0.556	-0.561	0.975	-0.005
O95573	ACSL3	4.081	4.220	4.185	4.187	4.150	4.186	0.697	0.036
P08133	ANXA6	1.652	1.602	1.456	1.401	1.627	1.428	0.034	-0.199
Q9UQN3	CHMP2B	0.188	0.128	0.357	0.229	0.158	0.293	0.245	0.135
O00629	KPNA4	1.828	1.725	1.675	1.641	1.776	1.658	0.237	-0.118
Q03001	DST	0.298	0.270	0.132	0.148	0.284	0.140	0.026	-0.144
Q9UI14	RABAC1	0.138	0.146	0.248	0.269	0.142	0.259	0.034	0.117
P98196	ATP11A	-0.041	-0.113	-0.004	-0.056	-0.077	-0.030	0.411	0.047
Q32NC0	C18orf21	-2.145	-2.169	-2.138	-2.183	-2.157	-2.161	0.901	-0.004
P00846	MT	2.915	2.965	2.844	2.889	2.940	2.866	0.162	-0.074
Q8IWB1	ITPRIP	0.557	0.606	0.682	0.725	0.581	0.704	0.065	0.123
P25789	PSMA4	3.068	2.964	2.901	2.891	3.016	2.896	0.258	-0.121
Q9H6S0	YTHDC2	2.366	2.302	2.280	2.290	2.334	2.285	0.366	-0.049
O14802	POLR3A	1.842	1.831	1.823	1.934	1.837	1.878	0.588	0.042
P54802	NAGLU	1.106	1.073	1.214	1.203	1.089	1.208	0.063	0.119
P40763	STAT3	-3.880	-3.599	-3.758	-3.568	-3.740	-3.663	0.700	0.077
Q96DX7	TRIM44	-2.142	-2.082	-1.954	-1.936	-2.112	-1.945	0.092	0.167
Q6ZS30	NBEAL1	-2.125	-2.246	-2.033	-2.214	-2.186	-2.124	0.635	0.062
Q9NP61	ARFGAP3	-1.038	-1.136	-0.914	-0.911	-1.087	-0.912	0.175	0.174
O00299	CLIC1	6.087	6.065	6.341	6.243	6.076	6.292	0.128	0.216
Q9UPN7	PPP6R1	0.983	1.038	1.026	1.042	1.011	1.034	0.557	0.023
P55212	CASP6	-0.991	-0.821	-0.961	-0.975	-0.906	-0.968	0.598	-0.062
Q9BUR4	WRAP53	-0.333	-0.276	-0.389	-0.337	-0.304	-0.363	0.267	-0.059
Q14146	URB2	2.522	2.507	2.524	2.547	2.515	2.535	0.296	0.021
P60763	RAC3	-3.929	-3.720	-3.757	-3.675	-3.825	-3.716	0.479	0.109
Q96BM9	ARL8A	1.773	1.734	2.030	2.036	1.754	2.033	0.040	0.280
Q9H9B1	EHMT1	-2.019	-2.003	-2.149	-2.070	-2.011	-2.109	0.231	-0.098
Q14677	CLINT1	-2.287	-2.349	-2.291	-2.409	-2.318	-2.350	0.691	-0.032
Q8N8R5	C2orf69	-0.212	-0.290	-0.331	-0.334	-0.251	-0.332	0.285	-0.081
O15240	VGF	-2.196	-2.115	-2.266	-2.177	-2.155	-2.221	0.387	-0.066
O14684	PTGES	-1.491	-1.469	-0.808	-1.022	-1.480	-0.915	0.116	0.565
Q96Q42	ALS2	1.726	1.623	1.642	1.623	1.675	1.633	0.563	-0.042
Q9UBI6	GNG12	0.369	0.233	0.322	0.228	0.301	0.275	0.785	-0.026
Q9NQ89	C12orf4	-0.799	-0.825	-0.896	-0.803	-0.812	-0.849	0.564	-0.037

O95757	HSPA4L	3.754	3.694	3.775	3.729	3.724	3.752	0.539	0.028
Q8WXF1	PSPC1	1.451	1.275	1.380	1.244	1.363	1.312	0.692	-0.051
P55786	NPEPPS	2.132	2.088	2.113	2.138	2.110	2.125	0.617	0.015
Q7L775	EPM2AIP1	0.054	0.251	-0.029	0.283	0.152	0.127	0.904	-0.026
P10644	PRKAR1A	0.900	0.879	0.734	0.804	0.889	0.769	0.156	-0.120
Q86UA1	PRPF39	0.839	0.707	0.754	0.696	0.773	0.725	0.599	-0.048
Q8WVT3	TRAPPC12	0.610	0.538	0.522	0.484	0.574	0.503	0.262	-0.071
Q7RTV5	PRXL2C	-3.732	-3.522	-3.490	-3.609	-3.627	-3.549	0.601	0.078
Q9BVI0	PHF20	-1.841	-1.924	-1.783	-1.796	-1.882	-1.789	0.260	0.093
Q8TE82	SH3TC1	-3.226	-2.897	-3.297	-3.009	-3.061	-3.153	0.717	-0.091
P62899	RPL31	-1.636	-1.244	-1.434	-1.271	-1.440	-1.353	0.737	0.088
Q15370	ELOB	-2.044	-1.797	-1.811	-2.161	-1.920	-1.986	0.791	-0.066
Q3MIR4	TMEM30B	-5.706	-4.805	-5.566	-4.782	-5.255	-5.174	0.904	0.081
P07099	EPHX1	3.817	3.791	3.754	3.771	3.804	3.763	0.139	-0.041
P05141	SLC25A5	6.080	6.010	5.987	5.989	6.045	5.988	0.351	-0.057
Q7L9B9	EEPD1	-0.065	-0.249	-0.357	-0.297	-0.157	-0.327	0.297	-0.170
Q9H2K8	TAOK3	-0.432	-0.452	-0.342	-0.401	-0.442	-0.371	0.225	0.071
P20248	CCNA2	-0.399	-0.265	-0.388	-0.270	-0.332	-0.329	0.977	0.003
Q96DX4	RSPRY1	-0.703	-0.743	-0.876	-0.682	-0.723	-0.779	0.666	-0.056
E7ERA6	RNF223	-5.215	-5.006	-5.100	-5.080	-5.110	-5.090	0.877	0.020
Q92585	MAML1	-3.055	-3.073	-3.170	-3.068	-3.064	-3.119	0.477	-0.055
P53621	COPA	-1.936	-1.995	-2.125	-1.677	-1.966	-1.901	0.821	0.065
Q01130	SRSF2	-0.115	-0.180	-0.099	-0.126	-0.147	-0.112	0.464	0.035
O43293	DAPK3	-1.709	-1.694	-1.756	-1.719	-1.701	-1.737	0.274	-0.036
Q9P2D1	CHD7	-0.063	-0.064	-0.502	-0.379	-0.064	-0.441	0.104	-0.377
P19338	NCL	8.610	8.537	8.654	8.602	8.574	8.628	0.359	0.055
Q9NRX5	SERINC1	0.826	0.721	0.982	0.949	0.774	0.966	0.144	0.192
Q6P4I2	WDR73	-1.017	-0.823	-1.000	-0.933	-0.920	-0.966	0.718	-0.046
Q96R06	SPAG5	0.777	0.802	0.628	0.657	0.789	0.643	0.018	-0.147
Q9BV40	VAMP8	-0.869	-0.762	-0.849	-0.968	-0.816	-0.908	0.367	-0.093
O75110	ATP9A	-1.889	-1.906	-1.947	-1.850	-1.898	-1.899	0.989	-0.001
P28749	RBL1	-3.112	-3.111	-3.194	-3.143	-3.112	-3.168	0.267	-0.057
O15211	RGL2	-3.320	-3.332	-3.135	-3.163	-3.326	-3.149	0.028	0.177
Q9NP77	SSU72	-0.273	-0.275	-0.468	-0.287	-0.274	-0.378	0.458	-0.104
O95167	NDUFA3	1.739	1.784	1.801	1.963	1.762	1.882	0.366	0.120

P04080	CSTB	4.099	4.187	4.299	4.251	4.143	4.275	0.154	0.132
P36871	PGM1	-0.115	-0.207	0.115	-0.090	-0.161	0.013	0.311	0.174
Q9Y2I7	PIKFYVE	0.302	0.237	0.293	0.391	0.270	0.342	0.360	0.072
O14880	MGST3	0.158	0.127	0.150	0.249	0.143	0.200	0.444	0.057
P09669	COX6C	3.756	3.807	3.667	3.671	3.782	3.669	0.139	-0.113
Q9NVH0	EXD2	-2.452	-2.456	-2.592	-2.279	-2.454	-2.435	0.924	0.019
Q9BRS8	LARP6	-0.534	-0.589	-0.611	-0.618	-0.561	-0.614	0.299	-0.053
Q8WV92	MITD1	0.279	0.081	0.132	0.098	0.180	0.115	0.629	-0.065
Q10471	GALNT2	1.653	1.708	1.563	1.611	1.681	1.587	0.127	-0.093
Q8IV32	CCDC71	-2.097	-2.164	-2.133	-2.118	-2.131	-2.126	0.903	0.005
P51692	STAT5B	-0.606	-0.643	-0.676	-0.627	-0.624	-0.651	0.481	-0.027
Q53FZ2	ACSM3	-4.105	-4.036	-3.973	-3.965	-4.071	-3.969	0.204	0.101
P56181	NDUFV3	2.366	2.295	2.336	2.336	2.330	2.336	0.908	0.005
P63172	DYNLT1	0.779	0.662	0.881	0.805	0.720	0.843	0.241	0.123
Q9BXY0	MAK16	2.640	2.522	2.651	2.590	2.581	2.621	0.629	0.040
Q70E73	RAPH1	0.222	0.259	0.287	0.340	0.241	0.314	0.170	0.073
Q6PL24	TMED8	0.153	0.277	0.130	0.317	0.215	0.224	0.946	0.009
P22415	USF1	-2.948	-3.114	-3.048	-3.034	-3.031	-3.041	0.924	-0.010
P15260	IFNGR1	-1.975	-1.712	-1.912	-1.581	-1.843	-1.746	0.692	0.097
O95365	ZBTB7A	-0.326	-0.322	-0.352	-0.290	-0.324	-0.321	0.937	0.003
Q9UNW9	NOVA2	0.257	0.209	0.247	0.176	0.233	0.211	0.669	-0.022
Q9UBW8	COPS7A	1.357	1.322	1.258	1.260	1.339	1.259	0.134	-0.080
Q9UNX4	WDR3	3.935	3.849	3.935	3.924	3.892	3.930	0.538	0.038
O15143	ARPC1B	4.543	4.474	4.565	4.512	4.508	4.538	0.569	0.030
Q7Z739	YTHDF3	1.288	1.379	1.309	1.346	1.334	1.328	0.922	-0.006
Q27J81	INF2	-3.532	-3.144	-3.474	-3.479	-3.338	-3.476	0.605	-0.138
Q14807	KIF22	-2.746	-2.388	-2.755	-2.478	-2.567	-2.617	0.848	-0.050
Q9NUP1	BLOC1S4	-1.794	-1.742	-1.797	-1.739	-1.768	-1.768	0.997	0.000
Q9BY11	PACSIN1	-0.471	-0.640	-0.589	-0.644	-0.555	-0.617	0.597	-0.061
Q13177	PAK2	5.403	5.355	5.248	5.284	5.379	5.266	0.073	-0.113
Q9BWN1	PRR14	-1.798	-1.866	-1.723	-1.786	-1.832	-1.755	0.239	0.077
Q8TAP8	PPP1R35	-0.891	-0.949	-0.849	-0.860	-0.920	-0.854	0.254	0.066
O15047	SETD1A	1.101	1.062	1.162	1.142	1.081	1.152	0.122	0.071
Q8N6I1	EID2	-2.553	-2.529	-2.692	-2.436	-2.541	-2.564	0.887	-0.023
Q96I59	NARS2	1.530	1.394	1.783	1.704	1.462	1.743	0.094	0.281

Q96BQ5	CCDC127		-0.106	-0.161	-0.045	-0.093	-0.133	-0.069	0.220	0.064
Q03111	MLLT1		0.011	0.149	0.138	0.124	0.080	0.131	0.593	0.051
Q08357	SLC20A2		-0.455	-0.175	-0.314	-0.261	-0.315	-0.288	0.877	0.028
P35869	AHR		-1.254	-1.118	-0.925	-1.141	-1.186	-1.033	0.373	0.153
Q9Y2F5	ICE1		-0.848	-0.835	-0.924	-0.687	-0.841	-0.805	0.812	0.036
Q96JB6	LOXL4		-4.197	-3.977	-4.245	-4.006	-4.087	-4.126	0.834	-0.039
Q86YT6	MIB1		0.128	0.114	0.173	0.254	0.121	0.214	0.255	0.092
Q15155	NOMO1		-2.729	-2.947	-2.961	-2.913	-2.838	-2.937	0.527	-0.099
O75400	PRPF40A		-3.279	-3.340	-3.476	-3.150	-3.310	-3.313	0.986	-0.004
Q4G176	ACSF3		2.941	2.859	3.067	3.072	2.900	3.070	0.151	0.169
Q99707	MTR		-1.551	-1.386	-1.593	-1.364	-1.469	-1.478	0.952	-0.010
P00813	ADA		2.124	2.040	2.111	2.139	2.082	2.125	0.485	0.043
Q9P2B7	CFAP97		-2.853	-2.829	-2.756	-2.642	-2.841	-2.699	0.232	0.142
P50135	HNMT		-2.162	-2.112	-1.752	-1.790	-2.137	-1.771	0.010	0.366
Q9ULC3	RAB23		1.683	1.645	1.550	1.577	1.664	1.563	0.060	-0.100
Q9BRS2	RIOK1		1.848	1.845	1.695	1.774	1.847	1.735	0.215	-0.112
P09488	GSTM1		1.879	1.807	1.828	1.860	1.843	1.844	0.971	0.002
P35226	BMI1		-3.162	-2.943	-2.861	-2.892	-3.052	-2.877	0.351	0.176
P42766	RPL35		5.060	5.044	5.066	5.061	5.052	5.063	0.371	0.011
Q9H4B7	TUBB1		-1.061	-1.229	-1.439	-1.226	-1.145	-1.332	0.307	-0.188
Q9H0Q0	CYRIA		-2.753	-2.770	-2.884	-2.705	-2.761	-2.794	0.775	-0.033
Q6ZSR9		0	0.512	0.488	0.424	0.536	0.500	0.480	0.784	-0.020
Q99653	CHP1		2.990	2.914	2.914	2.881	2.952	2.897	0.368	-0.055
Q96NC0	ZMAT2		2.395	2.190	2.358	2.226	2.292	2.292	0.998	0.000
O43678	NDUFA2		-0.997	-1.056	-0.941	-0.891	-1.027	-0.916	0.105	0.111
Q96GW9	MARS2		0.984	0.980	0.973	1.049	0.982	1.011	0.589	0.029
Q2M389	WASHC4		1.235	1.137	1.092	1.072	1.186	1.082	0.271	-0.104
Q9H8E8	KAT14		-2.159	-2.226	-2.179	-2.107	-2.193	-2.143	0.416	0.050
P0C7T5	ATXN1L		-0.782	-0.885	-0.833	-0.823	-0.834	-0.828	0.934	0.005
Q9GZU8	PSME3IP1		2.274	2.278	2.079	2.085	2.276	2.082	0.001	-0.193
Q96C23	GALM		-0.952	-0.633	-0.821	-0.791	-0.792	-0.806	0.947	-0.013
Q4G0A6	MINDY4		-5.231	-5.341	-5.274	-5.560	-5.286	-5.417	0.522	-0.131
Q96RY7	IFT140		-1.666	-1.688	-1.574	-1.520	-1.677	-1.547	0.094	0.130
P07902	GALT		-1.137	-1.199	-1.092	-0.951	-1.168	-1.021	0.251	0.146
Q8TD19	NEK9		3.731	3.698	3.439	3.415	3.714	3.427	0.007	-0.287

O00170	AIP	3.000	3.048	2.854	2.818	3.024	2.836	0.028	-0.188
Q99439	CNN2	0.981	0.919	0.950	0.841	0.950	0.895	0.497	-0.054
O75131	CPNE3	4.128	4.133	4.138	4.066	4.130	4.102	0.577	-0.028
Q9Y2V7	COG6	-3.507	-3.090	-3.493	-2.940	-3.299	-3.217	0.837	0.082
Q99547	MPHOSPH6	0.735	0.676	0.798	0.703	0.705	0.751	0.515	0.045
O43909	EXTL3	-4.981	-5.025	-5.101	-4.967	-5.003	-5.034	0.729	-0.031
O43296	ZNF264	-2.378	-2.539	-2.417	-2.394	-2.458	-2.406	0.630	0.053
Q9H668	STN1	-2.778	-2.739	-2.770	-2.785	-2.758	-2.778	0.497	-0.019
P56199	ITGA1	-3.210	-3.088	-3.227	-3.041	-3.149	-3.134	0.908	0.015
Q9UI10	EIF2B4	-5.691	-5.593	-5.725	-5.594	-5.642	-5.660	0.849	-0.018
Q8IVY1	C1orf210	-7.208	-7.247	-7.412	-7.092	-7.227	-7.252	0.904	-0.024
P26038	MSN	6.256	6.155	6.071	6.073	6.205	6.072	0.231	-0.133
Q8N201	INTS1	2.431	2.403	2.427	2.441	2.417	2.434	0.420	0.017
Q96GY3	LIN37	-0.936	-0.947	-0.945	-0.935	-0.941	-0.940	0.883	0.001
Q96CX2	KCTD12	-4.910	-4.678	-5.044	-4.867	-4.794	-4.955	0.390	-0.161
Q5SWA1	PPP1R15B	-3.853	-3.766	-3.938	-3.697	-3.809	-3.817	0.959	-0.008
Q8TB52	FBXO30	0.569	0.526	0.535	0.593	0.547	0.564	0.701	0.016
P62191	PSMC1	2.163	2.086	2.041	2.028	2.125	2.035	0.251	-0.090
Q9H4I2	ZHX3	-2.845	-2.999	-2.996	-3.058	-2.922	-3.027	0.385	-0.105
Q9NRP4	SDHAF3	-0.072	-0.113	-0.349	-0.307	-0.093	-0.328	0.015	-0.235
Q96IR7	HPDL	0.524	0.993	0.709	0.802	0.758	0.756	0.993	-0.003
O95707	POP4	0.110	0.158	0.148	0.138	0.134	0.143	0.769	0.009
O95402	MED26	-2.953	-3.031	-2.941	-3.040	-2.992	-2.991	0.983	0.002
Q3KRA6	C2orf76	-2.045	-1.933	-2.210	-1.982	-1.989	-2.096	0.514	-0.107
Q4KMQ1	TPRN	-2.161	-2.098	-2.380	-2.372	-2.129	-2.376	0.077	-0.247
B4DX44	ZNF736	-3.815	-4.056	-3.862	-3.776	-3.935	-3.819	0.506	0.116
Q9HC07	TMEM165	-3.100	-3.019	-2.968	-2.873	-3.059	-2.921	0.160	0.139
P21397	MAOA	-3.434	-3.715	-3.413	-3.512	-3.574	-3.463	0.570	0.111
Q9NUE0	ZDHHC18	-4.881	-4.870	-4.885	-4.931	-4.876	-4.908	0.387	-0.032
O75643	SNRNP200	5.532	5.494	5.470	5.447	5.513	5.458	0.159	-0.054
Q9NS91	RAD18	-0.847	-0.715	-0.874	-0.802	-0.781	-0.838	0.546	-0.057
P56537	EIF6	0.373	0.351	0.376	0.390	0.362	0.383	0.264	0.021
P49773	HINT1	4.003	3.974	3.904	3.953	3.989	3.928	0.193	-0.060
Q8IUW5	RELL1	-1.834	-1.848	-1.951	-1.826	-1.841	-1.889	0.586	-0.047
Q6IEG0	SNRNP48	-3.178	-3.181	-3.136	-3.143	-3.179	-3.140	0.040	0.040

Q8WUF5	PPP1R13L	0.801	1.124	0.935	0.899	0.962	0.917	0.826	-0.045
Q8N9M5	TMEM102	-3.118	-3.080	-3.140	-3.158	-3.099	-3.149	0.194	-0.050
Q9NRM2	ZNF277	-2.327	-2.295	-2.107	-2.125	-2.311	-2.116	0.018	0.195
P62136	PPP1CA	-1.472	-1.421	-1.452	-1.531	-1.446	-1.491	0.454	-0.045
Q13569	TDG	-1.712	-1.776	-1.680	-1.637	-1.744	-1.658	0.176	0.085
P41214	EIF2D	-2.009	-2.077	-2.066	-2.097	-2.043	-2.081	0.447	-0.038
O14737	PDCD5	3.601	3.555	3.444	3.519	3.578	3.481	0.185	-0.097
Q15847	ADIRF	3.041	2.905	2.131	2.206	2.973	2.168	0.020	-0.805
P46926	GNPDA1	0.057	-0.049	-0.159	-0.213	0.004	-0.186	0.125	-0.190
Q04837	SSBP1	3.751	3.647	3.777	3.755	3.699	3.766	0.411	0.068
O75190	DNAJB6	-1.976	-2.081	-1.692	-1.631	-2.029	-1.662	0.043	0.367
O00244	ATOX1	2.219	2.444	2.231	2.383	2.332	2.307	0.873	-0.025
Q9H993	ARMT1	3.133	3.024	3.040	3.014	3.078	3.027	0.514	-0.052
Q86V15	CASZ1	-1.455	-1.306	-1.395	-1.316	-1.380	-1.355	0.804	0.025
Q9Y3A6	TMED5	0.268	0.213	0.219	0.291	0.240	0.255	0.786	0.014
O60341	KDM1A	-2.704	-2.421	-2.649	-2.387	-2.562	-2.518	0.840	0.044
Q5TZA2	CROCC	-1.175	-1.230	-1.341	-1.307	-1.203	-1.324	0.084	-0.121
P36955	SERPINF1	-0.058	-0.116	-0.080	-0.109	-0.087	-0.095	0.840	-0.008
Q7Z3E5	ARMC9	-5.272	-5.403	-5.441	-5.457	-5.338	-5.449	0.336	-0.111
O00287	RFXAP	-4.083	-3.992	-4.190	-4.028	-4.038	-4.109	0.539	-0.072
Q99758	ABCA3	-3.029	-3.094	-3.123	-3.015	-3.061	-3.069	0.918	-0.008
P67936	TPM4	1.414	1.253	1.548	1.487	1.333	1.518	0.233	0.184
Q9HC36	MRM3	1.383	1.340	1.461	1.461	1.362	1.461	0.135	0.099
Q13427	PPIG	2.197	2.115	2.318	2.227	2.156	2.273	0.201	0.116
Q9Y314	NOSIP	1.724	1.708	1.522	1.512	1.716	1.517	0.005	-0.199
P35998	PSMC2	4.801	4.785	4.704	4.701	4.793	4.703	0.050	-0.091
P55198	MLLT6	-2.296	-2.325	-2.141	-2.209	-2.310	-2.175	0.119	0.135
P82909	MRPS36	0.870	0.774	0.938	0.917	0.822	0.927	0.259	0.105
O43633	CHMP2A	0.957	0.900	1.060	1.019	0.929	1.040	0.098	0.111
Q9UHL4	DPP7	-0.226	-0.191	-0.033	-0.014	-0.208	-0.024	0.025	0.184
Q96N03	VSTM2L	-3.009	-3.018	-3.207	-3.051	-3.013	-3.129	0.377	-0.115
A6NFI3	ZNF316	-0.629	-0.608	-0.572	-0.527	-0.619	-0.549	0.157	0.069
O95239	KIF4A	-0.537	-0.496	-0.844	-0.796	-0.517	-0.820	0.011	-0.303
P08238	HSP90AB1	6.447	6.359	6.120	6.400	6.403	6.260	0.486	-0.143
Q8N5L8	RPP25L	0.267	0.213	0.414	0.343	0.240	0.378	0.099	0.138

Q96I23	PYURF	0.140	-0.074	0.184	0.227	0.033	0.205	0.346	0.172
Q8TC26	TMEM163	-4.182	-4.226	-3.994	-4.155	-4.204	-4.075	0.342	0.129
O15235	MRPS12	0.603	0.522	0.717	0.571	0.563	0.644	0.458	0.081
P63167	DYNLL1	2.999	2.936	2.964	2.900	2.967	2.932	0.510	-0.035
Q9UKD2	MRTO4	2.708	2.727	2.808	2.746	2.717	2.777	0.282	0.060
O75569	PRKRA	-2.130	-1.966	-1.918	-1.934	-2.048	-1.926	0.375	0.122
Q14151	SAFB2	2.546	2.459	2.560	2.521	2.503	2.541	0.541	0.038
Q7L5N1	COPS6	3.865	3.865	3.694	3.766	3.865	3.730	0.165	-0.135
Q8IZV5	RDH10	-1.166	-1.014	-1.099	-0.898	-1.090	-0.998	0.547	0.092
P62993	GRB2	2.055	1.995	2.272	2.201	2.025	2.237	0.047	0.212
Q13459	MYO9B	-2.455	-2.484	-2.613	-2.596	-2.470	-2.604	0.026	-0.135
Q66K89	E4F1	-2.831	-2.942	-2.942	-2.913	-2.887	-2.927	0.596	-0.041
Q9NPF4	OSGEP	0.142	0.243	0.220	0.142	0.192	0.181	0.878	-0.011
Q96C24	SYTL4	-3.145	-2.939	-2.936	-2.822	-3.042	-2.879	0.331	0.163
Q9BY50	SEC11C	-1.941	-2.098	-1.807	-2.039	-2.020	-1.923	0.569	0.097
P24385	CCND1	-1.823	-1.749	-1.776	-1.827	-1.786	-1.801	0.761	-0.016
Q8TCJ2	STT3B	3.153	3.082	3.043	3.030	3.118	3.037	0.257	-0.081
Q8WVM0	TFB1M	0.924	0.851	0.835	0.820	0.887	0.828	0.340	-0.060
Q9BW60	ELOVL1	-2.152	-2.354	-2.148	-2.371	-2.253	-2.259	0.969	-0.007
P54709	ATP1B3	4.354	4.387	4.071	4.122	4.370	4.096	0.020	-0.274
Q01664	TFAP4	-0.187	-0.230	-0.215	-0.158	-0.209	-0.187	0.609	0.022
Q9HAV7	GRPEL1	3.531	3.531	3.553	3.525	3.531	3.539	0.679	0.008
P11047	LAMC1	1.749	1.734	1.865	1.812	1.742	1.838	0.151	0.097
Q9P2B4	CTTNBP2NL	0.395	0.453	0.365	0.416	0.424	0.390	0.481	-0.033
Q13573	SNW1	3.951	3.852	3.917	3.845	3.902	3.881	0.771	-0.021
O60437	PPL	2.657	3.011	2.920	2.771	2.834	2.845	0.960	0.012
Q6UXV4	APOOL	0.186	0.344	0.441	0.636	0.265	0.538	0.166	0.274
Q8ND82	ZNF280C	2.593	2.554	2.576	2.586	2.573	2.581	0.752	0.008
P25685	DNAJB1	2.641	2.555	2.660	2.617	2.598	2.638	0.516	0.041
Q6RFH5	WDR74	-1.044	-0.797	-0.718	-0.938	-0.920	-0.828	0.633	0.092
Q15437	SEC23B	2.200	2.195	2.246	2.237	2.198	2.242	0.027	0.044
Q13884	SNTB1	-1.751	-1.473	-1.942	-1.689	-1.612	-1.815	0.392	-0.204
P63098	PPP3R1	-1.679	-1.728	-1.741	-1.730	-1.704	-1.735	0.406	-0.032
Q15006	EMC2	0.707	0.638	0.641	0.689	0.672	0.665	0.875	-0.008
Q9UBS0	RPS6KB2	0.629	0.545	0.733	0.632	0.587	0.682	0.288	0.096

Q5TGY3	AHDC1	-1.057	-1.003	-1.189	-1.120	-1.030	-1.154	0.111	-0.125
Q9Y6M5	SLC30A1	1.151	1.071	1.165	1.098	1.111	1.131	0.736	0.020
Q14145	KEAP1	-0.357	-0.413	-0.546	-0.463	-0.385	-0.505	0.156	-0.120
Q03426	MVK	0.378	0.343	0.237	0.244	0.360	0.240	0.084	-0.120
Q5TC84	OGFRL1	-0.861	-1.016	-0.578	-0.724	-0.938	-0.651	0.115	0.288
Q2TAA5	ALG11	-1.446	-1.540	-1.485	-1.415	-1.493	-1.450	0.546	0.043
Q9NVD7	PARVA	2.535	2.545	2.435	2.411	2.540	2.423	0.035	-0.117
Q1W6H9	FAM110C	-7.415	-5.996	-6.887	-5.613	-6.705	-6.250	0.681	0.455
Q6NUQ1	RINT1	1.703	1.674	1.590	1.680	1.689	1.635	0.429	-0.054
Q99497	PARK7	5.970	5.858	6.011	5.972	5.914	5.991	0.386	0.077
Q9NS69	TOMM22	1.568	1.528	1.566	1.538	1.548	1.552	0.876	0.004
Q9BTV5	FSD1	0.992	0.963	0.824	0.870	0.977	0.847	0.058	-0.130
Q8NBP5	MFSD9	-3.575	-3.692	-3.708	-3.766	-3.633	-3.737	0.294	-0.104
Q9BZX2	UCK2	0.189	0.167	0.193	0.059	0.178	0.126	0.581	-0.052
Q99424	ACOX2	-2.515	-2.697	-2.505	-2.697	-2.606	-2.601	0.973	0.005
Q96IF1	AJUBA	-0.321	-0.059	-0.242	-0.001	-0.190	-0.121	0.737	0.069
P30520	ADSS2	3.491	3.476	3.484	3.425	3.484	3.454	0.497	-0.029
Q9NRN9	METTL5	0.760	0.711	0.738	0.653	0.735	0.695	0.518	-0.040
P14618	PKM	0.824	0.877	0.897	0.807	0.850	0.852	0.973	0.002
P53803	POLR2K	-1.773	-1.791	-1.820	-1.839	-1.782	-1.830	0.069	-0.048
P46939	UTRN	-3.535	-3.522	-3.737	-3.737	-3.529	-3.737	0.019	-0.208
Q9HCK8	CHD8	-1.082	-1.149	-1.243	-1.259	-1.115	-1.251	0.138	-0.136
Q13641	TPBG	-3.903	-3.617	-3.313	-3.568	-3.760	-3.440	0.238	0.320
O95159	ZFPL1	-0.658	-0.577	-0.614	-0.522	-0.618	-0.568	0.503	0.050
P07686	HEXB	4.995	4.953	4.899	4.889	4.974	4.894	0.148	-0.080
Q9H8W4	PLEKHF2	-0.780	-0.590	-0.731	-0.606	-0.685	-0.669	0.903	0.016
Q9H5K3	POMK	-1.392	-1.470	-1.359	-1.367	-1.431	-1.363	0.327	0.068
Q52LW3	ARHGAP29	1.638	1.724	1.779	1.768	1.681	1.774	0.273	0.093
O43175	PHGDH	4.639	4.781	4.788	4.726	4.710	4.757	0.626	0.047
Q8IUD2	ERC1	-0.891	-1.042	-0.876	-0.905	-0.967	-0.890	0.492	0.076
O00567	NOP56	5.523	5.466	5.612	5.583	5.494	5.598	0.121	0.103
Q86YM7	HOMER1	-3.286	-3.396	-3.323	-3.200	-3.341	-3.262	0.440	0.079
Q71F56	MED13L	-1.338	-1.357	-1.455	-1.316	-1.348	-1.386	0.680	-0.038
Q99536	VAT1	2.608	2.653	2.955	2.853	2.630	2.904	0.078	0.274
P82930	MRPS34	2.998	2.914	2.864	2.763	2.956	2.813	0.167	-0.143

P05091	ALDH2	-4.614	-2.599	-2.621	-3.074	-3.607	-2.848	0.587	0.759
Q9H7B2	RPF2	3.139	3.074	3.194	3.202	3.106	3.198	0.212	0.092
Q9NX40	OCIAD1	0.285	0.242	0.392	0.395	0.263	0.394	0.102	0.130
O95785	WIZ	-1.902	-1.819	-1.991	-1.890	-1.861	-1.941	0.349	-0.080
O95619	YEATS4	0.337	0.304	0.413	0.377	0.321	0.395	0.092	0.074
P17050	NAGA	0.605	0.366	0.705	0.499	0.486	0.602	0.539	0.117
Q8IW92	GLB1L2	-1.672	-1.655	-1.546	-1.520	-1.663	-1.533	0.021	0.130
P11142	HSPA8	5.854	5.820	5.795	5.749	5.837	5.772	0.164	-0.064
L0R6Q1	SLC35A4	0.636	0.562	0.451	0.514	0.599	0.483	0.141	-0.116
Q9H8Y5	ANKZF1	0.919	0.902	0.970	1.037	0.910	1.003	0.202	0.093
P15104	GLUL	-0.289	0.207	0.014	-0.209	-0.041	-0.098	0.862	-0.056
O94808	GFPT2	0.472	0.387	0.316	0.254	0.430	0.285	0.122	-0.145
Q9ULD5	ZNF777	-3.481	-3.478	-3.622	-3.525	-3.480	-3.574	0.303	-0.094
Q7Z6I8	C5orf24	-2.803	-2.864	-3.060	-3.033	-2.834	-3.046	0.055	-0.213
O95407	TNFRSF6B	-0.067	-0.127	-0.142	-0.033	-0.097	-0.087	0.893	0.010
Q75N03	CBLL1	-5.279	-5.360	-5.493	-5.283	-5.319	-5.388	0.634	-0.068
P00966	ASS1	3.288	3.588	3.401	3.426	3.438	3.414	0.898	-0.024
O95620	DUS4L	-3.888	-3.854	-3.655	-3.638	-3.871	-3.646	0.020	0.225
P47897	QARS1	-1.374	-1.722	-1.732	-1.650	-1.548	-1.691	0.559	-0.143
P04733	MT1F	0.841	0.713	0.957	0.915	0.777	0.936	0.217	0.159
Q8WW59	SPRYD4	1.173	1.170	1.100	1.145	1.172	1.123	0.270	-0.049
Q13043	STK4	-1.377	-1.396	-1.627	-1.574	-1.387	-1.600	0.053	-0.214
Q9H7F4	TMEM185B	-2.957	-3.002	-2.917	-2.759	-2.980	-2.838	0.307	0.142
Q9Y2R9	MRPS7	3.357	3.416	3.262	3.260	3.386	3.261	0.149	-0.125
A6NKD9	CCDC85C	0.145	0.184	-0.073	-0.044	0.164	-0.059	0.015	-0.223
Q9Y519	TMEM184B	-2.437	-2.375	-2.334	-2.359	-2.406	-2.347	0.275	0.059
Q9P260	RELCH	-2.101	-2.590	-2.261	-2.688	-2.345	-2.474	0.730	-0.129
O14979	HNRNPDL	-0.787	-0.817	-0.772	-0.700	-0.802	-0.736	0.288	0.066
P49207	RPL34	6.216	6.158	6.195	6.136	6.187	6.166	0.655	-0.022
Q96EK6	GNPNAT1	2.033	2.029	1.892	1.814	2.031	1.853	0.137	-0.178
Q9H4A4	RNPEP	4.605	4.639	4.596	4.597	4.622	4.596	0.379	-0.025
P85037	FOXK1	0.725	0.785	0.826	0.796	0.755	0.811	0.282	0.056
Q13610	PWP1	2.034	1.854	1.939	1.816	1.944	1.877	0.611	-0.067
Q9H7F0	ATP13A3	-2.378	-2.302	-2.597	-2.394	-2.340	-2.495	0.347	-0.155
Q9UKN8	GTF3C4	3.149	3.160	3.121	3.245	3.155	3.183	0.724	0.029

O95926	SYF2	-0.266	-0.208	-0.151	-0.204	-0.237	-0.177	0.267	0.060
Q7KZF4	SND1	5.963	5.906	5.956	5.926	5.935	5.941	0.872	0.006
Q96I36	COX14	-1.406	-1.232	-1.316	-1.272	-1.319	-1.294	0.823	0.025
Q8TF50	ZNF526	-2.192	-2.157	-2.151	-2.126	-2.174	-2.138	0.253	0.036
Q96AA3	RFT1	0.298	0.267	0.309	0.324	0.283	0.317	0.236	0.034
O43149	ZZEF1	0.533	0.443	0.436	0.449	0.488	0.442	0.496	-0.045
Q9H8P0	SRD5A3	-0.463	-0.502	-0.471	-0.475	-0.482	-0.473	0.721	0.009
O43402	EMC8	-0.488	-0.575	-0.551	-0.513	-0.532	-0.532	0.999	0.000
Q99541	PLIN2	2.268	2.187	2.636	2.675	2.228	2.655	0.028	0.427
Q08174	PCDH1	-1.948	-1.922	-2.306	-2.143	-1.935	-2.224	0.167	-0.289
Q7Z7K6	CENPV	-3.472	-3.623	-3.495	-3.500	-3.548	-3.498	0.629	0.050
P38646	HSPA9	7.212	7.153	7.293	7.267	7.183	7.280	0.148	0.097
Q9NPA0	EMC7	1.677	1.686	1.906	1.768	1.681	1.837	0.264	0.156
P23677	ITPKA	-2.058	-2.112	-2.044	-2.029	-2.085	-2.037	0.310	0.048
Q15181	PPA1	6.381	6.544	6.321	6.358	6.463	6.340	0.363	-0.123
Q5VWG9	TAF3	-0.083	-0.139	-0.182	-0.152	-0.111	-0.167	0.257	-0.056
O75880	SCO1	2.719	2.857	2.873	2.950	2.788	2.911	0.293	0.123
Q9HCC0	MCCC2	-0.004	-0.006	0.106	0.072	-0.005	0.089	0.113	0.094
Q9P253	VPS18	1.072	0.956	1.055	1.011	1.014	1.033	0.806	0.019
Q9Y2W2	WBP11	3.272	3.231	3.256	3.268	3.252	3.262	0.710	0.010
Q15582	TGFBI	-3.202	-3.245	-3.408	-3.315	-3.223	-3.362	0.164	-0.138
P42167	TMPO	1.820	1.769	1.849	1.871	1.795	1.860	0.197	0.066
Q15631	TSN	3.153	3.094	3.098	3.032	3.123	3.065	0.319	-0.058
O60762	DPM1	3.060	3.046	3.003	3.026	3.053	3.015	0.133	-0.038
Q96CM3	RPUSD4	-2.951	-2.954	-2.942	-2.854	-2.952	-2.898	0.434	0.054
Q96EB1	ELP4	-0.259	-0.348	-0.285	-0.337	-0.304	-0.311	0.903	-0.007
P48651	PTDSS1	2.017	2.019	2.047	2.011	2.018	2.029	0.645	0.011
Q6NUM9	RETSAT	0.400	0.304	0.411	0.419	0.352	0.415	0.412	0.063
Q14CX7	NAA25	-1.435	-1.576	-1.748	-1.659	-1.505	-1.703	0.164	-0.198
Q9NYH9	UTP6	3.184	3.129	3.230	3.212	3.157	3.221	0.232	0.065
Q13686	ALKBH1	-0.589	-0.765	-0.706	-0.840	-0.677	-0.773	0.483	-0.096
Q9HCG8	CWC22	2.563	2.558	2.610	2.547	2.561	2.578	0.673	0.018
Q9UKL3	CASP8AP2	0.155	0.126	-0.006	0.042	0.140	0.018	0.069	-0.122
Q7LBR1	CHMP1B	1.589	1.626	1.759	1.731	1.608	1.745	0.033	0.137
Q01459	CTBS	0.007	0.007	0.179	0.265	0.007	0.222	0.125	0.215

O75964	ATP5MG	3.469	3.404	3.382	3.443	3.436	3.413	0.643	-0.024
P42224	STAT1	-0.910	-0.903	-0.790	-0.777	-0.906	-0.783	0.010	0.123
Q9H089	LSG1	2.603	2.623	2.602	2.576	2.613	2.589	0.290	-0.024
Q8N9M1	C19orf47	-4.222	-4.245	-4.430	-4.060	-4.233	-4.245	0.960	-0.012
P35080	PFN2	2.202	2.334	1.707	1.974	2.268	1.841	0.146	-0.428
Q9UPT6	MAPK8IP3	-2.472	-2.474	-2.452	-2.287	-2.473	-2.369	0.428	0.103
Q14376	GALE	0.952	0.808	0.798	0.803	0.880	0.800	0.470	-0.080
Q96GS4	BORCS6	-1.126	-1.168	-1.084	-1.103	-1.147	-1.094	0.202	0.053
Q5T5X7	BEND3	-0.285	-0.328	-0.379	-0.369	-0.307	-0.374	0.176	-0.067
Q96N64	PWWP2A	-1.863	-1.895	-2.000	-1.802	-1.879	-1.901	0.865	-0.021
Q8N0T1	RBIS	-0.390	-0.345	-0.175	-0.189	-0.367	-0.182	0.057	0.185
Q86U28	ISCA2	1.438	1.406	1.245	1.347	1.422	1.296	0.220	-0.126
Q96I15	SCLY	-0.959	-1.092	-1.025	-1.137	-1.025	-1.081	0.588	-0.056
P50748	KNTC1	3.002	2.976	2.824	2.844	2.989	2.834	0.014	-0.155
P00533	EGFR	2.135	2.200	2.010	1.936	2.167	1.973	0.059	-0.194
Q8NB90	SPATA5	-0.959	-1.111	-1.083	-1.164	-1.035	-1.123	0.440	-0.088
Q9Y696	CLIC4	3.134	3.055	3.093	3.044	3.095	3.068	0.635	-0.027
P46013	MKI67	2.049	2.088	1.963	1.951	2.068	1.957	0.087	-0.111
P15153	RAC2	-2.473	-2.342	-1.762	-2.406	-2.407	-2.084	0.495	0.323
Q5VYY1	ANKRD22	0.845	0.871	1.011	0.777	0.858	0.894	0.809	0.036
Q9BTE6	AARSD1	-3.086	-3.080	-3.141	-3.101	-3.083	-3.121	0.301	-0.038
Q96AZ6	ISG20	-2.131	-2.226	-1.935	-2.083	-2.179	-2.009	0.215	0.170
P13693	TPT1	4.743	4.751	4.710	4.679	4.747	4.694	0.162	-0.052
Q96RR1	TWNK	-2.581	-2.490	-2.412	-2.345	-2.535	-2.378	0.120	0.157
P08572	COL4A2	-5.441	-5.498	-5.496	-5.301	-5.470	-5.399	0.599	0.071
Q99471	PFDN5	-0.298	-0.384	-0.439	-1.100	-0.341	-0.769	0.416	-0.428
Q6P1L5	FAM117B	-3.025	-2.920	-3.072	-2.835	-2.972	-2.954	0.902	0.019
P35269	GTF2F1	2.541	2.517	2.582	2.568	2.529	2.575	0.105	0.046
P35270	SPR	3.906	3.875	4.079	4.010	3.891	4.045	0.099	0.154
P49711	CTCF	-1.617	-1.681	-1.539	-1.684	-1.649	-1.612	0.702	0.037
Q13158	FADD	-0.657	-0.596	-0.677	-0.617	-0.626	-0.647	0.680	-0.021
Q96E14	RMI2	-1.902	-1.904	-1.991	-1.919	-1.903	-1.955	0.384	-0.052
Q15459	SF3A1	1.697	1.638	1.804	1.713	1.668	1.759	0.256	0.091
Q13613	MTMR1	-1.361	-1.901	-1.477	-2.182	-1.631	-1.830	0.701	-0.199
P03897	MT	-3.437	-3.456	-3.430	-3.400	-3.446	-3.415	0.243	0.031

Q8N5P1	ZC3H8	0.362	0.440	0.489	0.479	0.401	0.484	0.274	0.083
O15269	SPTLC1	2.178	2.221	2.364	2.338	2.200	2.351	0.042	0.151
O60568	PLOD3	4.066	3.989	4.213	4.227	4.027	4.220	0.117	0.192
O75044	SRGAP2	1.211	1.039	1.151	1.130	1.125	1.140	0.887	0.015
Q9UI43	MRM2	-1.103	-1.059	-1.213	-1.153	-1.081	-1.183	0.123	-0.102
Q01658	DR1	1.318	1.278	1.325	1.325	1.298	1.325	0.405	0.027
Q8IUH4	ZDHHC13	-3.183	-3.128	-3.073	-2.971	-3.155	-3.022	0.185	0.133
Q9HCE5	METTL14	0.777	0.715	0.881	0.847	0.746	0.864	0.112	0.118
Q96G21	IMP4	2.657	2.612	2.709	2.707	2.634	2.708	0.189	0.074
Q86UD0	SAPCD2	-0.818	-0.698	-1.072	-0.860	-0.758	-0.966	0.261	-0.208
O14896	IRF6	-3.540	-3.138	-3.411	-3.121	-3.339	-3.266	0.799	0.073
Q8N6M3	FITM2	-1.851	-1.736	-1.844	-1.748	-1.793	-1.796	0.973	-0.003
Q9Y2A9	B3GNT3	-4.517	-4.029	-4.254	-3.898	-4.273	-4.076	0.586	0.197
Q4W5G0	TIGD2	-2.767	-2.603	-2.658	-2.597	-2.685	-2.627	0.608	0.058
Q07352	ZFP36L1	0.818	0.713	0.532	0.523	0.766	0.527	0.136	-0.238
P78504	JAG1	-3.753	-4.092	-4.056	-4.152	-3.923	-4.104	0.471	-0.182
O14678	ABCD4	-2.699	-2.617	-2.546	-2.365	-2.658	-2.455	0.231	0.202
P46199	MTIF2	2.102	1.908	2.147	2.019	2.005	2.083	0.581	0.078
Q9H0U9	TSPYL1	-0.270	-0.241	0.022	0.044	-0.256	0.033	0.005	0.289
Q9UNZ5	C19orf53	1.842	1.785	1.824	1.851	1.813	1.838	0.547	0.024
Q13200	PSMD2	2.869	2.839	2.824	2.870	2.854	2.847	0.822	-0.007
Q6DT37	CDC42BPG	-1.085	-0.840	-0.952	-0.964	-0.963	-0.958	0.976	0.005
O60645	EXOC3	1.547	1.477	1.498	1.463	1.512	1.481	0.538	-0.031
Q9H1A4	ANAPC1	2.477	2.390	2.454	2.404	2.433	2.429	0.947	-0.004
Q9H7D0	DOCK5	0.926	0.979	0.952	0.924	0.952	0.938	0.688	-0.015
Q96S44	TP53RK	0.890	1.028	1.018	1.040	0.959	1.029	0.495	0.070
Q10472	GALNT1	-0.274	-0.295	-0.469	-0.394	-0.284	-0.432	0.136	-0.147
P43405	SYK	-6.377	-5.665	-6.032	-6.037	-6.021	-6.035	0.975	-0.014
O43681	GET3	4.740	4.646	4.587	4.642	4.693	4.614	0.315	-0.079
P62495	ETF1	1.394	1.281	1.260	1.297	1.337	1.279	0.478	-0.059
P53602	MVD	1.622	1.535	1.573	1.551	1.579	1.562	0.774	-0.016
Q96FN4	CPNE2	-0.748	-0.753	-0.568	-0.672	-0.751	-0.620	0.241	0.131
P03905	MT	-0.823	-0.916	-0.887	-0.840	-0.869	-0.863	0.920	0.006
Q8N8A6	DDX51	2.022	1.952	2.008	1.957	1.987	1.983	0.926	-0.005
O95429	BAG4	-6.046	-5.942	-6.009	-6.092	-5.994	-6.050	0.489	-0.057

O14544	SOCS6	-0.463	-0.587	-0.847	-0.852	-0.525	-0.850	0.119	-0.325
O75607	NPM3	0.544	0.361	0.499	0.496	0.452	0.497	0.709	0.045
P06493	CDK1	2.905	2.961	2.547	2.479	2.933	2.513	0.012	-0.420
Q9Y2D0	CA5B	-4.256	-4.167	-3.991	-3.908	-4.211	-3.950	0.051	0.261
O75533	SF3B1	6.096	6.067	6.052	6.022	6.082	6.037	0.168	-0.044
Q13751	LAMB3	-0.566	-0.367	-0.376	-0.432	-0.466	-0.404	0.643	0.062
Q15637	SF1	-0.417	-0.449	-0.526	-0.379	-0.433	-0.453	0.834	-0.020
P01024	C3	0.345	0.347	0.654	0.368	0.346	0.511	0.455	0.165
Q15003	NCAPH	2.072	1.977	1.838	1.796	2.025	1.817	0.103	-0.207
P62244	RPS15A	6.136	6.021	6.219	6.060	6.078	6.140	0.602	0.061
Q5VV41	ARHGEF16	-0.299	0.033	-0.095	-0.150	-0.133	-0.123	0.959	0.011
Q9HD42	CHMP1A	1.976	1.787	2.016	1.889	1.882	1.952	0.604	0.071
O00116	AGPS	3.499	3.459	3.530	3.489	3.479	3.509	0.403	0.030
P48634	PRRC2A	-2.017	-1.934	-1.818	-1.568	-1.976	-1.693	0.241	0.282
Q5VYV7	SLX4IP	-3.096	-3.034	-3.142	-3.044	-3.065	-3.093	0.687	-0.028
Q5TFE4	NT5DC1	0.274	0.292	0.319	0.410	0.283	0.364	0.318	0.081
Q13618	CUL3	-5.379	-4.982	-5.498	-5.111	-5.181	-5.304	0.700	-0.124
Q9P0S9	TMEM14C	-0.537	-0.518	-0.535	-0.415	-0.527	-0.475	0.543	0.052
Q99614	TTC1	4.112	4.082	4.050	4.060	4.097	4.055	0.196	-0.042
O75717	WDHD1	1.977	1.966	1.700	1.682	1.971	1.691	0.003	-0.280
Q86VU5	COMTD1	-0.476	-0.306	-0.323	-0.189	-0.391	-0.256	0.345	0.135
Q9UEG4	ZNF629	0.039	-0.010	0.064	0.061	0.015	0.062	0.300	0.048
Q9Y3D2	MSRB2	-0.299	-0.390	-0.215	-0.211	-0.345	-0.213	0.210	0.132
Q8NCD3	HJURP	-2.613	-2.578	-2.691	-2.599	-2.595	-2.645	0.464	-0.050
Q96QC0	PPP1R10	3.068	2.998	3.035	2.967	3.033	3.001	0.578	-0.032
P22570	FDXR	-1.159	-1.173	-1.184	-1.167	-1.166	-1.176	0.471	-0.009
Q9NRF9	POLE3	0.698	0.616	0.531	0.500	0.657	0.515	0.146	-0.141
Q92882	OSTF1	0.002	0.317	0.166	0.134	0.160	0.150	0.962	-0.009
P04818	TYMS	-3.311	-3.265	-4.693	-4.630	-3.288	-4.662	0.001	-1.374
Q9UKD1	GMEB2	-1.628	-1.579	-1.570	-1.536	-1.603	-1.553	0.251	0.051
Q01081	U2AF1	-1.486	-1.500	-1.437	-1.436	-1.493	-1.436	0.077	0.057
Q01831	XPC	-2.017	-1.993	-2.009	-1.979	-2.005	-1.994	0.638	0.011
Q9H8G2	CAAP1	0.096	0.064	0.173	0.068	0.080	0.121	0.577	0.041
Q9H6S3	EPS8L2	-3.733	-3.551	-3.760	-3.796	-3.642	-3.778	0.370	-0.136
P47944	MT4	-3.761	-3.862	-3.942	-3.957	-3.812	-3.949	0.219	-0.138

P09327	VIL1	-1.913	-1.523	-1.876	-1.646	-1.718	-1.761	0.871	-0.043
Q9Y6I8	PXMP4	-0.940	-1.004	-1.042	-0.993	-0.972	-1.017	0.381	-0.046
Q8N357	SLC35F6	-0.659	-0.592	-0.543	-0.436	-0.626	-0.490	0.187	0.136
Q6P1M3	LLGL2	-3.766	-3.316	-3.467	-3.426	-3.541	-3.446	0.746	0.095
Q9NS93	TM7SF3	-2.957	-2.735	-2.964	-2.852	-2.846	-2.908	0.683	-0.062
Q9UFW8	CGGBP1	-0.755	-0.705	-0.662	-0.683	-0.730	-0.672	0.223	0.058
P0C2W1	FBXO45	-0.424	-0.444	-0.570	-0.423	-0.434	-0.496	0.548	-0.062
Q9NX07	TRNAU1AP	-0.727	-0.746	-0.815	-0.777	-0.737	-0.796	0.152	-0.059
Q9H788	SH2D4A	-3.864	-2.637	-2.860	-3.032	-3.251	-2.946	0.706	0.305
Q9H8M2	BRD9	-1.994	-1.986	-1.915	-2.055	-1.990	-1.985	0.949	0.006
Q14558	PRPSAP1	-2.369	-2.374	-2.470	-2.254	-2.372	-2.362	0.945	0.009
Q96AH8	RAB7B	-2.764	-2.594	-2.681	-2.599	-2.679	-2.640	0.733	0.039
O94760	DDAH1	1.310	1.269	1.222	1.332	1.290	1.277	0.856	-0.013
Q9NYM9	BET1L	-1.081	-1.014	-1.056	-1.039	-1.047	-1.047	0.998	0.000
Q9NQ55	PPAN	-0.463	-0.497	-0.438	-0.411	-0.480	-0.424	0.128	0.056
P19419	ELK1	-1.839	-1.939	-1.889	-1.858	-1.889	-1.874	0.808	0.016
Q9NUJ3	TCP11L1	-3.336	-3.171	-3.419	-3.134	-3.253	-3.277	0.904	-0.023
Q9UNN5	FAF1	0.534	0.582	0.497	0.662	0.558	0.579	0.839	0.021
Q96PE2	ARHGEF17	-0.044	-0.055	-0.085	0.006	-0.050	-0.039	0.860	0.010
O95391	SLU7	1.295	1.214	1.478	1.350	1.254	1.414	0.193	0.160
Q8TD30	GPT2	-0.620	-0.549	-0.418	-0.567	-0.584	-0.493	0.419	0.092
O43187	IRAK2	-3.748	-3.775	-3.637	-3.762	-3.762	-3.699	0.495	0.063
Q14684	RRP1B	-0.977	-1.091	-1.142	-1.261	-1.034	-1.202	0.179	-0.168
P01034	CST3	-2.446	-2.384	-2.333	-2.227	-2.415	-2.280	0.190	0.135
Q86X53	ERICH1	-3.904	-4.126	-4.419	-3.903	-4.015	-4.161	0.675	-0.146
Q53RE8	ANKRD39	-3.381	-3.163	-3.840	-3.207	-3.272	-3.523	0.570	-0.251
Q8NEN9	PDZD8	0.025	0.083	-0.046	0.062	0.054	0.008	0.550	-0.046
Q9H467	CUEDC2	-0.373	-0.512	-0.159	-0.272	-0.443	-0.215	0.131	0.228
Q9HBU6	ETNK1	0.352	0.351	0.123	0.204	0.352	0.164	0.136	-0.188
O43172	PRPF4	-2.806	-3.549	-2.718	-3.466	-3.178	-3.092	0.886	0.086
Q9Y4B5	MTCL1	-0.188	-0.301	-0.244	-0.261	-0.244	-0.252	0.909	-0.008
Q9UPY8	MAPRE3	-1.963	-1.806	-1.842	-1.793	-1.884	-1.818	0.547	0.067
P14868	DARS1	2.524	2.432	2.483	2.427	2.478	2.455	0.712	-0.024
P09234	SNRPC	3.265	3.314	3.296	3.408	3.289	3.352	0.453	0.063
P47813	EIF1AX	-0.434	-0.685	-0.607	-0.738	-0.560	-0.672	0.532	-0.113

Q92947	GCDH	-2.771	-2.639	-2.696	-2.566	-2.705	-2.631	0.510	0.073
P51571	SSR4	3.932	3.885	3.823	3.828	3.909	3.826	0.171	-0.083
Q86V88	MDP1	-0.636	-0.497	-0.626	-0.676	-0.567	-0.651	0.425	-0.084
Q9BVV7	TIMM21	0.348	0.417	0.316	0.311	0.382	0.313	0.296	-0.069
Q13084	MRPL28	3.784	3.685	3.751	3.680	3.735	3.716	0.791	-0.019
P01011	SERPINA3	-0.165	-0.033	0.390	0.377	-0.099	0.384	0.084	0.483
P21579	SYT1	0.310	0.205	-0.215	-0.150	0.258	-0.183	0.030	-0.440
Q6UW63	POGLUT2	-1.881	-1.811	-1.909	-1.862	-1.846	-1.885	0.457	-0.040
Q9H9A7	RMI1	-1.162	-1.146	-1.342	-1.314	-1.154	-1.328	0.019	-0.174
Q8N5I4	DHRSX	-1.429	-1.393	-1.503	-1.353	-1.411	-1.428	0.861	-0.017
Q9Y6D9	MAD1L1	-1.269	-1.241	-1.211	-1.245	-1.255	-1.228	0.340	0.028
Q969Q0	RPL36AL	-0.250	-0.448	-0.222	-0.354	-0.349	-0.288	0.663	0.062
Q09161	NCBP1	3.905	3.889	3.854	3.759	3.897	3.806	0.301	-0.091
Q08378	GOLGA3	-1.709	-1.740	-1.773	-1.821	-1.724	-1.797	0.145	-0.072
P30046	DDT	3.360	2.941	3.242	2.984	3.151	3.113	0.895	-0.038
Q9Y3T9	NOC2L	4.177	4.165	4.240	4.232	4.171	4.236	0.017	0.064
Q92613	JADE3	-0.125	-0.120	-0.064	-0.085	-0.122	-0.074	0.121	0.048
Q96C01	FAM136A	3.839	3.691	3.889	3.835	3.765	3.862	0.399	0.097
P12004	PCNA	6.242	6.223	5.901	5.843	6.233	5.872	0.035	-0.361
O00461	GOLIM4	1.492	1.458	1.398	1.434	1.475	1.416	0.143	-0.059
Q6P3X3	TTC27	1.803	1.797	1.635	1.656	1.800	1.646	0.030	-0.154
Q969Q6	PPP2R3C	-2.294	-2.445	-2.387	-2.326	-2.369	-2.356	0.896	0.013
P15924	DSP	2.040	2.321	2.212	2.072	2.180	2.142	0.836	-0.039
P35548	MSX2	-5.272	-4.729	-5.309	-5.233	-5.001	-5.271	0.499	-0.271
Q9BZL1	UBL5	1.176	1.153	1.243	1.186	1.165	1.214	0.306	0.050
Q9NX63	CHCHD3	3.652	3.560	3.779	3.713	3.606	3.746	0.143	0.140
P04181	OAT	3.009	3.038	2.851	2.904	3.023	2.877	0.066	-0.146
L0R8F8	MIEF1	-1.289	-1.302	-1.338	-1.296	-1.296	-1.317	0.483	-0.022
P10599	TXN	2.658	2.588	2.671	2.603	2.623	2.637	0.795	0.014
P52907	CAPZA1	3.954	3.871	3.764	3.783	3.912	3.774	0.172	-0.138
Q96HE7	ERO1A	4.576	4.533	4.717	4.653	4.554	4.685	0.093	0.131
Q96DB5	RMDN1	-2.765	-2.897	-2.844	-2.794	-2.831	-2.819	0.887	0.012
Q9BTY2	FUCA2	-0.043	0.041	0.000	0.048	-0.001	0.024	0.666	0.025
Q15004	PCLAF	-2.029	-1.781	-1.921	-2.035	-1.905	-1.978	0.666	-0.073
P17900	GM2A	0.105	0.055	0.000	0.001	0.080	0.000	0.194	-0.080

P00505	GOT2	5.088	5.063	5.079	5.086	5.075	5.082	0.683	0.007
P54578	USP14	-0.300	-0.189	-0.339	-0.339	-0.245	-0.339	0.337	-0.094
Q9H7M9	VSIR	-3.196	-3.290	-3.106	-3.311	-3.243	-3.209	0.803	0.034
P51114	FXR1	-4.771	-4.373	-4.410	-4.430	-4.572	-4.420	0.584	0.152
Q96MF7	NSMCE2	0.312	0.294	0.242	0.279	0.303	0.260	0.223	-0.043
Q96JM3	CHAMP1	2.773	2.736	2.804	2.782	2.754	2.793	0.246	0.038
Q6PML9	SLC30A9	0.649	0.632	0.694	0.731	0.640	0.712	0.118	0.072
O15304	SIVA1	-2.584	-2.446	-2.554	-2.364	-2.515	-2.459	0.682	0.056
Q9BZE4	GTPBP4	2.263	2.365	2.190	2.298	2.314	2.244	0.449	-0.070
Q9Y5Q9	GTF3C3	1.543	1.546	1.512	1.605	1.544	1.558	0.810	0.014
Q9NT62	ATG3	-2.275	-2.278	-2.514	-2.547	-2.276	-2.530	0.041	-0.254
Q12882	DPYD	2.192	2.079	2.232	2.240	2.136	2.236	0.327	0.100
Q99719	SEPTIN5	-0.590	-0.653	-0.743	-0.757	-0.622	-0.750	0.141	-0.129
Q96GA3	LTV1	1.476	1.475	1.538	1.512	1.476	1.525	0.158	0.049
Q96A19	CCDC102A	-0.396	-0.430	-0.622	-0.551	-0.413	-0.587	0.084	-0.174
Q9HB19	PLEKHA2	0.986	0.898	0.923	0.833	0.942	0.878	0.415	-0.064
O60551	NMT2	3.352	3.261	3.258	3.265	3.306	3.261	0.504	-0.045
P51153	RAB13	2.124	2.046	1.813	1.827	2.085	1.820	0.084	-0.265
Q8IWF2	FOXRED2	-3.390	-3.508	-3.712	-3.670	-3.449	-3.691	0.123	-0.242
Q9Y2E4	DIP2C	-4.278	-4.389	-4.242	-4.182	-4.333	-4.212	0.228	0.121
O94876	TMCC1	-2.795	-2.736	-2.816	-2.793	-2.765	-2.804	0.393	-0.039
Q8NE62	CHDH	1.691	1.863	1.786	1.796	1.777	1.791	0.899	0.014
Q9UGM6	WARS2	1.090	0.996	1.198	1.146	1.043	1.172	0.174	0.129
P30049	ATP5F1D	0.290	0.191	0.315	0.339	0.241	0.327	0.323	0.086
O94964	SOGA1	-4.106	-4.207	-4.361	-4.439	-4.157	-4.400	0.069	-0.243
Q99879	H2BC14	2.913	2.554	3.007	2.646	2.733	2.827	0.749	0.093
Q04828	AKR1C1	-1.595	-1.466	-1.497	-1.418	-1.530	-1.457	0.456	0.073
P55036	PSMD4	2.493	2.477	2.493	2.462	2.485	2.478	0.735	-0.007
Q96F86	EDC3	1.631	1.563	1.599	1.574	1.597	1.587	0.809	-0.011
Q8WVC6	DCAKD	0.463	0.426	0.188	0.281	0.444	0.234	0.102	-0.210
P61916	NPC2	1.807	1.983	2.074	1.943	1.895	2.009	0.417	0.114
Q9H845	ACAD9	4.513	4.374	4.487	4.424	4.444	4.455	0.896	0.012
O75886	STAM2	-1.296	-1.198	-1.497	-1.487	-1.247	-1.492	0.123	-0.245
Q9UNA1	ARHGAP26	-3.879	-4.192	-3.848	-3.859	-4.035	-3.854	0.453	0.182
O94887	FARP2	-3.878	-3.915	-4.007	-3.895	-3.897	-3.951	0.504	-0.054

Q96JJ3	ELMO2	-1.112	-1.053	-1.075	-1.136	-1.082	-1.105	0.643	-0.023
Q8N4Q0	ZADH2	-0.935	-1.009	-0.827	-0.851	-0.972	-0.839	0.146	0.133
Q5VW36	FOCAD	-2.683	-2.529	-2.721	-2.524	-2.606	-2.623	0.906	-0.017
P43246	MSH2	0.646	0.679	0.524	0.606	0.662	0.565	0.223	-0.097
Q9BV57	ADI1	3.501	3.460	3.421	3.540	3.481	3.480	0.997	0.000
Q99543	DNAJC2	0.986	0.955	0.995	0.937	0.971	0.966	0.905	-0.005
P0DJ07	PET100	-0.549	-0.539	-0.743	-0.830	-0.544	-0.787	0.109	-0.243
P61619	SEC61A1	2.083	2.012	1.995	2.183	2.048	2.089	0.742	0.041
Q9Y5V0	ZNF706	-0.642	-0.554	-0.599	-0.539	-0.598	-0.569	0.649	0.029
P15056	BRAF	-1.712	-1.504	-1.763	-1.480	-1.608	-1.621	0.946	-0.014
O75947	ATP5PD	1.615	1.487	1.497	1.427	1.551	1.462	0.378	-0.089
Q9Y296	TRAPPC4	-1.809	-1.814	-1.790	-1.750	-1.812	-1.770	0.286	0.041
Q96AY3	FKBP10	3.633	3.455	3.549	3.431	3.544	3.490	0.670	-0.054
Q8IWU2	LMTK2	-0.833	-0.715	-0.518	-0.454	-0.774	-0.486	0.077	0.288
Q96KR6	FAM210B	-0.783	-0.762	-0.777	-0.829	-0.772	-0.803	0.433	-0.030
Q9H7C9	AAMDC	0.358	0.199	0.468	0.433	0.279	0.451	0.264	0.172
Q8IY47	KBTBD2	-0.657	-0.658	-0.823	-0.727	-0.657	-0.775	0.246	-0.118
P00374	DHFR	-0.765	-1.001	-1.188	-1.212	-0.883	-1.200	0.224	-0.317
O14777	NDC80	1.875	1.841	1.630	1.612	1.858	1.621	0.016	-0.238
Q9UPR3	SMG5	-0.182	-0.133	-0.235	-0.180	-0.158	-0.207	0.309	-0.050
O60232	ZNRD2	1.520	1.405	1.364	1.255	1.462	1.310	0.195	-0.153
O00303	EIF3F	4.988	4.909	4.873	4.843	4.948	4.858	0.233	-0.090
O94992	HEXIM1	1.879	1.791	1.842	1.797	1.835	1.820	0.797	-0.015
Q9NPI1	BRD7	-3.901	-4.029	-4.300	-3.761	-3.965	-4.031	0.849	-0.065
O00483	NDUFA4	2.922	2.905	2.909	2.840	2.913	2.874	0.452	-0.039
Q8N2W9	PIAS4	-1.845	-1.880	-1.915	-1.899	-1.863	-1.907	0.193	-0.045
A6NDB9	PALM3	-4.143	-4.375	-4.374	-4.292	-4.259	-4.333	0.639	-0.074
Q86XJ1	GAS2L3	-1.203	-1.354	-1.348	-1.225	-1.279	-1.286	0.945	-0.008
P23743	DGKA	0.512	0.744	0.437	0.463	0.628	0.450	0.366	-0.178
O96018	APBA3	-2.165	-2.227	-2.186	-2.209	-2.196	-2.197	0.971	-0.001
Q99595	TIMM17A	-4.524	-4.555	-4.359	-4.556	-4.540	-4.458	0.558	0.082
Q7L2J0	MEPCE	0.961	0.942	1.128	1.125	0.952	1.126	0.032	0.175
Q8N567	ZCCHC9	-0.140	-0.123	-0.030	0.041	-0.132	0.005	0.144	0.137
Q643R3	LPCAT4	0.444	0.343	0.419	0.385	0.394	0.402	0.891	0.009
Q15005	SPCS2	2.531	2.468	2.471	2.473	2.500	2.472	0.539	-0.028

Q96AB6	NTAN1	-1.376	-1.525	-1.338	-1.355	-1.451	-1.347	0.392	0.104
Q6N069	NAA16	-0.968	-1.061	-1.086	-1.085	-1.015	-1.086	0.369	-0.071
P07948	LYN	-2.560	-2.540	-2.497	-2.498	-2.550	-2.498	0.124	0.052
Q99470	SDF2	2.329	2.378	2.418	2.391	2.354	2.404	0.243	0.051
Q9H6T0	ESRP2	-5.208	-4.482	-4.994	-4.711	-4.845	-4.852	0.988	-0.007
Q9HAU5	UPF2	3.019	2.991	2.912	2.921	3.005	2.917	0.075	-0.088
O43156	TTI1	1.309	1.341	1.191	1.158	1.325	1.175	0.022	-0.150
Q9NQC3	RTN4	-2.493	-2.501	-2.696	-2.801	-2.497	-2.749	0.130	-0.252
P55769	SNU13	3.934	3.852	3.927	3.934	3.893	3.931	0.528	0.037
P62841	RPS15	4.687	4.632	4.763	4.572	4.660	4.668	0.947	0.008
Q5VW32	BROX	-1.383	-1.400	-1.386	-1.408	-1.392	-1.397	0.739	-0.005
O15144	ARPC2	5.970	6.030	6.006	6.011	6.000	6.009	0.822	0.009
Q9NWU5	MRPL22	-0.243	-0.332	-0.120	-0.168	-0.288	-0.144	0.140	0.144
Q8TAE6	PPP1R14C	-6.201	-6.399	-6.523	-6.320	-6.300	-6.422	0.483	-0.121
P21399	ACO1	3.773	3.779	3.883	3.830	3.776	3.857	0.197	0.081
Q5T9A4	ATAD3B	-2.600	-2.962	-2.496	-2.992	-2.781	-2.744	0.916	0.037
Q8WV22	NSMCE1	0.930	0.937	0.915	0.908	0.933	0.912	0.047	-0.022
P55145	MANF	2.733	2.807	2.671	2.683	2.770	2.677	0.233	-0.093
O14683	TP53I11	-4.353	-4.625	-4.119	-4.423	-4.489	-4.271	0.398	0.218
Q6ZN28	MACC1	-2.015	-1.718	-2.043	-1.711	-1.866	-1.877	0.967	-0.011
P17482	HOXB9	-0.566	-0.831	-0.610	-0.710	-0.699	-0.660	0.823	0.039
Q8TDN6	BRIX1	4.140	3.994	4.117	4.033	4.067	4.075	0.936	0.008
P60510	PPP4C	1.248	1.238	0.967	1.059	1.243	1.013	0.121	-0.230
Q92621	NUP205	5.149	5.039	5.083	5.051	5.094	5.067	0.713	-0.027
Q9BRD0	BUD13	-1.407	-1.446	-1.448	-1.292	-1.426	-1.370	0.601	0.056
P35527	KRT9	-1.442	-1.491	-2.343	-2.080	-1.466	-2.211	0.102	-0.745
Q9ULA0	DNPEP	-0.788	-0.860	-0.627	-0.754	-0.824	-0.691	0.242	0.133
Q9BPW8	NIPSNAP1	3.561	3.560	3.644	3.717	3.561	3.681	0.189	0.120
O15554	KCNN4	-1.151	-1.174	-1.114	-1.107	-1.163	-1.111	0.113	0.052
P84095	RHOG	2.296	2.219	2.242	2.222	2.257	2.232	0.628	-0.025
P02786	TFRC	6.789	6.763	6.472	6.480	6.776	6.476	0.017	-0.300
Q99584	S100A13	2.324	2.518	2.436	2.338	2.421	2.387	0.793	-0.034
Q15050	RRS1	2.994	2.880	2.988	2.927	2.937	2.958	0.785	0.021
Q92604	LPGAT1	-1.619	-1.654	-1.544	-1.617	-1.636	-1.581	0.345	0.056
Q9UF56	FBXL17	-5.866	-5.818	-6.207	-5.956	-5.842	-6.081	0.299	-0.240

O60563	CCNT1	0.866	0.750	0.955	0.934	0.808	0.945	0.248	0.137
P35573	AGL	-0.714	-0.850	-0.910	-0.630	-0.782	-0.770	0.948	0.012
P36915	GNL1	0.909	0.964	1.017	0.923	0.936	0.970	0.616	0.033
O43597	SPRY2	0.009	-0.069	-0.057	-0.116	-0.030	-0.086	0.376	-0.056
Q96MW7	TIGD1	-1.873	-1.810	-1.991	-1.927	-1.842	-1.959	0.120	-0.118
Q5HYA8	TMEM67	-3.753	-3.749	-3.682	-3.329	-3.751	-3.505	0.396	0.246
P14635	CCNB1	-2.654	-2.690	-2.790	-2.816	-2.672	-2.803	0.035	-0.131
O00635	TRIM38	-0.941	-1.097	-0.752	-0.886	-1.019	-0.819	0.194	0.200
Q14004	CDK13	-2.069	-2.078	-1.957	-2.017	-2.073	-1.987	0.204	0.086
Q9HA92	RSAD1	-3.372	-3.390	-3.364	-3.268	-3.381	-3.316	0.400	0.065
Q6PCD5	RFWD3	-2.182	-2.208	-2.155	-2.086	-2.195	-2.121	0.251	0.074
Q9H8K7	PAAT	-1.930	-1.846	-1.970	-1.838	-1.888	-1.904	0.857	-0.016
P02749	APOH	-1.202	-1.173	-0.584	-0.652	-1.187	-0.618	0.017	0.569
Q14166	TTLL12	4.338	4.339	4.332	4.276	4.338	4.304	0.433	-0.035
Q9BSH5	HDHD3	-0.475	-0.432	-0.302	-0.175	-0.454	-0.239	0.155	0.215
O15037	KHNYN	0.140	0.022	0.173	0.143	0.081	0.158	0.406	0.077
O75368	SH3BGRL	-0.623	-0.325	0.046	-0.487	-0.474	-0.220	0.514	0.253
Q9UBF2	COPG2	1.317	1.166	1.176	1.115	1.242	1.146	0.409	-0.096
O95164	UBL3	-2.392	-1.972	-2.184	-1.934	-2.182	-2.059	0.673	0.123
O94868	FCHSD2	-2.603	-2.513	-2.593	-2.449	-2.558	-2.521	0.713	0.037
Q14554	PDIA5	1.458	1.385	1.730	1.670	1.421	1.700	0.030	0.278
Q02790	FKBP4	6.268	6.206	6.129	6.117	6.237	6.123	0.158	-0.114
Q86VI3	IQGAP3	2.646	2.737	2.662	2.629	2.691	2.646	0.489	-0.046
P62253	UBE2G1	0.897	0.823	0.734	0.644	0.860	0.689	0.102	-0.171
Q12948	FOXC1	-2.390	-2.450	-2.462	-2.258	-2.420	-2.360	0.662	0.060
Q15233	NONO	4.146	3.587	4.055	3.629	3.866	3.842	0.952	-0.024
Q32P28	P3H1	-3.296	-3.503	-3.488	-3.464	-3.400	-3.476	0.596	-0.076
Q96Q15	SMG1	-2.058	-2.082	-2.012	-2.009	-2.070	-2.010	0.118	0.060
P49427	CDC34	-0.307	-0.406	-0.268	-0.314	-0.356	-0.291	0.395	0.065
P60842	EIF4A1	4.327	4.384	4.534	4.420	4.355	4.477	0.241	0.122
Q7Z624	CAMKMT	-1.088	-1.236	-1.274	-1.182	-1.162	-1.228	0.540	-0.066
Q6P1L8	MRPL14	1.910	1.875	1.845	1.817	1.893	1.831	0.114	-0.062
Q9NTZ6	RBM12	4.029	3.990	4.021	4.025	4.009	4.023	0.609	0.014
P26639	TARS1	-0.097	0.024	-0.062	-0.049	-0.036	-0.056	0.800	-0.020
O14548	COX7A2L	2.260	2.226	2.178	2.065	2.243	2.121	0.259	-0.121

Q8IXM3	MRPL41	2.479	2.423	2.418	2.389	2.451	2.404	0.313	-0.047
Q56P03	EAPP	-0.175	-0.187	-0.150	-0.164	-0.181	-0.157	0.120	0.024
Q9BXB4	OSBPL11	1.647	1.599	1.505	1.534	1.623	1.520	0.090	-0.104
Q8IWT0	ZBTB8OS	-0.284	-0.226	-0.188	-0.322	-0.255	-0.255	0.997	0.000
Q96JF6	ZNF594	-3.218	-3.204	-3.331	-3.254	-3.211	-3.293	0.276	-0.081
P62249	RPS16	5.707	5.715	5.672	5.637	5.711	5.654	0.183	-0.057
P04406	GAPDH	6.856	6.705	6.663	6.643	6.781	6.653	0.337	-0.128
Q8N6N7	ACBD7	1.092	1.281	0.783	0.822	1.187	0.803	0.142	-0.384
Q96GK7	FAHD2A	0.709	0.667	0.785	0.890	0.688	0.838	0.179	0.150
Q5SY16	NOL9	2.513	2.455	2.529	2.447	2.484	2.488	0.951	0.004
Q6ZTI6	RFLNA	-5.575	-5.520	-5.378	-5.275	-5.547	-5.327	0.095	0.220
P01137	TGFB1	-2.068	-1.822	-1.815	-1.735	-1.945	-1.775	0.385	0.170
Q6PJP8	DCLRE1A	-2.680	-2.486	-2.786	-2.571	-2.583	-2.678	0.581	-0.095
Q9P0U3	SENP1	-3.958	-3.910	-3.881	-3.771	-3.934	-3.826	0.269	0.108
P38935	IGHMBP2	0.647	0.646	0.532	0.515	0.646	0.523	0.043	-0.123
Q8TCD1	C18orf32	-1.068	-1.059	-1.067	-0.999	-1.064	-1.033	0.532	0.031
P0DPB5	POLR1D	-2.340	-2.436	-2.457	-2.449	-2.388	-2.453	0.405	-0.065
O75376	NCOR1	-1.544	-1.586	-1.600	-1.637	-1.565	-1.618	0.197	-0.054
Q12768	WASHC5	2.842	2.821	2.820	2.837	2.832	2.829	0.826	-0.003
Q96I24	FUBP3	2.982	2.953	2.976	2.966	2.968	2.971	0.842	0.004
Q13823	GNL2	3.039	3.121	3.132	3.156	3.080	3.144	0.352	0.064
Q16798	ME3	-0.582	-0.027	-0.353	-0.136	-0.305	-0.245	0.867	0.060
P00918	CA2	-2.016	-1.532	-1.781	-1.731	-1.774	-1.756	0.953	0.018
Q8TAA5	GRPEL2	0.647	0.642	0.635	0.639	0.645	0.637	0.128	-0.008
Q13557	CAMK2D	-5.179	-5.256	-5.239	-5.186	-5.217	-5.213	0.929	0.005
P17036	ZNF3	-3.706	-4.011	-4.024	-3.846	-3.858	-3.935	0.715	-0.077
Q8NC56	LEMD2	0.681	0.663	0.727	0.746	0.672	0.736	0.041	0.064
Q8N0X7	SPART	-1.726	-1.530	-1.641	-1.755	-1.628	-1.698	0.614	-0.070
D6REC4	CFAP99	-2.839	-2.820	-3.071	-3.036	-2.829	-3.053	0.020	-0.224
Q8N6N3	C1orf52	-1.127	-1.142	-0.981	-1.087	-1.135	-1.034	0.305	0.101
Q7L576	CYFIP1	1.654	1.710	1.674	1.759	1.682	1.716	0.577	0.035
Q00534	CDK6	1.660	1.666	1.377	1.366	1.663	1.372	0.002	-0.291
P18564	ITGB6	-3.770	-2.735	-3.242	-3.389	-3.253	-3.316	0.923	-0.063
P08842	STS	-2.279	-2.377	-2.187	-2.159	-2.328	-2.173	0.173	0.155
O14867	BACH1	0.468	0.389	0.495	0.457	0.429	0.476	0.424	0.048

P62753	RPS6	6.759	6.709	6.693	6.743	6.734	6.718	0.695	-0.016
Q86U90	YRDC	0.915	0.860	1.111	1.060	0.888	1.085	0.035	0.197
P51580	TPMT	2.195	2.032	2.049	2.112	2.114	2.081	0.761	-0.033
Q96QE5	TEFM	0.028	-0.038	0.023	-0.127	-0.005	-0.052	0.644	-0.047
O00506	STK25	-1.862	-1.857	-0.377	-0.423	-1.859	-0.400	0.009	1.460
Q13123	IK	3.462	3.434	3.512	3.450	3.448	3.481	0.464	0.034
Q9Y3C1	NOP16	0.866	0.783	0.946	0.844	0.825	0.895	0.401	0.070
P53992	SEC24C	2.885	2.842	2.956	2.848	2.864	2.902	0.609	0.038
Q9Y3D6	FIS1	1.014	0.996	1.071	1.050	1.005	1.060	0.058	0.056
Q9UEE5	STK17A	-3.843	-3.863	-3.981	-4.036	-3.853	-4.009	0.081	-0.155
P20337	RAB3B	-2.106	-1.762	-1.837	-2.062	-1.934	-1.950	0.948	-0.015
Q5T7W7	TSTD2	-4.746	-4.654	-4.783	-4.480	-4.700	-4.632	0.732	0.068
Q9UBQ7	GRHPR	3.805	3.742	3.890	3.850	3.774	3.870	0.141	0.097
Q14642	INPP5A	-0.583	-0.501	-0.569	-0.540	-0.542	-0.554	0.820	-0.012
P43304	GPD2	0.342	0.110	0.294	0.207	0.226	0.250	0.868	0.025
Q15814	TBCC	1.928	2.087	2.000	1.988	2.007	1.994	0.894	-0.013
Q9NZW5	PALS2	2.892	2.904	2.776	2.789	2.898	2.783	0.006	-0.115
Q96ME1	FBXL18	-3.247	-3.426	-3.281	-3.311	-3.337	-3.296	0.730	0.040
O60508	CDC40	2.025	2.015	1.942	2.035	2.020	1.989	0.625	-0.031
Q66GS9	CEP135	-2.394	-2.350	-2.422	-2.283	-2.372	-2.353	0.832	0.019
Q92686	NRGN	-3.416	-3.498	-3.702	-3.711	-3.457	-3.707	0.100	-0.250
Q09472	EP300	1.249	1.257	1.145	1.160	1.253	1.153	0.018	-0.100
Q9BRX2	PELO	1.421	1.406	1.460	1.454	1.414	1.457	0.078	0.044
Q9UMX5	NENF	2.038	2.009	2.006	1.981	2.024	1.994	0.260	-0.030
P08754	GNAI3	0.773	0.651	0.718	0.646	0.712	0.682	0.719	-0.030
Q8WWI1	LMO7	-2.522	-2.458	-2.574	-2.421	-2.490	-2.497	0.938	-0.008
O60684	KPNA6	2.043	1.893	1.904	1.886	1.968	1.895	0.507	-0.073
Q96E22	NUS1	-3.517	-3.571	-3.614	-3.513	-3.544	-3.564	0.772	-0.020
Q6EMK4	VASN	-5.993	-5.972	-6.026	-5.945	-5.982	-5.985	0.958	-0.003
P32320	CDA	-1.928	-1.573	-1.607	-1.700	-1.750	-1.654	0.683	0.097
O95832	CLDN1	-3.750	-3.916	-3.074	-3.394	-3.833	-3.234	0.116	0.599
O00411	POLRMT	1.517	1.525	1.487	1.469	1.521	1.478	0.090	-0.043
Q7Z434	MAVS	-2.979	-2.793	-2.954	-2.831	-2.886	-2.893	0.959	-0.007
Q9BRR3	PGAP4	-1.806	-1.695	-1.905	-1.796	-1.751	-1.851	0.328	-0.100
Q92551	IP6K1	-1.990	-2.197	-2.098	-2.303	-2.094	-2.201	0.540	-0.107

Q8N4P3	HDDC3	-2.784	-2.826	-2.804	-2.674	-2.805	-2.739	0.487	0.066
Q9HB90	RRAGC	-0.557	-0.697	-0.752	-0.771	-0.627	-0.761	0.302	-0.134
Q9NZ32	ACTR10	1.380	1.413	1.440	1.483	1.397	1.462	0.148	0.065
Q9P227	ARHGAP23	-4.008	-3.936	-4.098	-3.944	-3.972	-4.021	0.642	-0.049
Q5TH69	ARFGEF3	-2.366	-2.259	-2.217	-2.319	-2.313	-2.268	0.607	0.044
Q6UXN9	WDR82	3.371	3.301	3.302	3.268	3.336	3.285	0.362	-0.051
Q13636	RAB31	-1.186	-1.246	-1.350	-1.368	-1.216	-1.359	0.108	-0.143
Q9UGI8	TES	-5.569	-4.079	-3.978	-5.019	-4.824	-4.499	0.758	0.325
Q96DP5	MTFMT	-0.852	-0.851	-0.904	-0.875	-0.852	-0.889	0.232	-0.038
Q96D70	R3HDM4	-1.120	-1.148	-0.881	-0.848	-1.134	-0.864	0.007	0.270
P36507	MAP2K2	3.436	3.330	3.463	3.440	3.383	3.451	0.415	0.069
Q15361	TTF1	-0.105	-0.200	-0.154	-0.123	-0.153	-0.138	0.816	0.014
Q9Y5J9	TIMM8B	0.032	-0.041	-0.060	-0.087	-0.004	-0.073	0.284	-0.069
Q9BQC6	MRPL57	3.053	2.925	3.000	2.984	2.989	2.992	0.970	0.003
O43808	SLC25A17	0.719	0.677	0.602	0.637	0.698	0.620	0.106	-0.079
Q9BYK8	HELZ2	0.840	0.790	1.144	1.086	0.815	1.115	0.017	0.300
O43924	PDE6D	0.423	0.338	0.245	0.241	0.381	0.243	0.189	-0.138
Q14152	EIF3A	3.181	3.144	3.167	3.132	3.162	3.150	0.662	-0.013
Q9NZV6	MSRB1	-3.595	-3.782	-3.754	-3.802	-3.688	-3.778	0.508	-0.090
P09913	IFIT2	0.279	0.204	0.738	0.592	0.241	0.665	0.063	0.424
P41208	CETN2	0.560	0.517	0.505	0.397	0.538	0.451	0.329	-0.088
Q8WWN8	ARAP3	-3.208	-3.267	-3.627	-3.632	-3.237	-3.629	0.047	-0.392
O14530	TXNDC9	-0.391	-0.330	-0.452	-0.305	-0.361	-0.378	0.852	-0.018
P29373	CRABP2	-0.436	0.260	-0.267	-0.318	-0.088	-0.292	0.662	-0.204
Q9H6K4	OPA3	-2.095	-2.100	-2.042	-2.096	-2.097	-2.069	0.488	0.028
Q9UID6	ZNF639	-2.362	-2.254	-2.298	-2.227	-2.308	-2.262	0.560	0.046
Q99832	CCT7	-0.041	-0.514	-0.318	-0.304	-0.278	-0.311	0.910	-0.033
P46063	RECQL	5.867	5.815	5.941	5.910	5.841	5.926	0.136	0.085
Q8IWR0	ZC3H7A	1.127	1.112	1.063	1.100	1.120	1.081	0.254	-0.038
Q8IXL6	FAM20C	2.267	2.064	-0.544	0.015	2.165	-0.265	0.048	-2.430
P57060	RWDD2B	-1.355	-1.396	-1.467	-1.321	-1.375	-1.394	0.842	-0.019
Q9BZQ8	NIBAN1	1.915	1.795	2.457	2.399	1.855	2.428	0.032	0.574
Q8NCA9	ZNF784	-4.251	-4.421	-4.184	-4.301	-4.336	-4.243	0.472	0.093
O95271	TNKS	-5.554	-5.541	-5.570	-5.625	-5.548	-5.597	0.306	-0.050
Q9NX46	ADPRS	2.833	2.838	2.887	2.837	2.836	2.862	0.477	0.027

P02462	COL4A1	-3.242	-3.345	-3.037	-2.915	-3.293	-2.976	0.061	0.317
P52746	ZNF142	-3.601	-3.549	-3.552	-3.527	-3.575	-3.540	0.383	0.035
P19388	POLR2E	2.069	2.081	2.042	2.052	2.075	2.047	0.079	-0.028
P18583	SON	-1.924	-1.909	-1.909	-1.913	-1.917	-1.911	0.606	0.005
P31949	S100A11	5.859	5.869	5.994	5.914	5.864	5.954	0.259	0.090
Q9BR76	CORO1B	3.885	3.770	3.816	3.891	3.827	3.854	0.743	0.026
Q587I9	SFT2D3	-0.620	-0.550	-0.887	-0.776	-0.585	-0.831	0.083	-0.246
Q10570	CPSF1	4.005	4.021	4.013	3.990	4.013	4.001	0.498	-0.012
Q9NZJ0	DTL	-1.780	-1.623	-1.940	-1.839	-1.701	-1.890	0.201	-0.188
Q9Y6X3	MAU2	-1.347	-1.310	-1.367	-1.285	-1.329	-1.326	0.956	0.003
Q9NQP4	PFDN4	2.641	2.574	2.311	2.336	2.608	2.323	0.048	-0.284
P29034	S100A2	0.615	0.845	0.949	0.403	0.730	0.676	0.879	-0.054
Q9UFN0	NIPSNAP3A	1.271	1.136	1.479	1.329	1.204	1.404	0.186	0.200
Q7Z7B1	PIGW	-1.064	-1.098	-1.147	-1.074	-1.081	-1.111	0.567	-0.030
P43121	MCAM	-4.627	-4.881	-4.036	-4.372	-4.754	-4.204	0.130	0.550
Q9BPU6	DPYSL5	-0.511	-0.524	-0.230	-0.099	-0.518	-0.164	0.115	0.353
P51398	DAP3	-3.183	-3.175	-3.247	-3.223	-3.179	-3.235	0.100	-0.056
Q8TCS8	PNPT1	4.274	4.277	4.356	4.299	4.276	4.327	0.320	0.052
Q5T440	IBA57	0.099	0.163	0.122	0.225	0.131	0.174	0.564	0.043
Q9BRF8	CPPED1	-1.453	-1.378	-1.508	-1.301	-1.416	-1.405	0.933	0.011
Q8NI27	THOC2	3.482	3.471	3.455	3.508	3.477	3.481	0.886	0.005
O00571	DDX3X	2.046	1.695	2.223	1.895	1.870	2.059	0.515	0.189
P46060	RANGAP1	4.501	4.528	4.408	4.369	4.515	4.388	0.045	-0.126
P09497	CLTB	0.204	0.167	0.437	0.405	0.186	0.421	0.011	0.235
Q86W92	PPFIBP1	-2.868	-2.796	-2.759	-2.800	-2.832	-2.779	0.356	0.053
Q8NAP3	ZBTB38	-0.763	-0.761	-0.727	-0.670	-0.762	-0.698	0.265	0.064
P68371	TUBB4B	1.926	1.896	1.921	1.754	1.911	1.838	0.542	-0.073
Q9Y4G8	RAPGEF2	-4.894	-4.840	-4.670	-4.706	-4.867	-4.688	0.042	0.179
Q14764	MVP	4.704	4.628	4.839	4.799	4.666	4.819	0.103	0.153
P61604	HSPE1	7.198	7.216	7.360	7.304	7.207	7.332	0.112	0.125
Q9H3P7	ACBD3	2.090	2.102	2.102	2.073	2.096	2.088	0.662	-0.009
Q9P2W9	STX18	1.379	1.370	1.315	1.351	1.375	1.333	0.239	-0.042
Q9BUA3	SPINDOC	-0.993	-0.962	-1.105	-0.962	-0.978	-1.034	0.574	-0.056
P08729	KRT7	-0.161	1.177	1.134	-0.012	0.508	0.561	0.958	0.053
Q53GA4	PHLDA2	0.471	0.538	0.470	0.523	0.504	0.497	0.876	-0.008

Q96DT6	ATG4C	-0.576	-0.559	-0.663	-0.534	-0.568	-0.599	0.715	-0.031
Q93075	TATDN2	-4.010	-4.152	-3.963	-4.035	-4.081	-3.999	0.441	0.082
Q8WV74	NUDT8	-1.661	-1.807	-1.718	-1.818	-1.734	-1.768	0.740	-0.034
Q9UNX3	RPL26L1	-1.240	-1.332	-1.146	-1.231	-1.286	-1.189	0.260	0.098
P57740	NUP107	0.565	0.418	0.507	0.539	0.492	0.523	0.744	0.031
Q13451	FKBP5	3.273	3.113	3.300	3.237	3.193	3.268	0.513	0.075
Q8NBS9	TXNDC5	2.964	2.841	2.982	2.940	2.903	2.961	0.511	0.058
O75477	ERLIN1	3.018	2.934	2.992	2.933	2.976	2.963	0.815	-0.014
O75436	VPS26A	3.013	2.953	2.912	2.967	2.983	2.940	0.405	-0.043
Q9H944	MED20	-0.401	-0.167	-0.521	-0.404	-0.284	-0.463	0.344	-0.179
P07738	BPGM	-0.391	-0.471	-0.400	-0.413	-0.431	-0.406	0.653	0.024
P12956	XRCC6	3.484	3.711	3.504	3.710	3.598	3.607	0.958	0.009
Q5VTL8	PRPF38B	0.284	0.179	0.313	0.360	0.232	0.337	0.264	0.105
O76024	WFS1	1.508	1.753	1.575	1.649	1.630	1.612	0.907	-0.018
P52815	MRPL12	3.128	3.123	3.008	2.990	3.125	2.999	0.034	-0.126
O76041	NEBL	-1.448	-1.567	-1.547	-1.476	-1.507	-1.512	0.959	-0.004
Q86YZ3	HRNR	-1.949	-1.932	-2.355	-1.889	-1.941	-2.122	0.579	-0.181
Q9H0R6	QRSL1	-0.283	-0.291	-0.380	-0.375	-0.287	-0.377	0.005	-0.091
Q9GZL7	WDR12	4.345	4.300	4.333	4.284	4.322	4.309	0.727	-0.013
Q13951	CBFB	1.451	1.358	1.305	1.372	1.404	1.339	0.378	-0.066
Q9Y3I0	RTCB	5.298	5.250	5.328	5.270	5.274	5.299	0.573	0.025
P08069	IGF1R	1.331	1.404	1.336	1.321	1.368	1.328	0.472	-0.039
Q96FW1	OTUB1	3.333	3.344	3.170	3.246	3.339	3.208	0.175	-0.131
Q9H2G2	SLK	-3.375	-2.655	-3.004	-3.006	-3.015	-3.005	0.983	0.010
Q9H3R5	CENPH	-0.275	-0.219	-0.265	-0.260	-0.247	-0.262	0.688	-0.015
Q9HAZ1	CLK4	-2.552	-2.516	-2.529	-2.402	-2.534	-2.466	0.470	0.068
Q8WWC4	MAIP1	2.021	1.965	2.060	2.088	1.993	2.074	0.164	0.081
Q9H479	FN3K	2.197	1.994	2.187	2.135	2.095	2.161	0.634	0.066
Q8TB37	NUBPL	-2.914	-2.688	-2.952	-2.964	-2.801	-2.958	0.396	-0.157
Q8WVM8	SCFD1	1.683	1.614	1.557	1.548	1.648	1.553	0.216	-0.096
P22670	RFX1	-0.577	-0.587	-0.561	-0.495	-0.582	-0.528	0.340	0.055
P14866	HNRNPL	0.835	0.681	0.826	0.699	0.758	0.763	0.966	0.005
A6NCS6	C2orf72	-3.816	-3.873	-3.841	-3.828	-3.845	-3.835	0.781	0.010
Q9Y294	ASF1A	-2.535	-2.428	-2.579	-2.402	-2.482	-2.491	0.942	-0.009
P19532	TFE3	-1.895	-2.109	-1.836	-1.956	-2.002	-1.896	0.499	0.106

O76003	GLRX3	5.595	5.603	5.498	5.505	5.599	5.502	0.003	-0.097
P05455	SSB	6.217	6.161	6.209	6.211	6.189	6.210	0.592	0.021
P48506	GCLC	1.741	2.182	2.027	2.110	1.961	2.069	0.712	0.107
Q6ICB0	DESI1	0.147	0.139	-0.041	0.085	0.143	0.022	0.305	-0.121
Q9BST9	RTKN	-2.460	-2.329	-2.291	-2.223	-2.395	-2.257	0.241	0.138
Q9NWT6	HIF1AN	0.906	0.852	0.965	0.894	0.879	0.929	0.383	0.050
Q96A65	EXOC4	1.328	1.355	1.262	1.328	1.342	1.295	0.373	-0.047
Q16533	SNAPC1	-1.148	-1.175	-1.329	-1.268	-1.161	-1.299	0.099	-0.137
P11117	ACP2	-0.579	-0.619	-0.375	-0.474	-0.599	-0.424	0.139	0.175
Q9UKK6	NXT1	-1.247	-1.234	-1.443	-1.334	-1.241	-1.388	0.219	-0.148
P10412	H1	3.588	3.805	3.622	3.594	3.696	3.608	0.564	-0.088
O94927	HAUS5	-0.142	-0.169	-0.163	-0.087	-0.155	-0.125	0.569	0.030
Q96IG2	FBXL20	-2.773	-3.068	-2.683	-2.902	-2.920	-2.792	0.564	0.128
Q15759	MAPK11	-5.004	-4.930	-5.009	-4.833	-4.967	-4.921	0.699	0.046
Q9Y285	FARSA	1.126	1.080	0.990	0.973	1.103	0.981	0.087	-0.122
Q99590	SCAF11	-6.377	-6.335	-6.273	-6.198	-6.356	-6.235	0.139	0.120
Q9Y5A9	YTHDF2	-1.436	-1.617	-1.531	-1.571	-1.527	-1.551	0.834	-0.024
P23786	CPT2	4.470	4.514	4.537	4.543	4.492	4.540	0.266	0.048
Q9Y5T4	DNAJC15	-2.728	-2.574	-2.834	-2.562	-2.651	-2.698	0.799	-0.047
O43513	MED7	-0.656	-0.581	-0.573	-0.492	-0.619	-0.533	0.262	0.086
A1X283	SH3PXD2B	1.642	1.575	1.534	1.527	1.609	1.531	0.253	-0.078
Q6PID6	TTC33	-1.186	-1.359	-1.098	-1.216	-1.272	-1.157	0.396	0.116
P53597	SUCLG1	2.693	2.678	2.738	2.750	2.686	2.744	0.031	0.059
Q8WY22	BRI3BP	-0.618	-0.746	-0.483	-0.694	-0.682	-0.588	0.541	0.094
Q68CZ6	HAUS3	-0.699	-0.845	-0.760	-0.865	-0.772	-0.812	0.702	-0.040
O43257	ZNHIT1	-2.402	-2.342	-2.318	-2.218	-2.372	-2.268	0.244	0.104
Q7L1W4	LRRC8D	-0.921	-1.046	-1.251	-1.177	-0.983	-1.214	0.113	-0.230
Q96I76	GPATCH3	-4.860	-4.845	-5.135	-4.951	-4.853	-5.043	0.286	-0.190
Q9ULE0	WWC3	-3.480	-3.612	-3.456	-3.234	-3.546	-3.345	0.286	0.201
Q52LJ0	FAM98B	-3.013	-3.544	-2.993	-3.465	-3.279	-3.229	0.901	0.050
Q04721	NOTCH2	-0.100	-0.186	0.033	-0.002	-0.143	0.015	0.131	0.158
Q9HDC9	APMAP	2.987	2.879	2.887	2.770	2.933	2.828	0.319	-0.104
Q96EP0	RNF31	-3.338	-3.274	-3.322	-3.061	-3.306	-3.192	0.539	0.114
P08123	COL1A2	-1.270	-1.422	-1.452	-1.368	-1.346	-1.410	0.556	-0.064
O15372	EIF3H	4.455	4.417	4.353	4.376	4.436	4.364	0.111	-0.071

P12755	SKI	-0.876	-0.874	-0.944	-0.908	-0.875	-0.926	0.217	-0.051
O60303	KATNIP	-3.190	-3.099	-3.114	-3.034	-3.145	-3.074	0.364	0.071
Q96RK0	CIC	0.015	0.078	0.024	0.136	0.046	0.080	0.666	0.034
Q15257	PTPA	-2.185	-2.064	-2.176	-2.235	-2.124	-2.206	0.389	-0.081
P23258	TUBG1	0.965	1.024	1.066	0.958	0.995	1.012	0.806	0.018
Q76I76	SSH2	-2.017	-1.987	-2.110	-1.948	-2.002	-2.029	0.801	-0.026
Q9GZZ9	UBA5	0.042	0.067	0.230	0.147	0.054	0.188	0.168	0.134
Q14192	FHL2	3.371	3.409	3.240	3.145	3.390	3.193	0.113	-0.197
Q8IX07	ZFPM1	-2.516	-2.450	-2.531	-2.472	-2.483	-2.501	0.719	-0.018
P78368	CSNK1G2	-2.808	-2.715	-2.622	-2.582	-2.762	-2.602	0.139	0.160
P00491	PNP	4.643	4.673	4.478	4.525	4.658	4.502	0.043	-0.156
Q969G6	RFK	-2.987	-3.101	-2.822	-2.974	-3.044	-2.898	0.273	0.146
Q8TDR2	STK35	-3.371	-3.312	-3.091	-3.282	-3.341	-3.187	0.335	0.155
P78345	RPP38	2.191	2.180	2.185	2.188	2.186	2.187	0.929	0.001
O95994	AGR2	-0.501	-0.095	-0.018	-0.628	-0.298	-0.323	0.952	-0.025
P78406	RAE1	4.071	4.029	4.108	4.028	4.050	4.068	0.747	0.017
Q15907	RAB11B	0.216	0.191	0.161	-0.024	0.204	0.069	0.380	-0.135
Q96IK1	BOD1	-2.108	-2.186	-2.263	-2.167	-2.147	-2.215	0.390	-0.068
Q9H6Y2	WDR55	0.762	0.672	0.817	0.768	0.717	0.792	0.310	0.076
P15514	AREG	-0.220	-0.261	0.004	0.060	-0.241	0.032	0.020	0.273
Q9Y4D7	PLXND1	-2.986	-3.176	-3.079	-2.942	-3.081	-3.010	0.614	0.071
P26374	CHML	-0.961	-1.024	-1.185	-1.145	-0.993	-1.165	0.058	-0.172
Q8N0Z8	PUSL1	-1.879	-1.789	-1.871	-1.671	-1.834	-1.771	0.644	0.063
Q08999	RBL2	-0.826	-0.851	-0.718	-0.712	-0.838	-0.715	0.050	0.123
Q9HBM1	SPC25	1.944	1.908	1.541	1.560	1.926	1.550	0.010	-0.376
A6NHR9	SMCHD1	0.589	0.427	0.596	0.533	0.508	0.564	0.611	0.056
Q9Y4H2	IRS2	0.472	0.563	0.601	0.605	0.517	0.603	0.310	0.086
Q99766	DMAC2L	0.686	0.725	0.768	0.687	0.705	0.727	0.690	0.022
Q4KMP7	TBC1D10B	1.365	1.368	1.300	1.262	1.367	1.281	0.136	-0.086
Q9UJM3	ERRFI1	2.701	2.514	2.817	2.791	2.608	2.804	0.278	0.196
P62942	FKBP1A	1.318	1.356	1.309	1.326	1.337	1.317	0.473	-0.020
P50440	GATM	-6.695	-6.033	-6.038	-6.419	-6.364	-6.229	0.764	0.136
Q8WYQ3	CHCHD10	-1.317	-1.394	-1.611	-1.521	-1.355	-1.566	0.074	-0.210
O94819	KBTBD11	-2.561	-2.406	-2.663	-2.426	-2.483	-2.545	0.713	-0.061
O96006	ZBED1	-2.154	-2.300	-2.268	-2.098	-2.227	-2.183	0.731	0.044

P49411	TUFM	5.935	5.838	6.020	5.958	5.887	5.989	0.239	0.103
P00492	HPRT1	5.138	5.088	5.035	5.000	5.113	5.018	0.100	-0.095
Q9BTK6	PAGR1	-1.848	-1.903	-2.015	-1.945	-1.875	-1.980	0.149	-0.105
Q9H3G5	CPVL	-0.737	-0.475	-0.517	-0.663	-0.606	-0.590	0.930	0.015
Q9H2K0	MTIF3	-1.339	-1.461	-1.401	-1.446	-1.400	-1.424	0.766	-0.024
Q96EY4	TMA16	2.540	2.471	2.409	2.359	2.505	2.384	0.118	-0.121
Q9Y3S2	ZNF330	0.441	0.363	0.429	0.409	0.402	0.419	0.743	0.017
Q96BN8	OTULIN	0.461	0.384	0.555	0.497	0.422	0.526	0.174	0.104
Q7L273	KCTD9	-0.093	-0.126	-0.260	-0.269	-0.110	-0.265	0.054	-0.155
Q9H7S9	ZNF703	0.634	0.491	0.725	0.575	0.562	0.650	0.487	0.088
O94832	MYO1D	1.516	2.017	1.718	1.761	1.766	1.739	0.932	-0.027
O95562	SFT2D2	-0.839	-0.945	-0.897	-0.877	-0.892	-0.887	0.935	0.005
Q14586	ZNF267	-6.139	-6.224	-6.188	-6.373	-6.182	-6.280	0.469	-0.099
P26640	VARS1	4.574	4.583	4.492	4.537	4.578	4.514	0.208	-0.064
Q9Y4X5	ARIH1	1.307	1.279	1.380	1.280	1.293	1.330	0.594	0.037
P13473	LAMP2	-2.478	-2.476	-2.149	-2.248	-2.477	-2.198	0.111	0.279
P46934	NEDD4	-0.241	-0.257	-0.368	-0.286	-0.249	-0.327	0.300	-0.078
P49674	CSNK1E	-2.694	-2.743	-2.323	-2.240	-2.719	-2.282	0.021	0.437
Q9H008	LHPP	-5.278	-5.347	-5.291	-5.184	-5.313	-5.238	0.375	0.075
Q9NXR7	BABAM2	-1.464	-1.347	-1.313	-1.218	-1.405	-1.265	0.210	0.140
Q9H4M3	FBXO44	-3.733	-3.741	-4.085	-3.992	-3.737	-4.039	0.095	-0.302
O75832	PSMD10	3.180	3.146	3.109	3.172	3.163	3.141	0.611	-0.022
O75165	DNAJC13	4.726	4.681	4.609	4.629	4.704	4.619	0.127	-0.084
P13647	KRT5	-3.482	-3.501	-4.083	-3.692	-3.492	-3.888	0.291	-0.396
Q8N653	LZTR1	-1.855	-1.981	-1.928	-2.021	-1.918	-1.974	0.552	-0.056
Q8N3C0	ASCC3	2.608	2.599	2.535	2.531	2.604	2.533	0.019	-0.071
P11137	MAP2	-0.950	-0.984	-1.385	-1.447	-0.967	-1.416	0.015	-0.449
Q8NE71	ABCF1	-0.873	-0.785	-0.970	-0.843	-0.829	-0.907	0.432	-0.077
Q6PD74	AAGAB	-0.303	-0.345	-0.204	-0.413	-0.324	-0.308	0.905	0.016
Q9Y6G5	COMMD10	-0.819	-0.852	-0.843	-0.809	-0.836	-0.826	0.726	0.010
Q13488	TCIRG1	-0.768	-0.801	-0.917	-0.887	-0.784	-0.902	0.035	-0.118
Q3SY56	SP6	-3.525	-3.328	-3.592	-3.501	-3.426	-3.546	0.422	-0.120
Q96QE3	ATAD5	-2.124	-2.233	-2.374	-2.534	-2.178	-2.454	0.120	-0.276
P78536	ADAM17	-2.351	-2.039	-2.265	-2.107	-2.195	-2.186	0.965	0.009
P20591	MX1	-2.579	-2.427	-2.448	-1.843	-2.503	-2.145	0.439	0.358

Q9Y2G3	ATP11B	0.084	0.129	0.042	0.160	0.106	0.101	0.948	-0.005
P14735	IDE	3.248	3.197	3.163	3.096	3.222	3.129	0.170	-0.093
Q2VPB7	AP5B1	-2.932	-2.880	-2.962	-2.827	-2.906	-2.895	0.897	0.011
Q9UBU7	DBF4	-4.336	-4.242	-4.429	-4.564	-4.289	-4.496	0.143	-0.207
Q9NZ45	CISD1	0.790	0.736	0.799	0.848	0.763	0.823	0.237	0.060
Q9NX47	MARCHF5	-1.055	-1.174	-1.264	-1.222	-1.115	-1.243	0.252	-0.128
Q86YV9	HPS6	0.140	0.153	0.014	0.148	0.147	0.081	0.507	-0.066
P82675	MRPS5	1.709	1.718	1.745	1.672	1.713	1.709	0.915	-0.005
P62937	PPIA	6.592	6.458	6.676	6.508	6.525	6.592	0.602	0.067
P08865	RPSA	5.672	5.691	5.650	5.658	5.682	5.654	0.173	-0.028
Q8NG68	TTL	0.450	0.355	0.117	0.268	0.402	0.193	0.167	-0.210
P09958	FURIN	-6.038	-5.629	-5.681	-5.876	-5.834	-5.779	0.838	0.055
Q6IAN0	DHRS7B	1.418	1.313	1.362	1.258	1.366	1.310	0.533	-0.056
Q56NI9	ESCO2	-2.416	-2.533	-2.575	-2.559	-2.474	-2.567	0.354	-0.093
Q9H2G9	BLZF1	-3.425	-3.367	-3.444	-3.386	-3.396	-3.415	0.686	-0.019
Q8IUC4	RHPN2	-0.462	-0.320	-0.563	-0.361	-0.391	-0.462	0.630	-0.071
Q7Z7K2	ZNF467	-4.162	-4.192	-4.124	-4.164	-4.177	-4.144	0.329	0.033
O75663	TIPRL	0.028	0.017	0.181	0.239	0.023	0.210	0.088	0.188
Q9BYC9	MRPL20	1.404	1.369	1.311	1.369	1.386	1.340	0.324	-0.047
Q9BZR9	TRIM8	-2.411	-2.532	-2.446	-2.340	-2.471	-2.393	0.436	0.078
Q5TBC7	BCL2L15	-3.734	-3.555	-3.678	-3.516	-3.644	-3.597	0.734	0.047
P11166	SLC2A1	2.129	3.380	2.935	2.757	2.754	2.846	0.908	0.091
P43378	PTPN9	0.148	0.156	0.054	0.096	0.152	0.075	0.155	-0.077
P25398	RPS12	6.122	5.958	6.029	5.997	6.040	6.013	0.799	-0.027
O94880	PHF14	-1.410	-1.412	-1.399	-1.422	-1.411	-1.411	0.967	0.001
P62854	RPS26	4.039	4.007	3.852	3.836	4.023	3.844	0.025	-0.179
Q9BQ89	FAM110A	-5.159	-4.921	-5.251	-4.499	-5.040	-4.875	0.738	0.165
Q13541	EIF4EBP1	1.614	1.604	1.545	1.602	1.609	1.573	0.421	-0.036
Q9NZ01	TECR	3.063	3.010	2.897	2.928	3.036	2.913	0.078	-0.124
Q9Y547	HSPB11	-1.881	-1.771	-2.259	-2.182	-1.826	-2.220	0.036	-0.394
O00220	TNFRSF10A	-0.418	-0.464	-0.301	-0.321	-0.441	-0.311	0.074	0.129
P50479	PDLIM4	0.848	1.119	0.841	0.937	0.983	0.889	0.612	-0.094
O00186	STXBP3	3.094	2.970	3.156	3.132	3.032	3.144	0.312	0.112
P26447	S100A4	4.460	4.615	4.247	4.337	4.538	4.292	0.142	-0.246
Q6WKZ4	RAB11FIP1	-3.710	-3.771	-3.653	-3.792	-3.740	-3.722	0.842	0.018

Q7L3S4	ZNF771	-0.529	-0.732	-0.529	-0.620	-0.630	-0.575	0.684	0.056
Q6NUK1	SLC25A24	-1.847	-1.856	-1.988	-1.887	-1.852	-1.937	0.338	-0.086
Q13045	FLII	-2.550	-2.639	-2.590	-2.491	-2.595	-2.540	0.501	0.054
Q68D10	SPTY2D1	-2.864	-2.686	-2.838	-2.625	-2.775	-2.732	0.785	0.043
P04156	PRNP	0.392	0.351	0.472	0.442	0.372	0.457	0.090	0.085
Q5T280	SPOUT1	0.701	0.722	0.668	0.633	0.711	0.651	0.127	-0.061
Q9H063	MAF1	-0.850	-0.891	-0.766	-0.880	-0.870	-0.823	0.554	0.047
Q8N8R7	ARL14EP	-0.417	-0.437	-0.411	-0.369	-0.427	-0.390	0.296	0.037
P52292	KPNA2	3.330	3.430	3.284	3.259	3.380	3.271	0.259	-0.108
Q9H0E2	TOLLIP	0.097	-0.063	0.064	-0.095	0.017	-0.015	0.801	-0.032
Q9NZM1	MYOF	-3.723	-3.211	-3.756	-3.883	-3.467	-3.820	0.390	-0.353
Q04724	TLE1	-0.908	-0.894	-0.975	-0.998	-0.901	-0.986	0.035	-0.085
P24386	CHM	0.302	0.316	0.166	0.282	0.309	0.224	0.378	-0.085
Q9Y3B4	SF3B6	2.997	2.955	3.088	3.017	2.976	3.053	0.233	0.077
Q5U5X0	LYRM7	2.433	2.389	2.238	2.354	2.411	2.296	0.270	-0.115
Q562R1	ACTBL2	0.507	0.961	0.210	1.415	0.734	0.812	0.920	0.078
Q8IY37	DHX37	2.459	2.417	2.379	2.399	2.438	2.389	0.213	-0.049
P54132	BLM	-0.219	-0.062	-0.291	-0.210	-0.141	-0.251	0.374	-0.110
P40926	MDH2	6.094	5.966	6.138	6.055	6.030	6.097	0.488	0.067
P21283	ATP6V1C1	3.721	3.679	3.748	3.642	3.700	3.695	0.941	-0.005
Q9Y3C0	WASHC3	-0.021	-0.047	-0.111	-0.204	-0.034	-0.158	0.207	-0.124
A4D1E9	GTPBP10	0.647	0.603	0.519	0.516	0.625	0.518	0.126	-0.108
Q96MX6	DNAAF10	-3.089	-3.234	-3.319	-3.245	-3.161	-3.282	0.316	-0.121
Q9Y3C8	UFC1	2.958	2.879	2.771	2.832	2.918	2.802	0.152	-0.117
P54278	PMS2	-1.196	-1.422	-1.544	-1.571	-1.309	-1.558	0.268	-0.249
P20749	BCL3	-3.507	-3.633	-3.449	-3.474	-3.570	-3.462	0.329	0.108
O14545	TRAFD1	-1.145	-1.244	-1.234	-1.227	-1.195	-1.230	0.601	-0.036
Q9Y230	RUVBL2	-0.163	-0.267	-0.139	-0.204	-0.215	-0.172	0.566	0.043
Q9ULT8	HECTD1	3.080	3.044	2.965	2.975	3.062	2.970	0.099	-0.092
P25116	F2R	-0.198	-0.268	-0.674	-0.619	-0.233	-0.646	0.014	-0.413
Q86SQ0	PHLDB2	-0.210	-0.342	-0.226	-0.250	-0.276	-0.238	0.665	0.038
P47712	PLA2G4A	-3.707	-3.848	-3.753	-3.909	-3.778	-3.831	0.665	-0.053
P62701	RPS4X	5.553	5.504	5.534	5.565	5.529	5.550	0.552	0.021
Q8NB16	MLKL	0.361	0.246	0.185	0.057	0.303	0.121	0.168	-0.182
P18846	ATF1	-3.718	-3.475	-3.490	-3.455	-3.596	-3.472	0.491	0.124

Q9H1E3	NUCKS1	0.889	0.795	1.352	1.310	0.842	1.331	0.032	0.489
Q5JTH9	RRP12	0.626	0.575	0.801	0.691	0.601	0.746	0.189	0.146
Q9BRZ2	TRIM56	1.072	1.054	1.161	1.156	1.063	1.158	0.039	0.095
Q86XZ4	SPATS2	0.528	0.442	0.344	0.384	0.485	0.364	0.174	-0.121
Q9NR56	MBNL1	-0.327	-0.252	-0.326	-0.234	-0.290	-0.280	0.887	0.010
P46109	CRKL	3.414	3.383	3.491	3.416	3.399	3.454	0.359	0.055
Q08426	EHHADH	0.253	0.013	0.299	0.149	0.133	0.224	0.595	0.091
Q15477	SKIV2L	1.039	1.132	1.106	1.102	1.085	1.104	0.758	0.019
Q86XI8	ZSWIM9	-3.905	-3.839	-3.788	-3.730	-3.872	-3.759	0.127	0.113
O43741	PRKAB2	-1.747	-2.054	-1.759	-1.624	-1.901	-1.691	0.383	0.209
P11766	ADH5	4.916	4.920	4.874	4.998	4.918	4.936	0.819	0.018
Q16643	DBN1	-6.743	-6.837	-6.879	-6.873	-6.790	-6.876	0.316	-0.086
Q9Y3L3	SH3BP1	-4.597	-4.535	-4.653	-4.169	-4.566	-4.411	0.636	0.155
P0CG13	CHTF8	-3.398	-3.430	-3.614	-3.177	-3.414	-3.396	0.947	0.018
Q9Y4D8	HECTD4	-2.848	-2.923	-2.960	-2.681	-2.886	-2.821	0.723	0.065
Q9NX58	LYAR	3.948	3.879	4.070	4.024	3.913	4.047	0.100	0.133
P57772	EEFSEC	-1.092	-1.107	-1.078	-1.061	-1.100	-1.070	0.126	0.030
Q96MH6	TMEM68	-1.382	-1.331	-1.069	-1.112	-1.357	-1.090	0.016	0.266
P08582	MELTF	2.039	2.026	1.968	2.021	2.033	1.995	0.377	-0.038
Q5VWN6	TASOR2	0.125	0.090	0.163	0.165	0.108	0.164	0.192	0.056
Q53R41	FASTKD1	-1.897	-1.788	-1.852	-1.798	-1.843	-1.825	0.810	0.017
Q9NY61	AATF	2.833	2.753	2.872	2.806	2.793	2.839	0.469	0.046
Q64LD2	WDR25	-1.952	-1.987	-1.932	-1.889	-1.969	-1.910	0.172	0.059
P48681	NES	0.180	0.256	0.055	0.043	0.218	0.049	0.135	-0.169
Q9P003	CNIH4	-1.103	-1.064	-1.484	-0.945	-1.084	-1.214	0.713	-0.131
Q6DKI1	RPL7L1	1.814	1.878	1.862	1.950	1.846	1.906	0.394	0.060
Q14669	TRIP12	-2.973	-3.081	-2.870	-2.902	-3.027	-2.886	0.210	0.141
P37235	HPCAL1	1.952	1.968	1.692	1.796	1.960	1.744	0.144	-0.216
O75223	GGCT	0.948	0.806	0.883	0.828	0.877	0.856	0.817	-0.021
Q8IYD1	GSPT2	2.248	2.162	2.175	2.244	2.205	2.210	0.938	0.005
Q9H1E5	TMX4	0.768	0.774	0.324	0.370	0.771	0.347	0.032	-0.424
Q86WW8	COA5	-3.044	-2.936	-2.973	-2.896	-2.990	-2.934	0.498	0.056
P42330	AKR1C3	-4.205	-3.474	-3.156	-3.959	-3.839	-3.558	0.656	0.281
Q7Z6J0	SH3RF1	-2.864	-2.682	-2.925	-3.008	-2.773	-2.967	0.244	-0.194
Q5MNZ6	WDR45B	0.239	0.272	0.277	0.263	0.255	0.270	0.538	0.015

Q9NR30	DDX21	2.137	2.001	2.188	2.157	2.069	2.173	0.361	0.104
Q9BVJ6	UTP14A	1.658	1.557	1.728	1.618	1.607	1.673	0.472	0.066
Q9Y620	RAD54B	-2.596	-2.805	-2.858	-2.766	-2.701	-2.812	0.470	-0.111
Q9UHQ4	BCAP29	-2.735	-2.670	-2.689	-2.660	-2.703	-2.675	0.543	0.028
P57729	RAB38	-2.062	-1.740	-1.973	-1.964	-1.901	-1.968	0.749	-0.067
Q9P2J9	PDP2	-1.622	-1.669	-1.605	-1.556	-1.645	-1.580	0.196	0.065
O96014	WNT11	-4.678	-4.604	-4.745	-4.660	-4.641	-4.703	0.386	-0.062
A0A096LP01	SMIM26	-2.978	-2.916	-2.884	-2.884	-2.947	-2.884	0.290	0.063
Q13439	GOLGA4	-5.189	-5.210	-5.176	-5.129	-5.199	-5.152	0.261	0.047
Q9NRR8	CDC42SE1	-3.545	-3.545	-3.185	-3.168	-3.545	-3.176	0.014	0.369
Q96E09	PABIR1	-2.195	-2.158	-2.157	-2.254	-2.177	-2.206	0.655	-0.029
Q06190	PPP2R3A	-3.204	-3.221	-3.177	-3.152	-3.213	-3.164	0.104	0.048
Q9NRY6	PLSCR3	-1.452	-1.520	-1.270	-1.383	-1.486	-1.326	0.163	0.159
Q6DKJ4	NXN	-0.070	0.084	-0.231	-0.123	0.007	-0.177	0.205	-0.184
Q8N1G1	REXO1	-0.844	-0.902	-0.888	-0.821	-0.873	-0.854	0.720	0.018
Q8IZ07	ANKRD13A	-0.720	-0.751	-0.720	-0.765	-0.735	-0.743	0.825	-0.007
P40306	PSMB10	-0.911	-0.897	-0.404	-0.537	-0.904	-0.470	0.094	0.434
Q14690	PDCD11	5.434	5.396	5.434	5.441	5.415	5.437	0.452	0.022
Q99583	MNT	-1.526	-1.391	-1.566	-1.139	-1.459	-1.352	0.707	0.106
Q92688	ANP32B	-6.707	-6.398	-6.088	-6.114	-6.553	-6.101	0.208	0.452
P53794	SLC5A3	0.157	0.083	0.038	-0.094	0.120	-0.028	0.221	-0.148
P05198	EIF2S1	5.448	5.532	5.391	5.415	5.490	5.403	0.267	-0.088
Q8IXK2	GALNT12	-3.793	-3.579	-3.750	-3.829	-3.686	-3.789	0.506	-0.103
Q86VP6	CAND1	2.748	2.753	2.828	2.778	2.750	2.803	0.279	0.052
Q8NFH4	NUP37	2.590	2.571	2.586	2.528	2.580	2.557	0.567	-0.023
Q9HAS0	C17orf75	0.486	0.555	0.441	0.491	0.520	0.466	0.337	-0.054
Q9NUQ2	AGPAT5	0.984	0.859	0.989	0.926	0.922	0.957	0.675	0.036
Q2QGD7	ZXDC	-6.583	-6.774	-6.295	-6.628	-6.678	-6.461	0.399	0.217
O94804	STK10	2.380	2.364	2.300	2.291	2.372	2.295	0.026	-0.076
Q16649	NFIL3	-1.088	-1.237	-1.225	-1.290	-1.163	-1.258	0.408	-0.095
P02795	MT2A	0.041	-0.137	0.249	0.166	-0.048	0.208	0.171	0.256
Q15573	TAF1A	-4.198	-4.221	-4.222	-4.074	-4.210	-4.148	0.556	0.061
P50747	HLCS	1.953	1.922	2.054	2.017	1.937	2.036	0.057	0.098
Q6ZMI0	PPP1R21	-2.395	-2.512	-2.355	-2.344	-2.454	-2.350	0.323	0.104
Q15048	LRRC14	-1.583	-1.617	-1.673	-1.593	-1.600	-1.633	0.561	-0.033

Q5JPI9	EEF1AKMT2	-1.196	-1.339	-1.445	-1.335	-1.268	-1.390	0.314	-0.122
Q9Y337	KLK5	-4.235	-3.893	-4.190	-4.005	-4.064	-4.097	0.883	-0.033
Q9NV64	TMEM39A	-3.126	-3.122	-3.205	-2.982	-3.124	-3.094	0.829	0.031
A6NDU8	C5orf51	0.586	0.541	0.644	0.582	0.563	0.613	0.330	0.050
Q8IYL2	TRMT44	-2.799	-2.877	-2.948	-2.783	-2.838	-2.865	0.800	-0.028
P49327	FASN	7.219	7.317	7.361	7.332	7.268	7.346	0.341	0.079
Q6UW68	TMEM205	0.959	0.843	0.772	0.774	0.901	0.773	0.270	-0.128
Q13616	CUL1	4.378	4.306	4.312	4.252	4.342	4.282	0.334	-0.060
A4D2B0	MBLAC1	-2.840	-2.921	-3.040	-2.975	-2.881	-3.008	0.141	-0.127
Q8NEZ3	WDR19	-0.432	-0.521	-0.407	-0.395	-0.477	-0.401	0.336	0.076
Q9GZS3	WDR61	1.577	1.818	1.603	1.576	1.698	1.590	0.534	-0.108
Q9P258	RCC2	5.245	5.224	5.294	5.256	5.235	5.275	0.236	0.040
P52789	HK2	3.135	3.076	3.136	3.139	3.106	3.137	0.479	0.032
Q9BVI4	NOC4L	2.760	2.701	2.767	2.741	2.731	2.754	0.574	0.023
Q6IQ32	ADNP2	-0.080	-0.073	-0.146	-0.027	-0.076	-0.086	0.893	-0.010
Q15554	TERF2	1.063	0.990	1.027	1.008	1.027	1.017	0.838	-0.010
P20674	COX5A	2.704	2.729	2.676	2.618	2.717	2.647	0.212	-0.070
Q658Y4	FAM91A1	1.667	1.672	1.608	1.569	1.669	1.589	0.144	-0.081
P31689	DNAJA1	1.318	1.323	1.303	1.254	1.320	1.278	0.330	-0.042
Q7RTV0	PHF5A	2.959	2.881	3.147	3.051	2.920	3.099	0.107	0.179
Q9NYF8	BCLAF1	-2.844	-2.851	-2.731	-2.721	-2.847	-2.726	0.004	0.121
Q9Y485	DMXL1	0.229	0.125	0.161	0.133	0.177	0.147	0.663	-0.030
Q9NV31	IMP3	1.898	1.926	1.958	1.937	1.912	1.947	0.193	0.035
O75815	BCAR3	-2.112	-2.237	-2.456	-2.267	-2.174	-2.361	0.258	-0.187
Q14469	HES1	-0.170	-0.089	-0.074	0.000	-0.129	-0.037	0.236	0.093
Q04917	YWHAH	3.299	3.288	3.363	3.369	3.294	3.366	0.018	0.072
Q12929	EPS8	0.481	0.504	0.302	0.273	0.492	0.287	0.010	-0.205
Q08380	LGALS3BP	-0.097	0.609	0.895	0.374	0.256	0.634	0.485	0.379
Q8WVY7	UBLCP1	3.462	3.407	3.405	3.437	3.434	3.421	0.721	-0.014
Q15843	NEDD8	3.493	3.451	3.495	3.464	3.472	3.479	0.818	0.007
Q13546	RIPK1	1.074	0.889	0.782	0.770	0.982	0.776	0.268	-0.206
Q8N983	MRPL43	-3.723	-3.507	-3.548	-3.643	-3.615	-3.596	0.891	0.019
Q92616	GCN1	6.300	6.256	6.227	6.212	6.278	6.220	0.204	-0.058
Q6P5R6	RPL22L1	-0.795	-0.431	-0.565	-0.653	-0.613	-0.609	0.984	0.004
Q8N680	ZBTB2	-1.912	-1.825	-1.953	-1.812	-1.868	-1.883	0.881	-0.014

P61026	RAB10	2.327	2.355	2.431	2.475	2.341	2.453	0.066	0.112
Q9UID3	VPS51	-2.313	-2.084	-2.324	-2.065	-2.198	-2.195	0.985	0.004
P02760	AMBP	-3.024	-3.123	-2.952	-3.300	-3.073	-3.126	0.813	-0.053
Q9UNY4	TTF2	-0.241	-0.307	-0.220	-0.224	-0.274	-0.222	0.359	0.052
Q8TAD7	OCC1	-3.500	-3.484	-3.310	-3.701	-3.492	-3.506	0.955	-0.014
P49917	LIG4	-0.103	-0.226	0.005	-0.105	-0.165	-0.050	0.300	0.115
Q8WUH1	CHURC1	-1.943	-2.071	-1.705	-1.745	-2.007	-1.725	0.118	0.282
Q96QD8	SLC38A2	1.904	1.796	2.084	1.963	1.850	2.024	0.166	0.174
O94905	ERLIN2	3.175	3.149	3.257	3.194	3.162	3.226	0.261	0.064
Q6QNY0	BLOC1S3	0.113	0.100	0.141	0.016	0.106	0.078	0.732	-0.028
Q9UQR0	SCML2	-0.311	-0.164	-0.280	-0.237	-0.238	-0.258	0.825	-0.021
Q92785	DPF2	-2.375	-2.194	-2.425	-2.359	-2.284	-2.392	0.433	-0.108
Q9BY49	PECR	-0.383	-0.487	-0.380	-0.417	-0.435	-0.399	0.612	0.036
P00747	PLG	-2.705	-2.724	-2.674	-2.549	-2.714	-2.611	0.343	0.103
Q86U44	METTL3	2.233	2.136	2.109	2.119	2.184	2.114	0.383	-0.070
Q6ZRQ5	MMS22L	-0.270	-0.210	-0.432	-0.387	-0.240	-0.409	0.052	-0.169
Q16795	NDUFA9	3.839	3.847	3.865	3.895	3.843	3.880	0.226	0.037
Q9P2J5	LARS1	1.904	1.911	1.863	1.874	1.908	1.868	0.041	-0.039
O76080	ZFAND5	-0.890	-0.862	-0.829	-0.747	-0.876	-0.788	0.250	0.088
P48436	SOX9	0.601	0.549	0.313	0.426	0.575	0.370	0.126	-0.205
O00459	PIK3R2	-0.060	-0.075	-0.157	-0.096	-0.067	-0.126	0.293	-0.059
O43169	CYB5B	2.868	2.793	2.874	2.875	2.831	2.875	0.449	0.044
Q96BH1	RNF25	-0.671	-0.764	-0.733	-0.742	-0.718	-0.737	0.746	-0.020
Q8NFA0	USP32	0.147	0.094	0.115	0.191	0.120	0.153	0.563	0.033
P01023	A2M	-3.538	-3.639	-3.524	-3.709	-3.588	-3.617	0.819	-0.028
Q9H6Y7	RNF167	-1.699	-1.770	-1.828	-1.979	-1.735	-1.904	0.229	-0.169
O60443	GSDME	0.292	0.131	0.097	0.113	0.211	0.105	0.411	-0.106
O75874	IDH1	5.739	5.761	5.889	5.844	5.750	5.866	0.076	0.116
Q9BRV8	SIKE1	-3.514	-3.589	-3.549	-3.578	-3.551	-3.564	0.803	-0.012
Q9GZX9	TWSG1	-2.112	-2.319	-2.095	-2.165	-2.215	-2.130	0.557	0.085
Q9BQE4	SELENOS	-1.141	-1.092	-0.974	-0.980	-1.116	-0.977	0.107	0.140
P04066	FUCA1	-0.403	-0.102	0.030	-0.122	-0.253	-0.046	0.380	0.207
Q96GD0	PDXP	-0.102	-0.139	-0.159	-0.093	-0.121	-0.126	0.904	-0.005
Q99594	TEAD3	-1.859	-1.853	-1.957	-1.955	-1.856	-1.956	0.008	-0.100
Q8NC44	RETREG2	-2.482	-2.182	-2.607	-2.363	-2.332	-2.485	0.515	-0.153

Q9UHR6	ZNHIT2	0.183	0.108	0.136	0.166	0.145	0.151	0.904	0.006
Q9P013	CWC15	2.358	2.205	2.340	2.251	2.281	2.296	0.887	0.015
P27797	CALR	6.316	6.234	6.145	6.116	6.275	6.130	0.147	-0.145
Q9BZH6	WDR11	2.719	2.691	2.688	2.630	2.705	2.659	0.330	-0.046
O95503	CBX6	-3.376	-3.241	-3.181	-3.135	-3.309	-3.158	0.241	0.151
Q66LE6	PPP2R2D	-1.214	-1.452	-1.365	-1.439	-1.333	-1.402	0.667	-0.069
P42345	MTOR	3.683	3.644	3.654	3.670	3.664	3.662	0.951	-0.002
Q9HDC5	JPH1	-1.368	-1.250	-1.322	-1.254	-1.309	-1.288	0.795	0.021
P62341	SELENOT	0.166	0.176	0.125	0.170	0.171	0.147	0.480	-0.024
Q96FZ2	HMCES	2.391	2.294	2.162	2.194	2.342	2.178	0.157	-0.164
P61599	NAA20	-0.435	-0.437	-0.667	-0.476	-0.436	-0.572	0.392	-0.135
O00418	EEF2K	1.355	1.298	1.243	1.249	1.327	1.246	0.211	-0.080
Q9Y5R8	TRAPPC1	0.781	0.736	0.777	0.797	0.759	0.787	0.412	0.028
Q9H9G7	AGO3	-2.589	-2.635	-2.549	-2.630	-2.612	-2.589	0.686	0.023
P02787	TF	-0.501	-0.491	-0.232	-0.419	-0.496	-0.325	0.318	0.171
Q8TF72	SHROOM3	-1.709	-1.555	-1.667	-1.670	-1.632	-1.669	0.718	-0.036
P43243	MATR3	3.908	3.911	3.886	3.862	3.909	3.874	0.200	-0.036
P16333	NCK1	-1.051	-1.074	-1.014	-1.063	-1.063	-1.039	0.502	0.024
O43159	RRP8	1.526	1.445	1.536	1.554	1.485	1.545	0.374	0.060
P17028	ZNF24	-1.387	-1.370	-1.483	-1.417	-1.379	-1.450	0.259	-0.071
Q8IU60	DCP2	-1.468	-1.573	-1.238	-1.269	-1.521	-1.253	0.101	0.267
O75844	ZMPSTE24	4.610	4.559	4.655	4.555	4.584	4.605	0.756	0.021
Q6NSJ0	MYORG	-3.346	-3.300	-3.210	-3.162	-3.323	-3.186	0.054	0.137
O75391	SPAG7	1.365	1.323	1.240	1.229	1.344	1.234	0.106	-0.110
Q15311	RALBP1	0.616	0.528	0.567	0.521	0.572	0.544	0.646	-0.028
Q15008	PSMD6	1.991	1.823	2.005	1.922	1.907	1.964	0.623	0.057
Q96C86	DCPS	1.752	1.738	1.800	1.799	1.745	1.800	0.076	0.055
P51397	DAP	1.166	1.266	1.136	1.182	1.216	1.159	0.445	-0.057
Q8NEY8	PPHLN1	-3.406	-3.558	-3.411	-3.280	-3.482	-3.345	0.308	0.137
Q6ZNB6	NFXL1	-0.188	-0.150	-0.012	-0.038	-0.169	-0.025	0.033	0.144
Q96B54	ZNF428	-1.168	-1.239	-1.343	-1.285	-1.204	-1.314	0.144	-0.111
Q6P3W7	SCYL2	2.553	2.491	2.476	2.462	2.522	2.469	0.328	-0.053
P56270	MAZ	-1.444	-1.587	-1.494	-1.548	-1.515	-1.521	0.948	-0.006
Q96BN2	TADA1	-1.330	-1.322	-1.284	-1.316	-1.326	-1.300	0.336	0.026
Q8NCS4	TMEM35B	-2.781	-2.695	-2.545	-2.474	-2.738	-2.510	0.058	0.229

O15484	CAPN5	-0.985	-0.973	-1.076	-0.934	-0.979	-1.005	0.780	-0.026
Q99487	PAFAH2	-4.395	-4.360	-4.421	-4.367	-4.377	-4.394	0.659	-0.017
O43665	RGS10	-6.522	-6.696	-7.114	-7.000	-6.609	-7.057	0.065	-0.448
P42574	CASP3	1.548	1.479	1.524	1.569	1.513	1.546	0.523	0.033
Q16831	UPP1	-0.316	0.449	0.447	0.180	0.067	0.314	0.635	0.247
Q9BT78	COPS4	2.312	2.321	2.291	2.255	2.317	2.273	0.238	-0.044
O43291	SPINT2	-3.285	-3.046	-3.264	-3.261	-3.165	-3.263	0.565	-0.097
Q8NEM2	SHCBP1	-0.230	-0.240	-0.288	-0.247	-0.235	-0.267	0.352	-0.032
Q9BV44	THUMPD3	1.191	1.209	1.160	1.199	1.200	1.179	0.469	-0.021
P57768	SNX16	-3.105	-2.957	-3.075	-2.785	-3.031	-2.930	0.615	0.101
Q7Z417	NUFIP2	3.183	3.203	3.202	3.158	3.193	3.180	0.676	-0.012
Q96HY6	DDRGK1	-2.123	-2.109	-2.224	-1.939	-2.116	-2.081	0.848	0.035
Q9Y3E1	HDGFL3	-1.767	-1.896	-1.160	-1.304	-1.831	-1.232	0.026	0.599
Q9H553	ALG2	0.312	0.232	0.316	0.353	0.272	0.334	0.337	0.062
Q4U2R6	MRPL51	0.396	0.399	0.446	0.439	0.397	0.442	0.027	0.045
Q6VY07	PACS1	-2.439	-2.464	-2.550	-2.510	-2.452	-2.530	0.105	-0.078
Q9Y2H5	PLEKHA6	0.121	0.329	0.136	0.217	0.225	0.177	0.724	-0.048
P29317	EPHA2	3.449	3.441	3.360	3.324	3.445	3.342	0.095	-0.103
P62280	RPS11	6.049	5.907	5.980	5.826	5.978	5.903	0.550	-0.075
Q9UHG3	PCYOX1	0.869	0.950	0.923	0.941	0.910	0.932	0.681	0.022
P52732	KIF11	3.684	3.662	3.472	3.461	3.673	3.466	0.013	-0.207
Q92966	SNAPC3	-4.190	-4.197	-4.183	-4.109	-4.193	-4.146	0.419	0.047
Q6DD87	ZNF787	0.519	0.597	0.741	0.688	0.558	0.714	0.096	0.156
P06132	UROD	3.555	3.506	3.530	3.510	3.530	3.520	0.750	-0.010
Q5D1E8	ZC3H12A	-2.367	-2.306	-2.370	-2.241	-2.337	-2.306	0.722	0.031
Q6P9B6	MEAK7	1.387	1.457	1.274	1.351	1.422	1.312	0.173	-0.109
Q92562	FIG4	-0.313	-0.429	-0.263	-0.311	-0.371	-0.287	0.365	0.084
Q9Y6R7	FCGBP	-8.840	-9.380	-8.458	-9.125	-9.110	-8.791	0.538	0.319
O43933	PEX1	0.504	0.429	0.416	0.429	0.467	0.422	0.446	-0.044
P61758	VBP1	-0.794	-0.807	-0.827	-0.862	-0.800	-0.844	0.214	-0.044
Q9UQ90	SPG7	1.589	1.523	1.577	1.524	1.556	1.551	0.915	-0.005
O75683	SURF6	2.809	2.765	2.747	2.707	2.787	2.727	0.180	-0.060
Q9P0M6	MACROH2A2	1.078	0.965	1.763	1.727	1.022	1.745	0.034	0.724
P01111	NRAS	0.929	0.911	1.001	0.977	0.920	0.989	0.054	0.069
Q9NZ43	USE1	0.372	0.448	0.449	0.426	0.410	0.437	0.602	0.027

O75122	CLASP2	0.639	0.647	0.538	0.770	0.643	0.654	0.939	0.011
Q9NXW9	ALKBH4	-1.659	-1.630	-1.887	-1.606	-1.644	-1.747	0.599	-0.102
Q9BXS4	TMEM59	-0.782	-0.878	-0.696	-0.749	-0.830	-0.723	0.223	0.107
Q8TF76	HASPIN	-2.748	-2.576	-2.794	-2.568	-2.662	-2.681	0.908	-0.019
Q96KC8	DNAJC1	-0.142	-0.139	-0.096	-0.042	-0.141	-0.069	0.227	0.072
Q15785	TOMM34	4.631	4.715	4.508	4.557	4.673	4.532	0.131	-0.141
Q9NRV9	HEBP1	3.429	3.310	3.381	3.339	3.369	3.360	0.902	-0.009
P46776	RPL27A	4.485	4.480	4.449	4.471	4.483	4.460	0.284	-0.022
Q9UII2	ATP5IF1	1.849	1.725	1.679	1.587	1.787	1.633	0.195	-0.154
Q9UHD8	SEPTIN9	-1.593	-1.552	-1.694	-1.765	-1.573	-1.729	0.087	-0.157
Q99943	AGPAT1	0.877	0.755	0.769	0.839	0.816	0.804	0.881	-0.012
Q9P287	BCCIP	1.923	1.888	1.730	1.670	1.905	1.700	0.045	-0.206
P06280	GLA	1.480	1.368	1.376	1.415	1.424	1.396	0.699	-0.029
P31040	SDHA	-0.907	-0.866	-0.804	-0.823	-0.887	-0.814	0.131	0.073
Q8IV38	ANKMY2	0.411	0.243	0.259	0.261	0.327	0.260	0.570	-0.067
Q9Y5M8	SRPRB	2.657	2.550	2.607	2.514	2.604	2.560	0.603	-0.043
Q9P2R6	RERE	-0.915	-0.858	-0.966	-0.792	-0.886	-0.879	0.947	0.007
Q9Y5A6	ZSCAN21	-1.436	-1.425	-1.463	-1.412	-1.431	-1.438	0.837	-0.007
Q8N697	SLC15A4	-1.498	-1.689	-1.556	-1.579	-1.593	-1.568	0.833	0.026
O00159	MYO1C	-2.012	-1.913	-1.896	-1.755	-1.963	-1.825	0.267	0.137
Q99456	KRT12	-0.278	-0.054	-0.573	-0.191	-0.166	-0.382	0.451	-0.217
P62906	RPL10A	6.890	6.762	6.879	6.811	6.826	6.845	0.821	0.019
Q9NRG9	AAAS	0.648	0.582	0.569	0.486	0.615	0.528	0.246	-0.087
Q9NWT8	AURKAIP1	-1.876	-1.841	-1.851	-1.831	-1.859	-1.841	0.489	0.018
Q8NBI6	XXYLT1	-1.928	-2.058	-2.074	-2.161	-1.993	-2.118	0.268	-0.125
Q8IUZ5	PHYKPL	-0.585	-0.604	-0.353	-0.299	-0.595	-0.326	0.040	0.269
Q9NPB8	GPCPD1	-1.722	-1.823	-1.516	-1.542	-1.772	-1.529	0.114	0.243
O00268	TAF4	0.942	0.986	0.868	0.877	0.964	0.872	0.137	-0.092
Q9P015	MRPL15	3.731	3.638	3.831	3.737	3.685	3.784	0.272	0.099
Q9UH62	ARMCX3	0.854	0.727	0.720	0.615	0.791	0.667	0.277	-0.123
Q9NX70	MED29	-1.689	-1.740	-1.874	-1.836	-1.714	-1.855	0.056	-0.140
Q9BYC8	MRPL32	1.053	0.992	0.966	0.963	1.022	0.964	0.307	-0.058
Q9UBS4	DNAJB11	3.173	3.113	3.146	3.102	3.143	3.124	0.669	-0.019
Q9H3S7	PTPN23	2.169	2.134	2.154	2.161	2.151	2.158	0.785	0.006
Q9BV10	ALG12	-2.262	-2.356	-2.225	-2.170	-2.309	-2.198	0.207	0.111

Q13151	HNRNPA0	3.741	3.790	3.666	3.677	3.766	3.671	0.150	-0.094
P49591	SARS1	5.261	5.252	5.299	5.279	5.256	5.289	0.141	0.033
Q9UBB9	TFIP11	1.908	1.899	1.934	1.918	1.904	1.926	0.156	0.022
Q5T0W9	FAM83B	-1.559	-1.389	-1.456	-1.547	-1.474	-1.502	0.810	-0.027
Q5VWZ2	LYPLAL1	0.599	0.503	0.638	0.646	0.551	0.642	0.306	0.091
Q9NQR4	NIT2	3.636	3.569	3.703	3.727	3.603	3.715	0.153	0.112
Q92805	GOLGA1	0.979	0.968	0.991	0.985	0.974	0.988	0.177	0.015
Q13242	SRSF9	5.997	5.919	6.046	5.956	5.958	6.001	0.547	0.043
P62316	SNRPD2	0.898	0.787	0.845	0.743	0.842	0.794	0.585	-0.048
Q9HC35	EML4	-1.530	-1.677	-1.540	-1.503	-1.604	-1.521	0.455	0.083
O94826	TOMM70	4.801	4.718	4.783	4.767	4.760	4.775	0.776	0.015
P17931	LGALS3	6.078	6.156	6.092	6.027	6.117	6.059	0.378	-0.058
Q66PJ3	ARL6IP4	-6.294	-6.506	-6.168	-6.307	-6.400	-6.237	0.345	0.163
Q99816	TSG101	1.463	1.582	1.320	1.326	1.522	1.323	0.184	-0.199
Q9GZN4	PRSS22	-5.793	-5.856	-5.874	-6.147	-5.825	-6.010	0.396	-0.186
Q07020	RPL18	0.100	0.113	-0.025	0.050	0.107	0.012	0.232	-0.095
Q16718	NDUFA5	-2.183	-2.205	-2.061	-2.075	-2.194	-2.068	0.017	0.126
Q9H0U6	MRPL18	1.798	1.764	1.837	1.785	1.781	1.811	0.450	0.030
P60953	CDC42	1.702	1.688	1.676	1.676	1.695	1.676	0.211	-0.019
Q7Z4H3	HDDC2	-3.617	-3.782	-3.606	-3.713	-3.700	-3.659	0.727	0.041
Q9BW19	KIFC1	1.980	1.942	1.866	1.912	1.961	1.889	0.142	-0.072
Q8WU58	FAM222B	-2.993	-2.947	-3.050	-3.029	-2.970	-3.040	0.161	-0.070
Q71UM5	RPS27L	-1.287	-1.470	-1.222	-1.314	-1.378	-1.268	0.427	0.110
O95336	PGLS	4.715	4.626	4.802	4.756	4.671	4.779	0.206	0.108
Q9BQ04	RBM4B	-1.034	-1.119	-1.138	-1.100	-1.077	-1.119	0.495	-0.042
Q9HAV4	XPO5	5.149	5.135	5.161	5.124	5.142	5.143	0.986	0.000
Q9Y3D0	CIAO2B	0.200	0.248	-0.002	-0.036	0.224	-0.019	0.020	-0.243
Q96B77	TMEM186	-0.112	-0.091	-0.194	-0.120	-0.101	-0.157	0.360	-0.056
Q8N8N7	PTGR2	-0.421	-0.436	-0.443	-0.382	-0.429	-0.413	0.690	0.016
Q15057	ACAP2	3.132	3.054	3.241	3.166	3.093	3.204	0.177	0.110
Q9NVN8	GNL3L	0.973	1.050	1.058	1.125	1.012	1.092	0.261	0.080
Q13107	USP4	-2.720	-2.777	-2.836	-2.774	-2.749	-2.805	0.309	-0.057
Q9NVX0	HAUS2	-1.886	-2.047	-2.204	-2.232	-1.966	-2.218	0.189	-0.252
Q8WWY3	PRPF31	1.674	1.663	1.683	1.653	1.668	1.668	0.989	0.000
P53680	AP2S1	-1.591	-1.674	-1.725	-1.752	-1.633	-1.739	0.214	-0.106

Q49AR2	C5orf22	-1.471	-1.519	-1.698	-1.530	-1.495	-1.614	0.380	-0.119
Q5T6F2	UBAP2	0.400	0.458	0.338	0.458	0.429	0.398	0.704	-0.031
Q13885	TUBB2A	3.697	3.664	3.302	3.217	3.680	3.259	0.039	-0.421
Q99990	VGLL1	-4.764	-4.742	-4.497	-4.758	-4.753	-4.627	0.510	0.126
Q9UPY5	SLC7A11	0.262	0.105	0.133	-0.030	0.183	0.052	0.364	-0.132
Q13825	AUH	-2.013	-2.161	-1.830	-1.962	-2.087	-1.896	0.196	0.191
P51665	PSMD7	3.622	3.599	3.513	3.562	3.610	3.538	0.167	-0.073
O75940	SMNDC1	2.331	2.300	2.302	2.305	2.316	2.303	0.568	-0.012
Q6PJG2	MIDEAS	1.104	1.228	1.084	1.166	1.166	1.125	0.641	-0.041
Q14997	PSME4	1.131	1.011	1.054	0.996	1.071	1.025	0.584	-0.046
Q86UP2	KTN1	-0.246	-0.349	-0.294	-0.302	-0.298	-0.298	0.996	0.000
Q8IZ83	ALDH16A1	-0.605	-0.828	-0.591	-0.655	-0.716	-0.623	0.552	0.093
Q03169	TNFAIP2	1.309	1.242	1.526	1.415	1.276	1.470	0.119	0.195
P19784	CSNK2A2	3.319	3.280	3.293	3.241	3.300	3.267	0.423	-0.033
Q13425	SNTB2	0.852	0.792	0.614	0.667	0.822	0.641	0.047	-0.181
P49447	CYB561	-3.507	-3.186	-3.621	-3.365	-3.347	-3.493	0.552	-0.147
P04040	CAT	3.116	3.075	3.111	3.070	3.096	3.091	0.872	-0.005
Q8IWW6	ARHGAP12	-3.196	-3.361	-2.851	-2.928	-3.279	-2.889	0.089	0.389
P49840	GSK3A	0.505	0.490	0.406	0.525	0.498	0.466	0.683	-0.032
Q9Y2T2	AP3M1	2.506	2.508	2.443	2.466	2.507	2.454	0.132	-0.052
Q99436	PSMB7	3.870	3.800	3.839	3.781	3.835	3.810	0.643	-0.025
P60900	PSMA6	3.425	3.435	3.236	3.327	3.430	3.282	0.186	-0.148
Q9UBQ0	VPS29	-4.065	-3.789	-4.037	-3.988	-3.927	-4.013	0.645	-0.086
Q8IWA0	WDR75	4.120	4.089	4.080	4.121	4.104	4.101	0.903	-0.004
Q9NRW3	APOBEC3C	1.383	1.396	1.010	1.088	1.390	1.049	0.065	-0.341
O75319	DUSP11	-1.234	-1.146	-1.086	-0.978	-1.190	-1.032	0.155	0.158
Q9UIU6	SIX4	-0.459	-0.508	-0.563	-0.589	-0.483	-0.576	0.113	-0.093
Q9H0X4	FAM234A	-3.279	-3.351	-3.306	-3.436	-3.315	-3.371	0.551	-0.055
P62910	RPL32	4.824	4.728	4.719	4.654	4.776	4.686	0.277	-0.090
Q16134	ETFDH	-4.422	-4.381	-4.468	-4.037	-4.401	-4.253	0.615	0.149
Q8N954	GPATCH11	0.420	0.375	0.256	0.292	0.397	0.274	0.054	-0.123
Q13415	ORC1	-0.301	-0.255	-0.429	-0.371	-0.278	-0.400	0.089	-0.122
Q92820	GGH	2.817	3.149	2.745	2.735	2.983	2.740	0.381	-0.243
O15121	DEGS1	-0.861	-0.815	-0.904	-0.920	-0.838	-0.912	0.163	-0.074
Q96AE7	TTC17	-5.593	-5.662	-5.814	-5.668	-5.628	-5.741	0.338	-0.113

Q53HC5	KLHL26	-1.767	-1.731	-1.900	-1.761	-1.749	-1.831	0.443	-0.081
Q6ZNW5	GDPGP1	-3.311	-2.974	-3.171	-3.017	-3.142	-3.094	0.827	0.049
Q7Z2K6	ERMP1	2.520	2.359	2.519	2.471	2.440	2.495	0.616	0.055
P17987	TCP1	6.710	6.661	6.693	6.652	6.686	6.672	0.722	-0.013
P26196	DDX6	4.448	4.478	4.445	4.460	4.463	4.452	0.605	-0.011
Q9NYB0	TERF2IP	2.137	2.085	2.109	2.086	2.111	2.097	0.699	-0.013
O75843	AP1G2	1.022	1.122	0.928	0.916	1.072	0.922	0.200	-0.150
P52657	GTF2A2	0.421	0.256	0.380	0.318	0.338	0.349	0.919	0.011
Q969R8	ITFG2	-1.795	-1.851	-1.602	-1.775	-1.823	-1.688	0.347	0.134
Q7Z4L5	TTC21B	-1.434	-1.455	-1.478	-1.397	-1.445	-1.437	0.889	0.007
Q8NBF2	NHLRC2	1.261	1.226	1.237	1.236	1.244	1.236	0.753	-0.007
Q86X95	CIR1	-4.482	-4.680	-4.550	-4.484	-4.581	-4.517	0.636	0.064
Q86YQ8	CPNE8	-2.276	-2.330	-2.205	-2.183	-2.303	-2.194	0.116	0.109
A0AVT1	UBA6	-1.468	-1.558	-1.361	-1.525	-1.513	-1.443	0.550	0.070
P50583	NUDT2	0.416	0.411	0.412	0.490	0.413	0.451	0.513	0.038
P26651	ZFP36	-2.975	-3.177	-2.975	-3.063	-3.076	-3.019	0.676	0.057
Q96BR5	COA7	2.353	2.274	2.431	2.327	2.313	2.379	0.428	0.065
O75175	CNOT3	2.560	2.536	2.581	2.565	2.548	2.573	0.238	0.025
Q9NYW8	RBAK	-3.333	-3.425	-3.292	-3.458	-3.379	-3.375	0.971	0.004
Q96GG9	DCUN1D1	2.989	2.954	2.830	2.846	2.972	2.838	0.045	-0.133
P15036	ETS2	-3.654	-3.464	-3.244	-3.200	-3.559	-3.222	0.160	0.337
O95363	FARS2	0.961	0.881	0.799	0.836	0.921	0.817	0.191	-0.104
P02533	KRT14	-3.523	-3.632	-4.295	-4.315	-3.577	-4.305	0.041	-0.728
P50897	PPT1	-1.861	-1.827	-1.718	-1.758	-1.844	-1.738	0.059	0.106
Q9NVR5	DNAAF2	-2.018	-2.111	-2.013	-2.161	-2.065	-2.087	0.826	-0.022
Q5TEA3	DNAAF9	-3.484	-3.490	-3.473	-3.268	-3.487	-3.370	0.459	0.117
Q99661	KIF2C	0.359	0.469	0.350	0.340	0.414	0.345	0.426	-0.069
P55196	AFDN	-6.430	-6.507	-6.456	-6.352	-6.468	-6.404	0.431	0.064
Q49B96	COX19	1.678	1.696	1.693	1.742	1.687	1.718	0.411	0.031
Q8WUH2	TGFBRAP1	0.666	0.564	0.652	0.659	0.615	0.656	0.573	0.040
Q96CC6	RHBDF1	-3.034	-2.967	-3.049	-2.879	-3.001	-2.964	0.744	0.037
Q00796	SORD	3.827	3.822	3.747	3.774	3.824	3.761	0.122	-0.064
Q86WX3	RPS19BP1	1.093	1.015	1.090	1.041	1.054	1.066	0.826	0.012
Q3LXA3	TKFC	0.388	0.303	0.486	0.352	0.346	0.419	0.468	0.073
Q14651	PLS1	2.538	2.728	2.218	2.222	2.633	2.220	0.143	-0.413

P49792	RANBP2	5.740	5.671	5.759	5.717	5.705	5.738	0.515	0.033
Q92769	HDAC2	-2.925	-2.902	-3.459	-2.926	-2.914	-3.193	0.485	-0.279
Q9Y5S1	TRPV2	0.736	0.676	0.419	0.395	0.706	0.407	0.038	-0.299
Q13867	BLMH	3.012	2.921	2.993	3.026	2.966	3.009	0.513	0.043
Q96GY0	ZC2HC1A	1.157	1.178	1.105	1.132	1.168	1.119	0.114	-0.049
P10253	GAA	3.042	2.986	3.158	3.094	3.014	3.126	0.123	0.112
Q9P2W1	PSMC3IP	-3.166	-2.956	-3.356	-3.286	-3.061	-3.321	0.219	-0.260
Q8NDH2	CCDC168	-4.334	-4.328	-4.280	-4.197	-4.331	-4.238	0.267	0.093
Q08211	DHX9	6.437	6.383	6.431	6.406	6.410	6.418	0.821	0.008
Q8TEP8	CEP192	-1.746	-1.773	-1.958	-1.901	-1.760	-1.929	0.066	-0.170
O95376	ARIH2	1.706	1.695	1.640	1.710	1.701	1.675	0.590	-0.026
Q7L5N7	LPCAT2	-0.539	-0.110	-0.381	-0.259	-0.324	-0.320	0.986	0.005
Q9HBM0	VEZT	-2.484	-2.499	-2.630	-2.415	-2.491	-2.523	0.820	-0.031
Q7Z3J2	VPS35L	-3.476	-3.562	-3.331	-3.601	-3.519	-3.466	0.764	0.053
Q9Y4L1	HYOU1	4.925	4.880	5.005	5.062	4.902	5.033	0.074	0.131
O00479	HMGN4	1.007	0.818	1.366	1.284	0.913	1.325	0.103	0.412
Q7Z4H8	POGLUT3	-0.997	-1.107	-1.076	-1.047	-1.052	-1.062	0.889	-0.010
Q9Y2B0	CNPY2	1.590	1.559	1.675	1.669	1.574	1.672	0.090	0.098
Q9H840	GEMIN7	-0.744	-0.839	-0.966	-0.936	-0.791	-0.951	0.157	-0.160
Q08J23	NSUN2	-0.927	-1.039	-0.881	-0.919	-0.983	-0.900	0.364	0.082
P00352	ALDH1A1	7.558	7.400	7.356	7.361	7.479	7.358	0.369	-0.121
Q9BV38	WDR18	3.003	2.948	3.080	3.051	2.976	3.066	0.140	0.090
Q9Y2U8	LEMD3	1.732	1.763	1.804	1.828	1.748	1.816	0.076	0.069
P52294	KPNA1	1.886	1.790	1.843	1.815	1.838	1.829	0.879	-0.009
P17661	DES	0.518	0.422	0.379	0.467	0.470	0.423	0.546	-0.047
Q7L5D6	GET4	-2.042	-2.020	-2.003	-1.980	-2.031	-1.992	0.129	0.039
Q86SX6	GLRX5	1.177	1.265	1.131	1.047	1.221	1.089	0.163	-0.132
Q9Y478	PRKAB1	1.594	1.629	1.510	1.631	1.612	1.571	0.618	-0.041
Q9NSD4	ZNF275	-6.503	-5.907	-6.102	-5.830	-6.205	-5.966	0.568	0.239
Q9NZP8	C1RL	-3.965	-3.790	-3.760	-3.398	-3.878	-3.579	0.318	0.299
P46777	RPL5	5.879	5.824	5.891	5.829	5.852	5.860	0.858	0.008
Q15031	LARS2	2.763	2.771	2.672	2.716	2.767	2.694	0.177	-0.073
Q9Y5J6	TIMM10B	-1.814	-1.883	-1.753	-1.736	-1.848	-1.744	0.188	0.104
O00767	SCD	0.600	0.600	0.948	0.911	0.600	0.929	0.036	0.329
P61513	RPL37A	4.096	4.044	4.056	4.064	4.070	4.060	0.769	-0.010

O95563	MPC2	0.157	0.402	0.219	0.318	0.279	0.269	0.946	-0.011
Q13111	CHAF1A	-1.370	-1.482	-1.677	-1.572	-1.426	-1.624	0.123	-0.199
Q14202	ZMYM3	-2.630	-2.684	-2.664	-2.699	-2.657	-2.682	0.538	-0.025
P24588	AKAP5	-3.161	-3.076	-3.196	-3.170	-3.118	-3.183	0.356	-0.064
Q8IVT2	MISP	-0.093	0.396	0.171	0.199	0.151	0.185	0.912	0.034
O96005	CLPTM1	-4.692	-4.640	-4.745	-4.959	-4.666	-4.852	0.319	-0.186
Q9NWB6	ARGLU1	1.646	1.495	1.827	1.604	1.570	1.716	0.405	0.145
Q9NRK6	ABCB10	1.581	1.446	1.573	1.551	1.513	1.562	0.601	0.049
Q9BRQ0	PYGO2	-0.047	-0.077	0.000	-0.011	-0.062	-0.005	0.132	0.057
Q96IW7	SEC22A	-4.759	-4.601	-4.681	-4.635	-4.680	-4.658	0.827	0.022
Q9BW92	TARS2	1.429	1.349	1.374	1.388	1.389	1.381	0.877	-0.008
Q14147	DHX34	-0.369	-0.452	-0.461	-0.382	-0.410	-0.422	0.862	-0.011
P27449	ATP6V0C	1.918	1.920	1.941	1.871	1.919	1.906	0.768	-0.013
Q9NWR8	MCUB	-0.813	-0.907	-0.825	-0.828	-0.860	-0.826	0.607	0.033
O75718	CRTAP	2.950	2.870	2.813	2.875	2.910	2.844	0.332	-0.066
Q9Y5B0	CTDP1	-7.140	-7.012	-6.947	-7.221	-7.076	-7.084	0.965	-0.008
Q9UBP6	METTL1	0.260	0.301	0.292	0.326	0.281	0.309	0.407	0.028
O75529	TAF5L	-2.513	-2.573	-2.529	-2.444	-2.543	-2.487	0.405	0.056
Q9BVA6	FICD	-4.870	-4.978	-4.697	-4.830	-4.924	-4.763	0.206	0.161
Q9NRP2	CMC2	0.665	0.630	0.508	0.558	0.647	0.533	0.075	-0.114
Q9HC21	SLC25A19	-1.153	-1.155	-1.141	-1.098	-1.154	-1.119	0.351	0.035
Q9C0C9	UBE2O	3.465	3.391	3.377	3.393	3.428	3.385	0.447	-0.043
Q9Y6D5	ARFGEF2	2.009	2.042	2.013	2.024	2.025	2.019	0.753	-0.007
P02765	AHSG	0.906	0.794	0.768	0.600	0.850	0.684	0.258	-0.166
P49750	YLPM1	0.970	0.908	0.846	0.958	0.939	0.902	0.636	-0.037
Q9Y277	VDAC3	0.292	0.494	0.250	0.343	0.393	0.296	0.506	-0.097
Q9UKS6	PACSIN3	1.845	1.778	1.745	1.779	1.811	1.762	0.354	-0.050
Q9BV79	MECR	-2.143	-2.146	-1.827	-1.892	-2.144	-1.859	0.071	0.285
P31641	SLC6A6	-3.808	-3.557	-3.822	-3.578	-3.682	-3.700	0.927	-0.018
P17480	UBTF	0.768	0.687	0.727	0.758	0.727	0.742	0.779	0.015
Q9Y248	GINS2	-1.573	-1.470	-2.051	-1.847	-1.522	-1.949	0.101	-0.427
P82921	MRPS21	0.137	0.020	0.103	-0.028	0.079	0.037	0.686	-0.041
Q9NRG0	CHRAC1	0.889	0.909	0.772	0.728	0.899	0.750	0.056	-0.149
Q13464	ROCK1	3.504	3.461	3.408	3.393	3.483	3.401	0.137	-0.082
Q5VTR2	RNF20	2.949	2.953	2.929	2.969	2.951	2.949	0.930	-0.002

P56182	RRP1	3.255	3.167	3.271	3.210	3.211	3.240	0.647	0.029
P52926	HMGA2	-4.802	-3.903	-3.841	-5.550	-4.353	-4.696	0.766	-0.343
Q13257	MAD2L1	2.581	2.599	2.329	2.380	2.590	2.354	0.045	-0.236
Q9H6E4	CCDC134	0.837	0.784	0.797	0.804	0.811	0.800	0.761	-0.010
Q9NWH9	SLTM	1.087	1.055	1.203	1.163	1.071	1.183	0.052	0.112
P42858	HTT	2.453	2.367	2.391	2.421	2.410	2.406	0.936	-0.004
Q9BXJ9	NAA15	4.378	4.345	4.314	4.312	4.361	4.313	0.212	-0.048
O75446	SAP30	0.122	0.254	0.129	0.333	0.188	0.231	0.763	0.043
Q5TGL8	PXDC1	-1.429	-1.592	-1.338	-1.582	-1.510	-1.460	0.768	0.050
O75467	ZNF324	-4.249	-4.216	-4.449	-4.205	-4.233	-4.327	0.581	-0.094
P05161	ISG15	1.249	1.097	1.621	1.427	1.173	1.524	0.111	0.351
P51784	USP11	3.006	3.017	3.067	3.118	3.012	3.092	0.181	0.080
Q5T1M5	FKBP15	-1.694	-1.700	-1.722	-1.823	-1.697	-1.773	0.374	-0.075
Q5JSZ5	PRRC2B	-0.277	-0.339	-0.300	-0.277	-0.308	-0.289	0.653	0.019
P02771	AFP	-0.839	-0.953	-1.082	-1.095	-0.896	-1.088	0.179	-0.193
Q86UZ6	ZBTB46	-4.461	-4.496	-4.469	-4.389	-4.479	-4.429	0.412	0.050
P41250	GARS1	-0.731	-0.995	-0.944	-0.774	-0.863	-0.859	0.982	0.004
Q96D46	NMD3	2.587	2.537	2.586	2.578	2.562	2.582	0.563	0.020
Q01484	ANK2	-3.491	-3.608	-3.534	-3.518	-3.550	-3.526	0.754	0.024
Q08AF3	SLFN5	1.189	1.238	1.651	1.631	1.214	1.641	0.018	0.427
Q9H4L4	SENP3	1.851	1.723	1.898	1.795	1.787	1.847	0.548	0.059
P09651	HNRNPA1	-1.595	-1.631	-1.487	-1.545	-1.613	-1.516	0.127	0.097
P09467	FBP1	-2.314	-2.151	-2.235	-2.486	-2.232	-2.361	0.496	-0.128
Q9P0J6	MRPL36	-0.139	-0.258	-0.154	-0.188	-0.198	-0.171	0.723	0.028
Q4G0F5	VPS26B	1.534	1.408	1.243	1.268	1.471	1.255	0.169	-0.216
P48509	CD151	0.985	1.017	0.991	0.890	1.001	0.941	0.436	-0.060
Q9BRQ8	AIFM2	-1.638	-1.514	-1.390	-1.569	-1.576	-1.480	0.479	0.096
P61218	POLR2F	-0.868	-0.928	-0.836	-0.841	-0.898	-0.838	0.292	0.060
Q2NKX8	ERCC6L	3.380	3.277	3.147	3.170	3.328	3.159	0.173	-0.170
Q67FW5	B3GNTL1	-4.255	-4.379	-4.298	-4.281	-4.317	-4.289	0.734	0.027
Q8N961	ABTB2	-3.399	-3.367	-3.368	-3.435	-3.383	-3.401	0.685	-0.018
Q9UHV9	PFDN2	3.951	3.933	3.892	3.904	3.942	3.898	0.067	-0.045
Q02978	SLC25A11	0.896	0.862	0.892	0.906	0.879	0.899	0.432	0.020
Q9UII4	HERC5	-2.137	-2.295	-1.742	-1.653	-2.216	-1.698	0.048	0.519
Q9NWZ8	GEMIN8	-0.630	-0.670	-0.635	-0.742	-0.650	-0.688	0.607	-0.038

Q15436	SEC23A	1.849	1.895	1.813	1.894	1.872	1.853	0.735	-0.019
Q8WUD4	CCDC12	0.539	0.548	0.534	0.509	0.543	0.522	0.317	-0.022
Q13418	ILK	-0.903	-0.833	-0.996	-0.805	-0.868	-0.901	0.793	-0.033
Q14344	GNA13	-0.590	-0.781	-0.522	-0.552	-0.686	-0.537	0.358	0.149
Q9Y448	KNSTRN	-1.417	-1.463	-1.219	-1.351	-1.440	-1.285	0.230	0.155
Q5PRF9	SAMD4B	0.173	0.244	0.135	0.177	0.208	0.156	0.355	-0.053
Q14210	LY6D	-3.010	-2.732	-2.796	-2.622	-2.871	-2.709	0.443	0.162
Q9Y530	OARD1	1.027	1.050	1.102	1.029	1.039	1.066	0.590	0.027
Q96E16	SMIM19	-3.034	-2.840	-2.930	-2.877	-2.937	-2.903	0.789	0.034
P68104	EEF1A1	0.451	0.725	-0.070	0.395	0.588	0.163	0.283	-0.426
Q9Y678	COPG1	4.521	4.453	4.451	4.396	4.487	4.424	0.289	-0.064
P20618	PSMB1	3.892	3.831	3.784	3.808	3.861	3.796	0.251	-0.065
P21291	CSRP1	2.911	2.903	2.851	2.793	2.907	2.822	0.203	-0.085
Q13129	RLF	-0.397	-0.492	-0.576	-0.518	-0.445	-0.547	0.234	-0.102
Q6UX53	METTL7B	-2.312	-1.581	-2.016	-1.844	-1.946	-1.930	0.972	0.016
Q9BW30	TPPP3	-2.719	-1.455	-2.416	-2.188	-2.087	-2.302	0.792	-0.215
Q99569	PKP4	-7.834	-7.640	-7.757	-7.622	-7.737	-7.690	0.732	0.047
Q9Y376	CAB39	1.794	1.968	1.828	1.839	1.881	1.834	0.682	-0.047
Q9BXW6	OSBPL1A	0.333	0.284	0.136	0.220	0.309	0.178	0.143	-0.131
Q13263	TRIM28	3.266	3.342	3.250	3.261	3.304	3.256	0.424	-0.048
Q0VDF9	HSPA14	3.780	3.726	3.662	3.623	3.753	3.643	0.091	-0.110
P22695	UQCRC2	5.015	5.037	5.150	5.107	5.026	5.129	0.082	0.103
Q9BTY7	HGH1	0.162	0.186	0.036	0.150	0.174	0.093	0.384	-0.081
Q9Y2P8	RCL1	2.224	2.185	2.335	2.300	2.205	2.318	0.050	0.113
P00403	MT	3.108	3.174	3.080	3.036	3.141	3.058	0.188	-0.083
Q8NF37	LPCAT1	0.602	0.667	1.045	1.000	0.634	1.023	0.015	0.388
Q96GX2	ATXN7L3B	-2.067	-2.011	-2.230	-2.184	-2.039	-2.207	0.046	-0.168
Q9NQX4	MYO5C	0.157	0.426	0.165	0.225	0.291	0.195	0.601	-0.097
Q8IYL3	C1orf174	-1.177	-1.325	-1.237	-1.352	-1.251	-1.294	0.694	-0.043
Q12778	FOXO1	-5.497	-5.298	-5.034	-5.106	-5.398	-5.070	0.156	0.328
P11940	PABPC1	2.824	2.786	2.786	2.817	2.805	2.802	0.901	-0.003
Q6P158	DHX57	1.257	1.204	1.201	1.262	1.231	1.232	0.982	0.001
O95361	TRIM16	-4.371	-4.392	-3.752	-4.106	-4.381	-3.929	0.236	0.452
Q13586	STIM1	0.410	0.321	0.450	0.480	0.365	0.465	0.241	0.100
Q9NX31	OSER1	-3.226	-3.269	-3.539	-3.547	-3.247	-3.543	0.041	-0.295

Q9UGU0	TCF20	-5.256	-4.857	-5.033	-4.814	-5.057	-4.923	0.632	0.133
Q8WXD5	GEMIN6	0.667	0.663	0.429	0.437	0.665	0.433	0.003	-0.232
Q9UHN1	POLG2	0.640	0.480	0.620	0.609	0.560	0.614	0.621	0.054
Q12893	TMEM115	-2.802	-2.817	-2.850	-2.812	-2.809	-2.831	0.444	-0.022
Q9NP66	HMG20A	1.021	1.026	1.031	0.995	1.024	1.013	0.665	-0.010
P40692	MLH1	-0.361	-0.325	-0.525	-0.393	-0.343	-0.459	0.315	-0.116
Q15020	SART3	-1.016	-1.018	-0.636	-0.599	-1.017	-0.618	0.030	0.400
Q6UB99	ANKRD11	0.516	0.416	0.568	0.543	0.466	0.556	0.311	0.090
Q92876	KLK6	-0.746	-0.190	-0.347	-0.388	-0.468	-0.368	0.779	0.100
P21912	SDHB	3.951	3.963	4.015	3.986	3.957	4.001	0.163	0.044
P29083	GTF2E1	1.783	1.725	1.780	1.733	1.754	1.757	0.951	0.003
P12271	RLBP1	-3.222	-2.756	-3.215	-3.103	-2.989	-3.159	0.596	-0.170
Q9HAD4	WDR41	-2.002	-2.073	-1.846	-1.966	-2.038	-1.906	0.227	0.132
P07437	TUBB	4.321	4.373	4.240	4.314	4.347	4.277	0.274	-0.070
Q7RTP6	MICAL3	1.332	1.187	1.244	1.213	1.260	1.229	0.743	-0.031
O75508	CLDN11	-1.019	-1.160	-0.919	-0.719	-1.090	-0.819	0.171	0.271
P62805	H4	9.089	9.164	9.083	9.123	9.126	9.103	0.656	-0.023
P48729	CSNK1A1	-5.522	-5.420	-5.739	-5.292	-5.471	-5.515	0.876	-0.044
Q9C0H9	SRCIN1	-3.308	-3.446	-3.272	-3.414	-3.377	-3.343	0.765	0.034
Q9H7X3	ZNF696	-5.582	-5.298	-5.456	-5.423	-5.440	-5.439	0.998	0.000
Q9NZU5	LMCD1	-3.999	-3.803	-4.181	-3.864	-3.901	-4.022	0.592	-0.121
Q9H871	RMND5A	-0.150	-0.158	-0.183	-0.173	-0.154	-0.178	0.065	-0.024
P00734	F2	-1.310	-1.441	-1.134	-1.282	-1.376	-1.208	0.234	0.168
Q96QU6	ACCS	-4.945	-5.216	-5.049	-5.009	-5.080	-5.029	0.770	0.051
P15311	EZR	7.057	6.953	6.885	6.834	7.005	6.859	0.174	-0.146
Q8N9N7	LRRC57	0.655	0.603	0.682	0.637	0.629	0.659	0.467	0.031
Q8TCG2	PI4K2B	-0.515	-0.510	-0.500	-0.453	-0.513	-0.476	0.367	0.036
Q6PID8	KLHDC10	-6.267	-5.728	-6.035	-5.619	-5.998	-5.827	0.669	0.171
Q9Y5J1	UTP18	2.690	2.626	2.680	2.684	2.658	2.682	0.594	0.024
Q8WXX0	DNAH7	1.306	1.003	1.135	1.062	1.155	1.099	0.775	-0.056
Q9NV06	DCAF13	3.018	2.950	3.048	3.043	2.984	3.045	0.319	0.061
Q9HBK9	AS3MT	-0.590	-0.672	-0.756	-0.854	-0.631	-0.805	0.116	-0.174
A4D1U4	DENND11	-2.108	-2.037	-2.179	-1.993	-2.073	-2.086	0.911	-0.013
Q9HCY8	S100A14	-1.711	-1.401	-1.213	-1.371	-1.556	-1.292	0.308	0.264
P61421	ATP6V0D1	1.634	1.682	1.674	1.758	1.658	1.716	0.383	0.058

Q7Z7N9	TMEM179B	-2.033	-2.133	-1.862	-1.805	-2.083	-1.834	0.073	0.250
Q99798	ACO2	5.217	5.117	5.306	5.262	5.167	5.284	0.222	0.117
Q9Y312	AAR2	1.451	1.479	1.335	1.345	1.465	1.340	0.045	-0.125
Q6DD88	ATL3	3.550	3.592	3.655	3.587	3.571	3.621	0.359	0.050
Q9H773	DCTPP1	1.166	1.216	1.010	1.045	1.191	1.028	0.042	-0.163
Q9H3C7	GGNBP2	-3.656	-3.641	-3.823	-3.799	-3.649	-3.811	0.012	-0.162
Q6Q0C0	TRAF7	-3.769	-3.641	-3.615	-3.592	-3.705	-3.603	0.350	0.102
Q9UNK0	STX8	1.357	1.219	1.197	1.166	1.288	1.181	0.355	-0.107
P26358	DNMT1	-1.485	-1.537	-1.886	-1.822	-1.511	-1.854	0.016	-0.343
P49137	MAPKAPK2	-1.413	-1.499	-1.688	-1.665	-1.456	-1.676	0.104	-0.221
P54098	POLG	1.101	1.168	1.113	1.228	1.134	1.171	0.649	0.037
Q92674	CENPI	-2.619	-2.322	-2.755	-2.204	-2.470	-2.480	0.980	-0.009
Q03135	CAV1	0.715	0.694	0.524	0.480	0.704	0.502	0.035	-0.202
P07384	CAPN1	4.735	4.724	4.746	4.705	4.730	4.725	0.866	-0.004
O15031	PLXNB2	3.206	3.219	3.195	3.119	3.212	3.157	0.379	-0.055
Q15125	EBP	2.166	2.093	2.010	2.020	2.129	2.015	0.188	-0.114
Q9Y576	ASB1	-2.273	-2.144	-2.265	-2.049	-2.208	-2.157	0.730	0.052
Q8TEM1	NUP210	2.191	2.318	2.282	2.302	2.254	2.292	0.660	0.038
Q9Y3C6	PPIL1	3.110	3.001	3.145	3.030	3.056	3.087	0.728	0.032
Q86XI2	NCAPG2	-2.489	-2.412	-2.712	-2.634	-2.450	-2.673	0.056	-0.223
O95476	CTDNEP1	-1.836	-1.968	-1.864	-1.859	-1.902	-1.861	0.647	0.041
O94925	GLS	0.648	0.644	0.388	0.425	0.646	0.406	0.047	-0.239
Q9UI09	NDUFA12	0.386	0.350	0.614	0.525	0.368	0.570	0.102	0.202
P33316	DUT	-4.527	-4.413	-4.296	-4.115	-4.470	-4.205	0.155	0.264
Q8NBF6	AVL9	-1.103	-0.999	-1.130	-1.001	-1.051	-1.066	0.876	-0.015
P53367	ARFIP1	-2.307	-2.470	-2.323	-2.472	-2.388	-2.397	0.944	-0.009
Q3KQV9	UAP1L1	0.636	0.638	0.661	0.786	0.637	0.724	0.398	0.087
Q96BF6	NACC2	-2.969	-2.830	-2.859	-2.826	-2.899	-2.842	0.558	0.057
Q92665	MRPS31	3.070	3.029	3.023	2.929	3.049	2.976	0.341	-0.073
P34949	MPI	0.365	0.350	0.307	0.377	0.358	0.342	0.735	-0.015
Q86YB8	ERO1B	-1.522	-1.509	-1.408	-1.373	-1.516	-1.390	0.058	0.125
Q8N3D4	EHBP1L1	1.288	1.339	1.212	1.256	1.313	1.234	0.143	-0.080
Q8TD26	CHD6	-0.800	-0.788	-0.956	-0.872	-0.794	-0.914	0.209	-0.120
Q96BP2	CHCHD1	0.482	0.204	0.207	-0.067	0.343	0.070	0.298	-0.273
P19404	NDUFV2	2.198	2.139	2.243	2.164	2.168	2.203	0.556	0.035

Q92610	ZNF592	0.511	0.643	0.448	0.525	0.577	0.487	0.380	-0.091
Q6ZRV2	FAM83H	2.697	2.931	2.745	2.825	2.814	2.785	0.850	-0.028
Q9NQT5	EXOSC3	-0.124	-0.235	-0.321	-0.273	-0.180	-0.297	0.247	-0.118
Q8NBQ5	HSD17B11	3.386	3.293	3.349	3.344	3.340	3.347	0.909	0.007
P17612	PRKACA	-1.623	-1.797	-1.522	-1.764	-1.710	-1.643	0.702	0.067
Q9UPS8	ANKRD26	-2.745	-2.729	-3.098	-2.760	-2.737	-2.929	0.459	-0.192
O95816	BAG2	-5.064	-4.964	-5.073	-5.028	-5.014	-5.051	0.597	-0.037
Q9Y5U8	MPC1	-1.043	-0.919	-0.685	-0.847	-0.981	-0.766	0.177	0.215
Q969Z3	MTARC2	-0.787	-1.088	-0.567	-0.924	-0.937	-0.746	0.500	0.192
P54753	EPHB3	-2.433	-2.078	-2.526	-1.519	-2.256	-2.023	0.726	0.233
Q9NPA3	MID1IP1	-2.610	-2.341	-2.358	-2.145	-2.475	-2.252	0.328	0.223
Q9H7L9	SUDS3	0.772	0.765	0.674	0.670	0.769	0.672	0.004	-0.097
Q6P4A8	PLBD1	2.775	2.526	2.923	2.683	2.651	2.803	0.472	0.152
Q8N335	GPD1L	2.850	2.786	2.849	2.848	2.818	2.849	0.511	0.031
Q9H1C4	UNC93B1	-1.632	-1.599	-1.992	-1.875	-1.616	-1.933	0.097	-0.317
P38435	GGCX	-0.974	-0.990	-1.193	-1.043	-0.982	-1.118	0.317	-0.136
Q9UKF6	CPSF3	3.514	3.522	3.424	3.450	3.518	3.437	0.079	-0.081
P0C7P0	CISD3	-0.766	-0.666	-0.818	-0.736	-0.716	-0.777	0.446	-0.061
P51991	HNRNPA3	0.266	0.321	0.254	0.265	0.294	0.260	0.426	-0.034
O75427	LRCH4	0.629	0.665	0.656	0.656	0.647	0.656	0.694	0.009
Q9Y3E5	PTRH2	2.652	2.606	2.698	2.729	2.629	2.713	0.110	0.084
Q86UW9	DTX2	-4.001	-3.992	-3.884	-4.025	-3.996	-3.955	0.660	0.042
Q13126	MTAP	-4.101	-2.029	-2.564	-2.788	-3.065	-2.676	0.772	0.389
Q969S3	ZNF622	2.548	2.502	2.664	2.611	2.525	2.637	0.088	0.112
Q13423	NNT	2.339	2.515	2.801	2.646	2.427	2.723	0.130	0.296
Q9Y2H1	STK38L	-0.962	-0.776	-0.820	-0.880	-0.869	-0.850	0.874	0.019
Q9GZV5	WWTR1	-0.163	-0.345	-0.110	-0.219	-0.254	-0.164	0.504	0.090
Q86UX6	STK32C	-2.436	-2.482	-2.452	-2.450	-2.459	-2.451	0.785	0.008
P53350	PLK1	0.412	0.426	0.423	0.407	0.419	0.415	0.718	-0.004
Q14872	MTF1	-2.269	-2.313	-2.038	-2.199	-2.291	-2.119	0.261	0.172
Q96LJ7	DHRS1	0.564	0.537	0.216	0.294	0.551	0.255	0.059	-0.295
A8MXV4	NUDT19	2.910	2.770	2.926	2.972	2.840	2.949	0.345	0.109
Q8TDX7	NEK7	0.462	0.393	0.428	0.345	0.428	0.386	0.525	-0.041
Q6AHZ1	ZNF518A	-2.754	-2.593	-2.817	-2.631	-2.674	-2.724	0.721	-0.051
A0AVK6	E2F8	-5.624	-5.072	-5.613	-5.501	-5.348	-5.557	0.586	-0.209

Q16594	TAF9	-1.545	-1.537	-1.537	-1.471	-1.541	-1.504	0.464	0.037
Q8IYB8	SUPV3L1	3.561	3.479	3.667	3.627	3.520	3.647	0.154	0.126
P25490	YY1	0.569	0.508	0.556	0.541	0.538	0.549	0.796	0.010
Q9UK73	FEM1B	-2.023	-1.951	-1.797	-1.785	-1.987	-1.791	0.108	0.196
Q96LB3	IFT74	-0.464	-0.506	-0.439	-0.517	-0.485	-0.478	0.893	0.007
Q9Y5X1	SNX9	3.022	2.917	3.125	2.897	2.970	3.011	0.785	0.041
Q9H4K7	MTG2	-1.834	-1.739	-1.893	-1.747	-1.787	-1.820	0.748	-0.033
P57775	FBXW4	-2.651	-2.646	-2.630	-2.588	-2.649	-2.609	0.304	0.040
Q9NZ63	C9orf78	2.195	2.147	2.198	2.180	2.171	2.189	0.595	0.018
O60925	PFDN1	3.550	3.462	2.924	2.878	3.506	2.901	0.018	-0.604
Q00597	FANCC	-3.555	-3.664	-3.674	-3.607	-3.609	-3.641	0.681	-0.031
Q7L3B6	CDC37L1	0.414	0.368	0.425	0.516	0.391	0.470	0.299	0.079
Q96A59	MARVELD3	-4.522	-4.438	-4.337	-4.452	-4.480	-4.395	0.361	0.085
Q8IY67	RAVER1	1.141	1.068	0.993	1.125	1.104	1.059	0.623	-0.045
P63244	RACK1	7.363	7.287	7.258	7.198	7.325	7.228	0.189	-0.097
Q9UHQ9	CYB5R1	2.315	2.260	2.336	2.315	2.288	2.326	0.380	0.038
Q9NX55	HYPK	-0.235	-0.252	-0.419	-0.410	-0.244	-0.414	0.008	-0.171
P46019	PHKA2	0.286	0.155	0.214	0.160	0.220	0.187	0.708	-0.033
Q9BWQ6	YIPF2	-3.674	-3.662	-4.010	-3.763	-3.668	-3.887	0.326	-0.219
Q969R5	L3MBTL2	-2.330	-2.245	-2.256	-2.195	-2.288	-2.226	0.369	0.062
Q8WVK7	SKA2	-1.052	-1.261	-1.409	-1.541	-1.157	-1.475	0.146	-0.318
Q5TAX3	TUT4	0.107	0.044	0.136	0.228	0.075	0.182	0.212	0.107
Q92759	GTF2H4	0.290	0.336	0.299	0.329	0.313	0.314	0.985	0.001
O95721	SNAP29	2.460	2.389	2.340	2.281	2.424	2.311	0.138	-0.113
P23526	AHCY	5.344	5.234	5.299	5.297	5.289	5.298	0.897	0.009
Q8IYW5	RNF168	-2.655	-2.586	-2.742	-2.724	-2.620	-2.733	0.173	-0.112
Q8TAL5	C9orf43	-6.559	-6.693	-6.516	-6.344	-6.626	-6.430	0.221	0.196
Q86VR2	RETREG3	-0.368	-0.304	-0.700	-0.528	-0.336	-0.614	0.158	-0.278
O95163	ELP1	3.373	3.348	3.307	3.314	3.360	3.310	0.134	-0.050
Q8NC54	KCT2	-2.141	-2.187	-2.456	-2.431	-2.164	-2.443	0.019	-0.279
P30040	ERP29	4.339	4.276	4.402	4.382	4.308	4.392	0.206	0.084
O15020	SPTBN2	-2.930	-2.593	-2.671	-2.456	-2.762	-2.564	0.442	0.198
P40855	PEX19	-3.121	-3.147	-3.514	-3.500	-3.134	-3.507	0.006	-0.373
Q96ND0	FAM210A	0.110	0.035	0.192	0.183	0.072	0.188	0.194	0.116
Q9BTV4	TMEM43	2.412	2.358	2.417	2.384	2.385	2.400	0.684	0.015

Q8N1P7	CRYBG2	-1.159	-1.000	-1.186	-1.061	-1.079	-1.123	0.708	-0.044
Q8WUJ0	STYX	-3.029	-3.073	-3.189	-3.015	-3.051	-3.102	0.660	-0.051
P04114	APOB	1.113	0.891	1.119	1.084	1.002	1.101	0.534	0.099
Q9P016	THYN1	1.217	0.954	1.131	0.910	1.086	1.020	0.742	-0.065
Q9H0U3	MAGT1	-0.378	-0.275	-0.305	-0.253	-0.326	-0.279	0.524	0.047
P16885	PLCG2	1.647	1.544	1.575	1.569	1.595	1.572	0.732	-0.023
P56192	MARS1	3.211	3.091	3.285	3.156	3.151	3.220	0.512	0.070
Q96GF1	RNF185	-6.837	-7.147	-6.515	-6.891	-6.992	-6.703	0.362	0.289
Q09328	MGAT5	-2.673	-2.799	-2.548	-2.511	-2.736	-2.530	0.166	0.206
Q9Y2W1	THRAP3	4.939	4.880	4.996	4.942	4.910	4.969	0.275	0.060
Q96HC4	PDLIM5	-1.022	-1.119	-0.992	-1.185	-1.070	-1.088	0.887	-0.018
Q9UNK9	ANGEL1	-2.531	-2.464	-2.652	-2.634	-2.497	-2.643	0.123	-0.146
Q07960	ARHGAP1	4.166	4.143	4.142	4.104	4.155	4.123	0.316	-0.031
Q9BRA2	TXNDC17	5.204	5.130	5.101	5.098	5.167	5.100	0.321	-0.067
Q53EZ4	CEP55	-1.374	-1.371	-1.715	-1.629	-1.372	-1.672	0.091	-0.299
P53677	AP3M2	-1.041	-0.753	-1.106	-0.871	-0.897	-0.989	0.673	-0.092
Q53H12	AGK	3.876	3.824	3.606	3.604	3.850	3.605	0.068	-0.245
Q6NSJ5	LRRC8E	-0.651	-0.738	-0.768	-0.721	-0.694	-0.744	0.442	-0.050
P78560	CRADD	-1.712	-1.674	-1.730	-1.702	-1.693	-1.716	0.430	-0.024
O00400	SLC33A1	0.605	0.543	0.622	0.651	0.574	0.636	0.263	0.062
Q5U4P2	ASPHD1	-2.178	-2.241	-2.426	-2.235	-2.210	-2.331	0.414	-0.121
Q8NB78	KDM1B	-2.519	-2.606	-2.540	-2.469	-2.563	-2.504	0.409	0.059
Q96RY5	CRAMP1	-3.721	-3.747	-3.612	-3.678	-3.734	-3.645	0.192	0.089
Q53F19	NCBP3	0.000	0.014	0.022	0.047	0.007	0.035	0.222	0.028
Q99988	GDF15	-0.465	-0.485	-0.810	-0.846	-0.475	-0.828	0.009	-0.353
Q8NCH0	CHST14	-2.665	-2.790	-2.752	-2.689	-2.727	-2.720	0.933	0.007
Q6UVJ0	SASS6	-0.730	-0.772	-0.831	-0.801	-0.751	-0.816	0.141	-0.065
O75116	ROCK2	3.861	3.985	3.790	3.802	3.923	3.796	0.286	-0.127
Q99551	MTERF1	-2.207	-2.156	-2.368	-2.237	-2.182	-2.302	0.291	-0.121
Q9BVA1	TUBB2B	-0.587	-0.703	-1.102	-1.256	-0.645	-1.179	0.036	-0.534
O75746	SLC25A12	0.968	0.874	0.907	0.868	0.921	0.887	0.605	-0.033
P23396	RPS3	1.945	1.923	1.898	1.894	1.934	1.896	0.163	-0.038
Q9BVS5	TRMT61B	-0.303	-0.289	-0.374	-0.362	-0.296	-0.368	0.016	-0.072
P11169	SLC2A3	-1.346	-1.902	-0.094	-0.610	-1.624	-0.352	0.079	1.272
Q9H0M5	ZNF700	-3.438	-3.527	-3.136	-3.166	-3.483	-3.151	0.060	0.332

Q9BSR8	YIPF4	-0.269	-0.181	-0.181	-0.057	-0.225	-0.119	0.311	0.106
P35244	RPA3	3.281	3.248	3.025	3.031	3.264	3.028	0.038	-0.236
Q68DK7	MSL1	-2.767	-2.816	-2.757	-2.598	-2.792	-2.677	0.374	0.114
P56385	ATP5ME	1.858	1.834	1.857	1.742	1.846	1.799	0.569	-0.046
P21980	TGM2	6.288	6.172	5.796	5.718	6.230	5.757	0.029	-0.473
P13521	SCG2	-1.635	-1.615	-2.937	-2.690	-1.625	-2.813	0.064	-1.189
Q9UK58	CCNL1	-1.546	-1.386	-1.699	-1.603	-1.466	-1.651	0.214	-0.185
Q99747	NAPG	0.920	0.831	0.909	0.819	0.875	0.864	0.876	-0.011
P05423	POLR3D	0.171	0.224	0.217	0.346	0.197	0.282	0.398	0.084
Q9UHI6	DDX20	2.006	1.928	1.937	1.829	1.967	1.883	0.347	-0.084
P30291	WEE1	-3.010	-3.000	-3.343	-2.541	-3.005	-2.942	0.901	0.063
Q68D20	PMS2CL	-5.542	-5.293	-5.412	-5.655	-5.418	-5.534	0.574	-0.116
P63272	SUPT4H1	1.599	1.525	1.431	1.525	1.562	1.478	0.300	-0.084
P05556	ITGB1	0.960	0.982	1.159	1.085	0.971	1.122	0.129	0.151
Q16595	FXN	0.635	0.703	0.828	0.795	0.669	0.811	0.101	0.143
O15234	CASC3	0.609	0.472	0.493	0.495	0.541	0.494	0.621	-0.046
P62861	FAU	5.343	5.126	5.224	5.145	5.234	5.185	0.728	-0.050
Q9HBH1	PDF	-0.252	-0.353	-0.251	-0.235	-0.302	-0.243	0.447	0.059
Q9Y217	MTMR6	-0.458	-0.603	-0.430	-0.550	-0.530	-0.490	0.712	0.040
Q02318	CYP27A1	-4.038	-3.834	-4.132	-3.978	-3.936	-4.055	0.457	-0.119
Q96RQ3	MCCC1	3.360	3.315	3.364	3.391	3.338	3.377	0.294	0.040
O95295	SNAPIN	-0.766	-0.781	-0.922	-0.902	-0.773	-0.912	0.011	-0.139
Q16850	CYP51A1	-0.352	-0.461	-0.361	-0.431	-0.406	-0.396	0.891	0.010
Q92794	KAT6A	0.721	0.896	0.800	0.881	0.809	0.840	0.786	0.031
Q9BW61	DDA1	0.373	0.325	0.347	0.301	0.349	0.324	0.530	-0.025
Q9UBN7	HDAC6	-0.142	-0.305	-0.227	-0.316	-0.223	-0.272	0.668	-0.048
Q8NHQ9	DDX55	1.756	1.754	1.683	1.736	1.755	1.709	0.333	-0.046
O15258	RER1	2.370	2.358	2.355	2.260	2.364	2.308	0.447	-0.056
Q96IR2	ZNF845	-3.671	-3.842	-3.769	-3.640	-3.757	-3.704	0.675	0.053
Q6P9B9	INTS5	-0.071	-0.097	-0.149	-0.100	-0.084	-0.124	0.321	-0.041
P62491	RAB11A	-0.090	0.127	-0.163	-0.006	0.018	-0.085	0.528	-0.103
P52739	ZNF131	-3.805	-3.714	-3.962	-3.747	-3.760	-3.854	0.535	-0.095
P83881	RPL36A	3.252	2.983	3.330	3.101	3.118	3.215	0.637	0.098
Q96EK9	KTI12	0.082	0.029	0.036	0.062	0.056	0.049	0.849	-0.007
P39019	RPS19	6.937	6.866	6.962	6.898	6.901	6.930	0.608	0.029

Q9UKY1	ZHX1	-2.192	-2.162	-2.298	-2.154	-2.177	-2.226	0.620	-0.049
P01889	HLA	-0.692	-0.757	-0.142	-0.309	-0.725	-0.226	0.073	0.499
O43639	NCK2	-1.633	-1.354	-1.536	-1.506	-1.493	-1.521	0.878	-0.027
P83111	LACTB	0.139	0.097	0.296	0.245	0.118	0.270	0.048	0.152
A2RRP1	NBAS	0.111	0.130	0.075	0.040	0.120	0.057	0.118	-0.063
P18850	ATF6	-1.024	-1.163	-0.951	-1.012	-1.093	-0.982	0.327	0.112
O95070	YIF1A	-1.885	-1.999	-1.834	-1.823	-1.942	-1.829	0.294	0.114
Q9Y2D4	EXOC6B	-0.004	-0.140	-0.049	-0.005	-0.072	-0.027	0.630	0.045
Q9UL18	AGO1	0.363	0.365	0.217	0.322	0.364	0.269	0.324	-0.095
Q96AD5	PNPLA2	-2.159	-2.175	-2.266	-2.193	-2.167	-2.229	0.326	-0.062
P16422	EPCAM	0.435	0.812	0.496	0.530	0.623	0.513	0.662	-0.111
P63165	SUMO1	-0.548	-0.695	-0.394	-0.533	-0.621	-0.464	0.260	0.158
Q9NVX2	NLE1	0.766	0.769	0.725	0.783	0.768	0.754	0.719	-0.014
Q92615	LARP4B	3.239	3.198	3.253	3.218	3.219	3.235	0.600	0.017
Q14978	NOLC1	-1.279	-1.252	-1.213	-1.279	-1.265	-1.246	0.667	0.019
Q9UGP4	LIMD1	0.035	0.025	0.077	0.015	0.030	0.046	0.692	0.016
P27487	DPP4	-3.242	-3.105	-3.195	-3.154	-3.174	-3.174	0.995	-0.001
P07195	LDHB	0.352	0.290	1.847	0.413	0.321	1.130	0.461	0.809
P08581	MET	-3.381	-3.356	-3.196	-3.421	-3.368	-3.309	0.689	0.060
P62310	LSM3	2.025	2.011	1.999	1.987	2.018	1.993	0.121	-0.025
Q5SVZ6	ZMYM1	0.920	0.953	0.980	1.035	0.937	1.007	0.189	0.071
A0AV96	RBM47	-3.014	-3.167	-3.063	-3.071	-3.091	-3.067	0.810	0.024
O00161	SNAP23	0.898	0.929	0.845	0.928	0.914	0.887	0.633	-0.027
Q9NVM9	INTS13	0.074	0.042	-0.011	0.041	0.058	0.015	0.316	-0.043
L0R819	ASDURF	1.266	1.192	1.145	1.166	1.229	1.155	0.280	-0.074
Q9Y6N1	COX11	-1.249	-1.190	-1.176	-1.301	-1.219	-1.239	0.811	-0.020
Q9NSE4	IARS2	5.288	5.275	5.345	5.336	5.281	5.341	0.021	0.059
P54920	NAPA	3.116	3.166	3.028	3.100	3.141	3.064	0.236	-0.077
Q9NYU1	UGGT2	-2.562	-2.485	-2.524	-2.398	-2.524	-2.461	0.502	0.063
Q8TBC4	UBA3	0.056	0.168	0.040	0.182	0.112	0.111	0.994	-0.001
Q13796	SHROOM2	-3.643	-3.636	-3.703	-3.567	-3.640	-3.635	0.961	0.004
P13798	APEH	3.739	3.712	3.695	3.681	3.725	3.688	0.177	-0.037
Q05048	CSTF1	2.395	2.411	2.402	2.400	2.403	2.401	0.866	-0.002
O94923	GLCE	-0.438	-0.416	-0.376	-0.438	-0.427	-0.407	0.629	0.020
P42704	LRPPRC	7.617	7.606	7.575	7.531	7.612	7.553	0.212	-0.059

Q9H069	DRC3	-1.616	-1.636	-1.628	-1.532	-1.626	-1.580	0.512	0.046
O95858	TSPAN15	-2.135	-2.141	-2.251	-2.321	-2.138	-2.286	0.145	-0.148
Q9NRX1	PNO1	2.706	2.710	2.813	2.792	2.708	2.803	0.065	0.094
P52943	CRIP2	-0.869	-1.134	-0.486	-0.796	-1.001	-0.641	0.223	0.360
Q8NI37	PPTC7	-0.997	-1.060	-0.927	-0.747	-1.029	-0.837	0.255	0.192
Q9H7Z7	PTGES2	3.358	3.371	3.318	3.347	3.364	3.332	0.242	-0.032
Q70CQ3	USP30	-1.181	-1.323	-1.253	-1.256	-1.252	-1.254	0.978	-0.002
Q9NW13	RBM28	2.273	2.062	2.226	2.108	2.168	2.167	0.998	0.000
Q9H875	PRKRIP1	-0.237	-0.222	-0.356	-0.335	-0.229	-0.345	0.017	-0.116
Q3ZCW2	LGALSL	-1.406	-1.285	-1.084	-0.989	-1.345	-1.036	0.062	0.309
Q70UQ0	IKBIP	3.130	2.983	3.075	3.052	3.056	3.063	0.939	0.007
Q9BQE5	APOL2	0.651	0.566	0.938	0.873	0.609	0.905	0.036	0.297
P20933	AGA	-2.252	-2.096	-2.211	-2.145	-2.174	-2.178	0.964	-0.005
Q8WUX9	CHMP7	-2.063	-2.238	-2.091	-2.190	-2.151	-2.140	0.928	0.011
Q4VC31	MIX23	2.412	2.348	2.449	2.433	2.380	2.441	0.294	0.061
O95865	DDAH2	2.692	2.588	2.850	2.808	2.640	2.829	0.134	0.189
Q9NP92	MRPS30	2.375	2.447	2.258	2.325	2.411	2.291	0.136	-0.119
O43615	TIMM44	4.487	4.377	4.532	4.482	4.432	4.507	0.385	0.075
Q14241	ELOA	2.335	2.317	2.224	2.231	2.326	2.227	0.029	-0.099
Q8IXS8	FAM126B	-1.682	-1.795	-1.681	-1.622	-1.739	-1.652	0.342	0.087
Q92754	TFAP2C	-5.964	-5.642	-6.064	-5.841	-5.803	-5.953	0.532	-0.150
P43034	PAFAH1B1	4.279	4.255	4.256	4.257	4.267	4.256	0.543	-0.011
O95260	ATE1	-1.433	-1.545	-1.599	-1.617	-1.489	-1.608	0.272	-0.119
Q13206	DDX10	3.047	2.922	2.922	2.946	2.984	2.934	0.567	-0.050
O00214	LGALS8	-3.374	-3.409	-3.392	-3.316	-3.392	-3.354	0.499	0.038
Q9H2C2	ARV1	-1.581	-1.528	-1.565	-1.575	-1.554	-1.570	0.662	-0.015
Q6ZSS7	MFSD6	-3.376	-3.190	-3.456	-2.985	-3.283	-3.220	0.837	0.063
Q9Y4C4	MFHAS1	-3.825	-3.791	-3.950	-3.857	-3.808	-3.903	0.260	-0.095
P28288	ABCD3	0.967	1.004	0.888	0.870	0.986	0.879	0.065	-0.106
Q96GX9	APIP	-0.094	-0.203	0.067	0.014	-0.149	0.040	0.133	0.189
O75828	CBR3	3.190	3.080	2.793	2.822	3.135	2.807	0.088	-0.328
P12235	SLC25A4	1.920	1.914	2.048	1.970	1.917	2.009	0.251	0.092
O75489	NDUFS3	3.301	3.292	3.331	3.350	3.297	3.340	0.088	0.044
P57678	GEMIN4	2.717	2.737	2.542	2.614	2.727	2.578	0.132	-0.149
Q9UJY4	GGA2	1.055	0.993	0.955	0.958	1.024	0.956	0.270	-0.068

Q9H496	TOR1AIP2	-2.770	-2.926	-2.773	-3.005	-2.848	-2.889	0.799	-0.041
Q9NZC9	SMARCAL1	-0.075	-0.170	-0.161	0.006	-0.122	-0.078	0.697	0.045
Q9UGP8	SEC63	2.904	2.915	2.963	2.936	2.910	2.949	0.169	0.040
Q00403	GTF2B	1.253	1.213	1.300	1.245	1.233	1.273	0.374	0.039
Q15654	TRIP6	2.563	2.445	2.495	2.417	2.504	2.456	0.574	-0.048
Q9H939	PSTPIP2	-2.227	-2.351	-2.068	-2.156	-2.289	-2.112	0.158	0.178
O00458	IFRD1	-7.149	-7.473	-7.351	-6.988	-7.311	-7.169	0.620	0.142
Q9H329	EPB41L4B	-3.642	-3.654	-3.667	-3.810	-3.648	-3.739	0.424	-0.090
Q9NQE9	HINT3	-1.261	-1.132	-1.007	-0.797	-1.197	-0.902	0.166	0.295
Q8NI77	KIF18A	-0.761	-0.795	-1.010	-0.970	-0.778	-0.990	0.016	-0.212
Q92817	EVPL	2.275	2.638	2.409	2.428	2.457	2.419	0.869	-0.038
P45877	PPIC	-1.107	-0.954	-1.385	-1.350	-1.031	-1.367	0.127	-0.336
Q8IYA6	CKAP2L	-3.336	-3.417	-3.468	-3.592	-3.376	-3.530	0.193	-0.154
Q96IY1	NSL1	-1.562	-1.747	-1.528	-1.597	-1.655	-1.563	0.493	0.092
Q92536	SLC7A6	-1.196	-1.191	-1.460	-1.355	-1.193	-1.408	0.152	-0.214
Q9BTL4	IER2	-2.485	-2.550	-2.528	-2.507	-2.517	-2.517	0.997	0.000
Q92696	RABGGTA	2.125	2.112	2.080	2.087	2.118	2.083	0.061	-0.035
Q92544	TM9SF4	3.066	3.098	3.040	3.039	3.082	3.039	0.233	-0.043
A6NFY7	SDHAF1	-3.954	-3.930	-3.847	-3.978	-3.942	-3.913	0.729	0.030
Q96J01	THOC3	-0.691	-0.780	-0.854	-0.941	-0.736	-0.898	0.122	-0.162
O00391	QSOX1	-2.415	-2.686	-2.217	-2.299	-2.550	-2.258	0.255	0.293
P09661	SNRPA1	4.348	4.293	4.368	4.349	4.320	4.359	0.383	0.038
Q9UIV1	CNOT7	-2.466	-2.388	-2.625	-2.574	-2.427	-2.600	0.081	-0.172
Q9NRC8	SIRT7	-3.164	-3.232	-3.143	-3.174	-3.198	-3.159	0.437	0.039
Q9BXF6	RAB11FIP5	0.781	0.697	0.848	0.829	0.739	0.839	0.240	0.099
O95639	CPSF4	-3.113	-3.470	-3.382	-3.240	-3.292	-3.311	0.934	-0.019
Q9P2B2	PTGFRN	0.229	0.275	0.444	0.329	0.252	0.387	0.225	0.135
Q9BT17	MTG1	-0.663	-0.650	-0.784	-0.722	-0.656	-0.753	0.185	-0.097
Q9BXC9	BBS2	-1.921	-2.133	-1.914	-1.908	-2.027	-1.911	0.471	0.116
P47974	ZFP36L2	-4.594	-4.467	-4.628	-4.544	-4.530	-4.586	0.548	-0.056
O00541	PES1	-2.701	-2.831	-2.736	-2.826	-2.766	-2.781	0.867	-0.015
Q9Y512	SAMM50	4.175	4.189	4.186	4.207	4.182	4.197	0.387	0.015
Q00688	FKBP3	5.157	5.131	5.005	5.000	5.144	5.002	0.049	-0.142
Q96EK4	THAP11	-1.927	-1.849	-2.032	-2.125	-1.888	-2.079	0.091	-0.191
Q9HC52	CBX8	1.124	1.197	1.098	1.122	1.160	1.110	0.385	-0.051

Q15738	NSDHL	3.696	3.716	3.749	3.721	3.706	3.735	0.239	0.029
P78330	PSPH	2.474	2.376	2.427	2.371	2.425	2.399	0.700	-0.026
P16949	STMN1	2.974	2.788	2.644	2.603	2.881	2.624	0.208	-0.257
P28340	POLD1	3.553	3.543	3.395	3.354	3.548	3.375	0.061	-0.173
Q9Y399	MRPS2	2.374	2.386	2.332	2.329	2.380	2.331	0.067	-0.049
Q9BZF9	UACA	-3.471	-3.683	-3.776	-3.772	-3.577	-3.774	0.315	-0.197
O95139	NDUFB6	0.261	0.349	0.216	0.256	0.305	0.236	0.335	-0.069
Q6ZNE5	ATG14	-2.881	-2.811	-2.891	-2.890	-2.846	-2.891	0.423	-0.045
Q9BYD1	MRPL13	2.937	2.937	2.878	2.922	2.937	2.900	0.339	-0.037
Q9BYI3	FAM126A	-5.351	-5.238	-5.466	-5.404	-5.295	-5.435	0.195	-0.140
Q14012	CAMK1	-0.893	-1.099	-0.907	-1.002	-0.996	-0.955	0.762	0.041
O00584	RNASET2	-0.826	-0.892	-0.660	-0.733	-0.859	-0.697	0.082	0.162
Q14195	DPYSL3	-4.099	-3.808	-3.926	-3.883	-3.954	-3.905	0.793	0.049
Q29RF7	PDS5A	3.263	3.277	3.316	3.284	3.270	3.300	0.287	0.030
Q96FV2	SCRN2	-1.631	-1.619	-1.794	-1.948	-1.625	-1.871	0.191	-0.246
Q9Y5L4	TIMM13	2.695	2.620	2.649	2.628	2.657	2.638	0.706	-0.019
P04792	HSPB1	7.488	7.401	7.214	7.285	7.445	7.249	0.079	-0.195
Q96NB3	ZNF830	1.249	1.202	1.213	1.205	1.226	1.209	0.604	-0.017
Q96RU7	TRIB3	-1.317	-1.205	-1.481	-1.401	-1.261	-1.441	0.134	-0.180
Q969V6	MRTFA	-1.306	-1.400	-1.423	-1.391	-1.353	-1.407	0.443	-0.054
P17844	DDX5	1.107	1.271	1.067	1.161	1.189	1.114	0.528	-0.075
O43395	PRPF3	3.727	3.683	3.738	3.686	3.705	3.712	0.865	0.007
Q9BUH6	PAXX	0.478	0.812	0.547	0.696	0.645	0.622	0.914	-0.023
O95149	SNUPN	1.097	1.050	0.932	0.975	1.074	0.954	0.064	-0.120
Q8N2H3	PYROXD2	-3.108	-2.989	-3.059	-3.017	-3.049	-3.038	0.886	0.011
Q8WV24	PHLDA1	1.706	1.760	1.629	1.666	1.733	1.647	0.136	-0.085
Q14527	HLTF	-0.217	-0.082	-0.328	-0.282	-0.150	-0.305	0.235	-0.155
Q9H6E5	TUT1	-0.339	-0.285	-0.224	-0.146	-0.312	-0.185	0.131	0.127
P07741	APRT	-4.381	-4.062	-3.864	-4.377	-4.221	-4.120	0.775	0.101
Q15543	TAF13	-3.564	-3.542	-3.458	-3.431	-3.553	-3.444	0.029	0.108
Q8IWV7	UBR1	0.415	0.540	0.363	0.418	0.477	0.390	0.376	-0.087
P61964	WDR5	3.453	3.417	3.430	3.445	3.435	3.438	0.900	0.003
Q12907	LMAN2	4.453	4.350	4.352	4.293	4.402	4.322	0.341	-0.079
Q96FV9	THOC1	2.167	2.073	2.138	2.154	2.120	2.146	0.685	0.025
Q9HCM4	EPB41L5	-2.245	-2.239	-2.361	-2.443	-2.242	-2.402	0.158	-0.160

Q86UL3	GPAT4	0.417	0.394	0.496	0.427	0.406	0.462	0.332	0.056
O60347	TBC1D12	-2.845	-2.842	-2.870	-2.756	-2.844	-2.813	0.683	0.031
Q68D91	MBLAC2	-0.314	-0.384	-0.276	-0.294	-0.349	-0.285	0.305	0.064
Q14331	FRG1	1.561	1.439	1.583	1.498	1.500	1.541	0.643	0.041
Q9H7N4	SCAF1	2.459	2.399	2.566	2.480	2.429	2.523	0.227	0.094
Q9H583	HEATR1	4.935	4.899	4.937	4.918	4.917	4.928	0.677	0.010
Q53ET0	CRTC2	-0.621	-0.656	-0.656	-0.530	-0.638	-0.593	0.600	0.046
Q96IQ9	ZNF414	-6.157	-5.924	-6.014	-5.824	-6.040	-5.919	0.507	0.121
Q9UD71	PPP1R1B	-3.025	-2.919	-3.017	-2.698	-2.972	-2.857	0.602	0.114
P51570	GALK1	3.131	2.961	3.135	3.054	3.046	3.095	0.674	0.048
Q6IA69	NADSYN1	-0.762	-0.835	-0.919	-0.825	-0.798	-0.872	0.348	-0.074
Q86XL3	ANKLE2	-2.028	-2.090	-2.246	-2.126	-2.059	-2.186	0.243	-0.127
Q15293	RCN1	0.033	0.072	0.155	0.227	0.053	0.191	0.110	0.139
Q9ULW3	ABT1	2.242	2.161	2.129	2.185	2.202	2.157	0.467	-0.045
Q9Y5X2	SNX8	2.357	2.297	2.279	2.209	2.327	2.244	0.216	-0.083
O60885	BRD4	0.492	0.484	0.351	0.360	0.488	0.355	0.002	-0.133
Q16851	UGP2	-1.083	-1.087	-0.816	-0.903	-1.085	-0.860	0.120	0.225
P05362	ICAM1	-2.347	-2.224	-2.032	-2.289	-2.286	-2.160	0.501	0.125
O14672	ADAM10	0.813	0.740	0.829	0.849	0.776	0.839	0.320	0.063
P33176	KIF5B	5.202	5.131	5.130	5.050	5.166	5.090	0.290	-0.076
P42677	RPS27	0.746	0.748	0.771	0.732	0.747	0.752	0.848	0.005
P49716	CEBPD	-4.677	-4.831	-3.723	-3.505	-4.754	-3.614	0.018	1.140
Q96MX3	ZNF48	-0.851	-0.892	-0.371	-0.532	-0.871	-0.452	0.103	0.420
P24534	EEF1B2	6.884	6.876	6.868	6.850	6.880	6.859	0.219	-0.022
P24752	ACAT1	5.607	5.428	5.765	5.733	5.517	5.749	0.225	0.232
Q14019	COTL1	5.203	5.210	5.206	5.158	5.207	5.182	0.492	-0.025
Q15024	EXOSC7	2.472	2.349	2.373	2.354	2.411	2.364	0.586	-0.047
Q9H9F9	ACTR5	0.505	0.472	0.488	0.441	0.489	0.465	0.498	-0.024
Q96HS1	PGAM5	0.988	0.871	0.997	0.972	0.929	0.984	0.515	0.055
P52799	EFNB2	-4.409	-3.954	-4.390	-3.985	-4.182	-4.187	0.987	-0.006
Q8N3F8	MICALL1	1.638	1.568	1.726	1.701	1.603	1.713	0.164	0.110
O60884	DNAJA2	4.304	4.244	4.247	4.246	4.274	4.247	0.532	-0.027
P11802	CDK4	-0.365	-0.351	-0.363	-0.363	-0.358	-0.363	0.612	-0.005
P69905	HBA1	-2.351	-2.264	-1.886	-1.951	-2.307	-1.919	0.024	0.389
Q04760	GLO1	-0.043	-0.161	-0.089	0.083	-0.102	-0.003	0.457	0.098

Q56VL3	OCIAD2	0.149	0.298	0.236	0.299	0.224	0.268	0.662	0.044
P13682	ZNF35	-4.409	-4.421	-4.578	-4.323	-4.415	-4.450	0.828	-0.035
O75821	EIF3G	3.971	3.944	3.837	3.852	3.958	3.844	0.034	-0.113
Q9NV56	MRGBP	0.054	0.005	0.122	0.063	0.030	0.093	0.243	0.063
Q96DF8	ESS2	-0.285	-0.312	-0.369	-0.361	-0.298	-0.365	0.110	-0.067
Q9BVA0	KATNB1	1.116	1.073	1.044	1.107	1.095	1.076	0.671	-0.019
Q7L523	RRAGA	-1.738	-1.918	-1.485	-1.798	-1.828	-1.641	0.432	0.187
Q9NWQ8	PAG1	-2.992	-2.665	-2.752	-2.687	-2.829	-2.720	0.626	0.109
P49189	ALDH9A1	-1.770	-1.850	-1.669	-1.787	-1.810	-1.728	0.383	0.082
Q96SZ5	ADO	1.242	1.268	1.258	1.222	1.255	1.240	0.582	-0.015
Q9HBH0	RHOF	-3.721	-3.311	-3.548	-3.411	-3.516	-3.480	0.890	0.036
O95347	SMC2	-0.599	-0.466	-0.739	-0.612	-0.533	-0.676	0.261	-0.143
Q9H3E2	SNX25	-2.382	-2.367	-2.361	-2.300	-2.374	-2.331	0.374	0.044
Q8N6M0	OTUD6B	-1.383	-1.447	-1.447	-1.400	-1.415	-1.424	0.855	-0.008
P16104	H2AX	2.214	2.179	2.342	2.241	2.197	2.291	0.288	0.095
Q14258	TRIM25	4.394	4.308	4.444	4.369	4.351	4.406	0.436	0.055
Q69YN2	CWF19L1	-1.271	-1.368	-1.310	-1.437	-1.319	-1.373	0.575	-0.054
P18084	ITGB5	0.584	0.619	0.724	0.729	0.602	0.727	0.081	0.125
O60674	JAK2	-2.311	-2.191	-2.276	-2.094	-2.251	-2.185	0.617	0.065
Q6AI12	ANKRD40	-0.933	-0.928	-0.893	-0.760	-0.931	-0.826	0.363	0.104
Q9NWY4	HPF1	-0.175	-0.212	-0.297	-0.255	-0.194	-0.276	0.101	-0.082
A6NKF1	SAC3D1	-1.872	-1.847	-1.872	-1.808	-1.859	-1.840	0.656	0.019
Q9Y5B9	SUPT16H	6.333	6.320	6.274	6.227	6.327	6.250	0.168	-0.076
Q14249	ENDOG	2.243	2.077	2.158	2.145	2.160	2.151	0.934	-0.009
O95817	BAG3	2.527	2.467	2.348	2.420	2.497	2.384	0.141	-0.113
Q5J8M3	EMC4	-3.289	-3.295	-3.327	-3.464	-3.292	-3.396	0.372	-0.104
Q92508	PIEZO1	0.276	0.175	0.202	0.241	0.225	0.222	0.956	-0.004
P21926	CD9	4.126	4.033	4.103	4.036	4.080	4.069	0.875	-0.010
P60903	S100A10	4.365	4.432	4.422	4.302	4.398	4.362	0.664	-0.036
Q9NZE8	MRPL35	0.167	0.129	0.093	0.154	0.148	0.123	0.579	-0.025
Q86U38	NOP9	0.091	0.325	0.090	0.193	0.208	0.142	0.676	-0.066
Q9NPH0	ACP6	-0.739	-0.584	-0.662	-0.517	-0.661	-0.589	0.568	0.072
Q9P2C4	TMEM181	-0.537	-0.599	-0.543	-0.577	-0.568	-0.560	0.839	0.008
Q06136	KDSR	-0.712	-0.767	-0.683	-0.706	-0.739	-0.694	0.326	0.045
Q6PII3	CCDC174	0.244	0.231	0.346	0.235	0.238	0.290	0.516	0.052

Q9H0H3	KLHL25	-3.525	-3.396	-3.681	-3.408	-3.460	-3.545	0.652	-0.084
Q8NBU5	ATAD1	-1.219	-1.203	-1.088	-1.107	-1.211	-1.097	0.013	0.113
O00178	GTPBP1	2.443	2.367	2.356	2.386	2.405	2.371	0.533	-0.034
Q8WVK2	SNRNP27	1.395	1.295	1.222	1.340	1.345	1.281	0.501	-0.064
Q9UJX4	ANAPC5	-1.244	-1.267	-1.453	-1.353	-1.256	-1.403	0.192	-0.147
P61254	RPL26	0.712	0.675	0.804	0.670	0.694	0.737	0.634	0.043
Q15021	NCAPD2	4.212	4.281	4.012	4.046	4.246	4.029	0.059	-0.217
Q15836	VAMP3	-0.902	-0.847	-1.035	-1.002	-0.875	-1.019	0.066	-0.144
Q9HA64	FN3KRP	1.736	1.747	1.658	1.721	1.742	1.689	0.339	-0.052
P07199	CENPB	1.025	1.080	0.996	1.029	1.053	1.012	0.361	-0.040
Q7L2Z9	CENPQ	-1.751	-1.703	-1.722	-1.747	-1.727	-1.735	0.805	-0.008
Q7Z4F1	LRP10	-3.683	-3.818	-3.191	-3.267	-3.750	-3.229	0.038	0.521
Q9Y4K0	LOXL2	-0.914	-1.004	-1.161	-1.181	-0.959	-1.171	0.120	-0.212
Q13405	MRPL49	2.159	1.932	1.956	1.930	2.045	1.943	0.533	-0.102
Q9H3Y8	PPDPF	-5.359	-5.314	-5.421	-5.385	-5.336	-5.403	0.152	-0.066
Q13895	BYSL	3.324	3.718	3.548	3.582	3.521	3.565	0.860	0.044
Q9BT73	PSMG3	1.975	1.939	1.782	1.866	1.957	1.824	0.155	-0.133
Q9Y3B2	EXOSC1	1.484	1.512	1.482	1.490	1.498	1.486	0.545	-0.012
Q9Y6V7	DDX49	0.826	0.786	0.728	0.747	0.806	0.738	0.134	-0.068
P19525	EIF2AK2	2.141	2.141	2.007	2.040	2.141	2.023	0.089	-0.118
Q13136	PPFIA1	-1.691	-1.673	-1.745	-1.694	-1.682	-1.720	0.367	-0.038
Q9HD15	SRA1	2.364	2.208	2.235	2.179	2.286	2.207	0.486	-0.079
Q6P0N0	MIS18BP1	-0.807	-0.776	-0.832	-0.939	-0.791	-0.885	0.316	-0.094
P56556	NDUFA6	1.602	1.674	1.721	1.746	1.638	1.733	0.204	0.095
Q9UK45	LSM7	3.726	3.509	3.565	3.576	3.617	3.571	0.743	-0.046
P48553	TRAPPC10	0.321	0.253	0.236	0.292	0.287	0.264	0.660	-0.023
Q15834	CCDC85B	-1.886	-1.965	-1.971	-2.076	-1.925	-2.023	0.284	-0.098
Q9UBW7	ZMYM2	1.783	1.893	1.755	1.796	1.838	1.775	0.449	-0.063
Q9P2E9	RRBP1	3.557	3.678	3.778	3.703	3.618	3.740	0.253	0.123
P08708	RPS17	4.043	3.859	4.051	3.861	3.951	3.956	0.976	0.005
Q99611	SEPHS2	-1.383	-1.468	-1.243	-1.172	-1.426	-1.208	0.062	0.218
Q5T0Z8	C6orf132	1.988	2.346	2.094	2.154	2.167	2.124	0.850	-0.043
Q9H0C5	BTBD1	-3.509	-3.538	-3.234	-3.347	-3.524	-3.291	0.134	0.233
Q8N6H7	ARFGAP2	-2.360	-2.632	-2.387	-2.687	-2.496	-2.537	0.859	-0.041
Q10469	MGAT2	0.631	0.624	0.477	0.537	0.628	0.507	0.150	-0.121

Q9BQD7	ANTKMT	-2.757	-2.608	-2.667	-2.588	-2.683	-2.627	0.597	0.055
Q14435	GALNT3	-1.440	-0.780	-0.859	-1.049	-1.110	-0.954	0.719	0.156
Q9UHE8	STEAP1	-3.267	-2.979	-3.090	-3.165	-3.123	-3.128	0.980	-0.004
Q9UMX3	BOK	-3.000	-2.768	-3.044	-2.683	-2.884	-2.863	0.934	0.021
P60468	SEC61B	2.404	2.377	2.242	2.312	2.391	2.277	0.155	-0.113
Q13162	PRDX4	4.787	4.717	4.721	4.668	4.752	4.694	0.332	-0.058
Q9NP84	TNFRSF12A	-1.955	-1.938	-2.415	-2.295	-1.947	-2.355	0.088	-0.408
Q6PGP7	TTC37	1.319	1.468	1.402	1.503	1.393	1.453	0.585	0.059
P32969	RPL9	4.881	4.784	4.817	4.803	4.833	4.810	0.723	-0.023
Q96ST3	SIN3A	3.414	3.480	3.463	3.466	3.447	3.465	0.689	0.018
Q9Y3B9	RRP15	1.918	1.946	1.939	1.928	1.932	1.934	0.934	0.001
P20930	FLG	-2.624	-2.618	-2.764	-2.618	-2.621	-2.691	0.513	-0.070
Q96CN7	ISOC1	1.887	1.978	1.907	1.942	1.933	1.925	0.891	-0.008
P61006	RAB8A	1.024	1.063	0.881	0.900	1.043	0.890	0.041	-0.153
Q8IZ41	RASEF	-2.573	-2.576	-2.266	-2.335	-2.574	-2.300	0.080	0.274
P05204	HMGN2	5.447	5.403	5.140	5.016	5.425	5.078	0.084	-0.347
Q8IZQ5	SELENOH	1.785	1.786	1.824	1.872	1.785	1.848	0.235	0.063
P13196	ALAS1	0.048	0.129	0.175	0.187	0.088	0.181	0.259	0.093
Q9Y2U5	MAP3K2	-0.193	-0.356	-0.110	-0.165	-0.274	-0.137	0.322	0.137
O94782	USP1	-1.878	-1.844	-1.797	-1.808	-1.861	-1.802	0.151	0.059
Q9BUR5	APOO	-1.500	-1.477	-1.449	-1.384	-1.488	-1.416	0.245	0.072
Q96E39	RBMXL1	0.740	0.660	0.897	0.935	0.700	0.916	0.074	0.216
P14621	ACYP2	-1.148	-1.056	-0.876	-0.947	-1.102	-0.911	0.088	0.191
P63218	GNG5	0.413	0.437	0.325	0.455	0.425	0.390	0.684	-0.035
Q96FF7	MISP3	-3.777	-3.326	-3.543	-3.587	-3.551	-3.565	0.960	-0.014
A0JNW5	UHRF1BP1L	-0.530	-0.632	-0.708	-0.647	-0.581	-0.677	0.273	-0.097
Q96NL8	CFAP418	-3.172	-3.095	-3.236	-3.127	-3.133	-3.181	0.555	-0.048
Q16658	FSCN1	3.007	3.206	3.429	3.325	3.107	3.377	0.177	0.271
Q9UDY4	DNAJB4	-0.455	-0.406	-0.479	-0.368	-0.431	-0.423	0.915	0.008
Q9UNF1	MAGED2	0.128	0.269	0.346	0.468	0.199	0.407	0.159	0.208
P30711	GSTT1	-0.860	-0.706	-0.739	-0.862	-0.783	-0.801	0.874	-0.018
P00568	AK1	4.056	4.167	3.894	3.984	4.111	3.939	0.144	-0.172
Q9UNF0	PACSIN2	-1.679	-1.517	-1.665	-1.599	-1.598	-1.632	0.750	-0.034
P56524	HDAC4	-3.174	-3.128	-3.066	-3.112	-3.151	-3.089	0.197	0.062
P62834	RAP1A	0.700	0.769	0.806	0.824	0.734	0.815	0.239	0.081

Q8IZL8	PELP1	3.761	3.686	3.814	3.825	3.723	3.820	0.232	0.096
Q86YV5	PRAG1	0.763	0.821	0.962	0.977	0.792	0.969	0.089	0.177
A6NEL2	SOWAHB	-5.302	-5.363	-5.263	-5.219	-5.333	-5.241	0.147	0.091
Q9Y584	TIMM22	-1.389	-1.410	-1.444	-1.491	-1.399	-1.467	0.168	-0.068
Q9UNI6	DUSP12	-0.040	-0.006	-0.041	-0.009	-0.023	-0.025	0.932	-0.002
Q9H1Z4	WDR13	-1.138	-1.160	-1.167	-1.074	-1.149	-1.121	0.649	0.028
Q9Y5K6	CD2AP	3.797	3.887	3.936	3.809	3.842	3.873	0.735	0.031
Q99757	TXN2	-0.212	-0.393	-0.341	-0.355	-0.303	-0.348	0.706	-0.045
Q9UNN8	PROCR	-0.266	-0.388	-0.272	-0.319	-0.327	-0.295	0.699	0.031
O15357	INPPL1	0.250	0.153	0.189	0.084	0.201	0.137	0.463	-0.065
P14406	COX7A2	1.738	1.845	1.724	1.802	1.791	1.763	0.716	-0.028
P12429	ANXA3	1.067	2.000	1.429	1.110	1.534	1.270	0.673	-0.264
P15880	RPS2	6.737	6.679	6.730	6.653	6.708	6.691	0.768	-0.016
Q9Y4C8	RBM19	2.566	2.547	2.545	2.559	2.556	2.552	0.765	-0.004
Q9Y2G8	DNAJC16	-1.030	-1.018	-1.069	-1.070	-1.024	-1.070	0.079	-0.046
Q7Z794	KRT77	-2.335	-2.255	-2.498	-2.324	-2.295	-2.411	0.391	-0.116
Q9UM47	NOTCH3	-2.830	-2.799	-2.741	-2.679	-2.815	-2.710	0.136	0.104
Q15434	RBMS2	0.162	0.255	0.193	0.043	0.208	0.118	0.431	-0.090
Q8TBF4	ZCRB1	-1.055	-1.107	-1.010	-1.065	-1.081	-1.038	0.375	0.043
Q14185	DOCK1	2.335	2.272	2.339	2.317	2.304	2.328	0.583	0.024
Q8NBM8	PCYOX1L	0.497	0.515	0.374	0.505	0.506	0.440	0.496	-0.066
Q7Z3E2	CCDC186	-0.422	-0.412	-0.461	-0.381	-0.417	-0.421	0.927	-0.005
Q6UB28	METAP1D	-2.989	-2.962	-2.852	-2.933	-2.976	-2.892	0.266	0.083
Q8WXW3	PIBF1	-4.257	-4.232	-4.441	-4.229	-4.244	-4.335	0.548	-0.091
Q92521	PIGB	-3.378	-3.388	-3.334	-3.240	-3.383	-3.287	0.285	0.096
P57088	TMEM33	2.317	2.368	2.356	2.380	2.343	2.368	0.498	0.025
Q05639	EEF1A2	4.598	4.637	4.613	4.570	4.618	4.592	0.468	-0.026
Q9BZM1	PLA2G12A	-2.454	-2.566	-2.553	-2.604	-2.510	-2.579	0.418	-0.069
C9JI98	TMEM238	-3.526	-3.391	-3.235	-3.167	-3.459	-3.201	0.114	0.257
P13994	YJU2B	-3.571	-3.544	-3.571	-3.540	-3.558	-3.556	0.939	0.002
Q9BVJ7	DUSP23	-1.682	-1.164	-1.477	-0.910	-1.423	-1.194	0.612	0.229
Q9UBE0	SAE1	1.886	1.857	1.889	1.938	1.872	1.914	0.302	0.042
P67809	YBX1	4.234	4.184	4.093	4.047	4.209	4.070	0.054	-0.139
Q9H832	UBE2Z	1.415	1.336	1.470	1.284	1.376	1.377	0.994	0.001
Q8WV19	SFT2D1	-4.981	-4.945	-5.095	-4.758	-4.963	-4.927	0.863	0.037

Q9NWU2	GID8	-0.773	-0.681	-0.892	-0.935	-0.727	-0.914	0.110	-0.186
Q9UJ72	ANXA10	-2.686	-2.681	-2.381	-2.656	-2.683	-2.518	0.443	0.165
O14980	XPO1	6.430	6.405	6.308	6.287	6.418	6.297	0.021	-0.120
P62314	SNRPD1	0.813	0.895	0.680	0.773	0.854	0.727	0.179	-0.128
O95749	GGPS1	0.348	0.203	0.317	0.453	0.275	0.385	0.384	0.110
Q96ER3	SAAL1	0.870	0.810	0.705	0.704	0.840	0.705	0.140	-0.135
Q2YD98	UVSSA	-4.608	-4.611	-4.644	-4.544	-4.609	-4.594	0.809	0.015
Q7Z4W1	DCXR	3.018	3.053	3.132	3.105	3.036	3.119	0.071	0.083
Q9Y5U9	IER3IP1	-0.257	-0.417	-0.355	-0.256	-0.337	-0.306	0.776	0.031
Q96FK6	WDR89	-0.243	-0.247	-0.134	-0.192	-0.245	-0.163	0.216	0.082
Q96T58	SPEN	2.309	2.289	2.285	2.334	2.299	2.310	0.741	0.011
Q9BXR0	QTRT1	2.076	1.948	2.112	2.072	2.012	2.092	0.418	0.080
Q02156	PRKCE	-2.485	-2.501	-2.390	-2.343	-2.493	-2.367	0.089	0.126
Q9Y5K8	ATP6V1D	1.762	1.726	1.663	1.635	1.744	1.649	0.058	-0.096
P16219	ACADS	3.006	3.064	3.220	3.277	3.035	3.248	0.034	0.213
Q6BCY4	CYB5R2	-2.740	-2.631	-2.787	-2.631	-2.685	-2.709	0.828	-0.024
Q96BZ8	LENG1	-1.108	-1.099	-1.169	-1.087	-1.104	-1.128	0.661	-0.024
Q969Q5	RAB24	0.060	0.013	0.042	0.067	0.037	0.055	0.588	0.018
Q15058	KIF14	1.381	1.283	1.278	1.277	1.332	1.277	0.464	-0.055
Q5JSL3	DOCK11	4.887	4.779	4.738	4.734	4.833	4.736	0.322	-0.097
Q15170	TCEAL1	0.002	0.026	0.090	0.043	0.014	0.067	0.227	0.053
Q9UBV2	SEL1L	0.299	0.360	0.419	0.469	0.329	0.444	0.105	0.115
Q02338	BDH1	2.532	2.414	2.483	2.443	2.473	2.463	0.894	-0.010
P12270	TPR	5.413	5.353	5.452	5.433	5.383	5.443	0.279	0.060
Q01970	PLCB3	-0.187	-0.136	-0.353	-0.250	-0.162	-0.301	0.179	-0.139
Q9HBR0	SLC38A10	-0.932	-0.920	-0.967	-0.894	-0.926	-0.931	0.921	-0.005
O15516	CLOCK	-1.850	-1.793	-1.998	-1.888	-1.821	-1.943	0.228	-0.122
Q8N0V3	RBFA	-2.283	-2.169	-2.569	-2.271	-2.226	-2.420	0.400	-0.195
Q9HBL8	NMRAL1	1.553	1.465	1.393	1.432	1.509	1.413	0.238	-0.096
P38936	CDKN1A	-3.705	-3.770	-3.909	-3.893	-3.738	-3.901	0.109	-0.164
Q9UGR2	ZC3H7B	1.516	1.524	1.499	1.578	1.520	1.539	0.714	0.019
Q9H3Q1	CDC42EP4	0.279	0.454	0.473	0.412	0.366	0.443	0.537	0.076
Q8TCT8	SPPL2A	-1.165	-0.897	-0.924	-0.690	-1.031	-0.807	0.336	0.224
P06746	POLB	1.660	1.838	1.808	1.865	1.749	1.837	0.498	0.088
Q9Y6G9	DYNC1LI1	3.782	3.739	3.769	3.741	3.760	3.755	0.858	-0.005

O00203	AP3B1	-0.074	-0.189	-0.080	-0.175	-0.132	-0.127	0.959	0.004
Q9H3F6	KCTD10	-2.956	-2.969	-2.992	-3.055	-2.962	-3.023	0.294	-0.061
Q96DU7	ITPKC	-1.321	-1.342	-1.303	-1.191	-1.331	-1.247	0.368	0.084
P55072	VCP	6.499	6.513	6.495	6.502	6.506	6.498	0.479	-0.008
Q53T59	HS1BP3	1.470	1.335	1.414	1.321	1.402	1.368	0.717	-0.035
Q9NUG6	PDRG1	1.126	1.125	1.125	1.037	1.125	1.081	0.494	-0.045
Q96CS2	HAUS1	-4.530	-4.412	-4.273	-4.111	-4.471	-4.192	0.120	0.279
O94913	PCF11	2.022	1.992	1.977	2.043	2.007	2.010	0.940	0.003
Q9NQ92	COPRS	-3.028	-2.979	-3.213	-3.044	-3.004	-3.129	0.364	-0.125
Q6Y1H2	HACD2	0.122	0.097	0.276	0.286	0.109	0.281	0.025	0.171
Q8WUA2	PPIL4	2.311	2.342	2.410	2.357	2.326	2.384	0.234	0.057
P02545	LMNA	-4.402	-4.416	-4.559	-4.676	-4.409	-4.617	0.171	-0.209
P13639	EEF2	8.593	8.492	8.579	8.550	8.542	8.565	0.739	0.022
Q9NX62	BPNT2	2.119	2.054	2.131	2.154	2.086	2.142	0.310	0.056
Q9BRG1	VPS25	2.344	2.370	2.338	2.312	2.357	2.325	0.215	-0.032
O94811	TPPP	-3.099	-3.165	-2.786	-2.774	-3.132	-2.780	0.053	0.352
Q6P6B1	ERICH5	-2.256	-2.196	-2.455	-2.238	-2.226	-2.347	0.460	-0.121
O75460	ERN1	-2.185	-2.309	-1.914	-1.953	-2.247	-1.933	0.098	0.314
P62166	NCS1	-1.405	-1.367	-1.433	-1.377	-1.386	-1.405	0.643	-0.019
O43765	SGTA	4.009	3.888	3.832	3.773	3.949	3.802	0.210	-0.146
Q96P70	IPO9	4.496	4.460	4.269	4.247	4.478	4.258	0.017	-0.220
Q9NWS6	FAM118A	-1.684	-1.437	-1.499	-1.334	-1.560	-1.416	0.447	0.144
P21796	VDAC1	6.490	6.394	6.496	6.449	6.442	6.473	0.640	0.031
O75879	GATB	1.448	1.480	1.291	1.332	1.464	1.311	0.033	-0.153
Q9UKY7	CDV3	1.671	1.560	1.748	1.690	1.615	1.719	0.278	0.104
P51610	HCFC1	-1.281	-1.269	-1.221	-1.193	-1.275	-1.207	0.088	0.068
Q8IV56	PRR15	-2.584	-2.180	-2.627	-2.583	-2.382	-2.605	0.468	-0.223
A1L0T0	ILVBL	2.662	2.601	2.752	2.728	2.631	2.740	0.141	0.109
P32456	GBP2	-1.237	-1.274	-0.035	-0.119	-1.255	-0.077	0.009	1.179
Q6BDS2	UHRF1BP1	0.939	0.954	0.983	1.088	0.947	1.036	0.334	0.089
Q05513	PRKCZ	-3.387	-3.022	-3.408	-2.617	-3.205	-3.012	0.717	0.192
O43829	ZBTB14	-0.788	-0.636	-0.666	-0.496	-0.712	-0.581	0.370	0.131
Q96EX3	DYNC2I2	-0.224	-0.185	-0.452	-0.344	-0.204	-0.398	0.142	-0.194
Q8WW22	DNAJA4	-4.198	-3.998	-4.383	-3.945	-4.098	-4.164	0.820	-0.066
Q92791	P3H4	-1.142	-1.254	-1.444	-1.479	-1.198	-1.461	0.110	-0.263

O75390	CS	5.263	5.194	5.409	5.373	5.229	5.391	0.085	0.162
Q6NW29	RWDD4	-0.808	-0.894	-0.744	-0.802	-0.851	-0.773	0.288	0.078
Q9Y467	SALL2	-1.523	-1.648	-1.575	-1.586	-1.585	-1.580	0.952	0.005
Q6IPU0	CENPP	-1.945	-1.883	-1.894	-1.841	-1.914	-1.868	0.373	0.047
O00273	DFFA	1.895	1.830	1.945	1.999	1.863	1.972	0.127	0.110
Q15650	TRIP4	1.052	1.138	1.029	1.180	1.095	1.104	0.925	0.010
O96019	ACTL6A	-0.393	-0.231	-0.411	-0.384	-0.312	-0.398	0.478	-0.085
O75348	ATP6V1G1	1.775	1.730	1.724	1.754	1.753	1.739	0.668	-0.014
Q9NXG2	THUMPD1	2.673	2.672	2.740	2.709	2.673	2.725	0.185	0.052
Q9BSU1	PHAF1	-2.082	-2.169	-2.180	-2.112	-2.126	-2.146	0.744	-0.021
P13674	P4HA1	-3.000	-2.768	-2.970	-2.744	-2.884	-2.857	0.884	0.027
O60307	MAST3	-2.978	-2.985	-3.144	-3.023	-2.982	-3.083	0.341	-0.102
P08579	SNRPB2	3.696	3.745	3.750	3.751	3.720	3.750	0.434	0.030
P62304	SNRPE	3.230	3.149	3.126	3.194	3.189	3.160	0.640	-0.029
P53007	SLC25A1	4.116	4.079	4.161	4.165	4.098	4.163	0.169	0.065
O60934	NBN	2.204	2.207	2.197	2.165	2.206	2.181	0.364	-0.025
O43181	NDUFS4	1.014	0.959	1.034	1.154	0.987	1.094	0.295	0.107
Q96S15	WDR24	-1.228	-1.248	-1.182	-1.181	-1.238	-1.182	0.114	0.056
P42680	TEC	-3.463	-3.710	-3.299	-3.445	-3.587	-3.372	0.300	0.214
Q9BYJ9	YTHDF1	0.451	0.395	0.480	0.370	0.423	0.425	0.984	0.001
Q7Z569	BRAP	-0.535	-0.588	-0.706	-0.646	-0.562	-0.676	0.105	-0.114
Q96C00	ZBTB9	-2.094	-1.987	-1.936	-2.026	-2.040	-1.981	0.489	0.059
P61978	HNRNPK	1.549	1.478	1.424	1.422	1.513	1.423	0.239	-0.090
P09012	SNRPA	3.629	3.450	3.644	3.605	3.540	3.625	0.513	0.085
P49721	PSMB2	3.711	3.632	3.731	3.613	3.672	3.672	0.997	0.000
Q15397	PUM3	3.427	3.394	3.455	3.449	3.411	3.452	0.236	0.041
Q9BUL8	PDCD10	2.887	2.874	3.414	3.399	2.881	3.406	0.000	0.526
P17096	HMGA1	3.394	3.172	3.279	3.272	3.283	3.276	0.961	-0.007
Q9H9A6	LRRC40	3.843	3.780	3.761	3.733	3.811	3.747	0.258	-0.064
Q9GZP8	IMUP	0.568	0.443	0.585	0.436	0.505	0.510	0.965	0.005
O95025	SEMA3D	-1.456	-1.588	-1.611	-1.654	-1.522	-1.632	0.324	-0.110
Q9BPX5	ARPC5L	2.866	2.833	2.926	2.825	2.849	2.876	0.695	0.026
P35250	RFC2	-1.567	-1.474	-1.599	-1.560	-1.521	-1.580	0.408	-0.059
Q9BU61	NDUFAF3	-0.979	-1.006	-0.899	-0.944	-0.993	-0.921	0.141	0.071
A6NDG6	PGP	2.166	2.191	2.086	2.170	2.179	2.128	0.433	-0.050

Q9Y291	MRPS33	1.618	1.604	1.469	1.550	1.611	1.510	0.233	-0.101
P48637	GSS	2.201	2.167	2.112	2.146	2.184	2.129	0.150	-0.055
O75153	CLUH	4.713	4.668	4.479	4.475	4.690	4.477	0.063	-0.213
P56962	STX17	-0.337	-0.197	-0.294	-0.141	-0.267	-0.217	0.682	0.049
O76070	SNCG	-1.482	-1.537	-0.766	-1.253	-1.509	-1.010	0.285	0.500
Q99459	CDC5L	4.071	4.034	4.109	4.068	4.052	4.088	0.324	0.036
Q96EP5	DAZAP1	-2.752	-2.868	-3.157	-2.703	-2.810	-2.930	0.691	-0.120
Q96HP0	DOCK6	1.696	1.670	1.747	1.813	1.683	1.780	0.173	0.097
Q00341	HDLBP	3.289	3.126	3.262	3.149	3.208	3.206	0.985	-0.002
Q9UKG1	APPL1	2.043	2.004	2.114	2.099	2.023	2.106	0.109	0.083
P22626	HNRNPA2B1	2.072	2.043	2.074	2.087	2.057	2.081	0.338	0.023
Q9BRK4	LZTS2	-2.147	-2.264	-2.205	-2.253	-2.206	-2.229	0.760	-0.024
Q9NZB2	FAM120A	-3.329	-3.269	-3.435	-3.380	-3.299	-3.408	0.116	-0.109
P17568	NDUFB7	1.411	1.319	1.451	1.462	1.365	1.457	0.290	0.092
O43809	NUDT21	4.341	4.351	4.330	4.366	4.346	4.348	0.938	0.002
Q8N2G6	ZCCHC24	-2.047	-2.036	-2.083	-2.104	-2.041	-2.094	0.080	-0.052
Q53LP3	SOWAHC	-0.578	-0.457	-0.573	-0.551	-0.517	-0.562	0.593	-0.045
Q9ULV0	MYO5B	-2.003	-1.849	-2.000	-1.852	-1.926	-1.926	1.000	0.000
O60503	ADCY9	-2.425	-2.574	-2.501	-2.354	-2.500	-2.428	0.565	0.072
Q96DM3	RMC1	-1.746	-1.768	-1.691	-1.636	-1.757	-1.664	0.147	0.094
P63027	VAMP2	-0.635	-0.458	-0.756	-0.545	-0.547	-0.651	0.531	-0.104
P16930	FAH	-2.403	-2.158	-2.087	-2.078	-2.280	-2.082	0.352	0.198
Q8NC60	NOA1	1.177	1.218	0.993	0.962	1.197	0.978	0.017	-0.220
Q8WXA9	SREK1	0.926	0.965	0.925	0.897	0.945	0.911	0.293	-0.035
P47929	LGALS7	-0.826	-0.274	-0.541	-0.463	-0.550	-0.502	0.891	0.048
Q9GZU5	NYX	-3.220	-3.042	-3.194	-3.253	-3.131	-3.223	0.482	-0.092
Q9NSA3	CTNNBIP1	-3.215	-3.108	-3.290	-3.218	-3.162	-3.254	0.306	-0.093
Q16527	CSRP2	-1.862	-1.975	-1.901	-1.982	-1.919	-1.942	0.775	-0.023
O75531	BANF1	2.394	2.637	2.653	2.540	2.515	2.597	0.626	0.082
P33992	MCM5	5.830	5.838	5.736	5.731	5.834	5.733	0.005	-0.101
Q9Y295	DRG1	3.687	3.669	3.573	3.636	3.678	3.605	0.241	-0.073
Q9ULM6	CNOT6	-2.022	-2.075	-2.168	-2.165	-2.048	-2.167	0.139	-0.118
Q15800	MSMO1	-3.455	-3.024	-3.139	-3.151	-3.239	-3.145	0.738	0.094
Q8IVB5	LIX1L	-1.642	-1.798	-1.709	-1.641	-1.720	-1.675	0.674	0.045
Q96PZ2	FAM111A	-0.864	-0.717	-0.886	-0.838	-0.791	-0.862	0.503	-0.071

P86452	ZBED6	-4.880	-5.100	-5.022	-4.939	-4.990	-4.981	0.947	0.009
Q9BQ61	TRIR	2.913	2.878	2.846	2.841	2.895	2.843	0.197	-0.052
Q9P0P0	RNF181	-1.203	-1.129	-1.234	-1.021	-1.166	-1.128	0.784	0.038
Q9Y366	IFT52	-0.708	-0.735	-0.760	-0.668	-0.721	-0.714	0.899	0.007
O75063	FAM20B	0.395	0.332	0.326	0.264	0.363	0.295	0.263	-0.069
Q6PD62	CTR9	3.421	3.431	3.355	3.391	3.426	3.373	0.189	-0.053
Q7L2E3	DHX30	-3.594	-3.414	-3.593	-3.489	-3.504	-3.541	0.760	-0.037
Q92522	H1	3.524	3.605	3.871	3.831	3.564	3.851	0.050	0.286
Q0VGL1	LAMTOR4	-0.233	-0.315	-0.103	-0.267	-0.274	-0.185	0.465	0.089
Q9NXD2	MTMR10	-5.585	-5.540	-5.434	-5.393	-5.562	-5.414	0.040	0.149
Q13501	SQSTM1	-0.190	0.003	0.299	0.281	-0.094	0.290	0.154	0.383
Q9Y5Y2	NUBP2	0.594	0.547	0.474	0.529	0.570	0.502	0.203	-0.069
Q13574	DGKZ	4.539	4.498	4.628	4.568	4.518	4.598	0.172	0.080
Q9NWT1	PAK1IP1	2.578	2.551	2.549	2.557	2.565	2.553	0.541	-0.012
Q96B26	EXOSC8	2.155	2.084	2.130	2.059	2.120	2.095	0.664	-0.025
P25963	NFKBIA	-1.092	-1.110	-1.198	-1.185	-1.101	-1.192	0.017	-0.091
Q99615	DNAJC7	1.671	1.591	1.639	1.493	1.631	1.566	0.538	-0.065
Q8N163	CCAR2	-0.580	-0.687	-0.559	-0.585	-0.633	-0.572	0.450	0.062
Q13873	BMPR2	-5.014	-4.950	-4.804	-4.845	-4.982	-4.825	0.071	0.157
Q92530	PSMF1	2.844	2.782	2.693	2.669	2.813	2.681	0.112	-0.132
P35052	GPC1	-1.257	-1.321	-1.164	-1.323	-1.289	-1.243	0.670	0.046
Q1KMD3	HNRNPUL2	4.857	4.865	4.927	4.864	4.861	4.895	0.471	0.034
P30408	TM4SF1	0.093	0.184	0.424	0.292	0.138	0.358	0.127	0.219
P39748	FEN1	1.455	1.692	1.541	1.550	1.573	1.546	0.854	-0.028
Q9UKR5	ERG28	-0.725	-0.671	-0.757	-0.806	-0.698	-0.781	0.154	-0.083
Q9BSF4	TIMM29	0.413	0.442	0.413	0.404	0.428	0.408	0.395	-0.019
P46783	RPS10	3.999	3.836	4.002	3.864	3.918	3.933	0.900	0.015
Q8WUY8	NAT14	0.306	0.366	0.218	0.362	0.336	0.290	0.638	-0.046
Q6PJG6	BRAT1	1.005	1.006	0.750	0.783	1.006	0.766	0.043	-0.240
P36954	POLR2I	-0.509	-0.684	-0.445	-0.495	-0.596	-0.470	0.374	0.126
O14657	TOR1B	1.213	1.170	1.262	1.230	1.191	1.246	0.190	0.054
P49023	PXN	-2.567	-2.487	-2.885	-2.693	-2.527	-2.789	0.186	-0.262
P10606	COX5B	2.714	2.757	2.757	2.765	2.735	2.761	0.435	0.026
O75394	MRPL33	1.004	1.039	1.001	0.998	1.022	1.000	0.427	-0.022
P55084	HADHB	0.740	0.631	0.986	0.884	0.686	0.935	0.079	0.249

Q9NW82	WDR70	2.961	2.837	2.841	2.830	2.899	2.836	0.490	-0.063
Q9Y6Y0	IVNS1ABP	0.274	0.205	0.219	0.247	0.240	0.233	0.886	-0.006
A8MW92	PHF20L1	-2.742	-2.787	-2.618	-2.571	-2.764	-2.594	0.035	0.170
Q9UQR1	ZNF148	0.787	0.738	0.717	0.708	0.762	0.713	0.286	-0.050
Q8NCW5	NAXE	1.424	1.379	1.441	1.483	1.401	1.462	0.188	0.061
Q9NPD8	UBE2T	-2.136	-1.892	-1.986	-1.928	-2.014	-1.957	0.722	0.057
P61081	UBE2M	3.824	3.655	3.885	3.773	3.739	3.829	0.482	0.089
Q12962	TAF10	0.604	0.624	0.608	0.524	0.614	0.566	0.451	-0.048
Q5TA50	CPTP	-1.185	-1.216	-1.187	-1.109	-1.201	-1.148	0.387	0.053
Q03393	PTS	-1.309	-1.420	-1.293	-1.352	-1.365	-1.322	0.587	0.043
O14810	CPLX1	-3.000	-3.131	-2.924	-2.957	-3.066	-2.941	0.294	0.125
Q6ZWT7	MBOAT2	-1.598	-1.464	-1.552	-1.437	-1.531	-1.494	0.718	0.037
Q5T447	HECTD3	0.639	0.598	0.594	0.656	0.618	0.625	0.878	0.007
P05997	COL5A2	0.604	0.523	0.443	0.479	0.563	0.461	0.198	-0.103
P23921	RRM1	5.040	4.973	4.725	4.773	5.007	4.749	0.030	-0.258
Q9P086	MED11	-2.043	-2.034	-2.231	-1.939	-2.039	-2.085	0.804	-0.046
Q96MW5	COG8	0.299	0.276	0.313	0.304	0.288	0.309	0.301	0.021
P07339	CTSD	4.551	4.799	4.772	4.741	4.675	4.756	0.629	0.082
Q9GZS1	POLR1E	-0.134	-0.122	-0.180	-0.007	-0.128	-0.094	0.758	0.035
Q9HB20	PLEKHA3	-1.940	-1.973	-1.949	-1.887	-1.957	-1.918	0.411	0.039
Q8NC42	RNF149	-3.324	-3.509	-3.272	-3.219	-3.416	-3.246	0.299	0.171
Q7Z388	DPY19L4	-2.022	-1.961	-2.024	-2.003	-1.991	-2.013	0.604	-0.022
Q14165	MLEC	3.567	3.533	3.561	3.546	3.550	3.554	0.863	0.004
Q9NZJ5	EIF2AK3	-2.917	-2.954	-2.966	-2.954	-2.935	-2.960	0.401	-0.025
Q8NEF9	SRFBP1	1.924	1.840	1.914	1.843	1.882	1.878	0.952	-0.004
Q9UL45	BLOC1S6	-2.962	-3.081	-2.925	-2.995	-3.021	-2.960	0.486	0.061
P42773	CDKN2C	0.456	0.435	0.186	0.285	0.445	0.235	0.136	-0.210
P19484	TFEB	-1.909	-2.107	-1.843	-2.165	-2.008	-2.004	0.985	0.004
Q969F1	GTF3C6	-2.630	-2.657	-2.752	-2.707	-2.643	-2.730	0.106	-0.086
Q7L311	ARMCX2	-0.303	-0.329	-0.530	-0.478	-0.316	-0.504	0.046	-0.188
Q86Y82	STX12	1.655	1.650	1.435	1.430	1.653	1.433	0.000	-0.220
P42285	MTREX	4.588	4.546	4.590	4.545	4.567	4.567	0.988	0.001
P09758	TACSTD2	0.055	0.336	0.359	-0.178	0.195	0.091	0.772	-0.104
Q8WTT2	NOC3L	4.247	4.224	4.245	4.287	4.235	4.266	0.360	0.031
O60613	SELENOF	-0.190	-0.287	-0.316	-0.172	-0.238	-0.244	0.958	-0.005

Q7L8L6	FASTKD5	1.628	1.625	1.499	1.558	1.627	1.529	0.187	-0.098
P14923	JUP	1.892	2.635	2.312	2.252	2.264	2.282	0.968	0.018
Q5TF58	IFFO2	-3.653	-3.563	-3.413	-3.386	-3.608	-3.399	0.113	0.208
Q96CW1	AP2M1	-2.545	-2.621	-2.603	-2.634	-2.583	-2.619	0.515	-0.035
Q9UBI1	COMMD3	-0.630	-0.386	-0.510	-0.260	-0.508	-0.385	0.556	0.123
Q9P270	SLAIN2	1.592	1.542	1.429	1.465	1.567	1.447	0.069	-0.120
Q8NFJ5	GPRC5A	4.182	4.194	3.995	4.077	4.188	4.036	0.162	-0.152
Q96G42	KLHDC7B	-3.194	-3.244	-2.628	-2.664	-3.219	-2.646	0.004	0.573
Q9Y6H1	CHCHD2	1.423	1.540	1.397	1.285	1.482	1.341	0.226	-0.140
Q9H0D6	XRN2	0.537	0.434	0.447	0.429	0.486	0.438	0.520	-0.048
Q9Y6Y8	SEC23IP	1.190	1.237	1.041	1.013	1.213	1.027	0.036	-0.186
O75356	ENTPD5	-1.453	-1.462	-1.444	-1.446	-1.457	-1.445	0.213	0.012
Q7L5Y9	MAEA	-0.446	-0.456	-0.593	-0.507	-0.451	-0.550	0.256	-0.100
Q6ZRP7	QSOX2	2.149	2.217	2.221	2.270	2.183	2.246	0.288	0.062
O43286	B4GALT5	-3.737	-4.352	-4.149	-3.811	-4.044	-3.980	0.876	0.064
P18827	SDC1	-4.273	-4.426	-4.024	-4.191	-4.350	-4.107	0.167	0.242
Q9Y6A4	CFAP20	2.196	2.081	2.077	2.030	2.138	2.053	0.359	-0.085
P35914	HMGCL	1.230	1.226	1.280	1.260	1.228	1.270	0.138	0.042
Q9P0T4	ZNF581	-3.817	-3.812	-3.928	-3.811	-3.815	-3.870	0.521	-0.055
Q9H081	MIS12	-0.430	-0.350	-0.594	-0.531	-0.390	-0.563	0.082	-0.172
Q9NY74	ETAA1	-3.504	-3.495	-3.510	-3.406	-3.499	-3.458	0.569	0.041
Q9H6F5	CCDC86	1.486	1.513	1.456	1.514	1.500	1.485	0.712	-0.014
O75955	FLOT1	0.154	0.117	0.121	0.104	0.135	0.112	0.421	-0.023
Q16762	TST	2.451	2.483	2.608	2.613	2.467	2.611	0.064	0.144
Q9GZP1	NRSN2	-2.902	-3.042	-2.867	-2.688	-2.972	-2.778	0.236	0.194
Q99856	ARID3A	-1.192	-1.233	-1.222	-1.196	-1.212	-1.209	0.907	0.003
Q9BXL7	CARD11	-2.060	-2.108	-2.074	-1.980	-2.084	-2.027	0.423	0.057
Q15365	PCBP1	5.409	5.380	5.303	5.324	5.395	5.314	0.055	-0.081
Q8N5G2	MACO1	-1.610	-1.743	-1.848	-1.851	-1.677	-1.849	0.234	-0.173
Q96JH7	VCPIP1	2.035	2.162	2.119	2.196	2.098	2.158	0.524	0.059
Q5R3I4	TTC38	0.165	0.043	0.081	0.093	0.104	0.087	0.830	-0.017
P49588	AARS1	3.671	3.665	3.752	3.763	3.668	3.757	0.015	0.089
Q9BRT6	LLPH	1.923	2.060	2.035	1.956	1.992	1.995	0.968	0.004
P98170	XIAP	0.814	0.724	0.895	0.861	0.769	0.878	0.221	0.109
Q9NWZ3	IRAK4	-1.536	-1.482	-1.334	-1.351	-1.509	-1.343	0.080	0.166

Q14011	CIRBP	-3.813	-3.870	-4.084	-4.002	-3.841	-4.043	0.069	-0.201
Q5QP82	DCAF10	-4.235	-4.265	-4.052	-4.062	-4.250	-4.057	0.032	0.193
Q8NEW0	SLC30A7	0.565	0.195	0.532	0.163	0.380	0.348	0.913	-0.032
Q9Y2W3	SLC45A1	-2.521	-2.705	-2.321	-2.271	-2.613	-2.296	0.159	0.317
P61626	LYZ	-2.047	-1.646	-2.120	-1.936	-1.846	-2.028	0.526	-0.182
Q9NTN3	SLC35D1	-0.349	-0.283	-0.170	-0.163	-0.316	-0.167	0.135	0.149
A6NIH7	UNC119B	1.279	1.302	1.109	1.052	1.290	1.081	0.053	-0.210
P50395	GDI2	0.244	0.118	0.055	0.002	0.181	0.029	0.213	-0.152
Q9BWM7	SFXN3	-0.873	-0.612	-0.827	-0.936	-0.742	-0.882	0.469	-0.140
Q01650	SLC7A5	1.263	1.269	1.307	1.335	1.266	1.321	0.142	0.055
Q08AN1	ZNF616	-3.859	-4.067	-4.162	-3.994	-3.963	-4.078	0.483	-0.115
P17301	ITGA2	0.989	1.228	1.392	0.933	1.108	1.162	0.859	0.054
Q9Y333	LSM2	2.451	2.424	2.495	2.389	2.438	2.442	0.946	0.005
Q8TBP6	SLC25A40	-0.019	0.151	-0.006	0.016	0.066	0.005	0.603	-0.061
P46527	CDKN1B	-0.444	-0.537	0.042	-0.027	-0.490	0.008	0.017	0.498
Q6PCE3	PGM2L1	0.683	0.880	0.812	0.807	0.782	0.810	0.825	0.028
Q15545	TAF7	0.395	0.376	0.517	0.478	0.386	0.497	0.069	0.111
O43462	MBTPS2	-1.024	-1.011	-1.009	-1.072	-1.018	-1.040	0.600	-0.023
P30101	PDIA3	6.141	6.121	6.193	6.158	6.131	6.176	0.190	0.045
Q9BQ69	MACROD1	2.927	2.927	3.301	3.313	2.927	3.307	0.010	0.380
Q14149	MORC3	2.215	2.185	2.114	2.107	2.200	2.110	0.094	-0.089
Q8IYB1	MB21D2	-0.247	-0.271	-0.451	-0.291	-0.259	-0.371	0.390	-0.112
P45880	VDAC2	0.920	0.802	1.036	0.851	0.861	0.943	0.541	0.083
P62917	RPL8	4.971	4.897	4.999	4.961	4.934	4.980	0.416	0.046
Q6IAA8	LAMTOR1	1.299	1.340	1.274	1.345	1.320	1.310	0.836	-0.010
Q9BTT6	LRRC1	-4.344	-3.410	-4.125	-4.086	-3.877	-4.105	0.710	-0.228
Q9H792	PEAK1	-0.506	-0.509	-0.567	-0.526	-0.508	-0.547	0.308	-0.039
Q96MT3	PRICKLE1	-6.047	-6.222	-6.124	-6.203	-6.134	-6.164	0.801	-0.029
Q6NZI2	CAVIN1	2.712	2.656	2.511	2.452	2.684	2.482	0.038	-0.202
Q9BPY3	FAM118B	0.989	0.941	0.974	0.965	0.965	0.969	0.881	0.005
P19623	SRM	5.293	5.180	5.196	5.214	5.236	5.205	0.675	-0.032
Q01780	EXOSC10	-0.566	-0.158	-0.635	-0.172	-0.362	-0.403	0.906	-0.041
P78527	PRKDC	3.020	2.970	3.006	3.018	2.995	3.012	0.627	0.017
Q96FX7	TRMT61A	1.582	1.691	1.619	1.662	1.636	1.641	0.948	0.005
Q9NUU7	DDX19A	1.538	1.418	1.456	1.388	1.478	1.422	0.520	-0.056

O43325	LYRM1	-2.022	-1.994	-1.896	-1.718	-2.008	-1.807	0.259	0.201
Q9BWH2	FUNDC2	0.860	0.917	0.909	0.990	0.888	0.950	0.352	0.061
P12259	F5	1.764	1.771	2.108	2.084	1.767	2.096	0.013	0.329
P46821	MAP1B	0.589	0.558	1.340	0.911	0.574	1.126	0.235	0.552
Q96GM8	TOE1	-2.901	-2.995	-3.156	-2.945	-2.948	-3.050	0.503	-0.102
Q8NCN5	PDPR	2.289	2.255	2.601	2.570	2.272	2.585	0.005	0.313
Q9H0H0	INTS2	0.751	0.718	0.734	0.760	0.735	0.747	0.625	0.012
Q8IYI6	EXOC8	3.033	2.985	3.004	2.945	3.009	2.975	0.469	-0.034
P07237	P4HB	7.284	7.286	7.315	7.211	7.285	7.263	0.745	-0.022
P30041	PRDX6	5.302	5.129	5.312	5.248	5.215	5.280	0.592	0.064
Q9BYG5	PARD6B	-0.869	-0.640	-0.685	-0.712	-0.755	-0.699	0.709	0.056
P23025	XPA	-0.801	-0.833	-0.912	-0.830	-0.817	-0.871	0.395	-0.054
Q96I34	PPP1R16A	-1.774	-1.742	-1.604	-1.554	-1.758	-1.579	0.039	0.179
P30084	ECHS1	5.572	5.491	5.553	5.518	5.531	5.536	0.933	0.004
Q9H6U6	BCAS3	-3.402	-3.445	-3.334	-3.390	-3.424	-3.362	0.233	0.061
Q13489	BIRC3	-5.995	-6.141	-6.236	-6.207	-6.068	-6.222	0.272	-0.154
Q5SXM2	SNAPC4	-1.053	-1.106	-1.103	-1.094	-1.080	-1.099	0.608	-0.019
Q96S97	MYADM	2.516	2.396	2.508	2.564	2.456	2.536	0.390	0.080
Q9P215	POGK	-0.422	-0.441	-0.426	-0.413	-0.432	-0.419	0.398	0.012
P18074	ERCC2	0.701	0.673	0.754	0.767	0.687	0.760	0.075	0.073
P61160	ACTR2	2.804	2.870	3.204	3.206	2.837	3.205	0.057	0.368
Q92733	PRCC	1.326	1.228	1.416	1.253	1.277	1.334	0.621	0.057
Q6ZSY5	PPP1R3F	-3.689	-3.687	-3.556	-3.544	-3.688	-3.550	0.027	0.138
Q9ULJ7	ANKRD50	-0.745	-0.788	-0.896	-0.877	-0.766	-0.886	0.076	-0.120
P31937	HIBADH	3.255	3.150	3.234	3.267	3.203	3.251	0.520	0.048
P33991	MCM4	5.388	5.404	5.245	5.236	5.396	5.240	0.011	-0.155
Q7Z449	CYP2U1	-1.983	-1.947	-1.950	-1.776	-1.965	-1.863	0.445	0.102
Q5T0B9	ZNF362	-1.729	-1.823	-1.691	-1.602	-1.776	-1.647	0.185	0.129
