# Supplementary material for: New Function Annotation of PROSER2 in Pancreatic Ductal Adenocarcinoma
Source: J Proteome Res. 2024 Jan 31;23(3):905–15. doi: 10.1021/acs.jproteome.3c00632 (PMC10913870; doi:10.1021/acs.jproteome.3c00632)
Supplement: Supplementary file 2 — pr3c00632_si_002.pdf [file pr3c00632_si_002.pdf]

Table S2. List of selected 97 proteins for STRING analysis (|log2FC| < 0.3 and p-value < 0.05)

Uniprot	Gene name	Protein abundances (They were log2-transformed and divided by reference value after normalization by column-median method)				Miapaca_C_avg	Miapaca_P_avg	P-value	log2FC(M_P/M_C)
		Miapaca_C_1	Miapaca_C_2	Miapaca_P_1	Miapaca_P_2				
Q8IXL6	FAM20C	2.267	2.064	-0.544	0.015	2.165	-0.265	0.048	-2.430
P04818	TYMS	-3.311	-3.265	-4.693	-4.630	-3.288	-4.662	0.001	-1.374
P08779	KRT16	-2.222	-2.193	-3.306	-3.261	-2.208	-3.283	0.002	-1.076
Q15847	ADIRF	3.041	2.905	2.131	2.206	2.973	2.168	0.020	-0.805
P02533	KRT14	-3.523	-3.632	-4.295	-4.315	-3.577	-4.305	0.041	-0.728
P04264	KRT1	2.826	2.771	2.076	2.084	2.799	2.080	0.021	-0.719
O94907	DKK1	-1.769	-1.695	-2.392	-2.341	-1.732	-2.367	0.008	-0.635
P06703	S100A6	4.740	4.722	4.135	4.115	4.731	4.125	0.001	-0.606
O60925	PFDN1	3.550	3.462	2.924	2.878	3.506	2.901	0.018	-0.604
Q13576	IQGAP2	1.081	1.008	0.452	0.483	1.044	0.467	0.019	-0.577
Q9BVA1	TUBB2B	-0.587	-0.703	-1.102	-1.256	-0.645	-1.179	0.036	-0.534
O00622	CCN1	1.573	1.487	0.982	1.045	1.530	1.013	0.014	-0.517
Q8WUM9	SLC20A1	0.161	0.117	-0.394	-0.319	0.139	-0.357	0.016	-0.496
Q9NX18	SDHAF2	0.521	0.458	-0.020	0.038	0.490	0.009	0.008	-0.481
P21980	TGM2	6.288	6.172	5.796	5.718	6.230	5.757	0.029	-0.473
Q9NYY3	PLK2	0.679	0.720	0.228	0.228	0.699	0.228	0.027	-0.472
P11137	MAP2	-0.950	-0.984	-1.385	-1.447	-0.967	-1.416	0.015	-0.449
P21579	SYT1	0.310	0.205	-0.215	-0.150	0.258	-0.183	0.030	-0.440
Q9H1E5	TMX4	0.768	0.774	0.324	0.370	0.771	0.347	0.032	-0.424
Q13885	TUBB2A	3.697	3.664	3.302	3.217	3.680	3.259	0.039	-0.421
P06493	CDK1	2.905	2.961	2.547	2.479	2.933	2.513	0.012	-0.420
P25116	F2R	-0.198	-0.268	-0.674	-0.619	-0.233	-0.646	0.014	-0.413
Q9Y547	HSPB11	-1.881	-1.771	-2.259	-2.182	-1.826	-2.220	0.036	-0.394
Q8WWN8	ARAP3	-3.208	-3.267	-3.627	-3.632	-3.237	-3.629	0.047	-0.392
Q9BRT9	GINS4	-0.466	-0.483	-0.874	-0.858	-0.475	-0.866	0.001	-0.391
Q14563	SEMA3A	1.260	1.139	0.780	0.865	1.199	0.823	0.046	-0.377
Q9HBM1	SPC25	1.944	1.908	1.541	1.560	1.926	1.550	0.010	-0.376
P40855	PEX19	-3.121	-3.147	-3.514	-3.500	-3.134	-3.507	0.006	-0.373
Q9BX63	BRIP1	-3.613	-3.626	-3.956	-4.012	-3.619	-3.984	0.038	-0.364
P12004	PCNA	6.242	6.223	5.901	5.843	6.233	5.872	0.035	-0.361
P98179	RBM3	1.997	2.003	1.627	1.660	2.000	1.643	0.025	-0.356
Q99988	GDF15	-0.465	-0.485	-0.810	-0.846	-0.475	-0.828	0.009	-0.353
P26358	DNMT1	-1.485	-1.537	-1.886	-1.822	-1.511	-1.854	0.016	-0.343
Q92887	ABCC2	-1.271	-1.331	-1.659	-1.621	-1.301	-1.640	0.018	-0.339
Q9Y6I9	TEX264	0.288	0.239	-0.047	-0.100	0.263	-0.073	0.012	-0.336
P31321	PRKAR1B	-1.741	-1.726	-2.090	-2.046	-1.733	-2.068	0.024	-0.335
Q6XZF7	DNMBP	2.197	2.153	1.829	1.857	2.175	1.843	0.011	-0.332
P42575	CASP2	-1.266	-1.222	-1.620	-1.530	-1.244	-1.575	0.048	-0.331
P07942	LAMB1	1.223	1.277	0.903	0.957	1.250	0.930	0.014	-0.320
Q07864	POLE	1.808	1.810	1.491	1.500	1.809	1.496	0.006	-0.313
O95239	KIF4A	-0.537	-0.496	-0.844	-0.796	-0.517	-0.820	0.011	-0.303
P02786	TFRC	6.789	6.763	6.472	6.480	6.776	6.476	0.017	-0.300
Q9BYK8	HELZ2	0.840	0.790	1.144	1.086	0.815	1.115	0.017	0.300
Q8NCN5	PDPR	2.289	2.255	2.601	2.570	2.272	2.585	0.005	0.313
P35237	SERPINB6	5.390	5.317	5.687	5.661	5.353	5.674	0.048	0.321
Q9Y3L5	RAP2C	-2.632	-2.530	-2.290	-2.223	-2.581	-2.257	0.046	0.324
P12259	F5	1.764	1.771	2.108	2.084	1.767	2.096	0.013	0.329
O00767	SCD	0.600	0.600	0.948	0.911	0.600	0.929	0.036	0.329
Q16763	UBE2S	0.017	0.026	0.353	0.363	0.021	0.358	0.000	0.337
P23381	WARS1	3.775	3.739	4.084	4.108	3.757	4.096	0.007	0.339
O14832	PHYH	-2.308	-2.358	-1.936	-2.007	-2.333	-1.971	0.019	0.361
P17275	JUNB	2.209	2.103	2.566	2.470	2.156	2.518	0.038	0.362
P50135	HNMT	-2.162	-2.112	-1.752	-1.790	-2.137	-1.771	0.010	0.366
O75190	DNAJB6	-1.976	-2.081	-1.692	-1.631	-2.029	-1.662	0.043	0.367
A6NGB9	WIPF3	-5.026	-4.937	-4.665	-4.564	-4.982	-4.614	0.033	0.367
Q9NRR8	CDC42SE1	-3.545	-3.545	-3.185	-3.168	-3.545	-3.176	0.014	0.369
Q9BQ69	MACROD1	2.927	2.927	3.301	3.313	2.927	3.307	0.010	0.380
Q9Y5Q0	FADS3	-3.216	-3.258	-2.824	-2.888	-3.237	-2.856	0.016	0.380
Q8NF37	LPCAT1	0.602	0.667	1.045	1.000	0.634	1.023	0.015	0.388
P69905	HBA1	-2.351	-2.264	-1.886	-1.951	-2.307	-1.919	0.024	0.389
Q15020	SART3	-1.016	-1.018	-0.636	-0.599	-1.017	-0.618	0.030	0.400
P04083	ANXA1	8.164	8.117	8.574	8.513	8.141	8.544	0.011	0.403
P30740	SERPINB1	3.138	3.084	3.549	3.494	3.111	3.521	0.009	0.411
O00443	PIK3C2A	1.835	1.786	2.260	2.198	1.811	2.229	0.011	0.418
Q00978	IRF9	0.021	0.021	0.463	0.425	0.021	0.444	0.029	0.423
Q08AF3	SLFN5	1.189	1.238	1.651	1.631	1.214	1.641	0.018	0.427
Q99541	PLIN2	2.268	2.187	2.636	2.675	2.228	2.655	0.028	0.427
P61129	ZC3H6	-1.973	-2.020	-1.559	-1.563	-1.996	-1.561	0.033	0.436
P49674	CSNK1E	-2.694	-2.743	-2.323	-2.240	-2.719	-2.282	0.021	0.437
Q9Y664	KPTN	-2.966	-2.973	-2.547	-2.509	-2.970	-2.528	0.023	0.442
Q8TDB6	DTX3L	1.400	1.341	1.839	1.793	1.370	1.816	0.009	0.445
P02656	APOC3	-3.731	-3.725	-3.291	-3.234	-3.728	-3.262	0.037	0.465
Q13287	NMI	1.081	0.956	1.530	1.442	1.019	1.486	0.033	0.467
Q16832	DDR2	-1.226	-1.247	-0.810	-0.719	-1.237	-0.764	0.049	0.472
Q9H1E3	NUCKS1	0.889	0.795	1.352	1.310	0.842	1.331	0.032	0.489
O00534	VWA5A	-3.064	-3.096	-2.546	-2.635	-3.080	-2.590	0.037	0.490
P46527	CDKN1B	-0.444	-0.537	0.042	-0.027	-0.490	0.008	0.017	0.498

Q9UII4	HERC5	-2.137	-2.295	-1.742	-1.653	-2.216	-1.698	0.048	0.519
Q7Z4F1	LRP10	-3.683	-3.818	-3.191	-3.267	-3.750	-3.229	0.038	0.521
Q9BUL8	PDCD10	2.887	2.874	3.414	3.399	2.881	3.406	0.000	0.526
Q8N9Z2	CCDC71L	0.143	0.044	0.662	0.608	0.093	0.635	0.023	0.542
O14879	IFIT3	0.639	0.552	1.192	1.091	0.596	1.141	0.016	0.545
P02749	APOH	-1.202	-1.173	-0.584	-0.652	-1.187	-0.618	0.017	0.569
Q96G42	KLHDC7B	-3.194	-3.244	-2.628	-2.664	-3.219	-2.646	0.004	0.573
Q9BZQ8	NIBAN1	1.915	1.795	2.457	2.399	1.855	2.428	0.032	0.574
Q9Y3E1	HDGFL3	-1.767	-1.896	-1.160	-1.304	-1.831	-1.232	0.026	0.599
P04004	VTN	0.446	0.277	1.082	0.966	0.361	1.024	0.031	0.663
Q9UJF2	RASAL2	-3.645	-3.596	-2.997	-2.853	-3.620	-2.925	0.045	0.695
Q9P0M6	MACROH2A2	1.078	0.965	1.763	1.727	1.022	1.745	0.034	0.724
P43490	NAMPT	5.820	5.672	6.637	6.509	5.746	6.573	0.015	0.827
Q15646	OASL	-1.098	-0.860	-0.193	-0.074	-0.979	-0.133	0.048	0.846
Q9BUT9	MCRIP2	-4.142	-4.059	-3.045	-3.097	-4.101	-3.071	0.005	1.030
P49716	CEBPD	-4.677	-4.831	-3.723	-3.505	-4.754	-3.614	0.018	1.140
P32456	GBP2	-1.237	-1.274	-0.035	-0.119	-1.255	-0.077	0.009	1.179
P22676	CALB2	3.108	3.296	4.455	4.345	3.202	4.400	0.016	1.198
O00506	STK25	-1.862	-1.857	-0.377	-0.423	-1.859	-0.400	0.009	1.460
Q86WR7	PROSER2	1.387	0.896	4.914	4.789	1.142	4.852	0.032	3.710
