# Supplementary material for: New Function Annotation of PROSER2 in Pancreatic Ductal Adenocarcinoma
Source: J Proteome Res. 2024 Jan 31;23(3):905–15. doi: 10.1021/acs.jproteome.3c00632 (PMC10913870; doi:10.1021/acs.jproteome.3c00632)
Supplement: Supplementary file 3 — pr3c00632_si_003.pdf [file pr3c00632_si_003.pdf]

Supplementary table S3. List of upregulated and downregulated proteins (|log2FC| < 0.5 and p-value < 0.05)

Uniprot	Gene name	Log_2FC	P-value
Q86WR7	PROSER2	3.71	3.2.E-02
O00506	STK25	1.46	9.2.E-03
P22676	CALB2	1.2	1.6.E-02
P32456	GBP2	1.18	8.6.E-03
P49716	CEBPD	1.14	1.8.E-02
Q9BUT9	MCRIP2	1.03	4.8.E-03
Q15646	OASL	0.85	4.8.E-02
P43490	NAMPT	0.83	1.5.E-02
Q9P0M6	MACROH2A[illegible]	0.72	3.4.E-02
Q9UJF2	RASAL2	0.7	4.5.E-02
P04004	VTN	0.66	3.1.E-02
Q9Y3E1	HDGFL3	0.6	2.6.E-02
Q9BZQ8	NIBAN1	0.57	3.2.E-02
Q96G42	KLHDC7B	0.57	4.3.E-03
P02749	APOH	0.57	1.7.E-02
O14879	IFIT3	0.55	1.6.E-02
Q8N9Z2	CCDC71L	0.54	2.3.E-02
Q9BUL8	PDCD10	0.53	4.0.E-04
Q7Z4F1	LRP10	0.52	3.8.E-02
Q9UII4	HERC5	0.52	4.8.E-02

Uniprot	Gene name	Log_2FC	P-value
Q8IXL6	FAM20C	-2.43	4.8.E-02
P04818	TYMS	-1.37	1.3.E-03
P08779	KRT16	-1.08	1.6.E-03
Q15847	ADIRF	-0.8	2.0.E-02
P02533	KRT14	-0.73	4.1.E-02
P04264	KRT1	-0.72	2.1.E-02
O94907	DKK1	-0.63	8.1.E-03
P06703	S100A6	-0.61	5.2.E-04
O60925	PFDN1	-0.6	1.8.E-02
Q13576	IQGAP2	-0.58	1.9.E-02
Q9BVA1	TUBB2B	-0.53	3.6.E-02
O00622	CCN1	-0.52	1.4.E-02
