# Supplementary material for: New Function Annotation of PROSER2 in Pancreatic Ductal Adenocarcinoma
Source: J Proteome Res. 2024 Jan 31;23(3):905–15. doi: 10.1021/acs.jproteome.3c00632 (PMC10913870; doi:10.1021/acs.jproteome.3c00632)
Supplement: Supplementary file 4 — pr3c00632_si_004.pdf [file pr3c00632_si_004.pdf]

Supporting Information

New Function Annotation of PROSER2 in Pancreatic Ductal Adenocarcinoma

Yu-Sun Lee[1,2], Jieun Im[1], Yeji Yang[3,8], Hea Ji Lee[3], Mi Rim Lee[4], Sang-Myung Woo[4,5], Sang-Jae Park[5,6], Sun-Young Kong[4,7], Jin Young Kim[3,8], Heeyoun Hwang[3,8], Yun-Hee Kim[1,4*]*

[1]Division of Convergence Technology, Research Institute of National Cancer Center, Goyang 10408, Republic of Korea

[2]Department of Biomedical Science, Graduate School, Kyung Hee University, Seoul 02447, Republic of Korea

[3]Research Center for Bioconvergence Analysis, Korea Basic Science Institute, Cheongju, 28119, Republic of Korea

[4]Department of Cancer Biomedical Science, National Cancer Center Graduate School of Cancer Science and Policy, Goyang 10408, Republic of Korea

[5]Department of Center for Liver and Pancreatobiliary Cancer, Nationa Cancer Center, Goyang 10408, Republic of Korea

[6]Department of Targeted Therapy Branch, Research Institute of National Cancer Center, Goyang 10408, Republic of Korea

[7]Critical Diseases Diagnostics Convergence Research Center, Korea Research Institute of Bioscience and Biotechnology, Daejeon, 34141, Republic of Korea

[8]Critical Diseases Diagnostics Convergence Research Center, Korea Research Institute of Bioscience and Biotechnology, Daejeon, 34141, Republic of Korea

Contents

Figure S1. The entire membrane of western blot analysis from Figure 2. (A) Evaluation of PROSER2 expression in PDAC cell lines and (B) PDOXc. (C) The validation of PROSER2 in PROSER2-overexpressing MIA PaCa-2 cell line, (D) PROSER2 knockdown SNU-213, and SNU-410 cell lines. 4

Figure S2. STRING analysis images and GO-term analysis. Bioinformatic analysis by STRING. The STRING analysis using Cytoscape 3.9.1 program to reveal function interaction between the deregulated proteins from identification proteins by LC-MS/MS data. Pathway list from the changed expression level of candidate protein by PROSER2. Red-colored circle indicates the apoptosis signaling pathway and closely related proteins with PROSER2. Blue-colored circle represents the regulation of cell migration and green-colored circle represents Type I interferon signaling pathway. Yellow-colored circle represents mitotic cell cycle. 5

Figure S3. The entire membrane of western blot analysis from Figure 3. Expression of STK25 and PDCD10 and demonstration of binding of PROSER2 with SKT25 and PDCD10 in PROSER2-overexpressing MIA PaCa-2 cells, as assessed using immunoprecipitation and western blot analyses, respectively. 6

Figure S4. The colocalization analysis between PROSER2 and STK25, PDCD10 in SNU-213 and SNU-410 cells. (A) Confocal micrographs of PROSER2 (green) and STK25 (red) and in SNU-213 and SNU-410 (scale bar = 5 μm). Graphs indicate the colocalization of PROSER2 (green) with STK25 (red) based on expression intensity. (B) Confocal micrographs of PROSER2 (green) and PDCD10 (red) in SNU-213 and SNU-410. Graphs indicate the colocalization of PROSER2 (green) with STK25 (red) based on expression intensity (scale bar = 5 μm). 7

Figure S5. The entire membrane of western blot analysis from Figure 5. (A) Expression of p-AMPK and PROSER2 in STK25-knockdown SNU-410 cells, as assessed using western blot analysis. (B) Expression of p-AMPK in PROSER2-overexpressing MIA PaCa-2 cells. 8

Contents of supporting information PDF file

Table S1. The identified and quantified proteins from large-scale proteogenomic research on PDAC.

Table S2. List of total proteins from PROSER2 overexpressed in MIA PaCa-2 cell line.

Table S3. List of selected 97 proteins for STRING analysis ($|log2FC| < 0.3$ and p-value < 0.05)

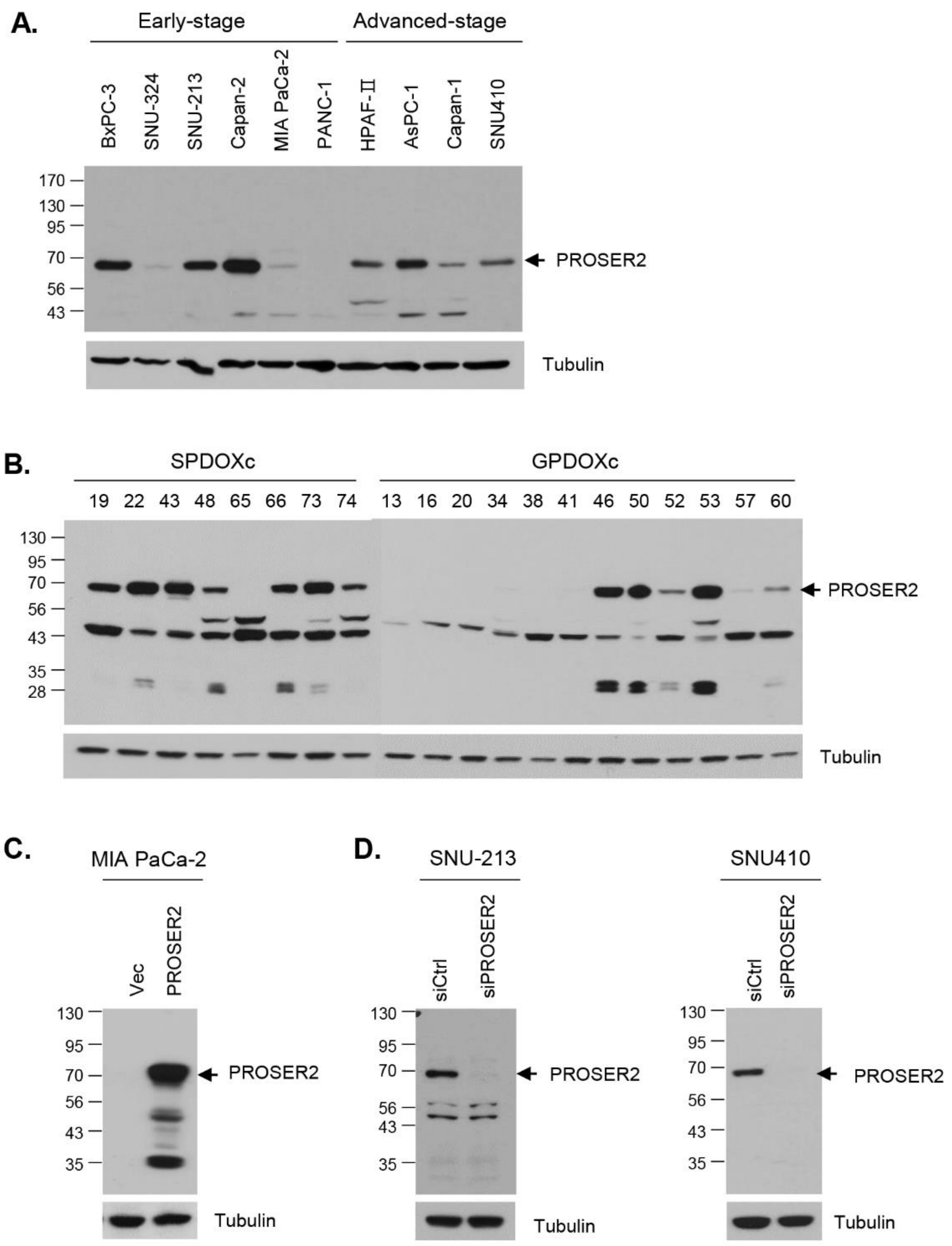

Figure S1. The entire membrane of western blot analysis from Figure 2. (A) Evaluation of PROSER2 expression in PDAC cell lines and (B) PDOXc. (C) The validation of PROSER2 in PROSER2-overexpressing MIA PaCa-2 cell line, (D) PROSER2 knockdown SNU-213, and SNU-410 cell lines.

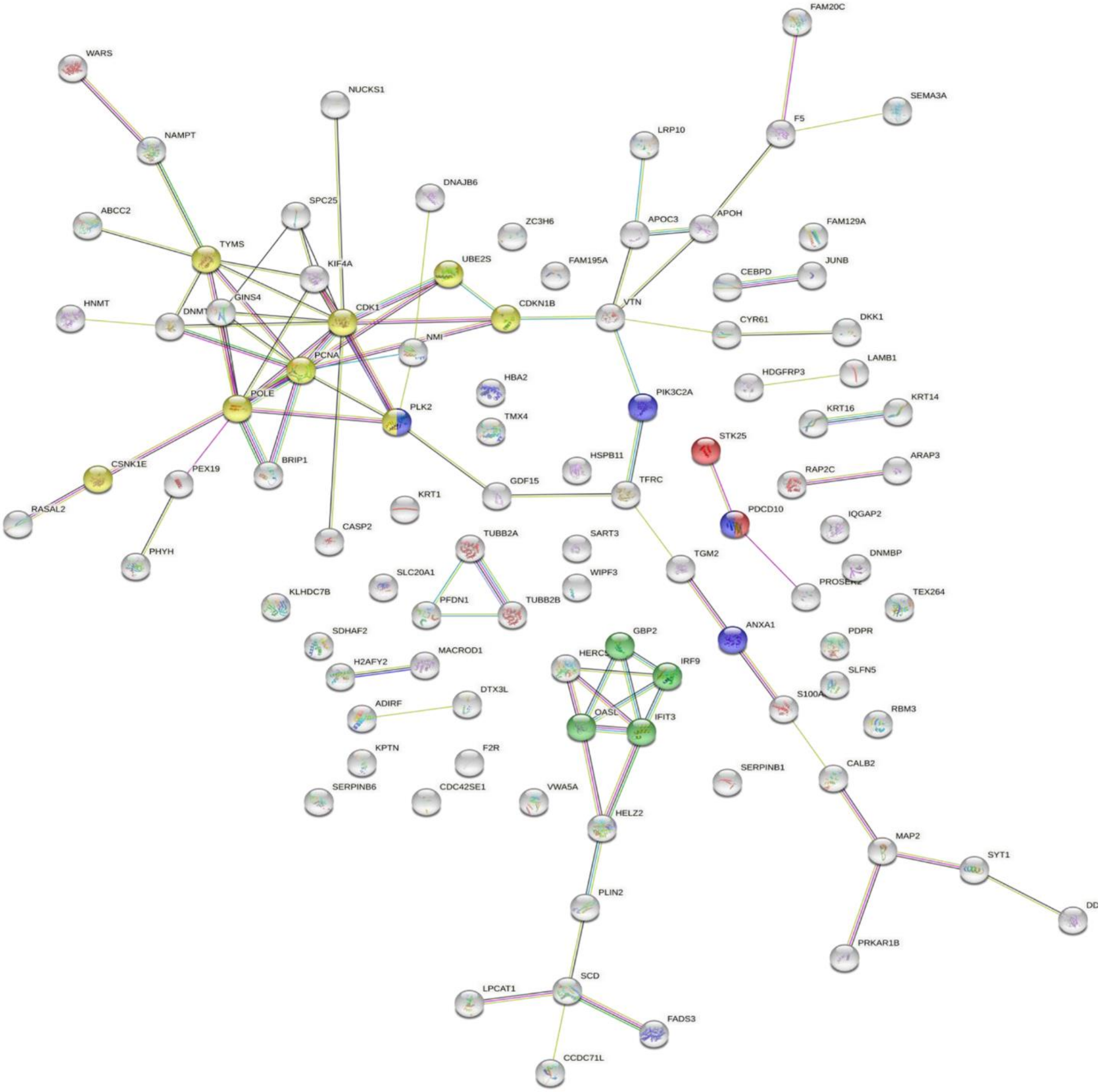

Figure S2. STRING analysis images and GO-term analysis. Bioinformatic analysis by STRING. The STRING analysis using Cytoscape 3.9.1 program to reveal function interaction between the deregulated proteins from identification proteins by LC-MS/MS data. Pathway list from the changed expression level of candidate protein by PROSER2. Red-colored circle indicates the apoptosis signaling pathway and closely related proteins with PROSER2. Blue-colored circle represents the regulation of cell migration and green-colored circle represents Type I interferon

signaling pathway. Yellow-colored circle represents mitotic cell cycle.

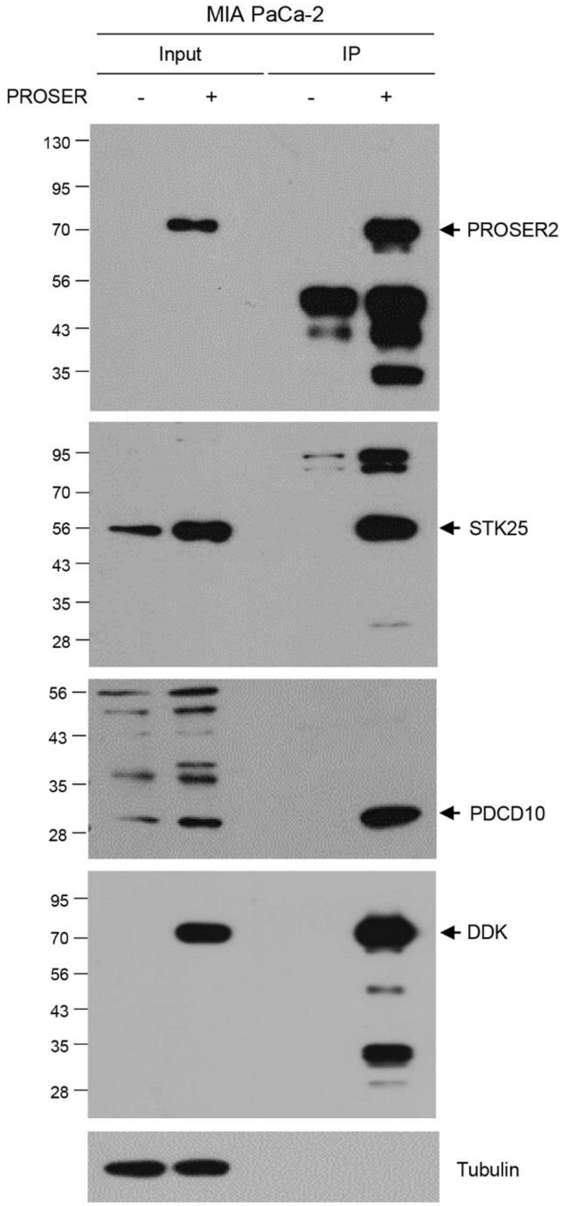

Figure S3. The entire membrane of western blot analysis from Figure 3. Expression of STK25 and PDCD10 and demonstration of binding of PROSER2 with SKT25 and PDCD10 in PROSER2-overexpressing MIA PaCa-2 cells, as assessed using immunoprecipitation and western blot analyses, respectively.

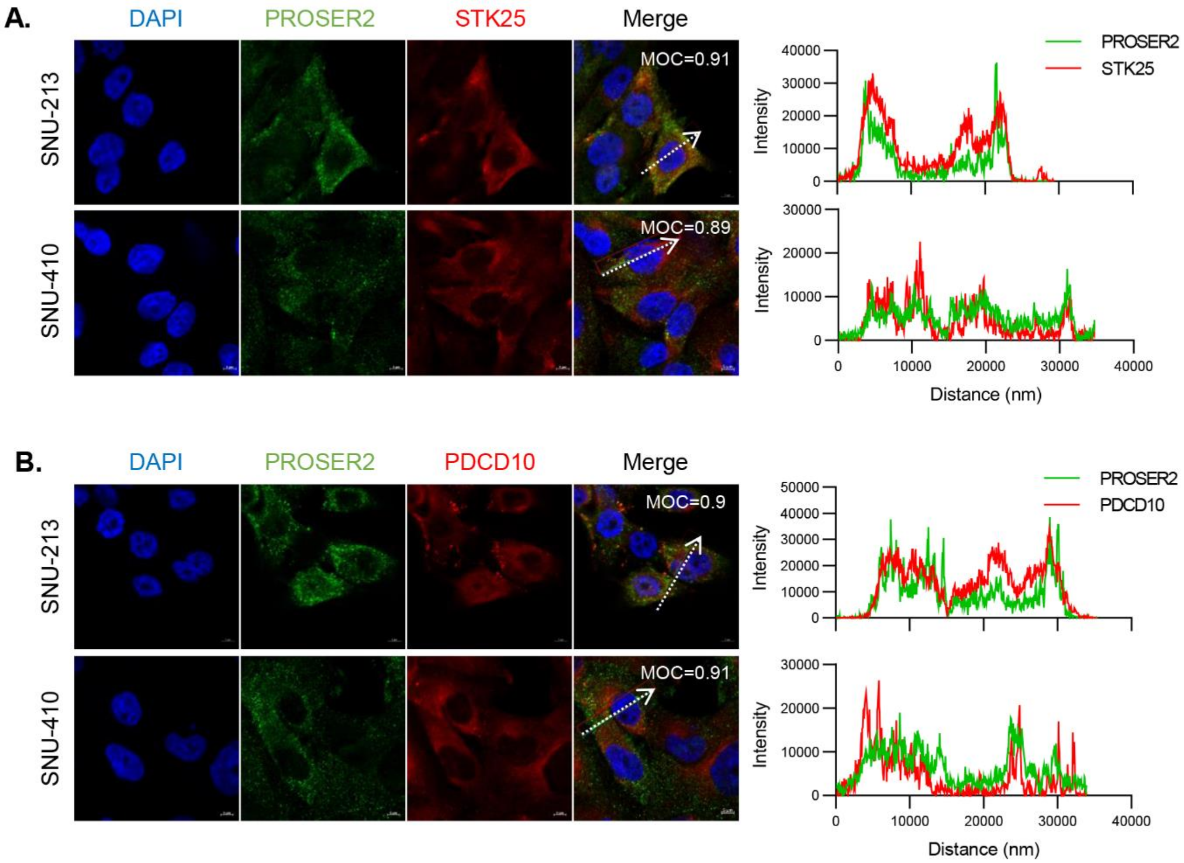

Figure S4. The colocalization analysis between PROSER2 and STK25, PDCD10 in SNU-213 and SNU-410 cells. (A) Confocal micrographs of PROSER2 (green) and STK25 (red) and in SNU-213 and SNU-410 (scale bar = 5 μm). Graphs indicate the colocalization of PROSER2 (green) with STK25 (red) based on expression intensity. (B) Confocal micrographs of PROSER2 (green) and PDCD10 (red) in SNU-213 and SNU-410. Graphs indicate the colocalization of PROSER2 (green) with PDCD10 (red) based on expression intensity (scale bar = 5 μm).

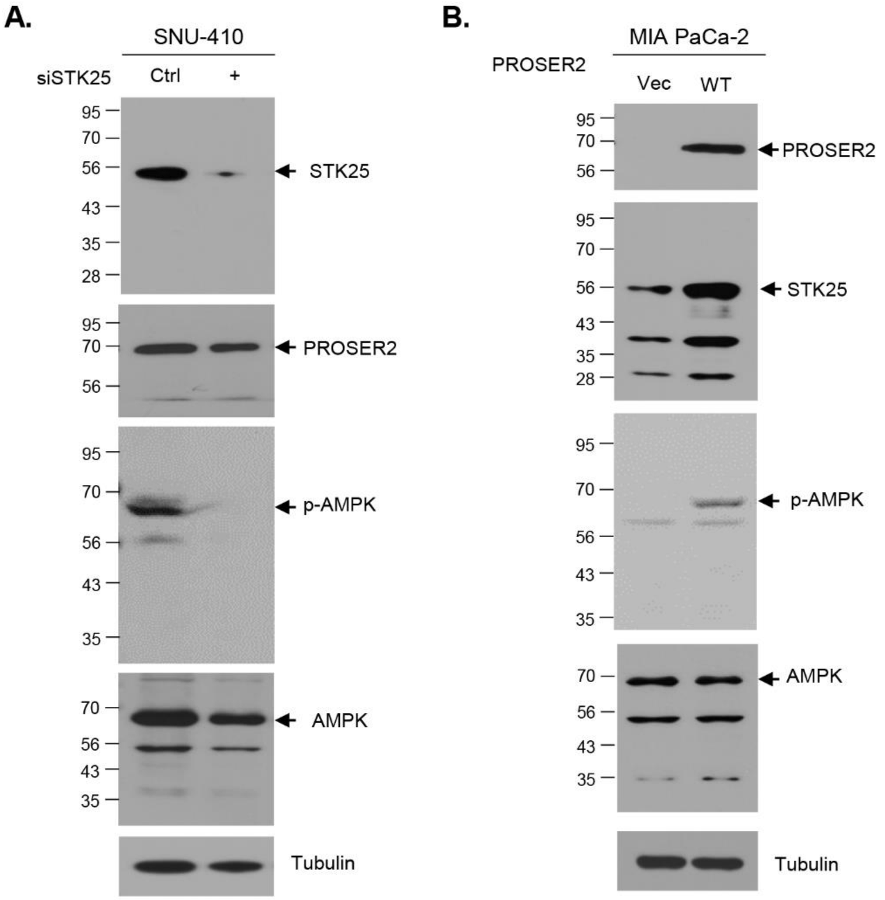

Figure S5. The entire membrane of western blot analysis from Figure 5. (A) Expression of p-AMPK and PROSER2 in STK25-knockdown SNU-410 cells, as assessed using western blot analysis. (B) Expression of p-AMPK in PROSER2-overexpressing MIA PaCa-2 cells.
